# Comprehensive UHPLC–Orbitrap–MS Profiling of Phloroglucinol α-Pyrones in *Helichrysum italicum* (Roth) G. Don (Asteraceae)

**DOI:** 10.3390/plants15172619

**Published:** 2026-08-27

**Authors:** Yulian Voynikov, Teodor Marinov, Paraskev Nedialkov

**Affiliations:** 1 Department of Chemistry, Faculty of Pharmacy, Medical University, 1000 Sofia, Bulgaria; 2 Department of Pharmacognosy, Faculty of Pharmacy, Medical University, 1000 Sofia, Bulgaria; t.marinov@pharmfac.mu-sofia.bg (T.M.); pnedialkov@pharmfac.mu-sofia.bg (P.N.)

**Keywords:** *Helichrysum italicum*, phloroglucinol α-pyrones, arzanol, UHPLC–HRMS, Orbitrap, metabolite profiling

## Abstract

*Helichrysum italicum* is a Mediterranean medicinal plant whose phloroglucinol (PG) α-pyrones constitute a structurally distinctive and pharmacologically relevant metabolite class, yet no systematic high-resolution mass spectrometric (HRMS) study has comprehensively characterized them. In this study, the acetone extract of inflorescences from *Helichrysum italicum* (Roth) G. Don was profiled by UHPLC–Orbitrap–MS in negative ion mode using full-scan and data-dependent MS/MS acquisition; exact-mass elemental composition assignment and HCD fragmentation analysis were combined with unsupervised clustering of fragment-ion and neutral-loss intensity matrices, validated against a manually curated annotation table using the adjusted Rand index (ARI) and cluster purity. From a total of 59 annotated compounds, 23 corresponded to known PG-α-pyrones previously reported from *Helichrysum* and 36 represented structures not previously described from the genus. Diagnostic fragment ions (FIs) and neutral losses (NLs) were established for identifying specific substituents of the α-pyrone and PG moieties. Semi-quantification gave a total PG-α-pyrone content of 0.019% dry weight, dominated by arzanol. This study establishes a UHPLC–HRMS framework for profiling phloroglucinol α-pyrone derivatives and confirms *H. italicum* as a rich source of this class.

## 1. Introduction

*Helichrysum italicum* (Roth) G. Don (Asteraceae) is a Mediterranean medicinal plant traditionally used for the treatment of inflammatory disorders, skin conditions, infections, and respiratory ailments [1]. Previous phytochemical investigations on the genus *Helichrysum* found the presence of flavonoids, benzoic and hydroxycinnamic acid derivatives, coumarins, terpenoids, benzofurans and pyrone derivatives and analogs [2,3,4]. Among these, phloroglucinol–α-pyrone conjugates constitute one of the most structurally distinctive and pharmacologically relevant metabolite classes reported from this genus [5].

Phloroglucinol α-pyrones (PG-α-pyr) are derived from the coupling of a acylphloroglucinol (2,4,6-trihydroxyacetophenone) unit with substituted α-pyrone rings via methylene bridges. Since the first reports in the 1970s [6,7,8], more than fifty structurally characterized PG-α-pyrones have been isolated from eleven *Helichrysum* species distributed across the Mediterranean, Africa, and Iran [5].

Based on structural features of the α-pyrone and phloroglucinol moieties, these compounds can be grouped into several subclasses as recently reviewed by us [9], including monopyrones [10,11,12,13], dipyrones [5,6,10,14,15,16,17], 3-non-alkylated phloroglucinol α-pyrones (3-NA PG-α-pyr) [18], 3-prenyl phloroglucinol α-pyrones (3-prenyl PG-α-pyr) including arzanol and related derivatives [10,11,12,14], 3-geranyl PG-α-pyr [5,14,19], 2-prenyl PG-α-pyr such as arenol A and homoarenol [6,17], and O-methylated derivatives of prenylated PG-α-pyr [7,8,18]. More structurally complex derivatives have also been reported, including benzofuran-, benzopyran-, chromane-, hetero-trimeric, and spiroketal derivatives [5,8,17,18,19].

Of this class of secondary metabolites, arzanol has been the most extensively studied representative. It was isolated from the aerial parts of *H. italicum* and yields of up to 0.48% (*w*/*w*) in *H. stoechas* [14] and up to 0.296% (*w*/*w*) in *H. italicum* ssp. *microphyllum* have been reported [11]. Arzanol exhibits potent anti-inflammatory activity through inhibition of NF-κB signaling (IC_50_ ≈ 12 µM) [10], dual inhibition of mPGES-1 (IC_50_ = 0.4 µM) and 5-LOX (IC_50_ = 3.1 µM) [20,21], and suppression of pro-inflammatory cytokines including IL-1β, TNF-α, IL-6, and IL-8 [10]. *In vivo* studies confirmed significant anti-inflammatory efficacy in rat pleurisy models [21]. Additionally, arzanol demonstrates antioxidant properties [13,22], antibacterial activity against Gram-positive pathogens including drug-resistant *Staphylococcus aureus* strains [11], neuroprotective effects against oxidative stress in keratinocytes and neuronal cells [23,24], and modulation of metabolic and mood-related pathways via SIRT1 inhibition [25].

Besides arzanol, several structurally related helipyrones A–C were demonstrated to have anti-inflammatory and moderate antibacterial effects [10,11,17]; 3-prenyl norauricepyrone and italidipyrone were identified as potential HIV-reverse transcriptase inhibitors [26].

Preparative isolation followed by NMR elucidation [6,7,8,10,11,12,14,16,17,18,19] remains the reference method for complete structural elucidation, but it is material-intensive, and trace constituents are commonly lost during the multi-step chromatography required to reach a pure compound. Progress has been correspondingly slow: fewer than sixty structures have been described in five decades, distributed across eleven *Helichrysum* species [9].

Chromatographic profiling with UV or DAD detection [3,4] quantifies individual targets such as arzanol but conveys no structural information; because all members of the class share the same acylphloroglucinol/α-pyrone chromophore, homologs cannot be distinguished from one another by their spectra.

LC–MS profiling has been applied to the genus using hydroalcoholic, aqueous and solvent-fractionated extracts [2,3,4,27,28], and MS fragmentation pathways have been proposed for individual pyrone and phloroglucinol constituents [5,29]. These studies established that the class is tractable by LC–MS, but annotation has rested chiefly on exact mass, ultraviolet spectra and comparison with the isolation literature. No transferable rule set has been published that assigns, from a single MS^2^ spectrum, the α-pyrone C6 substituent, the C1 acyl chain and the C3 prenyl or geranyl side chain simultaneously, and none have addressed how the cyclized scaffolds—benzofuran, benzopyran and spiroketal—may be distinguished from their open-chain counterparts by MS^2^ alone.

Despite the considerable structural diversity and pharmacological interest, to date, no systematic high-resolution mass spectrometry (HRMS) study has comprehensively characterized the qualitative and quantitative distribution of phloroglucinol α-pyrone derivatives. 

In this study, *H. italicum* was selected as the target species because it represents the most extensively reported source of PG-α-pyrones [12,30]. Given the structural similarities among subclasses—particularly variations in C1 acyl chains, C3 prenyl/geranyl substitution, and α-pyrone alkylation patterns—advanced UHPLC–HRMS analysis provides a means for initial screening and comparative metabolite profiling.

Therefore, the purpose of the present study is to characterize phloroglucinol α-pyrone derivatives in *H. italicum* extracts using UHPLC–Orbitrap–MS. By integrating exact-mass elemental composition assignment, MS/MS fragmentation pattern analysis, and multivariate statistical evaluation of fragment-ion relationships, we sought to establish an analytical framework for the comprehensive profiling and semi-quantitative assessment of this structurally diverse and biologically significant metabolite class.

## 2. Results and Discussion

The extract analyzed in this study was a total acetone extract, prepared without fractionation. Phloroglucinol α-pyrones are non-glycosylated, lipophilic polyphenols, and in the isolation literature, they are recovered either directly from acetone extracts [10,11,12] or—where extraction begins with a hydroalcoholic solvent and only after partition into a lipophilic phase—dichloromethane [5,29], n-hexane [3] or ethyl acetate [31]. Aqueous and hydroalcoholic extracts of *H. italicum* are themselves dominated by acylquinic acids and flavonoid glycosides [3,28], and a comparative survey of separation techniques applied to the species reaches the same conclusion [32]. A single extraction step therefore yields an extract enriched in the target class and the crude extract remains suitable for the determination of other metabolite classes.

The following sections describe the mass spectrometric features used to annotate phloroglucinol α-pyrone derivatives in the *H. italicum* extract. First, the diagnostic fragment ions (FIs) and neutral losses (NLs) that define each structural element are presented. These features are then applied systematically across the individual structural subclasses. Building on these annotations, the FI and NL data are examined by unsupervised clustering to assess how far MS/MS features alone encode structural information (Section 2.7, Section 2.8 and Section 2.9), and the semi-quantitative distribution of the annotated compounds is compared with literature values (Section 2.10). The confidence attached to the annotations and the constraints on the quantitative data are addressed in Section 2.11. Together, these analyses establish a UHPLC–HRMS framework for profiling this structurally diverse metabolite class.

### 2.1. MS Features for Determining the α-Pyrone Unit

In negative ion mode HCD MS/MS, the α-pyrone moieties undergo decarboxylation, yielding a neutral loss of CO_2_ (43.9898 Da). The presence of homologous alkyl substituents at C6 results in systematic mass shifts of +14.0151 Da in both the intact α-pyrone fragment and its decarboxylated product ion. Fragmentation across the methylene bridge determines whether the α-pyrone is retained as a charged fragment or eliminated as a neutral species.

As observed for all detected PG-α-pyrones in *H. italicum*, structural variation within the α-pyrone moiety arises from the alkyl substituent at C6; diagnostic NLs and fragment ions (FIs)—a homologous series of ions—enable the assignment of this substituent (Table 1 and Table 2). The α-pyrone unit generates two diagnostic fragment ions: the 6-alkyl-5-methyl-4-hydroxy-2*H*-pyran-2-one anion, denoted as [α-pyr]^−^, and its decarboxylated (−43.9898 Da) product, the [α-pyr-CO_2_]^−^ ion (Table 2). Depending on the alkyl substituent at C6, these fragment ions exhibit incremental mass increases of 14.0151 Da, corresponding to successive methylene (–CH_2_–) units. As exemplified for arzanol (cmpd **20**) (Section 2.5), the C6-ethyl substituent is indicated by the presence of [α-pyr-H]^−^ at *m*/*z* 153.0558 and its decarboxylated product [α-pyr-CO_2_-H]^−^ at *m*/*z* 109.0659; the complementary diagnostic neutral losses are 154.0630 Da and 166.0630 Da.

Dipyrones and benzopyrans display these α-pyrone-derived fragment ions as major peaks (60–100%), whereas the other classes display them at lower relative intensity (15–30%).

### 2.2. MS Features for Determining the Phloroglucinol Structure

Structural diversity within phloroglucinol-derived metabolites arises primarily from variations in the C1 acyl substituent and the nature of the C3 side chain.

**Figure 1:**
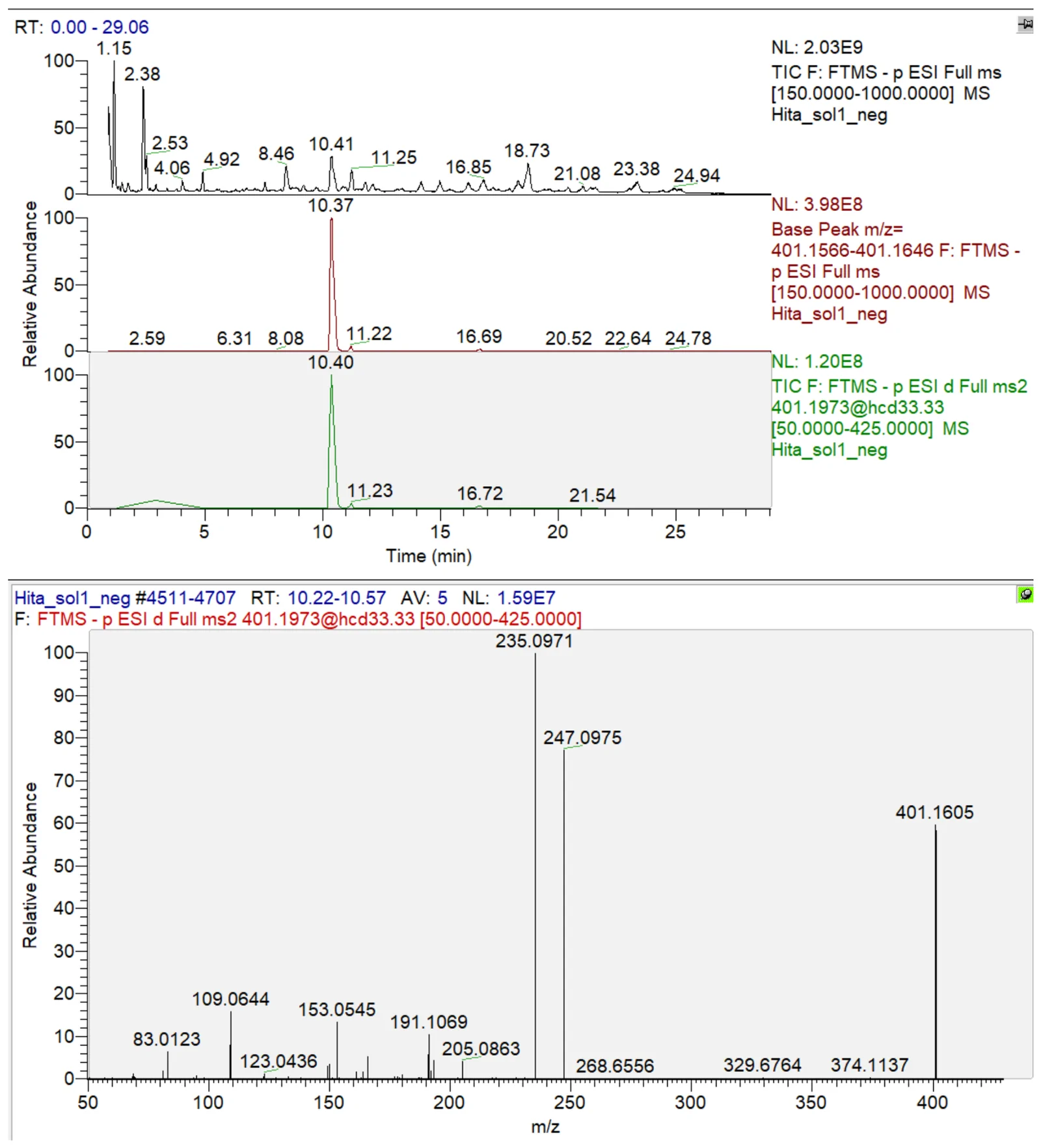
TIC and EIC of molecular ion at *m*/*z* 401.1606 (identified as arzanol) in negative ESI–Orbitrap MS/MS. TIC, total ion chromatogram; EIC, extracted-ion chromatogram; ESI, electrospray ionization.

All described classes undergo initial fragmentation via cleavage on either side of the methylene bridge, yielding *Fragment A (PG-CH_2_)* and *Fragment B (PG)*, corresponding to acylphenone-derived ions with and without retention of the methylene unit, respectively. To our knowledge, this is the first report to propose a methylenecyclohexadienone structure for *Fragment A*. This is exemplified in the proposed fragmentation for arzanol (Figure 1; see Section 2.5: 3-prenyl PGs). Proposed fragmentation pathways for all detected compounds are compiled in Supplementary File S2; representative schemes are shown in Section 2.5.

The C3 position may remain unsubstituted, as observed in 3-non-alkylated PG-α-pyr (3-NA PG-α-pyr), or may carry prenyl (3-prenyl PG-α-pyr) or geranyl (3-geranyl PG-α-pyr) side chains. These isoprenoid substituents may be hydroxylated, usually at C8, as in heliarzanol [11]. Intramolecular cyclization involving prenyl/geranyl substituents leads to the formation of benzopyran-, benzofuran-, and chromane-type PG-α-pyrone derivatives.

A structurally distinct subclass is represented by the spiroketal derivatives, where the phloroglucinol-derived benzofuran core forms a spiro-fused system with a five-membered lactone (furan-3-one), generating a 3*H*,3′*H*-spiro[benzofuran-2,2′-furan]-3′-one scaffold.

### 2.3. MS Features for Determining the C1 Alkyl Chain

The structural variability of the acetophenone moiety stems from the C1 acyl substituent. A set of diagnostic fragment ions corresponding to the deprotonated acyl moieties—a homologous series differing by CH_2_ units (14.0151 Da)—allows straightforward identification of the C1 acyl group (Table 3).

For example, as in the fragmentation of arzanol (Section 2.5), the acetyl substituent is unambiguously characterized by a fragment ion at *m*/*z* 83.01385 (C_4_H_3_O_2_^−^), while propanoyl, butanoyl, and pentanoyl substituents yield ions at *m*/*z* 97.02950 (C_5_H_5_O_2_^−^), 111.04515 (C_6_H_7_O_2_^−^), and 125.06080 (C_7_H_9_O_2_^−^), respectively.

### 2.4. MS Features for Determining the C3 Prenyl/Geranyl Chain

All compounds possessing an acylphenone moiety are C3-alkylated, except the 3-NA PG-α-pyr [9,18]. The C3 side chain is mostly prenyl or geranyl, but it may also be hydroxylated [9].

#### 2.4.1. In 3-Prenyl PGs

Fragment ions at *m*/*z* 205.08702 and *m*/*z* 193.08702 are exclusively observed in C3-prenylated phloroglucinol-α-pyrones and together constitute a diagnostic pair for this scaffold. The 205.08702 *m*/*z* ion arises from Fragment A (PG-CH_2_), whereas the 193.08702 *m*/*z* ion arises from Fragment B (PG), in both cases following cleavage of the C1 acyl substituent, at approx. 3–5% relative intensity. The concurrent presence of both ions confirms the C3-prenylated phloroglucinol-α-pyrone scaffold.

#### 2.4.2. In 3-(Hydroxyprenyl) PGs

While *m*/*z* 205.08702 and *m*/*z* 193.08702 are diagnostic for the 3-prenyl PG core, the corresponding fragment ions for the 3-(hydroxyprenyl) derivatives (cmpds **24** and **29**) are 221.08193 (C_12_H_13_O_4_^−^) formed from fragment A after C1 cleavage, and the fragment ion 209.08193 *m*/*z* (C_11_H_13_O_4_^−^) and its dehydrated product ion 191.07137 *m*/*z* (C_11_H_11_O_3_^−^). The placement of the hydroxyl on the C3 prenyl group was inferred from the structure of heliarzanol [9,11]. Another set of compounds was detected whose fragment ions corresponded to those diagnostic for C3 3-(hydroxyprenyl) derivatives but were heavier by two hydrogens (2.014 Da). Based on comparison with the fragmentation of 3-(hydroxyprenyl) derivatives, we inferred, from the 2.014 Da (H_2_) difference, that the hydrogenation is located on the C3 side chain; thus, instead of a prenyl group, these compounds carry a 3-(hydroxyprenyl) moiety. Such fragment ions include 223.09758 *m*/*z* (C_12_H_15_O_4_^−^) and 211.09758 (C_11_H_15_O_4_^−^) *m*/*z* formed after C1 cleavage from Fragment A or Fragment B, respectively.

#### 2.4.3. In 3-Geranyl PGs

The fragment ion at *m*/*z* 203.07137 is characteristic of the C3 geranyl group and is derived from cleavage of both the C1 acyl group and a prenyl unit from the geranyl side chain.

#### 2.4.4. In 3-(Hydroxygeranyl) PGs

In 3-(hydroxygeranyl) PGs, the masses of the characteristic fragment ions are higher by an additional oxygen atom (+15.999 Da). Based on comparison with the fragmentation of related compounds, the hydroxyl group was assigned to the C3 side chain (cmpds **48**, **53**, and **54**).

### 2.5. MS/MS Characterization by Classes

A representative total ion chromatogram (TIC) of the *H. italicum* acetone extract, together with the extracted-ion chromatogram (EIC) of arzanol [M-H]^−^ 401.1606 *m*/*z*, is shown in Figure 1. The exact-mass elemental compositions, measured [M-H]^−^ values, retention times and MS^2^ fragment ions of all 59 annotated compounds are compiled in Table A1; the corresponding MS/MS spectra are provided for each compound individually in Supplementary File S1. The diagnostic ions described in the following subsections are compared across all structural subclasses in Section 2.6.

#### 2.5.1. Mono- and Dipyrones

**Figure 2:**
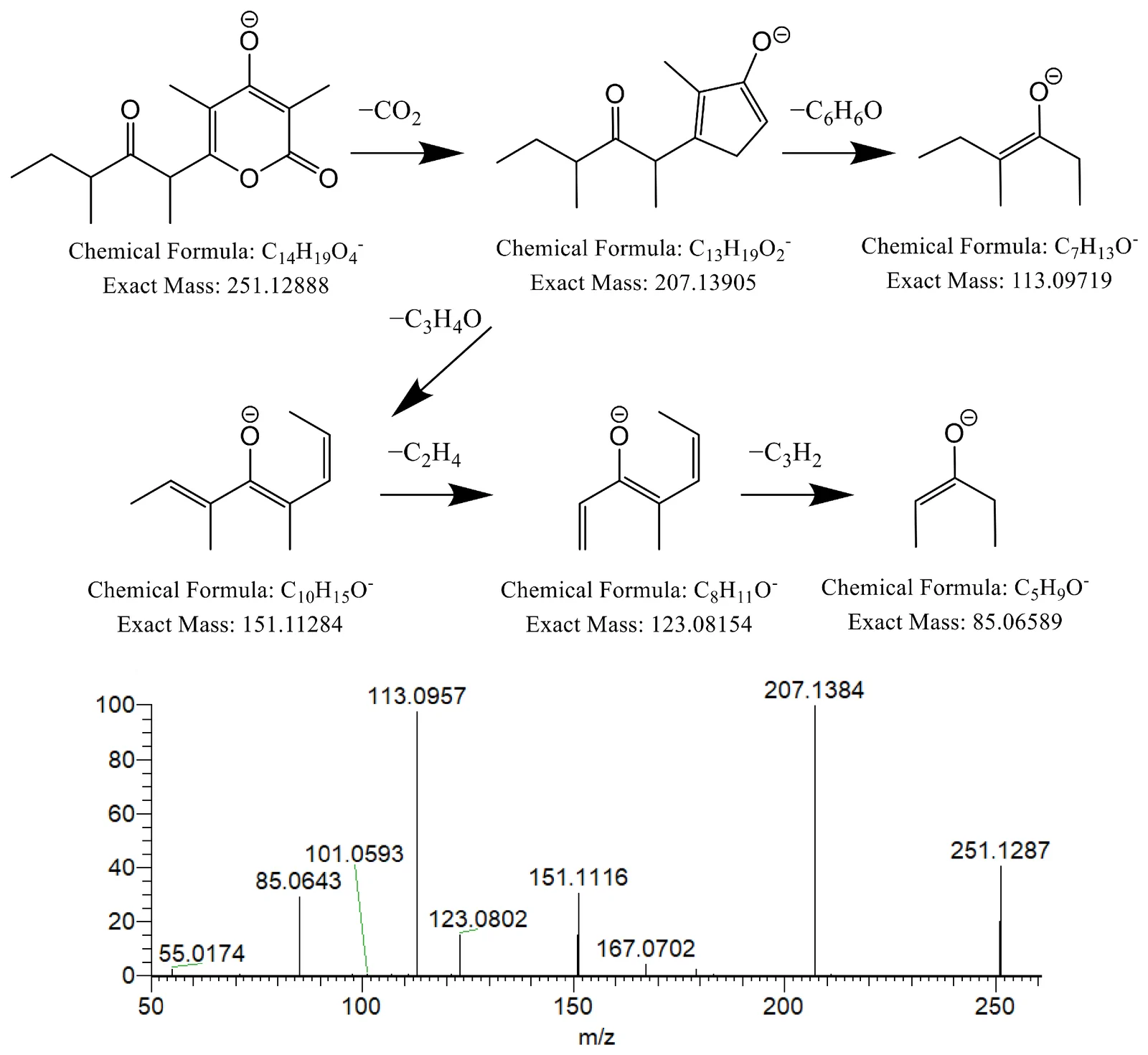
Proposed fragmentation behavior of monopyrone, with [M-H]^−^ at *m*/*z* 251.12888 identified as micropyrone.

The only monopyrone detected was micropyrone (cmpd **1**) (Figure 2). Micropyrone (cmpd **1**) (C_14_H_20_O_4_, exact mass 252.1362 Da) was isolated in several studies from aerial parts of *H. italicum* [10,11,12,33].

**Figure 3:**
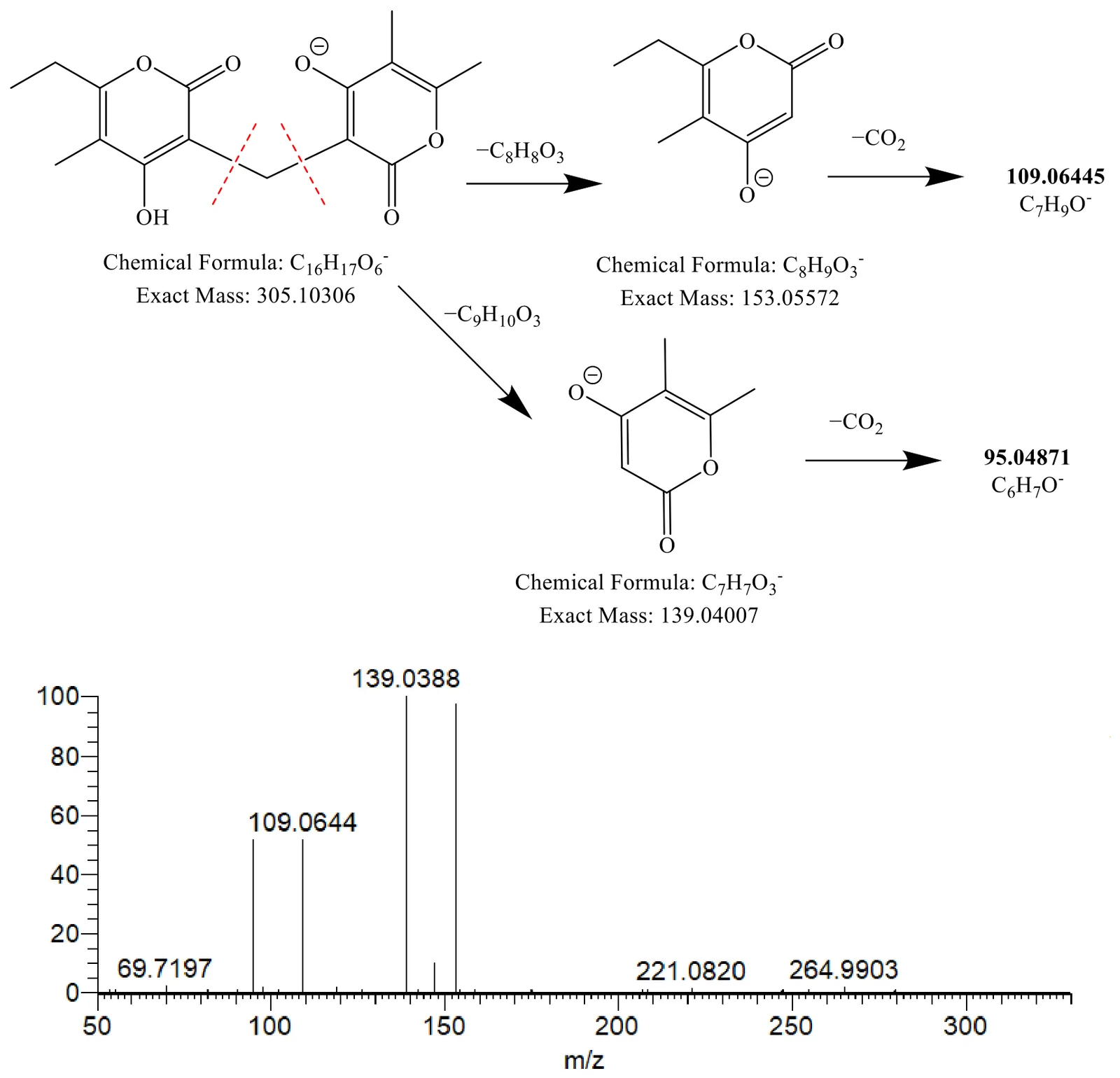
Proposed fragmentation pattern of helipyrone B (cmpd **2**), with [M-H]^−^ at *m*/*z* 305.10306.

Dipyrones consist of two alpha-pyrones linked through a methylene bridge. Two dipyrones were detected—helipyrone A (cmpd **4**) and B (cmpd **2**) with [M-H]^−^ at *m*/*z* 319.1187 and 305.1031, respectively (Figure 3). Helipyrone B (cmpd **2**) has been isolated from the flowers and aerial parts of *H. stoechas* [16,17] and the roots of *H. arenarium* [6], as well as from the aerial parts of *H. oocephalum* [5]. Helipyrone A (cmpd **4**) (C_17_H_20_O_6_, exact mass 320.126 Da) has been the most frequently isolated compound from the *Helichrysum* species. It was reported in *H. stoechas* flowers [14], *H. italicum* ssp. *microphyllum* aerial parts with flowers [10,11,15,33], and *H. oocephalum* aerial parts [5].

The dipyrones fragment on either side of the methylene bridge, forming characteristic pyrone fragment ions, which in turn undergo a characteristic loss of CO_2_. As observed in other α-pyrone dimers, cleavage on the α-pyrone side generates the α-pyrone fragment ion, whereas cleavage on the opposing side leads to neutral loss of the α-pyrone–CH_2_ unit.

#### 2.5.2. 3-Non-Alkylated Phloroglucinols (3-NA PGs)

3-Non-alkylated phloroglucinol–α-pyrones (3-NA PG-α-pyr) (cmpds **3**, **5**, **6**, **7**, **9**, **10**, **15**, **16**, **17**) consist of an acylphloroglucinol (2,4,6-trihydroxyacylphenone) moiety linked to a substituted α-pyrone ring via a methylene bridge. A defining structural feature of this subclass is the absence of a substituent at the C3 position of the phloroglucinol core. The only representatives of 3-NA PGs isolated from *Helichrysum* species are norauricepyrone (C_20_H_24_O_7_, exact mass 376.1522 Da) and methyl-norauricepyrone (C_21_H_26_O_7_, exact mass 390.1673 Da) [9]. They were co-isolated from the roots of *H. cephaloideum* DC. collected in Transvaal, South Africa [18].

**Figure 4:**
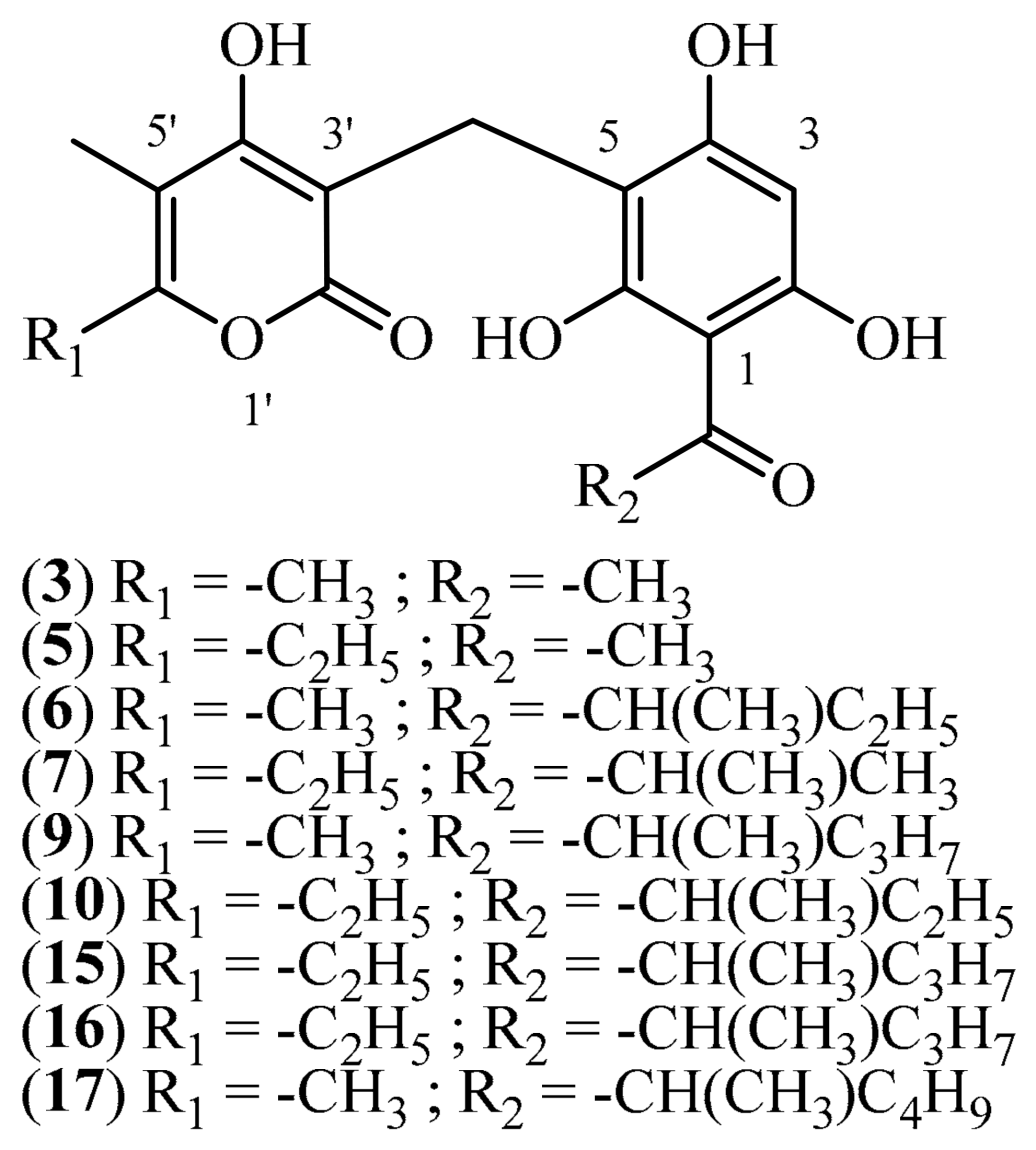
Detected 3-NA PG-α-pyr in *H. italicum* in this study.

Structural variability of the 3-NA PGs arises primarily from differences in the alkyl chain of the C1 acyl group and the alkyl substituent at the C6 position of the α-pyrone ring (Figure 4).

**Figure 5:**
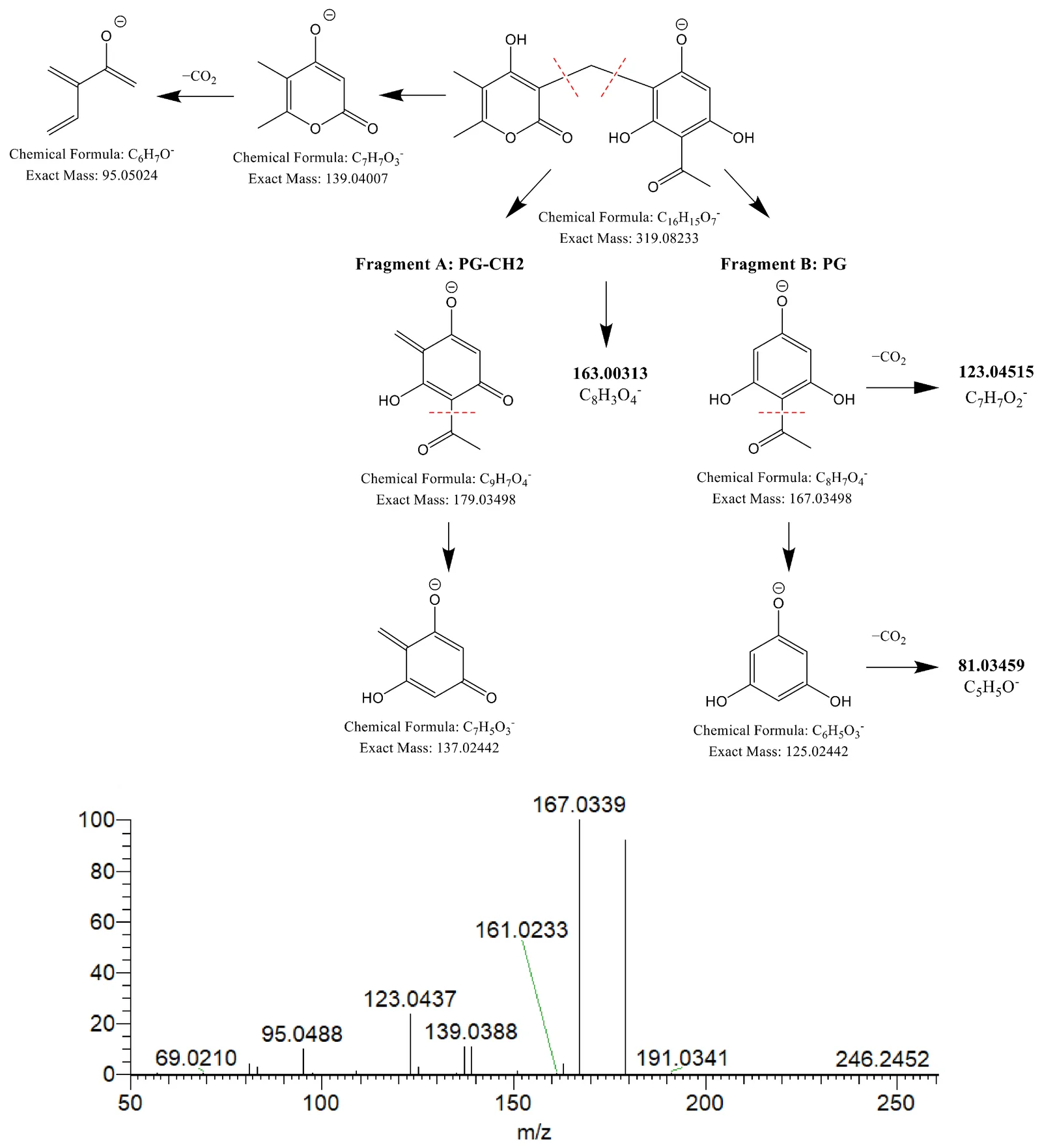
Proposed fragmentation pathway of cmpd **3**, with [M-H]^−^ at *m*/*z* 319.08233 belonging to the 3-NA PGs in negative ion mode.

The molecular ion (relative intensity ~20–60%) undergoes initial cleavage on either side of the methylene bridge, generating two principal fragment ion series (Figure 5). Cleavage on the α-pyrone side produces a phloroglucinol-containing fragment retaining the C5 methylene (Fragment A, PG–CH_2_), which appears at 80–90% relative intensity, together with the corresponding α-pyrone fragment ion at 10–15% relative intensity. In contrast, cleavage on the phloroglucinol side yields the phloroglucinol fragment ion (Fragment B, PG) as the base peak, with no corresponding α-pyrone fragment ion detected, indicating loss of the α-pyrone–CH_2_ moiety as a neutral species. No meaningful fragment ions are observed in the *m*/*z* region between the molecular ion and Fragment A.

The α-pyrone moiety generates the characteristic fragment ion pair described earlier ([α-pyr-H]^−^ and [α-pyr-CO_2_-H]^−^), depending on the substitution pattern at C6. Within the 3-NA PG subclass, additional diagnostic fragmentation arises from the phloroglucinol-containing fragments. From Fragment A (PG–CH_2_), loss of the C1 acyl substituent yields a characteristic ion at *m*/*z* 137.0244, observed in all 3-NA PGs at 10–20% relative intensity.

Further fragmentation of Fragment B (PG) involves sequential losses of the C1 acyl substituent and CO_2_. Loss of the C1 acyl group produces the phloroglucinol anion at *m*/*z* 125.0244, typically observed at approximately 5% relative intensity. Combined loss of both CO_2_ and the C1 acyl group results in the formation of a fragment ion at *m*/*z* 81.0346 (~5% relative intensity). In addition, a fragment ion at *m*/*z* 163.0031 (C_8_H_3_O_4_^−^) is consistently detected in all 3-NA PGs at low relative intensity (2–6%), although its origin could not be conclusively assigned.

The exclusive presence of *m*/*z* 137.0244 and 125.0244 (→ *m*/*z* 81.03) and 163.0031 provides structural confirmation for the 3-non-alkylated phloroglucinol-α-pyrone (3-NA PGs) scaffold.

The elimination of CO_2_ from phloroglucinol-derived fragments was corroborated by the fragmentation analysis of standard 2′,4′,6′-trihydroxyacetophenone (THAP) under identical HCD conditions.

THAP gave [M–H]^−^ at *m*/*z* 167.0339 together with a product ion at *m*/*z* 123.0438, corresponding to the neutral loss of CO_2_. This confirms that the acylphloroglucinol moiety itself undergoes decarboxylation, consistent with the ring-opening/re-cyclisation decarboxylation reported for *meta*-polyphenolic anions [34,35], and independently supports the assignment of the *m*/*z* 125.0244 → 81.0346 transition described above.

#### 2.5.3. 3-Prenyl PGs

**Figure 6:**
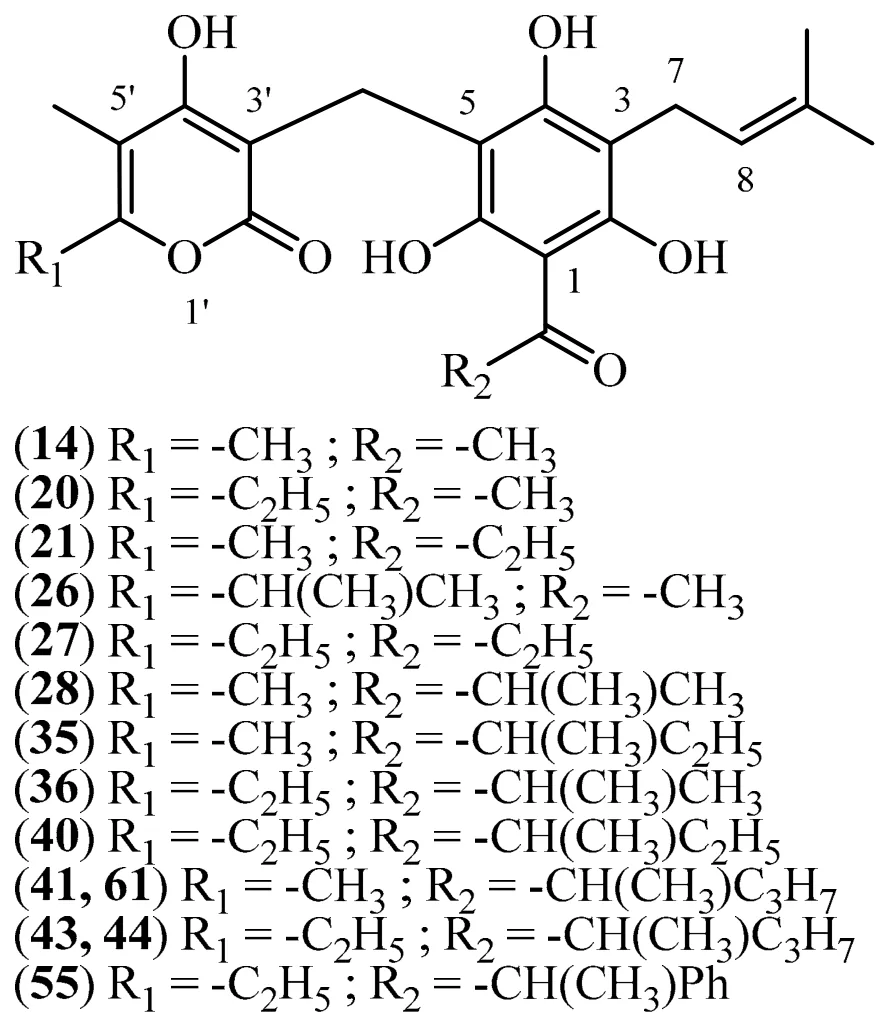
Identified 3-prenyl PG–α-pyr in *H. italicum* in this study.

Phloroglucinol–α-pyrone derivatives bearing a C3 prenyl substituent and a C1 acyl group (3-prenyl PG–α-pyr) represent the most abundant subclass within the phloroglucinol–α-pyrone family, in both quantity and variety (Figure 6).

In *H. italicum* ssp. *microphyllum*, arzanol (cmpd **20**) was isolated from dried aerial parts with flowers [10,11,33]; in *H. stoechas*, it was obtained from the flowers [14]. The compound was also isolated from the dried aerial parts of *H. italicum* (Roth) G. Don [12]. Helitalone B (cmpd **26**) [12] and heliarzanol [11] were isolated from the flowers of *H. italicum*.

Among the detected 3-prenyl PGs in our study, several have already been described in the literature. In aerial parts of *H. oocephalum*, arenol B (cmpd **28**) and arenol C (cmpd **35**) were isolated [5]. 6-O-desmethylauricepyrone (cmpd **36**) and 23-methyl-6-O-desmethylauricepyrone (cmpd **40**) are present in *H. odoratissimum* [8]. Furthermore, 23-methyl-6-O-desmethylauricepyrone (cmpd **40**) and its analog 23-ethyl-6-O-desmethyl-auricepyrone (cmpd **43**/**44**) were isolated from the roots of *H. mixtum* and the aerial parts of *H. stenopterum* [18].

**Figure 7:**
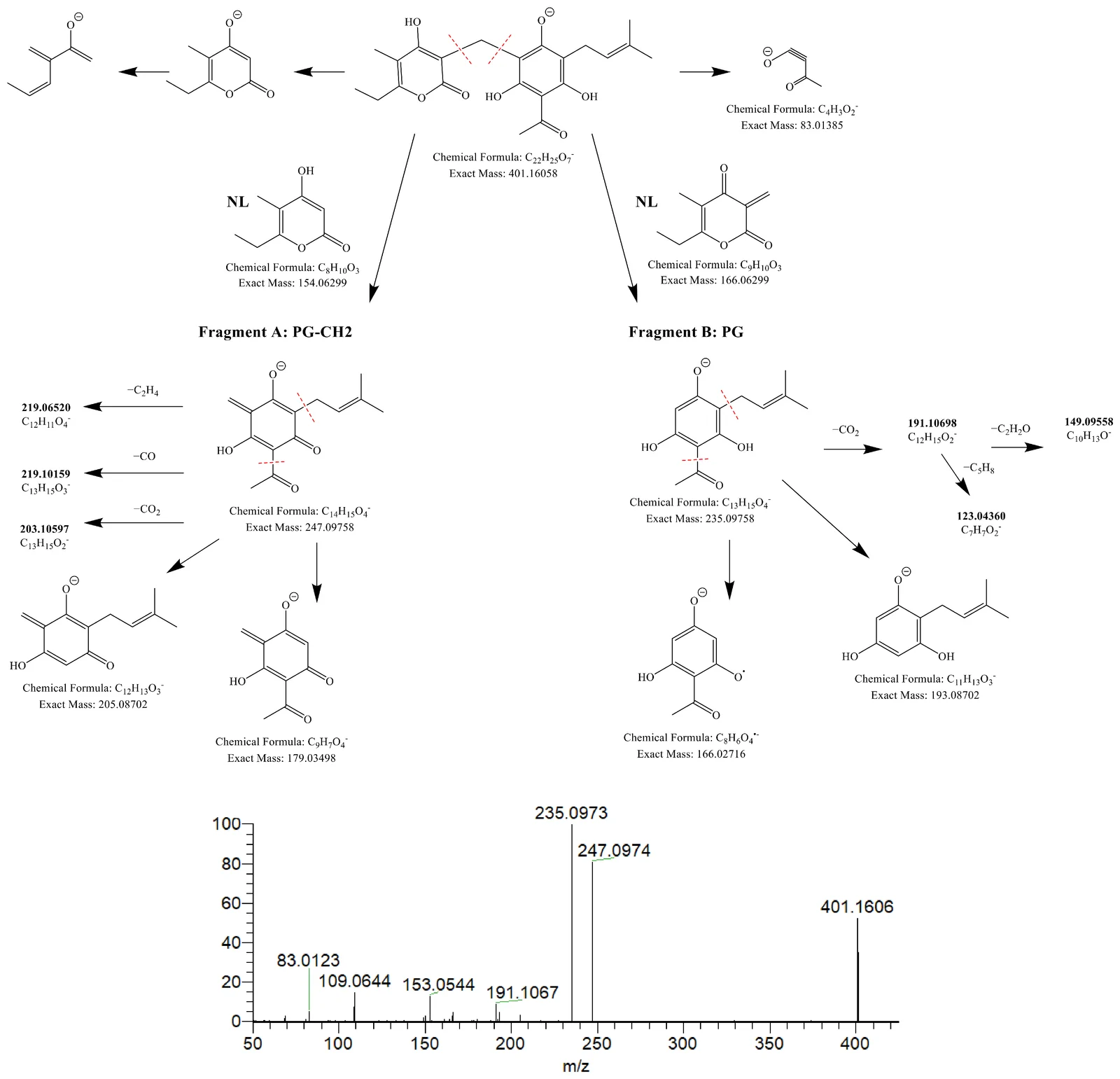
Proposed fragmentation pattern of arzanol (cmpd **20**), with [M-H]^−^ at *m*/*z* 401.16058 in negative ion mode.

The fragmentation behavior of 3-prenyl PGs (Figure 7) is governed by initial cleavage at either side of the methylene bridge, generating Fragment A (PG–CH_2_) and Fragment B (PG), analogously to the behavior observed for 3-NA PGs. The fragmentation pattern of the α-pyrone moiety follows the characteristic pathways described previously.

Fragment A (PG–CH_2_) undergoes neutral losses of C_2_H_4_, CO, and CO_2_. In addition, cleavage of the C1 acyl substituent leads to the formation of a fragment ion at *m*/*z* 205.0870 (3–5% intensity), characteristic for 3-prenyl PGs. Cleavage of the C3 prenyl side chain is also observed; however, a fragment ion corresponding to the simultaneous loss of both the C1 acyl and C3 prenyl substituents was absent.

Fragment B (PG) undergoes loss of CO_2_, followed by subsequent cleavage of either the C1 acyl substituent or the C3 prenyl side chain. Cleavage of the C3 prenyl group gives a characteristic fragment ion at *m*/*z* 193.0870 as a minor peak (4–5%).

Substitution at the C3 side chain is directly reflected in the mass and composition of the diagnostic fragment ions derived from both Fragment A and Fragment B.

In 3-prenyl PGs (cmpds **14**, **20**, **21**, **26**, **27**, **28**, **35**, **36**, **40**, **41**, **43**, **44**, **55**, and **61**), the ions at *m*/*z* 205.0870 (C_12_H_13_O_3_^−^) and 193.0870 (C_11_H_13_O_3_^−^) constitute a diagnostic pair for the C3-prenylated phloroglucinol scaffold. The ion at *m*/*z* 205.0870 originates from Fragment A following cleavage of the C1 acyl substituent, whereas the ion at *m*/*z* 193.0870 is generated from Fragment B upon loss of the C3 prenyl side chain. The concurrent presence of these two fragment ions indicates a C3-prenylated phloroglucinol structure.

In 3-(hydroxyprenyl) PGs (cmpds **24** and **29**), substitution within the C3 prenyl side chain is reflected by systematic mass shifts in the diagnostic fragment ions, corresponding to the incorporation of an additional hydroxyl group (+15.999 Da relative to the non-hydroxylated analogs, corresponding to an additional oxygen atom). Accordingly, the characteristic fragment ions are observed at *m*/*z* 221.0819 (C_12_H_13_O_4_^−^), formed from Fragment A following cleavage of the C1 acyl substituent. Additional fragment ions at *m*/*z* 209.0819 (C_11_H_13_O_4_^−^) and its dehydrated product ion at *m*/*z* 191.0714 (C_11_H_11_O_3_^−^) are also detected. The occurrence of the dehydration product supports the presence of a hydroxyl group within the C3 side chain. The position of the hydroxyl group was inferred based on the reported structure of heliarzanol [9,11].

A further subgroup, assigned as 3-(hydroxydihydroprenyl) PGs (cmpds **25** and **32**), exhibits fragment ions analogous to those of the 3-(hydroxyprenyl) derivatives but shifted by +2.014 Da, corresponding to an addition of H_2_. Accordingly, fragment ions at *m*/*z* 223.0976 (C_12_H_15_O_4_^−^) and 211.0976 (C_11_H_15_O_4_^−^) are observed, formed after cleavage of the C1 acyl substituent from Fragment A and Fragment B, respectively. The consistent +2.014 Da shift relative to the corresponding 3-(hydroxyprenyl) diagnostic fragments supported the assignment of hydrogenation localized within the C3 side chain.

In 3-geranyl PGs (cmpds **42**, **47**, and **50**), the presence of the extended C_10_ isoprenoid side chain is clearly reflected in the masses of the fragment ions derived from both Fragment A and Fragment B. A characteristic fragment ion at *m*/*z* 261.1496 (C_16_H_21_O_3_^−^) is observed from Fragment A and serves as a diagnostic feature of C3-geranyl substitution. Additionally, a fragment ion at *m*/*z* 203.0714 is consistently detected and is attributed to secondary fragmentation involving cleavage of the C1 acyl substituent together with partial degradation of the geranyl side chain, corresponding to loss of a prenyl unit.

In 3-(hydroxygeranyl) PGs (cmpds **48**, **53**, and **54**), the diagnostic fragment ions exhibit systematic mass increases consistent with the incorporation of a hydroxyl group (+15.999 Da corresponding to an additional oxygen atom). Accordingly, the fragment ions characteristic of geranyl-containing compounds are shifted to higher *m*/*z* values. Based on comparison with the fragmentation behavior of related compounds, the hydroxyl group is assigned to the C3 side chain.

#### 2.5.4. Benzofurans (BFs)

**Figure 8:**
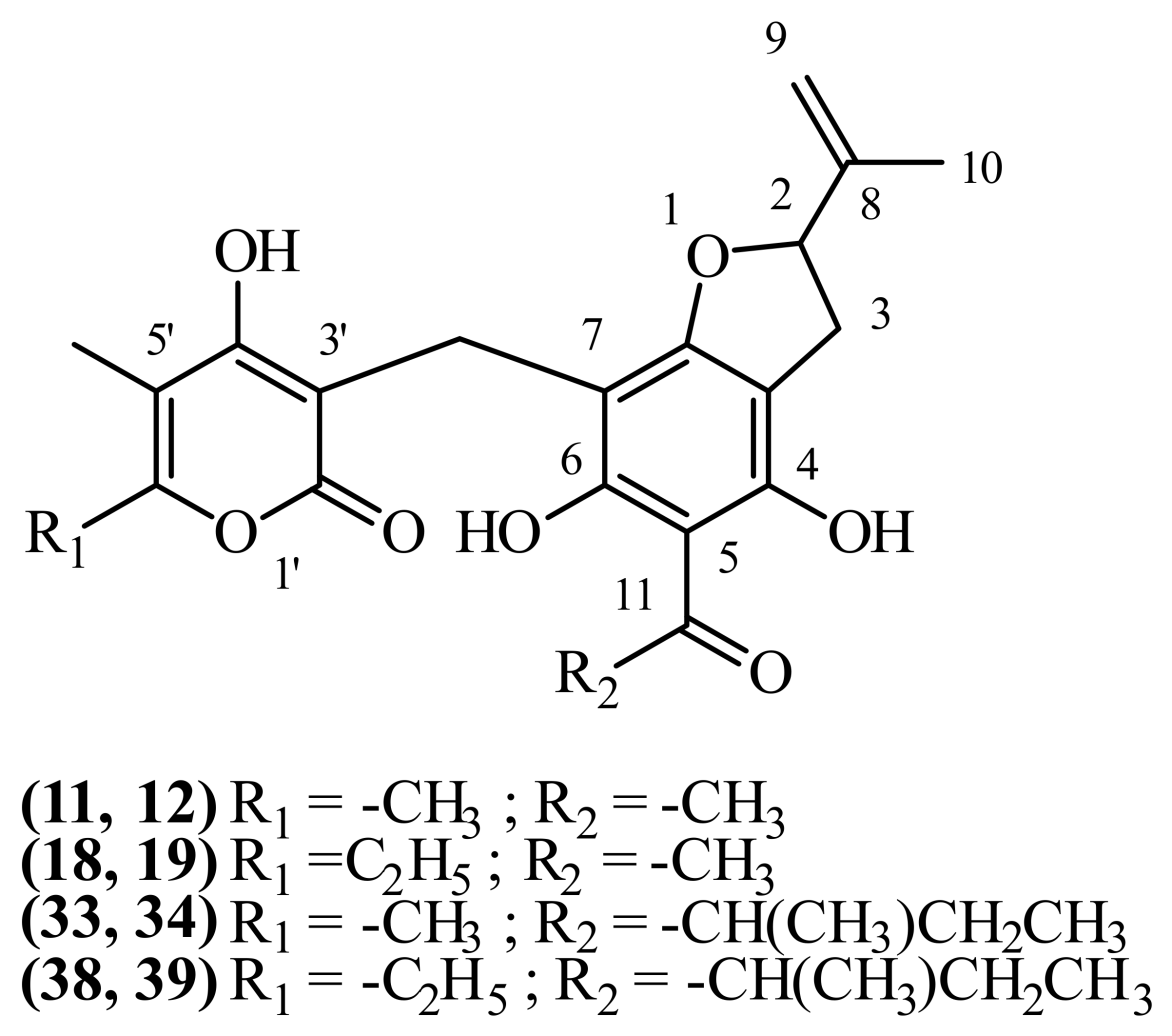
Identified BFs in *H. italicum*.

Benzofuran (BF) derivatives (cmpds **11**, **12**, **18**, **19**, **33**, **34**, **38**, and **39**) are characterized by a fused benzofuran ring system formed via intramolecular cyclization of the C3 prenyl or geranyl side chain with a phenolic hydroxyl group of the acylphloroglucinol (acetophenone) moiety (Figure 8).

Among identified BFs, several have been previously isolated from *Helichrysum* species. Italipyrone (cmpd **18**/**19**) (C_22_H_24_O_7_, exact mass 400.1522 Da) was isolated from the aerial parts of *H. italicum* [8] and *H. stoechas* [17]. 22-Methyl-22-ethyl-italipyrone (cmpds **38**/**39**) (C_25_H_30_O_7_, exact mass 442.1991 Da) was isolated from the roots of *H. cephaloideum* [8,18] and from the roots of *H. mixtum* [18].

**Figure 9:**
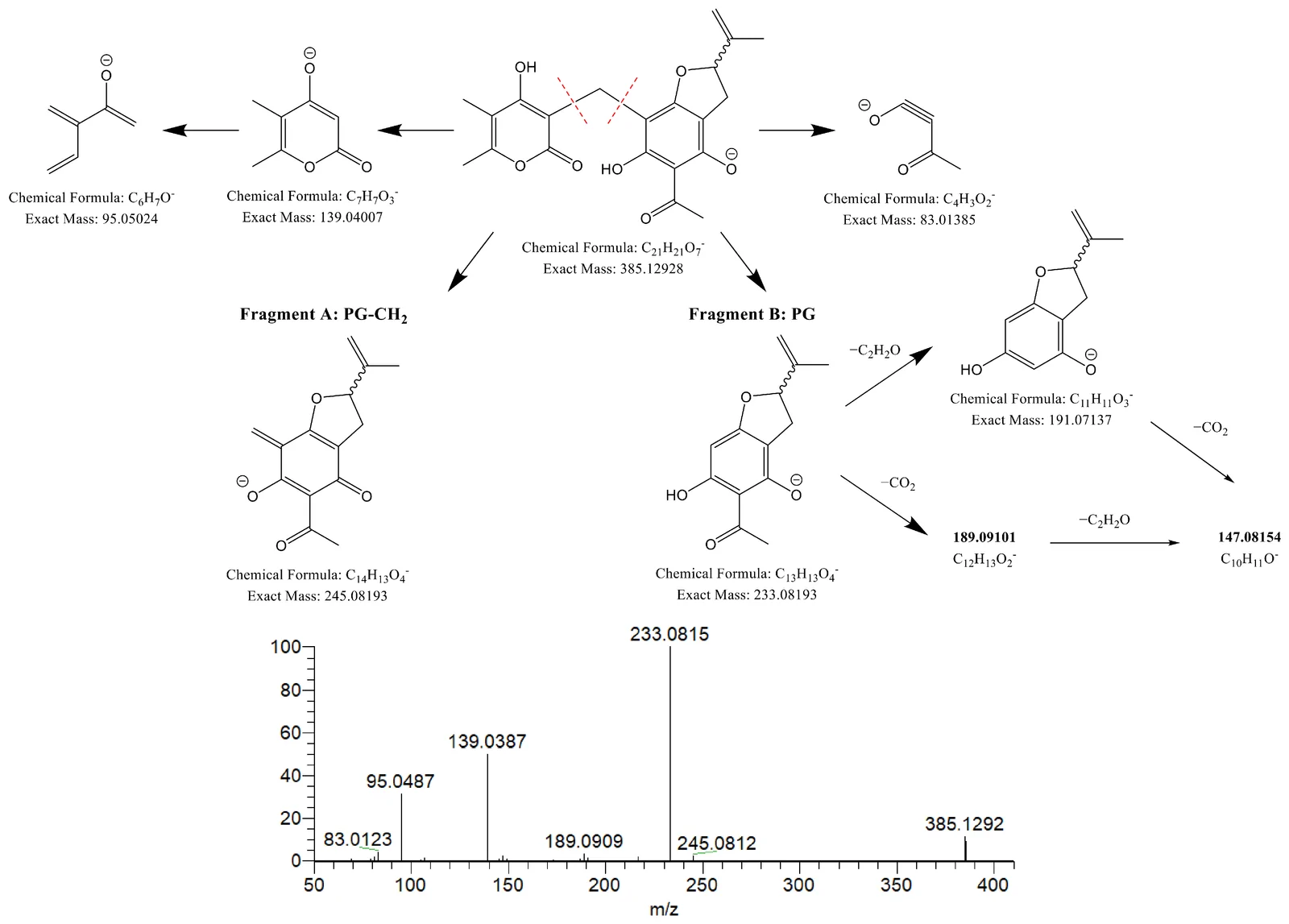
Fragmentation pathway of cmpd **11**, with [M-H]^−^ at *m*/*z* 385.12928 belonging to the (BFs) in negative ion mode.

As in other phloroglucinol–α-pyrone-derived scaffolds, structural variability among BFs arises primarily from differences in the alkyl chain of the C1 acyl substituent and the alkyl substituent at the C6 position of the α-pyrone ring (Figure 9).

The α-pyrone moiety produces a characteristic fragment ion pair consisting of the intact α-pyrone fragment [α-pyr-H]^−^ (~15–60% relative intensity) and its decarboxylated product [α-pyr-CO_2_-H]^−^ (~15–40% relative intensity). In BFs, these α-pyrone-derived fragment ions generally appear at lower relative intensity compared to those observed in benzopyran (BP) derivatives, providing a useful criterion for distinguishing between the two subclasses.

Cleavage on the α-pyrone side of the methylene bridge generates Fragment A, which is typically observed only at low abundance (~0–10% relative intensity). The dominant fragmentation pathway is the formation of Fragment B through cleavage on the phloroglucinol side, which constitutes the base peak. Fragment B undergoes a loss of CO_2_ and/or C1 cleavage, resulting in the fragment ion at *m*/*z* 147.0815.

#### 2.5.5. Benzopyrans (BPs)

**Figure 10:**
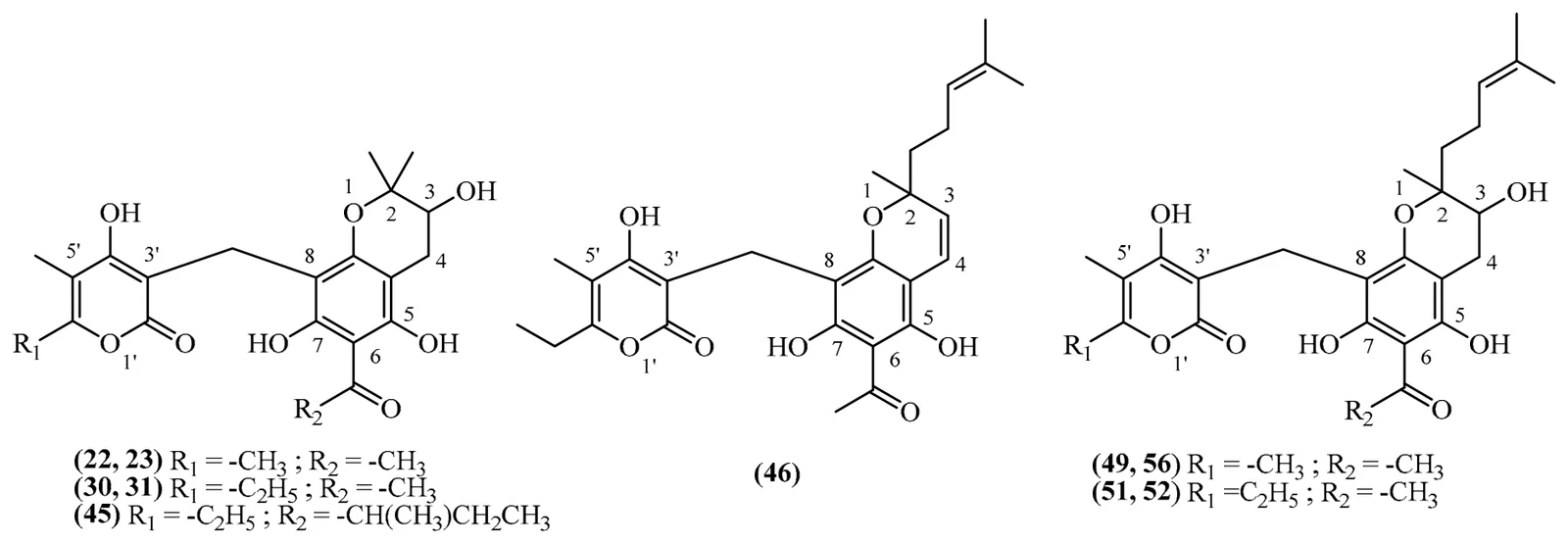
Identified BPs in *H. italicum*.

Benzopyran (BP) derivatives (cmpds **22**, **23**, **30**, **31**, **45**, **46**, **49**, **51**, **52**, and **56**) are characterized by a fused benzopyran ring system formed via intramolecular cyclization between the C3 prenyl or geranyl side chain and the para-hydroxyl group of the acylphloroglucinol moiety (Figure 10). Among the identified compounds, plicatipyrone (C_22_H_26_O_8_, exact mass 418.1628 Da) (cmpds **30**/**31**) was identified in the flower heads of *H. plicatum* [8] and in the aerial parts and flowers of *H. stoechas* [17].

As in other phloroglucinol–α-pyrone-derived scaffolds, structural variability among BPs arises primarily from differences in the alkyl chain of the C1 acyl substituent and the alkyl substituent at the C6 of the α-pyrone ring.

The molecular ion is typically observed at relative intensity 20–85%.

The α-pyrone moiety produces a characteristic fragment ion pair consisting of the intact α-pyrone fragment [α-pyr-H]^−^ (~85–100% relative intensity) and its decarboxylated product [α-pyr-CO_2_-H]^−^ (~60–85%), which provide information about the alkyl substituent at the C6. As mentioned in the previous section, the α-pyrone-derived fragment ions in BPs appear at much greater intensity than those of BFs, allowing the two subclasses to be differentiated.

Cleavage on the α-pyrone side of the methylene bridge generates Fragment A ([M − α-pyr-H]^−^), which in BPs appears only at low abundance (~0–5% relative intensity). In contrast, cleavage on the phloroglucinol side yields the phloroglucinol fragment (PG), which constitutes the dominant fragment ion and consistently appears as the base peak.

Cmpds **22**, **23**, **30**, **31** and **45** have similar fragmentation.

**Figure 11:**
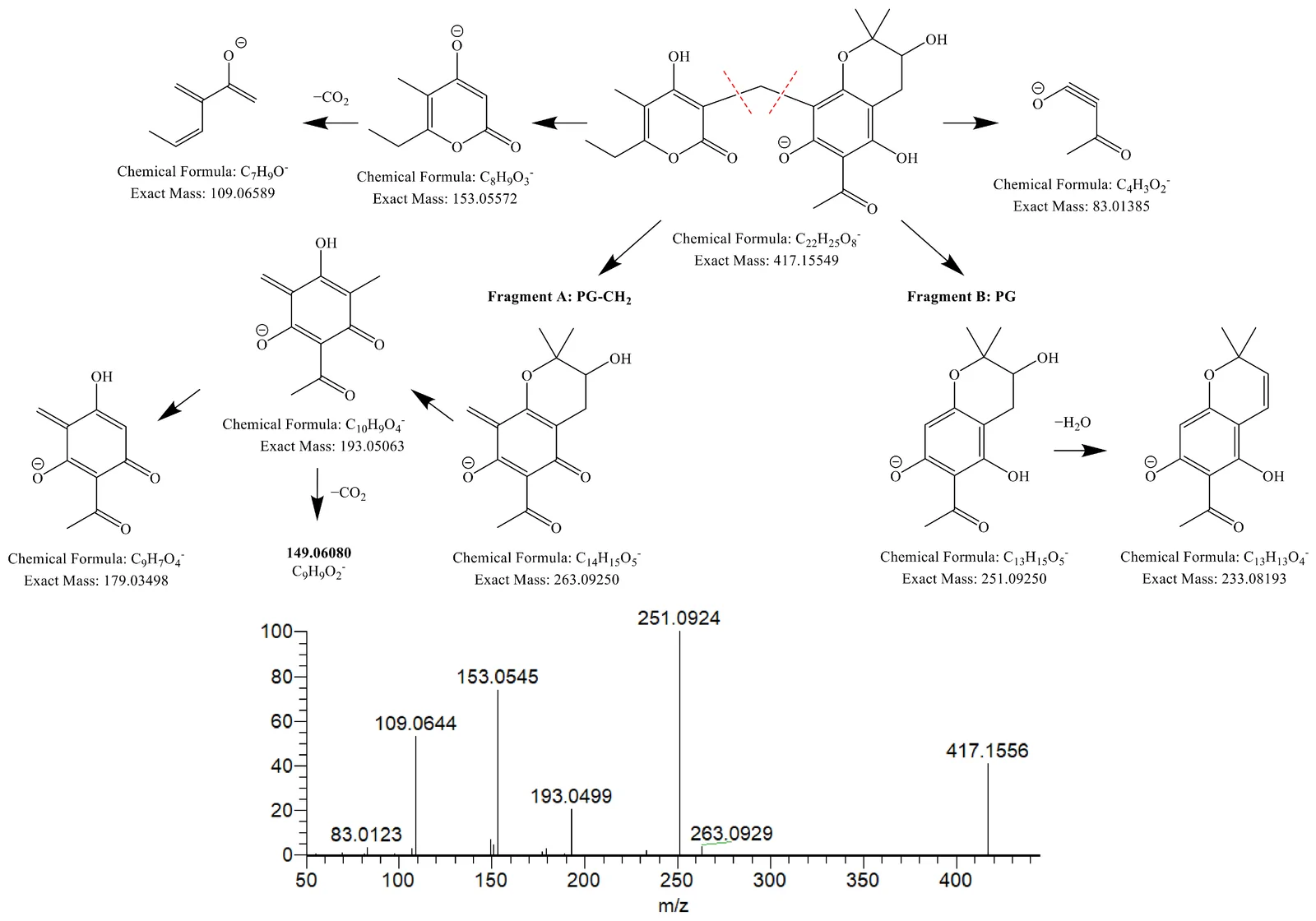
Proposed fragmentation pattern of cmpds **30**/**31** (BPs), with [M-H]^−^ at *m*/*z* 417.15549 in negative ion mode.

This is exemplified in the fragmentation scheme of cmpds **30**/**31**, with [M-H]− 417.1555 *m*/*z* (Figure 11) cleavage of the pyran moiety from Fragment A, yielding an ion at *m*/*z* 193.0506, which can decarboxylate to give a fragment with *m*/*z* 149.0608. Cmpds **51**/**52** differ from cmpds **30**/**31** by the presence of a cyclized geranyl side chain instead of a prenyl. The assignment of a cyclized geranyl moiety is supported by the observed mass differences in combination with diagnostic fragment ions confirming the remaining structural elements. In particular, the presence of the characteristic fragment ion at *m*/*z* 83.01385 confirms the C1 acyl substituent, while the α-pyrone moiety is verified by the corresponding fragment ion pairs reflecting the substitution at the C6 position. Additionally, fragment ions at *m*/*z* 179 and 193 further support the proposed structure. Taken together, these features indicate that cmpds **51**/**52** contain a cyclized geranyl side chain in place of a prenyl substituent.

Cmpd **46** differs from cmpds **51**/**52** by the presence of a double bond at the C3 side chain instead of a hydroxyl. This structural variation was inferred from characteristic mass shifts for the C3 side chain relative to the spectra of cmpds **51**/**52**.

#### 2.5.6. Spiroketals (SKs)

**Figure 12:**
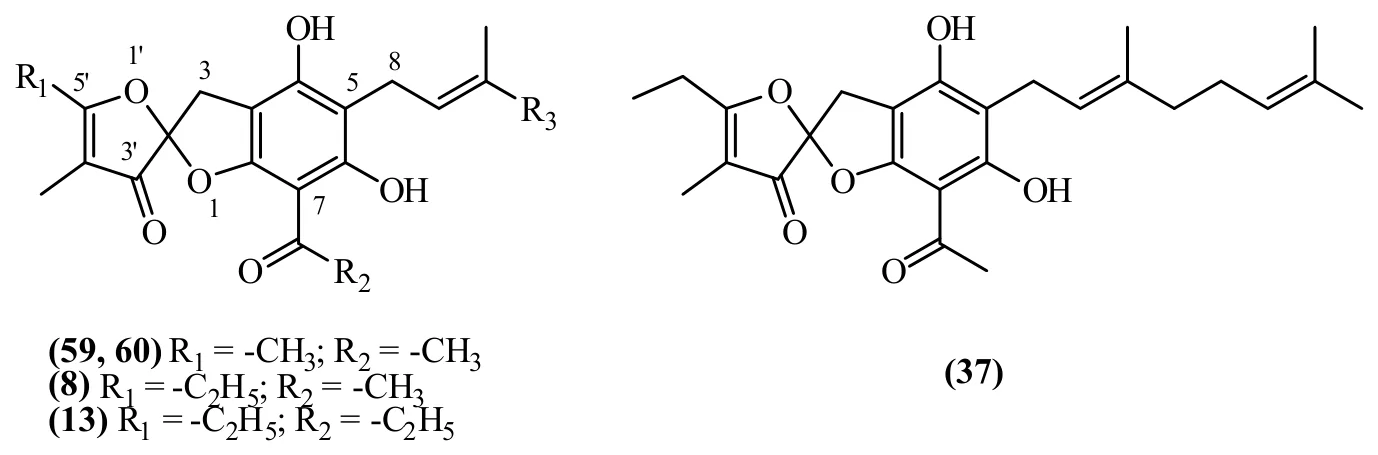
Identified SKs in *H. italicum* in this study.

Spiroketal (SK) derivatives are characterized by a 3*H*,3′*H*-spiro[benzofuran-2,2′-furan]-3′-one scaffold. They comprise a dihydrobenzofuran ring forming a spiro junction with a γ-lactone, which distinguishes SKs from phloroglucinol α-pyrone derivatives (Figure 12).

Among the spiroketals annotated in our study are helispiroketal A (C_20_H_22_O_6_, exact mass 358.1416 Da) (cmpds **59/60**) and helispiroketal F (C_21_H_24_O_6_, 372.1573 Da) (cmpd **8**).

**Figure 13:**
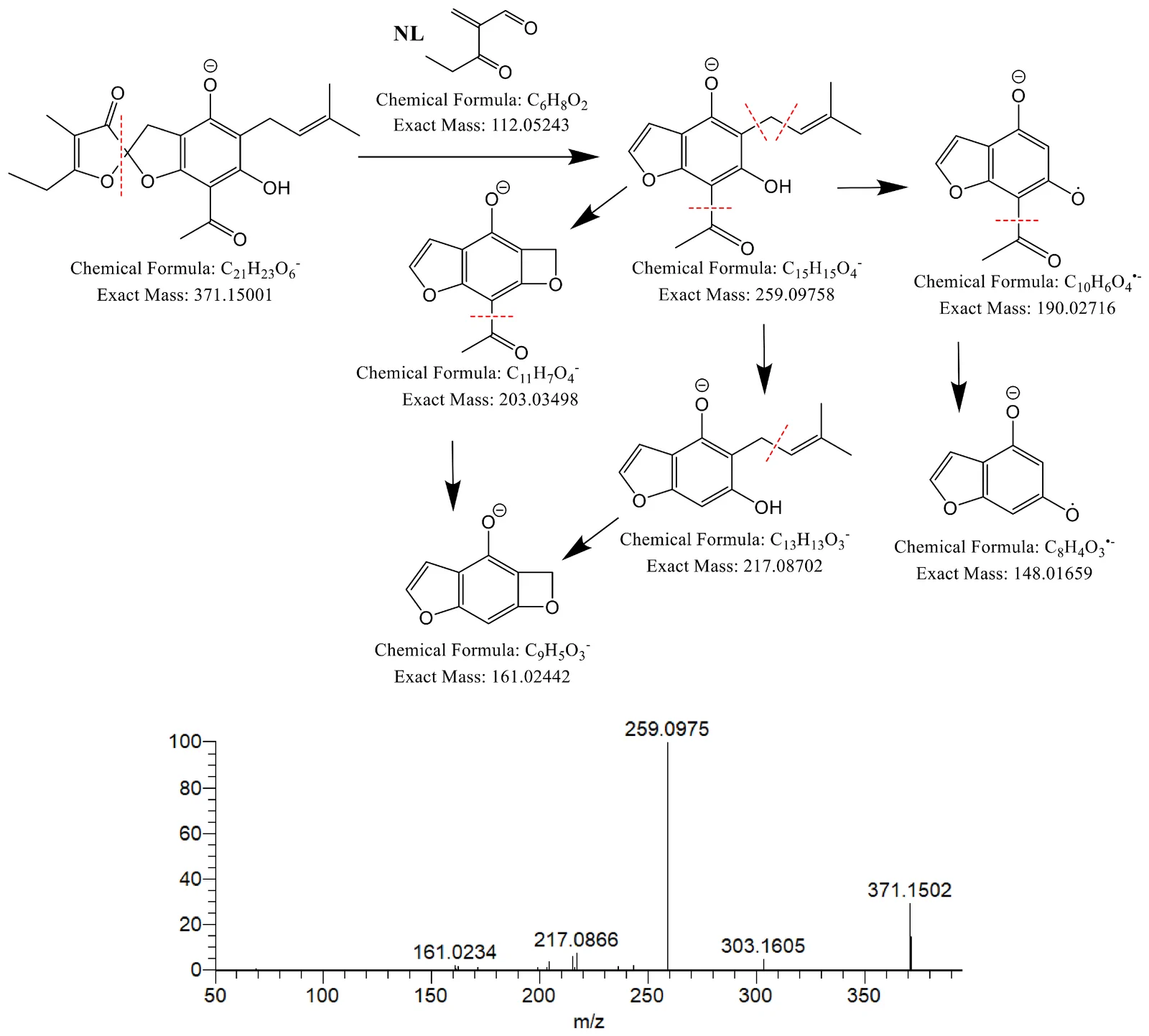
Proposed fragmentation of helispiroketal F (cmpd **8**), belonging to the SKs, with [M-H]^−^ at *m*/*z* 371.15001 in negative ion mode.

The molecular ion is typically observed at moderate relative intensity (~25–65%). The fragmentation pattern is dominated by Fragment B (PG), which appears as the base peak, whereas the complementary Fragment A (PG–CH_2_) is detected only at very low abundance (~0–5%). A notable characteristic of SK spectra is the absence of other abundant fragment ions. As exemplified in the proposed fragmentation behavior of helispiroketal F (cmpd **8**) (Figure 13), the structure of the substituted furanone moiety is deduced from the neutral loss from the molecular ion to Fragment B (PG).

A fragment ion at *m*/*z* 215.0350 is detected in all representatives except compound **13**, suggesting that its formation is associated with the presence of a C1 methyl substituent. This ion is observed specifically in the SK derivatives, although only at low relative intensity (~2–5%).

Another fragment ion at *m*/*z* 229.0506 is detected in all SK derivatives; however, its relative intensity exceeds 1% only in cmpds **13** and **37**, reaching a maximum of 5.2% in cmpd **13**. This ion differs from *m*/*z* 215.035 by 14.01565 Da (CH_2_), indicating that both fragments likely originate from closely related structures involving the C1 substituent. A fragment ion at *m*/*z* 243.0663 is detected in all spiro derivatives at low relative intensity (1–5%). Although its exact structure could not be conclusively assigned, all identified spiro derivatives share a methyl substituent at the C6′ position, while differing in the substituent at C1. This observation suggests that the *m*/*z* 243.0663 fragment originates from a structural element independent of the C1 substituent, although its precise origin remains unresolved.

### 2.6. Comparative Fragmentation Behavior Across Structural Subclasses

The fragmentation behavior of the subclasses is compared in Table 4.

The relative abundance of Fragment A separates open-chain from cyclized scaffolds. In the 33 open-chain phloroglucinol α-pyrones, Fragment A (PG–CH_2_) is observed at 57–95% relative intensity, whereas in the 23 cyclized compounds (benzofurans, benzopyrans and spiroketals), it does not exceed 15% and is frequently absent. This separation is maintained across all three α-pyrone C6 substituents and all six C1 acyl substituents present in the dataset. Fragment B (PG) is the base peak in all three cyclized subclasses and in most open-chain compounds.

The intensity of the α-pyrone ion pair resolves the cyclized subclasses from one another. The ions [α-pyr-H]^−^ and [α-pyr-CO_2_-H]^−^ reach 70–100% and 50–72%, respectively, in the benzopyrans, 16–51% and 15–33% in the benzofurans, and 6–16% and 6–20% in the open-chain series; in the spiroketals, which carry a γ-lactone in place of the α-pyrone ring, the pair is absent. In the dipyrones, the [M-H]^−^ ion is a minor peak (≤3%), and the α-pyrone pair fragment ions dominate the MS/MS spectrum.

The distribution of diagnostic ions across the subclasses is given in Supplementary Table S1.

The ion at *m*/*z* 125.0244 was detected in all nine 3-NA PG-α-pyrones and in none of the remaining 50 compounds, and the pair at *m*/*z* 205.0870/193.0870 in all fourteen 3-prenyl PG-α-pyrones and in none of the other 45. The ions at *m*/*z* 243.0663 and 215.0350 occur only in the spiroketals. The ion at *m*/*z* 137.0244 is present in every 3-NA PG-α-pyrone at 10–17% but also appears at ≤6% in seven benzopyran and geranyl derivatives.

Isobaric coincidences between subclasses occur. The ions at *m*/*z* 221.0819 and 209.0819 constitute the Fragment A/B pair of the C6-propyl 3-NA derivatives (cmpds **6** and **10**, 85–100%), while arising in the hydroxyprenyl derivatives as minor ions formed after loss of the C1 acyl group; *m*/*z* 223.0976 is likewise the Fragment B of cmpds **9**, **15** and **16** and a C3 diagnostic in cmpds **25** and **32**. The ion at *m*/*z* 203.0714, present in the geranyl derivatives, is also produced by dehydration of Fragment A in the 3-NA derivatives. These ions are consequently diagnostic only when read together with the neutral loss relating them to the precursor. The same ambiguity accounts for the weaker agreement of the fragment-ion matrix with subclass annotation in the clustering analysis presented below (Section 2.7, Section 2.8 and Section 2.9).

### 2.7. Clustering Analysis

The objective of the clustering analysis was to evaluate the extent to which MS/MS features encode chemically meaningful structural information in an unsupervised manner. In particular, the comparison between fragment-ion and neutral-loss representations was used to test whether different MS/MS data capture distinct structural determinants of phloroglucinol-derived metabolites. Rather than assuming that MS/MS similarity directly reflects overall molecular similarity, this approach examines which structural features are preserved in each representation (fragment ions or NLs).

The unsupervised clustering of MS/MS data can recover chemically interpretable groupings, but the nature of these groupings depends strongly on the feature definition.

These findings are directly relevant for MS/MS-based annotation workflows. The complementary nature of the two representations suggests that combining both feature types can improve structural annotation in complex metabolite datasets, particularly when reference spectra are limited or unavailable.

At the same time, the relatively low agreement with manual annotations highlights inherent limitations of the current approach and indicates directions for methodological improvement. First, aggregating fragment ions across compounds based solely on *m*/*z* introduces ambiguity due to isobaric fragments, which can artificially increase similarity between structurally unrelated compounds. Incorporating additional constraints, such as elemental composition filtering or fragmentation-rule-based annotation, could reduce this effect. Second, global feature pooling may obscure compound-specific fragmentation patterns; weighting schemes that account for fragment uniqueness or information content could enhance discrimination. Third, the current representation treats all retained features equally, whereas diagnostic ions or neutral losses could be prioritized based on prior knowledge or statistical importance. Finally, integrating fragment-ion and neutral-loss information within a unified framework, rather than analyzing them separately, may capture the multidimensional nature of MS/MS fragmentation.

Overall, the clustering results should be interpreted not as a direct proxy for structural classification accuracy, but as a tool for probing the relationship between molecular structure and fragmentation behavior. In this context, discrepancies between clustering and manual annotation are informative, as they reveal which structural features are emphasized or obscured by different MS/MS representations. Such insights are essential for the rational development of more robust and chemically informed MS/MS-based similarity and annotation methods.

### 2.8. Feature Selection for Fragment Ions (FIs) and Neutral Losses (NLs)

**Figure 14:**
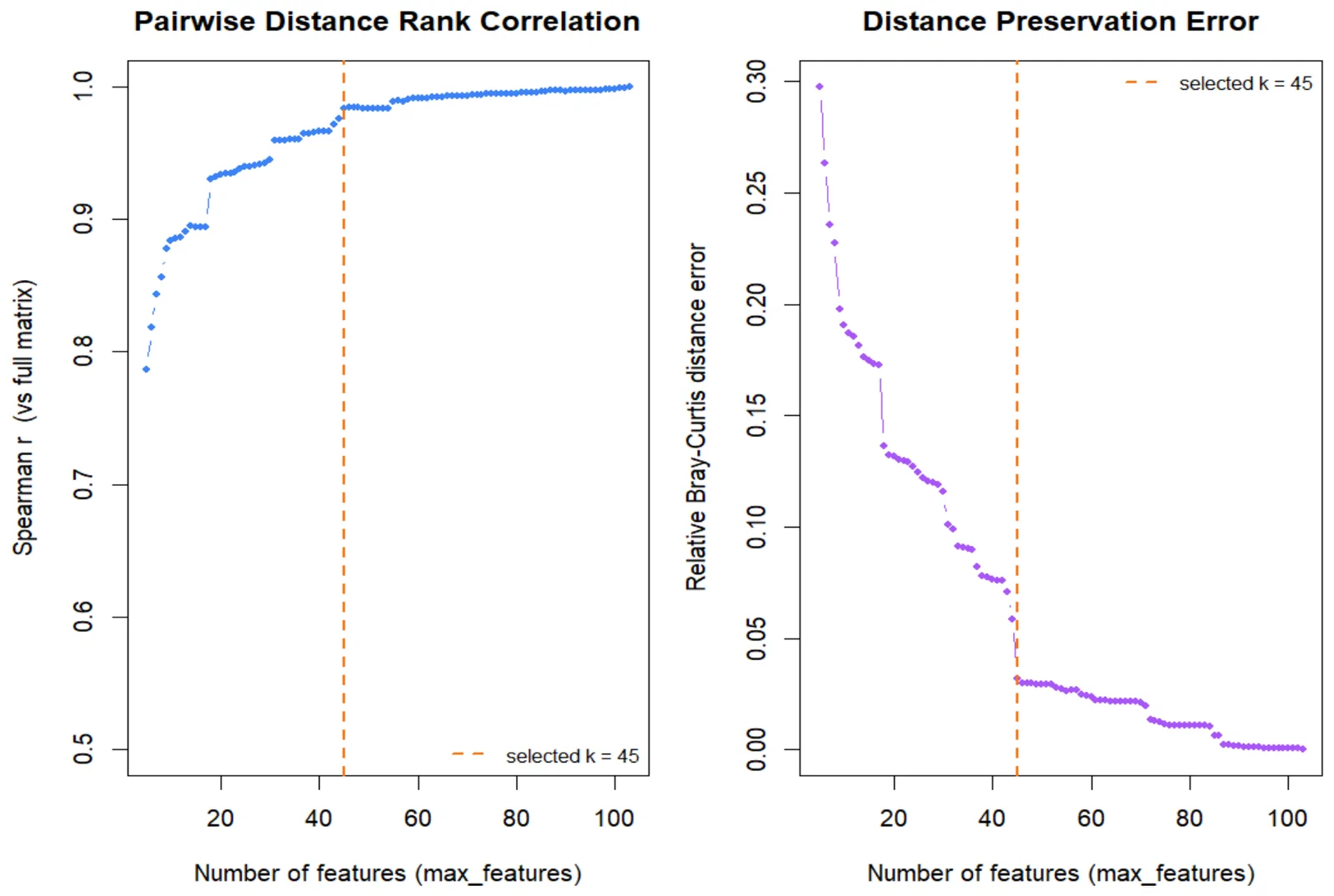
Automatic selection of the number of retained fragment-ion features. The Spearman rank-correlation curve (preservation of pairwise compound-dissimilarity rankings relative to the full candidate matrix) and the distance-error curve (deviation from the full-matrix dissimilarities) are shown as a function of the number of ranked fragment-ion features. The selected number corresponds to the elbow of the Spearman curve. FI, fragment ion; NL, neutral loss. The Spearman curve measures preservation of pairwise compound-dissimilarity rankings relative to the full candidate feature matrix.

Automatic feature-number selection was used to determine the number of fragment-ion features retained for downstream multivariate analysis. The Spearman correlation curve showed the degree to which reduced fragment-ion matrices preserved the pairwise compound-dissimilarity structure of the full candidate matrix. The complementary distance-error curve showed the corresponding deviation from the full-matrix dissimilarities. In the present dataset, the curve reached an elbow at 40 retained fragment-ion features, with a Spearman correlation of 0.9694 and a distance error of 0.0703 (Figure 14). Increasing the number of retained ions beyond this point produced only gradual improvement, indicating diminishing returns from additional features. Therefore, the selected matrix was considered a compact representation that preserved most of the global compound-to-compound dissimilarity structure while reducing feature redundancy.

This feature-selection step should not be interpreted as identifying the 40 most structurally diagnostic fragment ions individually. Rather, it identifies the smallest ranked subset that adequately reproduces the dissimilarity structure of the full fragment-ion matrix under the applied ranking and elbow criterion.

### 2.9. MS/MS-Based Clustering

**Figure 15:**
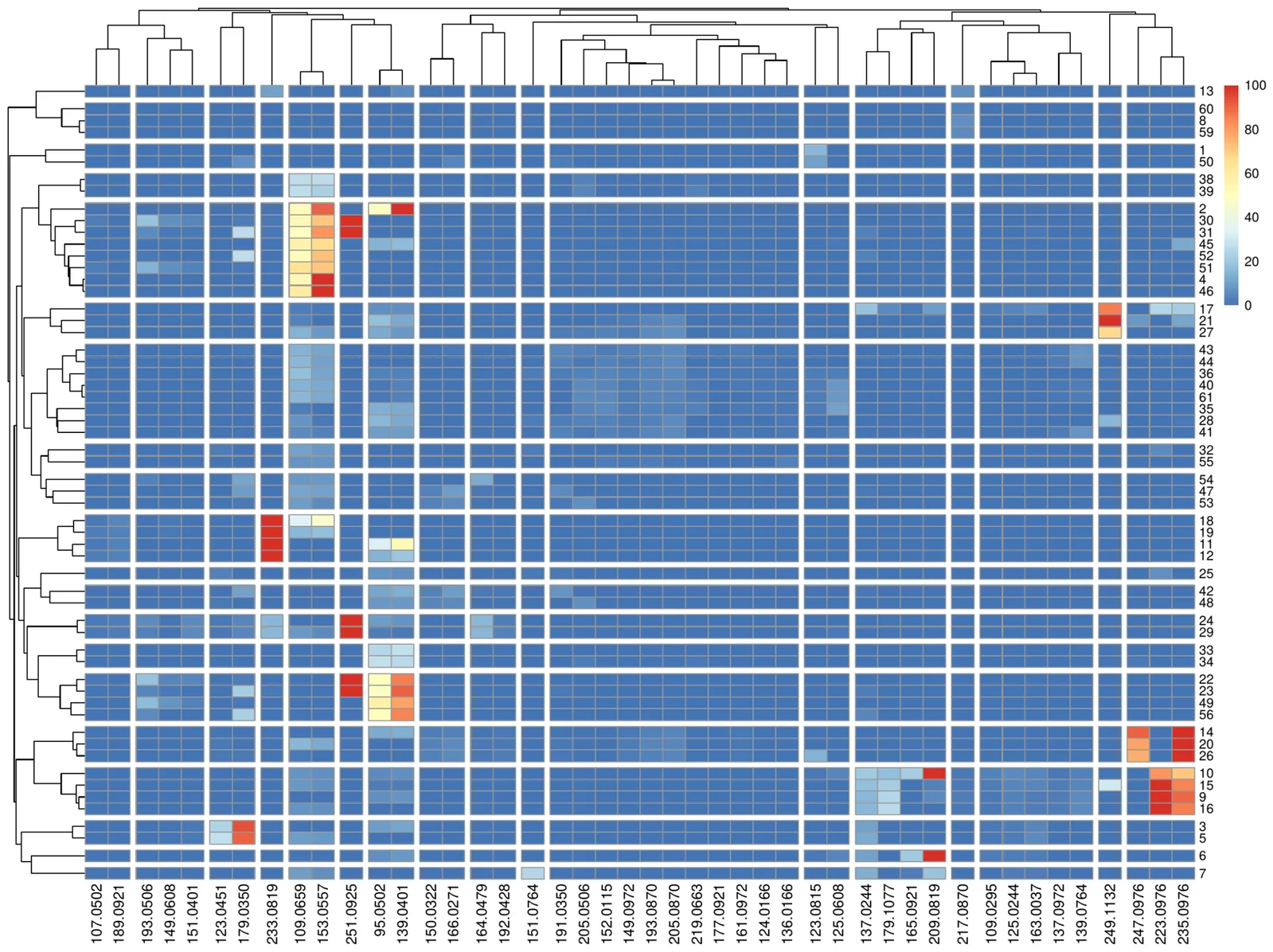
Hierarchical heatmap of the compound × fragment-ion intensity matrix. Rows represent compounds and columns the retained fragment-ion features. Cell color indicates relative fragment-ion intensity within each MS/MS spectrum (0–100%). Compounds and fragment ions were clustered independently using Bray–Curtis dissimilarity and UPGMA (average) linkage; colored row and column blocks correspond to the PAM-silhouette partition. The row dendrogram reflects compound-level similarity based on shared fragment-ion profiles; the column dendrogram groups fragment ions with similar distributions across compounds. FI, fragment ion; UPGMA, unweighted pair-group method with arithmetic mean (average linkage); PAM, partitioning around medoids.

**Figure 16:**
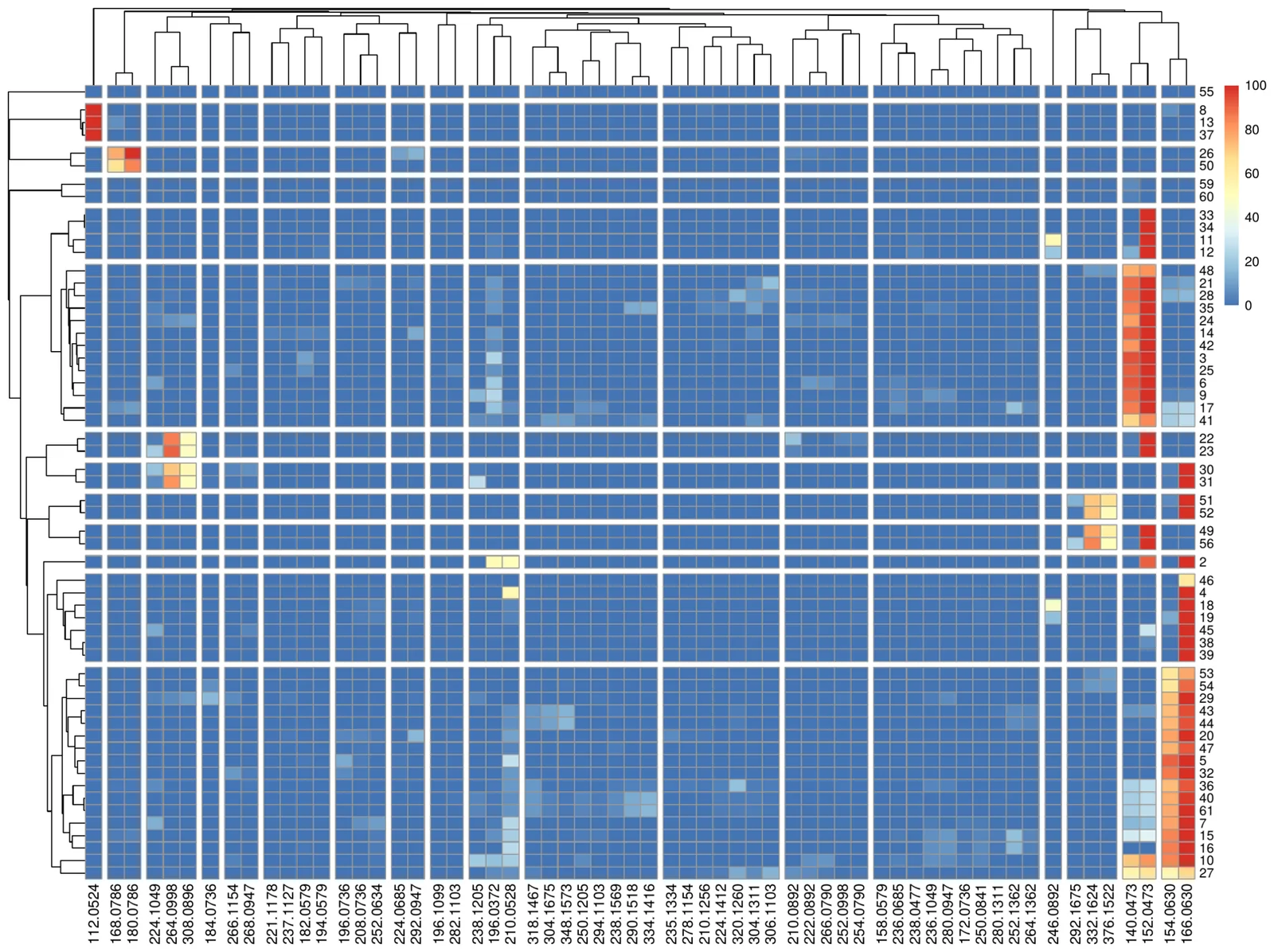
Hierarchical heatmap of the compound × neutral-loss intensity matrix. Rows represent compounds and columns the retained neutral-loss features; cell color, dissimilarity, linkage, and block coloring are as in Figure 15. Compared with the fragment-ion map, the neutral-loss map compresses the dataset into fewer, larger compound blocks, with the two largest dominated respectively by α-pyrone C6-methyl and C6-ethyl phloroglucinol derivatives. NL, neutral loss; UPGMA, unweighted pair-group method with arithmetic mean (average linkage); PAM, partitioning around medoids.

In the hierarchical heatmaps shown (Figure 15 and Figure 16), compounds (rows) and features (columns) were clustered independently on Bray–Curtis dissimilarity of relative intensities using average (UPGMA) linkage, with the number of compound clusters fixed by PAM silhouette optimization. Under these settings, the fragment-ion (FI) matrix resolved 20 compound clusters and the neutral-loss (NL) matrix 13.

The same C6-methyl and C6-ethyl compounds in the fragment-ion clustering (Figure 15) were intermixed within fragment-ion phloroglucinol–α-pyrone clusters. However, the fragment-ion clusters were informative for the C3 side chain. Several FI clusters were homogeneous in C3 substitution—cluster 7 contained only C3-unsubstituted compounds, while clusters 18, 14, and 9 were dominated by C3-prenyl derivatives and cluster 20 consisted entirely of C3-geranyl derivatives. This is consistent with the C3 substituent governing which product ions are produced and survive: prenyl and geranyl side chains generate the characteristic high-mass diagnostic series seen in the dendrogram—*m*/*z* 233.0819, 235.0976, 249.1132, 251.0925, 275.1289, 303.1602, 319.1551—whereas ions *m*/*z* 109.0659, 139.0401, 153.0557, 179.0350, 193.0506 were characteristic of the benzopyran clusters. Accordingly, fragment-ion clustering agreed better than neutral-loss clustering with the C3 substitution pattern (ARI 0.201 vs. 0.025; Table 5).

The most informative result concerns the predominant phloroglucinol–α-pyrone class (*n* = 33). In the neutral-loss heatmap (Figure 16), this class collapsed into two large, internally near-perfect blocks: cluster 2 (*n* = 13) consisted exclusively of α-pyrone C6-methyl derivatives and cluster 3 (*n* = 11) exclusively of C6-ethyl derivatives. Neutral-loss clustering separated the entire phloroglucinol–α-pyrone class according to the alkyl substituent on the α-pyrone ring, with essentially no cross-contamination. The C6 alkyl group manifests as a recurrent, conserved mass-difference feature. The C6-substituent agreement was higher for neutral losses than for fragment ions (ARI 0.264 vs. 0.043; Table 5).

The two representations were informative at different structural levels. Neutral-loss clustering reproduced the structural subclass better than fragment-ion clustering (best ARI 0.424 vs. 0.145), while fragment-ion clustering was more informative for the C3 substituent. The C1 phloroglucinol substituent was poorly recovered by both representations (FI 0.067, NL 0.001).

Both representations nonetheless retained high internal coherence: clustering purity at the subclass level was 0.948 for both feature spaces, confirming that the clusters are chemically meaningful even where their correspondence to a particular annotation level is limited. Fragment-ion spectra in this compound family are dominated by a small number of abundant product ions arising from cleavage of the methylene bridge, yielding the phloroglucinol (Fragment B) and phloroglucinol–CH_2_ (Fragment A) units; because these ions are shared across subclasses, fragment-ion profiles converge, and scaffold-level distinctions are blurred.

While neutral-loss clustering recovered scaffold-specific groups—benzofurans in cluster 6, spiroketals in clusters 4 and 13, and benzopyrans in clusters 7, 9, 10, and 11—its cluster 1 united eight compounds of three different nominal scaffolds (four benzofurans, two benzopyrans, and two dipyrones). These compounds were grouped together despite belonging to different scaffolds, indicating that neutral-loss similarity does not always coincide with scaffold identity. The benzopyran class was the least stably recovered by either representation, fragmenting into three FI clusters and five small NL clusters.

Given that fragment-ion representations are prone to isobaric ambiguity, structurally distinct fragments from different precursors may share identical *m*/*z* and be aggregated as a single feature, artificially inflating similarity between compounds of different classes; specific instances among the diagnostic ions are presented in Supplementary Table S1. Neutral-loss features are less affected because they depend on the precursor–fragment relationship, though coincidental mass equivalence may still occur. The combined use of fragment-ion and neutral-loss matrices therefore provides a more complete description of the structural information contained in the MS/MS data.

### 2.10. Relative Distribution of PG-α-Pyrones in H. italicum and Comparison with Literature Data

**Figure 17:**
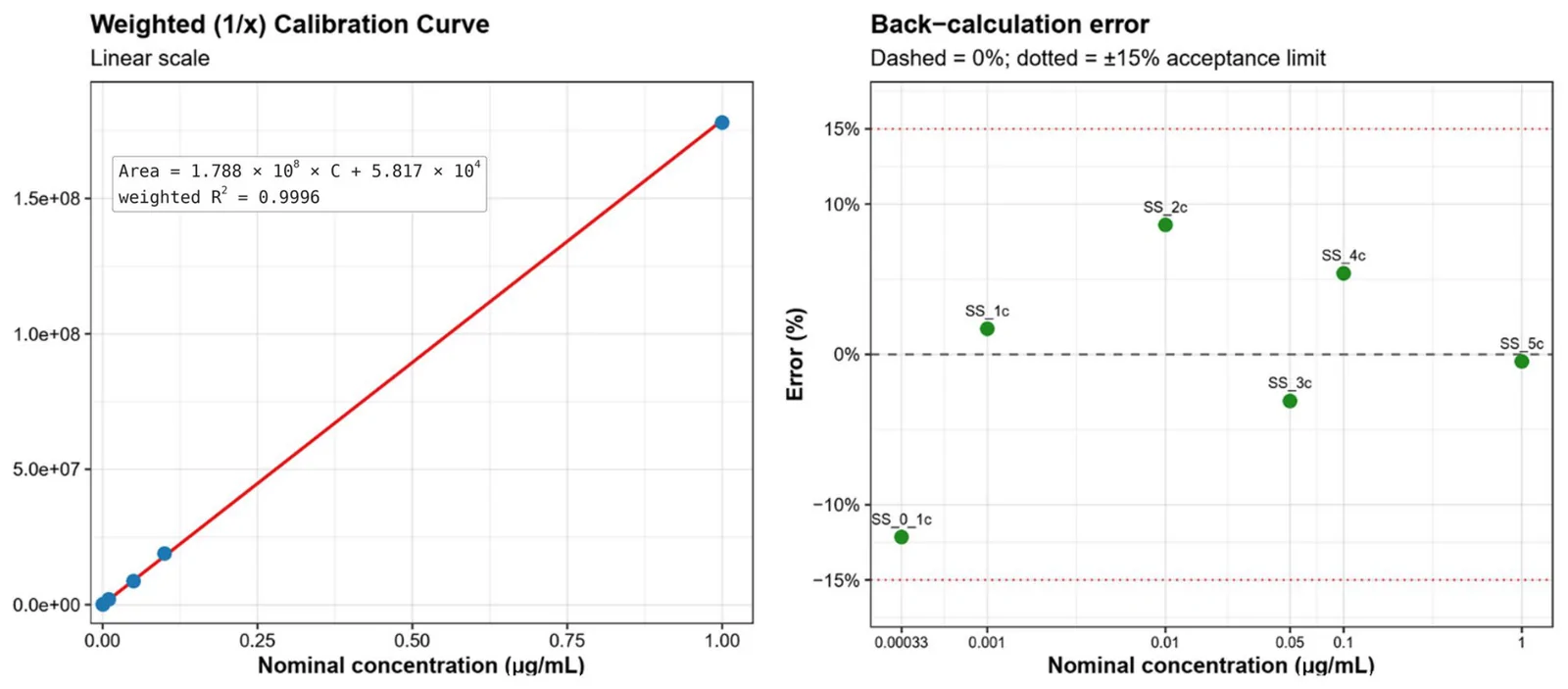
Calibration data for the semi-quantitative assessment of phloroglucinol α-pyrones. Left: Weighted (1/x) external calibration curve constructed with 2′,4′,6′-trihydroxyacetophenone monohydrate over the range 0.000330–1.000 µg/mL. The fitted model was Area = b1 × C + b0, where C is the concentration in the injected vial. Right: Back-calculation error for each calibration level. b0, intercept and b1, slope of the fitted calibration model; C, concentration in the injected vial.

The semi-quantitative data obtained for *H. italicum* show a total PG-α-pyrone content of approximately 0.01915% DW, expressed as 2,4,6-trihydroxyacetophenone equivalents. These data are LC–HRMS response-based estimates calculated against a single external standard and should therefore be regarded as semi-quantitative. Literature values, in contrast, generally represent isolated and purified compound yields after extraction and chromatographic fractionation. Therefore, the comparison is most useful for evaluating the order of magnitude, relative abundance, and whether the detected compounds fall within the known quantitative range reported for *Helichrysum* PG-α-pyrones. The external calibration curve and the corresponding back-calculation errors are given in Figure 17, and the compound-wise semi-quantitative distribution is presented in Table 6. For compounds detected as two chromatographically resolved peaks with identical elemental composition and fragmentation (isomer pairs, e.g., cmpds **18**/**19**, **30**/**31**, **38**/**39**, **43**/**44**, and **59**/**60**), the peak areas of both isomers were summed, and the reported content refers to the pair rather than to a single peak.

The total PG-α-pyrone pool is dominated by a small number of compounds. Arzanol (cmpd **20**) was the major component, representing 0.00542% DW and 28.29% of the total semi-quantified PG-α-pyrone pool. This value is higher than the low arzanol yield reported for *H. italicum* (Roth) G. Don (0.002% *w*/*w*) [12], but lower than the arzanol-rich reports from *H. italicum* ssp. *microphyllum* (0.081–0.295% *w*/*w*) [10,11,33] and from *H. stoechas* flowers (0.48% *w*/*w*) [14]. Thus, the present extract contains arzanol as the dominant PG-α-pyrone, but not at the extremely high levels reported for the most arzanol-rich plant sources.

The only monopyrone detected in our study, micropyrone (cmpd **1**), was estimated at 0.000059% DW, which is below the reported isolation yields from *H. italicum* and *H. italicum* ssp. *microphyllum* aerial parts (0.0015–0.0482% *w*/*w*) [10,11,12,33]. Helipyrone B (cmpd **2**) was present at 0.0000128% DW, similar in magnitude to the very low yield reported from *H. oocephalum* (0.00001% *w*/*w*) [5]. Helipyrone A (cmpd **4**) was estimated at 0.0000558% DW, close to the lower end of the literature range (0.00007–0.041% *w*/*w*) and far below the higher values reported from *H. italicum* ssp. *microphyllum* (0.022–0.0256% *w*/*w*) [5,10,11,14,15,33]. These comparisons indicate that, in the present extract, the simple monopyrone-/dipyrone-related compounds are minor constituents relative to the dominant prenylated PG-α-pyrone series.

The spiroketal group was also quantitatively minor. The total spiroketal subclass content was 0.00125% DW, representing approximately 6.5% of the total PG-α-pyrone pool. Helispiroketal F (cmpd **8**) was estimated at 0.000679% DW, while helispiroketal A (cmpds **59**/**60**) was estimated at 0.000476% DW. Reported isolated yields of helispiroketals are generally extremely low, mostly around 0.000018–0.000055% *w*/*w* [5].

Among benzofuran and benzopyran derivatives, several compounds were present at low-to-moderate levels. Italipyrone-related features (cmpds **18**/**19**) together accounted for 0.000246% DW, higher than the reported isolated yield of italipyrone from commercially sourced *H. italicum* (0.000068% *w*/*w*) [8]. Plicatipyrone-related features (cmpds **30**/**31**) together accounted for 0.000267% DW, close to the reported yield of plicatipyrone from *H. plicatum* flower heads (0.00022% *w*/*w*) [8]. These values suggest that some cyclized PG-α-pyrone derivatives occur at levels comparable to published isolation yields, even though they are not major contributors to the total pool.

The 3-prenyl and higher-alkylated PG-α-pyrone analogs showed a broad quantitative range. Compound **35**, assigned as arenol C, was estimated at 0.000630% DW, which is higher than the very low yield reported from *H. oocephalum* (0.00002% *w*/*w*) [5]. The isomeric feature assigned as 6-O-desmethylauricepyrone/3-prenyl norauricepyrone was estimated at 0.000491% DW. This is higher than the yield reported for 3-prenyl norauricepyrone from *H. oocephalum* (0.00001% *w*/*w*) [5], but far below the high yield reported for 6-O-desmethylauricepyrone from *H. odoratissimum* (0.133% *w*/*w*) [8]. Similarly, cmpd **40**, assigned as 23-methyl-6-O-desmethylauricepyrone, reached 0.000739% DW, far below the literature yields reported from *H. odoratissimum* (0.033% *w*/*w*), *H. mixtum* roots (0.07% *w*/*w*), and *H. stenopterum* aerial parts (0.09% *w*/*w*) [8,18]. This indicates that the present *H. italicum* profile contains these analogs, but not at the high levels reported in certain non-*italicum* species or root-rich materials.

The achyroclinopyrone-related compounds were among the more relevant higher-mass components. Compound **42**, assigned as 18,18-bis-desmethyl achyroclinopyrone C, was estimated at 0.00134% DW, below the high yield reported from *H. stoechas* (0.0857% *w*/*w*) [14] and also lower than the yield from *H. decumbens* (0.0032% *w*/*w*) [19]. In contrast, compound 47, assigned as 18,18-bis-desmethyl achyroclinopyrone A, reached 0.00177% DW, exceeding the reported *H. decumbens* yield (0.0008% *w*/*w*) [19]. These comparisons suggest that the present extract contains quantitatively relevant amounts of achyroclinopyrone-type analogs, although still below the highest reported levels in *H. stoechas*.

Overall, comparison with the literature supports the conclusion that the present *H. italicum* extract is characterized by a broad PG-α-pyrone profile with one dominant arzanol-type component and multiple lower-abundance analogs. The total of 0.01915% DW is within the same order of magnitude as several reported isolated PG-α-pyrone yields, but below the exceptionally high arzanol yields reported for selected *H. stoechas* [14] and *H. italicum* ssp. *microphyllum* materials [10,11,33]. The present data therefore support the role of *H. italicum* as a rich source of PG-α-pyrones, while also showing that its quantitative profile depends strongly on the specific compound subclass and plant source. A compound-by-compound comparison with previously reported isolation yields is given in Table A2.

### 2.11. Limitations

The diagnostic rules established in this paper were not verified against authentic standards representing each structural subclass, since no such reference material is commercially available; only arzanol is obtainable, and it was not available for this study. The rules rest instead on the accurate-mass elemental composition of every diagnostic ion, on the internal consistency of the homologous fragment series across substituent variants, on the complementarity of fragment ions and neutral losses arising from the same cleavage, and on their ability to reproduce the substitution patterns of the 23 compounds whose structures were previously established by NMR.

Structural assignments in this study correspond to Level 2b of the Schymanski scheme [36]—probable structures supported by accurate mass and diagnostic MS/MS—rather than Level 1 identifications, as the individual α-pyrones were not isolated and no authentic reference standards were available. Consequently, MS/MS-based annotation cannot establish stereochemistry, and where indicated in the text, the position of side-chain hydroxylation was inferred by analogy to reported structures. In several cases, two chromatographically resolved compounds shared the same elemental composition and the same diagnostic fragmentation (denoted as pairs, e.g., **11**/**12**, **30**/**31**, **38**/**39**, **59**/**60**). For these, the MS/MS data establish the scaffold and substituents and are consistent with the previously reported structure, but do not indicate which of the two peaks corresponds to it. The trivial name is therefore given to both members to signal that one of the pair is the reported compound while the other is an unresolved isomer.

Quantification was performed against a single external standard (2′,4′,6′-trihydroxyacetophenone). Because authentic reference standards were unavailable for the annotated compounds, no analyte-specific determination of accuracy, precision or recovery was possible, and no analyte-free matrix of the species exists against which matrix-matched recovery could be established. The quantitative data are therefore presented as a relative distribution of the compound class within a single extract, not as validated concentrations, and comparison with isolation yields reported in the literature is restricted to order of magnitude.

The unsupervised clustering is intended as an exploratory, hypothesis-generating analysis of the relationship between molecular structure and fragmentation behavior, not as an independent validation of the structural annotations. Agreement with the manual annotation was modest (adjusted Rand index 0.145–0.424), and each feature representation corresponded to only a subset of structural levels. The high clustering purity (0.948) largely reflects the dominance of a few shared fragment ions and neutral losses arising from cleavage of the methylene bridge, rather than fine structural discrimination; the results should be interpreted accordingly.

The study was performed on a single commercial batch of dried inflorescences. No voucher specimen with collection data, harvest date or documented drying regime is available.

The extraction protocol was selected on established grounds for this compound class rather than optimized by a formal experimental design; solvent, extraction time, solid-to-liquid ratio and cycle number were not varied systematically. The contents reported are those recoverable under the stated conditions.

## 3. Materials and Methods

### 3.1. Plant Material and Sample Preparation

The aerial-dried inflorescences of *Helichrysum italicum* (Roth) G. Don were obtained from a commercial supplier (Greekherbay, Patras, Greece) as a standard dried herbal drug. Botanical identity was confirmed by one of the authors (PN). On receipt, the drug was stored in a sealed, light-protected container at 5 °C. For sample preparation, the dried plant material was mechanically ground to a particle size of approximately 0.3 mm. An accurately weighed portion of the powdered plant material (~1 g) was transferred into a 100 mL glass flask and extracted with 20 mL of acetone (100%, *v*/*v*). Extraction was performed by ultrasonication (15 min), followed by orbital stirring (600 rpm, 15 min). The procedure was repeated three times (3 × 20 mL). The combined extracts were filtered through qualitative filter paper and evaporated to dryness under vacuum.

The resulting residue (~85 mg) was quantitatively transferred with methanol (MeOH, 100%, *v*/*v*) into a 10 mL volumetric flask and diluted with the same solvent.

For LC–HRMS analysis, 100 µL of the latter solution was diluted to 1.0 mL with MeOH. The solution was filtered through a 0.45 µm syringe filter, and 1 μL was injected into the UHPLC–HRMS instrument.

### 3.2. UHPLC-HRMS Instrument

UHPLC–HRMS analyses were performed using a Q Exactive Plus Orbitrap MS (Thermo Fisher Scientific, Karlsruhe, Germany) equipped with a heated electrospray ionization (HESI-II) source. The chromatographic system consisted of a Dionex UltiMate 3000 RSLC comprising an SRD-3600 degasser, an HPG-3400RS binary pump with solvent selection valve, a thermostatted autosampler (WPS-3000TRS), and a column compartment (TCC-3000RS) (Thermo Fisher Scientific, Germany). Chromatographic separation was achieved using a Kinetex EVO C18 1.7 µm analytical column coupled with a SecurityGuard ULTRA EVO C18 guard cartridge (Phenomenex, Torrance, CA, USA).

### 3.3. Chromatographic Parameters

The mobile phase consisted of water containing 0.1% formic acid (A) and acetonitrile/water (95:5, *v*/*v*) containing 0.1% formic acid (B). The flow rate was maintained at 0.3 mL/min. The gradient program was as follows: 30% B at 0.5 min, increased to 40% B at 2.0 min, 80% B at 24.0 min, and 95% B at 27.0 min. This composition was held until 29.5 min, followed by re-equilibration to 50% B at 30.0 min.

### 3.4. Mass Spectrometric Parameters

Mass spectrometric detection was performed in negative ion mode using full-scan (FS) and data-dependent MS/MS (DD-MS^2^) acquisition over an acquisition window of 0.9–29.06 min, with in-source CID disabled and a default charge state of 1.

Full-scan spectra were acquired at a resolution of 35,000 (FWHM at *m*/*z* 200) with one microscan, an AGC target of 1 × 10^6^, a maximum injection time of 128 ms and a scan range of *m*/*z* 150–1000, in profile mode.

Data-dependent MS/MS spectra were acquired in Top5 mode at a resolution of 17,500 with one microscan, an AGC target of 1 × 10^5^, a maximum injection time of 64 ms, a loop count of 5, an MSX count of 1 and an isolation window of 0.4 *m*/*z* with zero isolation offset; no fixed first mass was set. Fragmentation was performed using stepped normalized collision energies of 20 and 50 (NCE, %). Precursor selection used a minimum AGC target of 2.5 × 10^3^, corresponding to an intensity threshold of 3.9 × 10^4^, with apex triggering between 2 and 4 s and dynamic exclusion of 5.0 s. Charge-state exclusion and isotope exclusion were not applied, no inclusion or exclusion list was used, and the instrument was configured not to select additional precursors when idle.

Extracted-ion chromatograms were generated at a mass tolerance of ± 5 ppm from full-scan data acquired at a resolving power of 35,000 (*m*/*z* 200), and data-dependent MS^2^ used an isolation window of 0.4 *m*/*z*. Peak homogeneity was confirmed by the constancy of the accurate mass and of the relative intensities of the diagnostic fragment ions across the leading edge, apex and trailing edge of each integrated peak. Chromatographically resolved isobaric isomers were not merged but are reported as separate entries (Table 6 and Table A1).

### 3.5. Data Processing

Vendor *.raw (Thermo Fisher Scientific) files were converted to *.ms1 (MS1 data) and *.mgf (MS2 data) files by msConvertGUI 3.0 (ProteoWizard) and imported into the R programming language (4.5.2. “Part in a Rumble”) operated from RStudio (2026.01.1+403 “Apple Blossom”). MS1 data below 1 × 10^4^ intensity and MS2 data below 8 × 10^3^ intensity were excluded.

### 3.6. Annotation Confidence

Structural assignments were graded according to the confidence-level scheme of Schymanski et al. [36]. Compounds were annotated by combining accurate-mass elemental composition (mass error usually < 2–3 ppm) with diagnostic HCD fragmentation that defined the structural subclass and the α-pyrone C6, C1-acyl, and C3 substituents through the fragment-ion and neutral-loss series established in Section 2.1, Section 2.2, Section 2.3 and Section 2.4. Assignments are reported at confidence Level 2b (plausible structure), although no individual α-pyrone was verified against an authentic reference standard and MS/MS-based annotation does not establish stereochemistry.

### 3.7. Matrix Effect

Matrix effects were evaluated by comparing the signal response of calibration standards prepared in solvent with those prepared in a plant matrix. The matrix consisted of an extract of *Achyrocline satureioides* (Lam.) DC., prepared under identical conditions to the *Helichrysum italicum* extract. This alternative matrix was selected because the target analyte is naturally present in *H. italicum*, precluding the use of matrix-matched calibration in the same extract. The signal response in the matrix ranged between 95% and 105% relative to the solvent-based calibration, indicating negligible matrix effects. Consequently, external calibration in solvent was employed for subsequent quantitative analysis.

### 3.8. Semi-Quantitative Assessment of Phloroglucinol α-Pyrones

Phloroglucinol α-pyrones were assessed semi-quantitatively using 2′,4′,6′-trihydroxyacetophenone monohydrate (BLDPharm, Kaiserslautern, Germany; Cat No. BD33753-5g) as an external calibration standard. Quantification was based on chromatographic peak areas obtained from extracted-ion chromatograms of the corresponding deprotonated molecular ions. External calibration was performed over the range 0.000330–1.000 µg/mL using six concentration levels. The calibration model was fitted by 1/x weighted linear regression according to $A=b_{0}+b_{1}C$, where A is the peak area and C is the concentration in the injected vial. Concentrations of individual compounds were calculated as $C_{\mathrm{vial}}=(A-b_{0})/b_{1}$. The calculated concentrations were then converted to µg/g dry weight and % dry weight according to the sample-preparation dilution scheme. The original dry extract was dissolved in 10 mL methanol, and 100 µL of this solution was further diluted to 1.0 mL before UHPLC–HRMS analysis, corresponding to a 10-fold conversion from the injected vial to the original extract solution.

The limit of quantification (LOQ) was determined empirically as the lowest calibration level at which the back-calculated concentration remained within ± 20% of nominal. The LOQ was accordingly set at 0.000330 µg/mL and the LOD estimated as one third of that value (0.000110 µg/mL). Compounds below the LOQ or above the upper calibration level were flagged accordingly. All calculations, back-calculation of calibration levels, concentration conversion, and graphical outputs were performed in R.

Where two chromatographically resolved peaks shared the same elemental composition and the same diagnostic fragmentation and were therefore assigned to the same presumed structure (isomer pairs, e.g., cmpds **18**/**19**, **30**/**31**, **38**/**39**, **43**/**44**, and **59**/**60**), their extracted-ion peak areas were summed prior to quantification, and the combined value is reported for the corresponding trivial name in Table A2; the two peaks are nevertheless listed separately in Table 6 and Table A1.

### 3.9. Estimation of Recovery

Recovery was evaluated by spiking an extract of *Achyrocline satureioides* with 2′,4′,6′-trihydroxyacetophenone monohydrate at concentrations of 0.1 and 0.001 µg mL^−1^. The corresponding recoveries were 75% and 85%, respectively. The mean recovery (80%) was subsequently applied as a correction factor for quantitative results.

### 3.10. Fragment-Ion Feature Selection and Matrix Construction

Fragment ions detected across all annotated compounds were pooled. Fragment-ion features were initially retained if they were detected in at least two compounds and exhibited a minimum relative intensity of 0.5%. Reference fragments were retained when applicable. Non-detected fragments were encoded as zero.

To reduce dimensionality, a feature-selection procedure was applied. Candidate fragment ions were ranked by priority, with reference fragments retained first, followed by fragments detected in a larger number of compounds and then by decreasing maximum relative intensity across compounds. Reduced matrices containing progressively increasing numbers of ranked fragment ions were generated and compared with the full candidate matrix. For each reduced matrix, pairwise compound dissimilarities were compared with those obtained from the full candidate matrix using Spearman rank correlation. A complementary distance-error metric was also calculated to estimate the deviation of the reduced-matrix dissimilarities from the full-matrix dissimilarities.

The optimal number of retained fragment-ion features was selected as the elbow point of the Spearman-correlation curve, corresponding to a compromise between matrix compactness and preservation of the original dissimilarity structure. When an upper feature cap was specified, the final number of retained fragment ions was defined as the smaller value between the user-defined cap and the automatically selected optimum. The number of retained fragment ions was capped at 60, but the final number was selected automatically as the smaller value between this cap and the estimated elbow-point optimum. Compounds with no retained fragment ions in the final selected feature set were retained in the matrix as zero rows to preserve compound identity but were excluded from active clustering and assigned to an auxiliary category (“the rest”) during downstream interpretation.

### 3.11. Dissimilarity Analysis and Clustering

All dissimilarities were calculated in R using the vegdist function (method = “bray”) implemented in the *vegan* (version 2.7-5) package [37]. When data were binarized (presence/absence), Jaccard dissimilarity was used.

Pairwise dissimilarities among compounds were calculated using the Bray–Curtis index implemented in the *vegdist* function of the R package *vegan* (version 2.7-5) [37]. Fragment ion relationships were evaluated analogously using the transposed matrix.

Hierarchical clustering was performed on both the compound and fragment distance matrices using the unweighted pair-group method with arithmetic mean (UPGMA; average linkage), implemented via *hclust* in base R. Ward linkage was not used due to incompatibility with non-Euclidean dissimilarities. The quality of the resulting dendrogram was assessed by the cophenetic correlation coefficient, calculated as the Pearson correlation between the original dissimilarity matrix and the cophenetic distance matrix derived from the dendrogram.

Compounds with no detected fragment ions across the selected feature set were excluded from the active clustering and subsequently assigned to an auxiliary category (“the rest”).

### 3.12. Determination of Optimal Cluster Number

The optimal number of clusters was determined independently for compounds and fragment ions using Partitioning Around Medoids (PAM) applied to the precomputed Bray–Curtis dissimilarity matrix. For each candidate k, the average silhouette width was calculated. The optimal number of clusters was defined as the smallest k within a tolerance of 0.02 of the maximum average silhouette width, thereby avoiding over-partitioning while retaining near-optimal clustering quality. The range of k values evaluated was constrained to 2 ≤ k ≤ min (20, n − 1), where n denotes the number of compounds or fragment ions. Final cluster membership was assigned using PAM at the selected k.

### 3.13. Clustering Visualizations

The fragment-ion intensity matrix was Hellinger-transformed by dividing each row by its sum and taking the square root. This transformation down-weights highly abundant fragment ions and reduces the influence of double-zeros. 

A hierarchical heatmap was constructed using the *pheatmap* package (version 1.0.13) [38], with both compounds (rows) and fragment ions (columns) clustered using Bray–Curtis dissimilarity and UPGMA linkage. The optimal number of clusters was determined separately for compounds and fragment ions using PAM applied to the Bray–Curtis dissimilarity matrix, with the number of clusters selected based on the average silhouette width. Compound dendrograms were visualized using fviz_dend. Pairwise Bray–Curtis dissimilarities among compounds were additionally displayed as distance heatmaps to provide a direct visual assessment of inter-compound similarity structure.

### 3.14. Validation of the MS/MS-Based Clustering

Cluster assignments obtained from the fragment-ion matrix and the neutral-loss matrix were evaluated against the manually curated reference annotation table. The reference annotations comprised the main structural scaffold classification (subclass; monopyrones, dipyrones, phloroglucinol α-pyrones, benzofuran-, benzopyran-, and spiroketal derivatives) and three substituent-level descriptors: C6_a_pyr (substitution at C6′ of the α-pyrone), C1_pg (substitution at C1 of the acetophenone moiety), and C3_pg (substitution at C3 of the acetophenone moiety).

Cluster memberships generated by PAM, hierarchical clustering, and *k*-means were merged with the reference table using the compound identifier (№). External validation was performed using the adjusted Rand index (ARI) and overall clustering purity, calculated as the proportion of compounds belonging to the most frequent structural class within their respective cluster. Values range from greater than 0 to 1 (with the lower bound depending on cluster size), with higher values indicating greater class homogeneity within clusters. Compounds assigned to the auxiliary category “the rest” were excluded from the analysis. Because some substituent-level annotations were not defined for all compounds, the effective sample size depended on the descriptor used and ranged from 58 compounds for the scaffold-level classification (notes) to **57**–**58** for C6_a_pyr, **55**–**56** for C1_pg, and **47**–**48** for C3_pg.

For the fragment-ion dataset, PAM clustering produced 19 clusters plus one residual entry (compound **37**), whereas neutral-loss clustering produced 13 clusters plus one residual entry (compound **1**). Cluster composition was examined by tabulating the number of compounds from each structural class within every PAM cluster. Agreement between fragment-ion- and neutral-loss-based clustering was additionally evaluated using ARI in order to determine whether both feature spaces captured similar or complementary structural information.

## 4. Conclusions

This study provides the first comprehensive UHPLC–Orbitrap–MS characterization of the phloroglucinol α-pyrone metabolites of *Helichrysum italicum*. Fifty-nine compounds were profiled and structurally annotated (confidence level 2b) through exact-mass elemental composition assignment combined with a systematic interpretation of HCD fragmentation. Of these, 23 were annotated as known PG-α-pyrones previously reported from *Helichrysum* species, consistent with the HRMS/MS data, whereas 36 compounds—comprising 22 phloroglucinol–α-pyrones, 8 benzopyrans, 4 benzofurans and 2 spiroketals—correspond to structures not previously described from the genus and are characterized here for the first time. Several of these newly described structures, including the second most abundant constituent of the extract, are major components of the PG-α-pyrone pool rather than trace metabolites. Diagnostic fragment ions and complementary neutral losses were defined for the α-pyrone C6 alkyl group, the C1 acyl chain, and the C3 prenyl/geranyl side chain, enabling substituent-level structural assignment across monopyrone, dipyrone, 3-non-alkylated, prenyl, geranyl, benzofuran, benzopyran, and spiroketal subclasses. Unsupervised clustering showed that fragment-ion and neutral-loss representations encode complementary structural information, with neutral losses reflecting the scaffold and α-pyrone C6 substituent and fragment ions reflecting the C3 side chain; their combined use therefore offers a more complete basis for MS/MS-based annotation than either representation alone. Semi-quantitative analysis confirmed an arzanol-dominated profile with a total PG-α-pyrone content within the order of magnitude reported for the genus, reinforcing the standing of *H. italicum* as a rich source of these bioactive metabolites. Future work incorporating elemental-composition-constrained fragment matching and integrated fragment-ion/neutral-loss models may further improve the structural resolution of MS/MS-based profiling for this and related metabolite classes.

## Figures and Tables

**Table 1:** Diagnostic NLs for characterizing the C6 alkyl substituent of the α-pyrone unit.

C6 (α-pyr)	NL Mass α-pyr	Elem. Comp.	NL Mass α-pyr-CH_2_	Elem. Comp.
**R =**	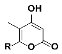		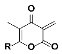	
**Methyl**	140.0473	C_7_H_8_O_3_	152.0473	C_8_H_8_O_3_
**Ethyl**	154.0630	C_8_H_10_O_3_	166.0630	C_9_H_10_O_3_
**Propyl**	168.0786	C_9_H_12_O_3_	180.0786	C_10_H_12_O_3_
**Butyl**	182.0943	C_10_H_14_O_3_	194.0943	C_11_H_14_O_3_
**Pentyl**	196.1099	C_11_H_16_O_3_	208.1099	C_12_H_16_O_3_
**Hexyl**	210.1256	C_12_H_18_O_3_	222.1256	C_13_H_18_O_3_

Values are calculated monoisotopic masses (Da) for the neutral loss and, in the paired columns, for the corresponding loss extended by a CH_2_ unit; the accompanying elemental composition is given for each.

**Table 2:** Diagnostic fragment ions for characterizing the C6 alkyl substituent of the α-pyrone unit.

C6 (α-pyr)	[α-pyr-H]-	Elem. Comp.	[α-pyr-CO_2_-H]-	Elem. Comp.
**R =**	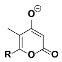		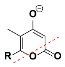	
**Methyl**	139.0401	C_7_H_7_O_3_^−^	95.0502	C_6_H_7_O^−^
**Ethyl**	153.0558	C_8_H_9_O_3_^−^	109.0659	C_7_H_9_O^−^
**Propyl**	167.0715	C_9_H_11_O_3_^−^	123.0816	C_8_H_11_O^−^
**Butyl**	181.0872	C_10_H_13_O_3_^−^	137.0973	C_9_H_13_O^−^
**Pentyl**	195.1029	C_11_H_15_O_3_^−^	151.1130	C_10_H_15_O^−^
**Hexyl**	209.1186	C_12_H_17_O_3_^−^	165.1287	C_11_H_17_O^−^

Values are calculated *m*/*z* for the [α-pyr-H]^−^ and [α-pyr-CO_2_-H]^−^ ions in negative-ion mode, with the elemental composition of each ion.

**Table 3:** Diagnostic fragment ions for the deprotonated C1 acyl substituents of the acylphloroglucinol moiety.

R 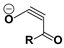	Fragment Ion *m*/*z*	Elem. Comp.
**Methyl**	83.01385	C_4_H_3_O_2_^−^
**Ethyl**	97.02895	C_5_H_5_O_2_^−^
**Propyl**	111.0441	C_6_H_7_O_2_^−^
**Butyl**	125.0592	C_7_H_9_O_2_^−^
**Pentyl**	139.0743	C_8_H_11_O_2_^−^
**Hexyl**	153.0894	C_9_H_13_O_2_^−^
**Phenylethyl**	173.0608	C_11_H_9_O_2_^−^

**Table 4:** Comparative MS/MS behavior of phloroglucinol α-pyrone derivatives annotated in *H. italicum*.

Subclass (*n*)	[M–H]^−^	Fragment A (PG–CH_2_)	Fragment B (PG)	[α-pyr-H]^−^	[α-pyr-CO_2_–H]^−^	Base Peak	Specific Ions
monopyrone (1)	40	n.a.	n.a.	n.a.	n.a.	[M–H–CO_2_]^−^	207.1390 (100)
dipyrones (2)	≤3	n.a.	n.a.	93–100	52–53	[α-pyr–H]^−^	—
3-NA PG-α-pyr (9)	24–64	80–95	100	6–12	6–12	Fragment B	137.0244 (10–17); 125.0244 (3–5); 163.0031 (2–5); 81.0346 (3–8)
3-prenyl PG-α-pyr (14)	55–100	70–94	86–100	10–16	8–20	Fragment B (11/14)	205.0870 (3–7); 193.0870 (3–5)
3-(hydroxyprenyl) (2)	65–86	72–77	100	6–7	10	Fragment B	191.0714 (2.5) ^a^
3-(hydroxydihydroprenyl) (2)	85–89	87–93	100	8–9	9–11	Fragment B	223.0976 (7–8) ^a^; 211.0976 (1–2)
3-geranyl (3)	94–100	63–88	80–100	10–14	11–12	[M–H]^−^ (2/3)	203.0714 (3–4) ^a^
3-(hydroxygeranyl) (3)	100	57–73	77–88	6–10	10–11	[M–H]^−^	—
benzofurans (8)	12–40	0–14	100	16–51	15–33	Fragment B	147.0815 (2–4) ^b^
benzopyrans (10)	19–83	0–5	57–100	70–100	50–72	Fragment B (9/10)	193.0506 (4–20); 149.0608 (2–10)
spiroketals (5)	24–65	0	100	absent	absent	Fragment B	243.0663 (1–4); 215.0350 (2–9) ^c^; 229.0506 (2–5) ^c^

n.a., not applicable (no phloroglucinol moiety); *n*, number of compounds in the subclass. ^a^ Isobaric with the Fragment A/B ions of the C6-propyl and C6-butyl 3-NA derivatives (cmpds 6, 9, 10, 15, 16), in which they reach 85–100%, distinguished by the neutral loss relating them to the precursor. ^b^ Detected also in benzofurans (cmpds 11, 12, 18, 19). ^c^ *m*/*z* 215.0350 was detected in all spiroketals except cmpd 13; *m*/*z* 229.0506 exceeded 1% relative intensity in cmpds 13 and 37 only.

**Table 5:** Clustering validation against manual structural annotations. For each annotation level, the best-performing clustering solution was selected separately for the fragment-ion (FI) and neutral-loss (NL) matrices on the basis of ARI. Bold ARI marks the better-agreeing representation at each level. *n* = 58 compounds.

Annotation	Structural Meaning	Fragment-Ion Matrix			Neutral-Loss Matrix		
Level		**Method**	**ARI**	**Purity**	**Method**	**ARI**	**Purity**
subclass	Structural subclass	Hier.	0.145	0.948	Hier.	0.424	0.948
C6_a_pyr	α-pyrone C6 substituent	PAM	0.043	0.807	PAM	0.264	0.966
C1_pg	Phloroglucinol C1 substituent	PAM	0.067	0.818	PAM	0.001	0.625
C3_pg	Phloroglucinol C3 substituent	PAM	0.201	0.809	PAM	0.025	0.562

Annotation levels (row labels): **subclass**, structural subclass of the compound (phloroglucinol α-pyrone, benzofuran, benzopyran, spiroketal, monopyrone, dipyrone); **C6_a_pyr**, alkyl substituent at C6 of the α-pyrone ring; **C1_pg**, acyl substituent at C1 of the phloroglucinol (acylphenone) moiety; **C3_pg**, side chain at C3 of the phloroglucinol core (unsubstituted, prenyl, hydroxyprenyl, hydroxydihydroprenyl, geranyl, hydroxygeranyl). Hier., hierarchical clustering; PAM, partitioning around medoids, with clustering settings as described in Section 3.13. ARI, adjusted Rand index—agreement between the clustering and the manual annotation, corrected for chance (1 = perfect agreement, 0 = no better than random); Purity, fraction of compounds belonging to the majority annotation class within their assigned cluster.

**Table 6:** Semi-quantitative distribution of the 59 phloroglucinol α-pyrone derivatives identified in the *Helichrysum italicum* extract. For each compound, the assigned numerical identifier (№), structural subclass, recovery-corrected content (% dry weight, expressed as 2′,4′,6′-trihydroxyacetophenone equivalents), relative contribution to the total PG-α-pyrone pool (%), and abundance rank (by decreasing content) are given. Trivial names are listed for the 23 compounds corresponding to previously reported structures; the remaining 36 entries represent derivatives not previously described from the genus.

№	* DW (%)	Subclass	Contribution (%)	Abundance Rank	Trivial
**1**	5.9 × 10^−5^	monopyr	0.31	33	micropyrone
**2**	1.3 × 10^−5^	dipyr	0.07	53	helipyrone B
**3**	7.4 × 10^−5^	phlor_pyr	0.39	30	
**4**	5.6 × 10^−5^	dipyr	0.29	35	helipyrone A
**5**	2.3 × 10^−4^	phlor_pyr	1.19	13	
**6**	4.9 × 10^−5^	phlor_pyr	0.26	37	
**7**	2.4 × 10^−5^	phlor_pyr	0.12	41	
**8**	6.8 × 10^−4^	spiro	3.55	6	helispiroketal F
**9**	5.8 × 10^−5^	phlor_pyr	0.31	34	
**10**	1.4 × 10^−4^	phlor_pyr	0.74	19	
**11**	6.6 × 10^−5^	benzofuran	0.34	32	
**12**	7.8 × 10^−5^	benzofuran	0.40	28	
**13**	1.5 × 10^−5^	spiro	0.08	47	
**14**	3.1 × 10^−3^	phlor_pyr	16.02	2	
**15**	1.0 × 10^−4^	phlor_pyr	0.52	24	
**16**	4.0 × 10^−5^	phlor_pyr	0.21	38	
**17**	2.5 × 10^−5^	phlor_pyr	0.13	39	
**18**	1.1 × 10^−4^	benzofuran	0.55	23	italipyrone
**19**	1.4 × 10^−4^	benzofuran	0.74	20	italipyrone
**20**	5.4 × 10^−3^	phlor_pyr	28.29	1	arzanol
**21**	1.5 × 10^−4^	phlor_pyr	0.80	18	
**22**	8.0 × 10^−5^	benzopyran	0.42	27	
**23**	5.3 × 10^−5^	benzopyran	0.28	36	
**24**	9.9 × 10^−5^	phlor_pyr	0.52	25	
**25**	1.2 × 10^−5^	phlor_pyr	0.06	54	
**26**	8.0 × 10^−5^	phlor_pyr	0.42	26	helitalone B
**27**	4.8 × 10^−4^	phlor_pyr	2.48	10	
**28**	1.9 × 10^−4^	phlor_pyr	0.98	16	arenol B
**29**	2.2 × 10^−4^	phlor_pyr	1.14	14	
**30**	1.6 × 10^−4^	benzopyran	0.82	17	plicatipyrone
**31**	1.1 × 10^−4^	benzopyran	0.57	22	plicatipyrone
**32**	2.5 × 10^−5^	phlor_pyr	0.13	40	
**33**	4.4 × 10^−6^	benzofuran	0.02	59	
**34**	1.4 × 10^−5^	benzofuran	0.07	49	
**35**	6.3 × 10^−4^	phlor_pyr	3.29	8	arenol C
**36**	4.9 × 10^−4^	phlor_pyr	2.56	9	6-O-desmethylauricepyrone/3-prenyl norauricepyrone
**37**	7.3 × 10^−5^	spiro	0.38	31	
**38**	1.1 × 10^−5^	benzofuran	0.06	55	22-methyl-22-ethyl-italipyrone
**39**	2.1 × 10^−5^	benzofuran	0.11	43	22-methyl-22-ethyl-italipyrone
**40**	7.4 × 10^−4^	phlor_pyr	3.86	5	23-methyl-6-O-desmethylauricepyrone
**41**	1.9 × 10^−4^	phlor_pyr	0.98	15	
**42**	1.3 × 10^−3^	phlor_pyr	6.99	4	18,18-bis-desmethyl achyroclinopyrone C
**43**	3.9 × 10^−4^	phlor_pyr	2.02	12	23-ethyl-6-O-desmethyl-auricepyrone
**44**	1.2 × 10^−4^	phlor_pyr	0.62	21	23-ethyl-6-O-desmethyl-auricepyrone
**45**	1.3 × 10^−5^	benzopyran	0.07	51	
**46**	1.4 × 10^−5^	benzopyran	0.07	50	
**47**	1.8 × 10^−3^	phlor_pyr	9.27	3	18,18-bis-desmethyl achyroclinopyrone A
**48**	1.0 × 10^−5^	phlor_pyr	0.05	56	
**49**	1.3 × 10^−5^	benzopyran	0.07	52	
**50**	1.4 × 10^−5^	phlor_pyr	0.08	48	8′-methyl-18,18-bis-desmethyl achyroclinopyrone A
**51**	2.3 × 10^−5^	benzopyran	0.12	42	
**52**	9.3 × 10^−6^	benzopyran	0.05	57	
**53**	1.9 × 10^−5^	phlor_pyr	0.10	44	
**54**	1.5 × 10^−5^	phlor_pyr	0.08	46	
**55**	1.7 × 10^−5^	phlor_pyr	0.09	45	
**56**	4.6 × 10^−6^	benzopyran	0.02	58	
**59**	4.0 × 10^−4^	spiro	2.11	11	helispiroketal A
**60**	7.6 × 10^−5^	spiro	0.40	29	helispiroketal A
**61**	6.4 × 10^−4^	phlor_pyr	3.34	7	

Subclass abbreviations: monopyr, monopyrone; dipyr, dipyrone; phlor_pyr, phloroglucinol α-pyrone; benzofuran, benzofuran derivative; benzopyran, benzopyran derivative; spiro, spiroketal derivative. * Values are LC–HRMS response-based estimates obtained against a single external standard and are therefore semi-quantitative.

## Data Availability

The original contributions presented in this study are included in the article/Supplementary Materials. Further inquiries can be directed to the corresponding author.

## Supplementary Materials

The following supporting information can be downloaded at: https://www.mdpi.com/article/10.3390/plants15172619/s1.

## Abbreviations

The following abbreviations are used in this manuscript:

**Abbreviation**	**Definition**
ARI	adjusted Rand index
CID/HCD	collision-induced dissociation/higher-energy collisional dissociation
DDA	data-dependent acquisition
DW	dry weight
EIC	extracted-ion chromatogram
ESI	electrospray ionization
FI	fragment ion
HRMS	high-resolution mass spectrometry
LC–HRMS	liquid chromatography–high-resolution mass spectrometry
MS/MS (MS^2^)	tandem mass spectrometry
NL	neutral loss
PAM	partitioning around medoids
PG	phloroglucinol (acylphloroglucinol) moiety
PG-α-pyr	phloroglucinol α-pyrone
TIC	total ion chromatogram
UHPLC	ultra-high-performance liquid chromatography
UPGMA	unweighted pair-group method with arithmetic mean (average linkage)
Fragment A/Fragment B	acylphloroglucinol-derived fragment ions with and without retention of the methylene bridge, respectively

## Appendix A

**Table A1:** Exact-mass and tandem mass spectrometric data for the phloroglucinol α-pyrone derivatives detected in the *Helichrysum italicum* extract by UHPLC–Orbitrap–MS in negative ionization mode. For each compound, the assigned identifier, elemental composition of the deprotonated molecule, measured *m*/*z* of the [M–H]^−^ ion, and retention time are provided; MS^2^ fragment ions are given as *m*/*z* with relative intensities (% base peak) in parentheses.

Compound	Elemental Composition	[M-H]^−^	MS^2^	t_R_ (Min)
**1**	C_14_H_19_O_4_^−^	251.1289	251.1289 (39.5), 207.139 (100), 179.1077 (2.3), 167.0714 (4.1), 151.1128 (31.3), 123.0815 (15), 113.0972 (94.4), 85.0659 (28.4), 55.0189 (2.6)	2.93
**2**	C_16_H_17_O_6_^−^	305.1031	247.0976 (1.3), 221.0819 (1.6), 153.0557 (93.3), 139.0401 (100), 109.0659 (51.8), 95.0502 (49.7)	8.74
**3**	C_16_H_15_O_7_^−^	319.0823	319.0823 (24.2), 179.035 (95), 167.035 (100), 163.0037 (3.9), 151.0401 (1.3), 139.0401 (11.9), 137.0244 (10.7), 125.0244 (3.1), 123.0451 (23), 109.0295 (1.4), 95.0502 (10.3), 83.0138 (3.1), 81.0346 (4.7)	5.12
**4**	C_17_H_19_O_6_^−^	319.1187	319.1187 (2.6), 153.0557 (100), 109.0659 (53)	11.25
**5**	C_17_H_17_O_7_^−^	333.0980	333.098 (27.1), 179.035 (90.8), 167.035 (100), 163.0037 (5.2), 153.0557 (8.8), 151.0401 (1.3), 151.0037 (1.6), 137.0244 (12.9), 125.0244 (2.7), 123.0451 (28.2), 109.0659 (10.1), 109.0295 (2.1), 83.0138 (3.9), 81.0346 (4)	6.77
**6**	C_19_H_21_O_7_^−^	361.1293	361.1293 (38.2), 221.0819 (89.1), 209.0819 (100), 203.0714 (2.9), 165.0921 (18.9), 163.0037 (2.2), 139.0401 (8.2), 137.0244 (10.2), 125.0608 (4.3), 125.0244 (2.6), 123.0815 (1.3), 109.0295 (2.4), 95.0502 (5.9), 81.0346 (3.4)	10.93
**7**	C_19_H_21_O_7_^−^	361.1293	361.1293 (49.8), 207.0663 (79.6), 207.051 (1.8), 195.0663 (100), 189.0557 (3.9), 179.0714 (1.3), 179.035 (1.1), 177.0557 (1.3), 165.0921 (4.6), 163.0037 (3.3), 153.0557 (8.5), 151.0764 (23.6), 139.0401 (1.1), 137.0244 (13.8), 125.0244 (3.6), 111.0451 (3.8), 109.0659 (11.5), 109.0295 (1.5), 95.0502 (2.6), 83.0138 (1.1), 81.0346 (5)	11.35
**8**	C_21_H_23_O_6_^−^	371.1500	371.15 (28.6), 303.1602 (4.7), 259.0976 (100), 243.0663 (1.8), 236.0326 (1.4), 217.087 (7), 216.0428 (1.3), 215.035 (5.9), 204.0428 (3.8), 203.035 (1.1), 162.0322 (1.4), 161.0244 (1.9)	11.24
**9**	C_20_H_23_O_7_^−^	375.1449	375.1449 (50.6), 235.0976 (92.6), 223.0976 (100), 221.0819 (2.5), 217.087 (2.8), 209.0819 (3), 179.1077 (21.9), 179.0925 (1.1), 174.0322 (1.8), 163.0037 (3), 139.0764 (5.5), 139.0401 (7.2), 137.0972 (2.6), 137.0244 (14.2), 125.0244 (3.5), 122.0373 (1.1), 109.0295 (2.1), 95.0502 (6.2), 83.0138 (1.2), 81.0346 (5.5)	13.60
**10**	C_20_H_23_O_7_^−^	375.1449	375.1449 (63.9), 221.0819 (85.4), 209.0819 (100), 203.0714 (2.8), 179.1077 (9.2), 165.0921 (22), 163.0037 (4.2), 153.0557 (6.7), 139.0401 (3), 137.0244 (15), 125.0608 (5.5), 125.0244 (4), 123.0815 (2.5), 109.0659 (6.3), 109.0295 (2.7), 95.0502 (3.5), 83.0138 (1.1), 81.0346 (7.9)	13.14
**11**	C_21_H_21_O_7_^−^	385.1293	385.1293 (11.8), 245.0819 (2.8), 233.0819 (100), 217.0506 (1.8), 191.0714 (1.7), 189.0921 (3.6), 187.0764 (1.3), 149.0608 (1.2), 147.0815 (2.4), 145.0659 (1), 139.0401 (51.2), 107.0502 (1.8), 95.0502 (30.7), 83.0138 (4.1), 81.0346 (2.1), 79.0553 (1.3)	13.90
**12**	C_21_H_21_O_7_^−^	385.1293	385.1293 (15.3), 245.0819 (13.6), 233.0819 (100), 217.0506 (1.1), 191.0714 (2.8), 189.0921 (4), 187.0764 (1.1), 173.0608 (1.1), 149.0608 (1.5), 147.0815 (3.6), 145.0659 (1.3), 139.0401 (19.1), 107.0502 (2.3), 95.0502 (16.2), 83.0138 (5.1), 81.0346 (1.6), 79.0553 (1.4)	17.00
**13**	C_22_H_25_O_6_^−^	385.1657	385.1657 (42.4), 287.1289 (14.9), 273.1496 (1.7), 273.1132 (100), 243.0663 (1.1), 229.0506 (5.2), 218.0584 (1.5), 217.087 (7.3), 217.0506 (1.3), 162.0322 (1.2), 161.0244 (1.1)	13.61
**14**	C_21_H_23_O_7_^−^	387.1449	387.1449 (55.3), 247.0976 (93.7), 235.0976 (100), 205.087 (4.3), 193.087 (3.8), 191.1077 (8.1), 166.0271 (2.8), 150.0322 (2.5), 139.0401 (13.4), 95.0502 (13.2), 83.0138 (5.3)	8.46
**15**	C_21_H_25_O_7_^−^	389.1606	389.1606 (53), 235.0976 (84), 223.0976 (100), 217.087 (2), 179.1077 (21.6), 163.0037 (2.8), 153.0557 (6.6), 139.0764 (5.7), 137.0972 (2.7), 137.0244 (11.5), 125.0244 (3.1), 109.0659 (8.2), 109.0295 (1.6), 107.0138 (1.5), 81.0346 (5.4)	15.37
**16**	C_21_H_25_O_7_^−^	389.1606	389.1606 (50.5), 235.0976 (84.3), 223.0976 (100), 217.087 (1.9), 207.1027 (2.3), 179.1077 (24.4), 163.0037 (2.8), 153.0557 (7.6), 139.0764 (6.1), 137.0972 (3.7), 137.0244 (17.3), 125.0244 (4.6), 109.0659 (8.1), 109.0295 (2.4), 83.0138 (2.4), 81.0346 (7)	15.86
**17**	C_21_H_25_O_7_^−^	389.1606	389.1606 (59.6), 249.1344 (1.5), 249.1132 (85.9), 237.1344 (2), 237.1132 (100), 237.0768 (1.3), 235.0976 (4.1), 223.0976 (4.6), 221.0819 (7.2), 209.0819 (8.9), 193.1234 (20.7), 174.0322 (1.1), 165.0921 (1.1), 163.0037 (4.5), 153.0921 (7.9), 151.1128 (1.9), 139.0401 (6.3), 137.0244 (17), 125.0244 (4), 109.0295 (2.2), 95.0502 (6.7), 81.0346 (7.5)	15.10
**18**	C_22_H_23_O_7_^−^	399.1449	399.1449 (12.9), 245.0819 (2.8), 233.0819 (100), 217.0506 (1.1), 191.0714 (2.1), 189.0921 (4.8), 187.0764 (1.5), 153.0557 (46.4), 149.0608 (1.4), 147.0815 (3.6), 145.0659 (1.7), 109.0659 (32.8), 107.0502 (1.9), 105.071 (1.1), 83.0138 (4), 81.0346 (2.3)	16.43
**19**	C_22_H_23_O_7_^−^	399.1449	399.1449 (18.7), 245.0819 (13), 233.0819 (100), 217.0506 (1.4), 191.0714 (3.2), 189.0921 (3.3), 187.0764 (1.4), 173.0608 (1.3), 153.0557 (16.1), 149.0608 (1), 147.0815 (3.8), 145.0659 (1.6), 109.0659 (15.4), 107.0502 (2.6), 105.071 (1.3), 83.0138 (6.2), 81.0346 (1.4), 79.0553 (1.3)	19.64
**20**	C_22_H_25_O_7_^−^	401.1606	401.1606 (56.5), 247.0976 (87.6), 235.0976 (100), 205.087 (5.4), 203.1077 (1.6), 193.087 (4.2), 192.0428 (2.2), 191.1077 (12.2), 191.035 (2), 166.0271 (5.8), 164.0479 (2.4), 161.0972 (1.9), 153.0557 (16.1), 150.0322 (3.8), 149.0972 (3.4), 123.0451 (1.6), 109.0659 (17.1), 83.0138 (6.9), 81.0346 (3.2)	10.37
**21**	C_22_H_25_O_7_^−^	401.1606	401.1606 (68.3), 261.1132 (89.5), 249.1132 (100), 247.0976 (2.5), 235.0976 (4.4), 206.0584 (1), 205.1234 (8), 205.087 (5.4), 193.087 (3.6), 191.035 (1.1), 180.0428 (2.7), 149.0972 (2.6), 139.0401 (11.9), 136.0166 (1.1), 109.0659 (1.1), 97.0295 (7.8), 95.0502 (18.3)	11.23
**22**	C_21_H_23_O_8_^−^	403.1398	403.1398 (34.7), 263.0925 (2.9), 251.0925 (100), 193.0506 (18.8), 179.035 (2.2), 177.0193 (1.1), 151.0401 (4.5), 149.0608 (5), 139.0401 (85.1), 107.0502 (2), 95.0502 (50.8), 83.0138 (2.7)	4.85
**23**	C_21_H_23_O_8_^−^	403.1398	403.1398 (22.3), 263.0925 (2.8), 251.0925 (100), 233.0819 (2), 193.0506 (4.6), 191.035 (1.3), 179.035 (21.5), 149.0608 (1.6), 139.0401 (90.8), 137.0244 (3.7), 95.0502 (49.6), 83.0138 (1.3)	6.90
**24**	C_21_H_23_O_8_^−^	403.1398	403.1398 (65.4), 263.0925 (77.3), 251.0925 (100), 233.0819 (13.7), 193.0506 (5.2), 192.0428 (2.7), 189.0921 (2.6), 181.0506 (3.1), 180.0428 (2.3), 179.035 (4.8), 164.0479 (15.8), 163.0401 (1.7), 151.0401 (5.5), 139.0401 (7.3), 137.0608 (6.2), 125.0608 (1), 123.0451 (1.6), 99.0451 (3.5), 95.0502 (9.5), 83.0138 (7.5), 81.0346 (7.3)	9.97
**25**	C_21_H_25_O_8_^−^	405.1555	405.1555 (85), 265.1293 (1.7), 265.1081 (92.8), 253.1081 (100), 249.0768 (1.6), 223.0976 (8.1), 211.0976 (1.2), 209.1183 (8.9), 207.0663 (1.4), 199.0401 (4.1), 191.035 (1.7), 151.0764 (1.9), 139.0401 (8.1), 133.0659 (1.4), 123.0451 (4.5), 121.0659 (1.3), 95.0502 (8.8), 83.0138 (7.1), 81.0346 (12)	5.44
**26**	C_23_H_27_O_7_^−^	415.1762	415.1762 (75.7), 259.0976 (2.3), 247.0976 (73.6), 235.0976 (100), 231.0663 (1.7), 219.0663 (1.1), 205.087 (4.4), 193.087 (4.5), 192.0428 (2.1), 191.1077 (10.8), 191.035 (1.8), 180.0428 (1.3), 179.035 (1.4), 177.0921 (1.5), 167.0714 (12.5), 166.0271 (5.1), 164.0479 (1.7), 161.0972 (2), 151.0764 (1.2), 150.0322 (3.1), 149.0972 (3.6), 133.0659 (1.3), 123.0815 (15.4), 123.0451 (1.7), 109.0659 (2.3), 95.0502 (1.2), 83.0138 (7.1), 81.0346 (3.2), 57.0346 (1)	12.45
**27**	C_23_H_27_O_7_^−^	415.1762	415.1762 (82.4), 275.1289 (1), 263.1289 (1.1), 261.1132 (77.3), 249.1132 (100), 233.1183 (1), 206.0584 (1), 205.1234 (9.9), 205.087 (5.3), 205.0506 (2.2), 193.087 (3.6), 191.035 (1.2), 180.0428 (2.9), 161.0972 (2.5), 153.0557 (11.9), 152.0115 (2.5), 149.0972 (1.3), 136.0166 (1.3), 109.0659 (16.4), 97.0295 (6.1), 95.0502 (2.9)	13.45
**28**	C_23_H_27_O_7_^−^	415.1762	415.1762 (85.8), 275.1289 (86.4), 263.1289 (100), 257.1183 (1.6), 247.134 (2.3), 219.139 (6.2), 205.087 (3.1), 205.0506 (3.2), 194.0584 (2), 193.087 (2.8), 191.035 (1.6), 177.0921 (1.6), 152.0115 (2.5), 151.0764 (2.9), 149.0972 (2.1), 139.0401 (13.3), 111.0451 (8.9), 109.0659 (5.3), 95.0502 (12.7)	13.13
**29**	C_22_H_25_O_8_^−^	417.1555	417.1555 (86.3), 263.0925 (72.5), 251.0925 (100), 233.0819 (16.7), 193.0506 (3.1), 192.0428 (3.8), 191.0714 (2.5), 189.0921 (2.5), 181.0506 (2.5), 180.0428 (3), 179.035 (4.7), 177.0193 (2), 165.0557 (1.8), 164.0479 (17.4), 153.0557 (5.9), 151.0401 (6.2), 149.0244 (1.1), 147.0815 (2.2), 139.0401 (1.2), 137.0608 (5.3), 125.0608 (2), 123.0451 (1), 109.0659 (10.4), 107.0502 (1.1), 99.0451 (4.1), 95.0502 (3), 83.0138 (8.5), 81.0346 (9.2)	12.42
**30**	C_22_H_25_O_8_^−^	417.1555	417.1555 (39.1), 263.0925 (3.5), 251.0925 (100), 233.0819 (1.8), 193.0506 (19.8), 179.035 (2.6), 177.0193 (1.7), 153.0557 (71.7), 151.0401 (4.4), 149.0608 (6.7), 109.0659 (54.3), 109.0295 (1.2), 107.0502 (2.9), 83.0138 (3.5)	6.30
**31**	C_22_H_25_O_8_^−^	417.1555	417.1555 (19.2), 263.0925 (2.7), 251.0925 (100), 251.0714 (2), 233.0819 (1.2), 193.0506 (6.6), 179.035 (27.6), 163.0037 (2.2), 153.0557 (77.7), 137.0244 (4.9), 109.0659 (50.1), 83.0138 (1.2)	8.87
**32**	C_22_H_27_O_8_^−^	419.1711	419.1711 (89.2), 265.1081 (86.7), 253.1081 (100), 223.0976 (6.5), 211.0976 (2.1), 209.1183 (8.4), 195.1027 (1.8), 165.0557 (1.1), 153.0557 (8.6), 151.0764 (3.7), 143.1077 (1), 133.0659 (3.4), 123.0451 (3.2), 121.0659 (3.3), 109.0659 (10.9), 83.0138 (8.2), 81.0346 (14.4), 57.0346 (2.1)	7.22
**33**	C_24_H_27_O_7_^−^	427.1762	427.1762 (32.1), 275.1289 (100), 205.0506 (2.5), 139.0401 (28.4), 95.0502 (22.7)	16.76
**34**	C_24_H_27_O_7_^−^	427.1762	427.1762 (25), 287.1289 (1.4), 275.1289 (100), 252.0639 (1.1), 219.0663 (3.6), 205.0506 (4.6), 176.0115 (1.4), 164.0115 (1.1), 139.0401 (27.4), 133.0659 (1.5), 95.0502 (28.9)	17.86
**35**	C_24_H_29_O_7_^−^	429.1919	429.1919 (75), 289.1809 (1.2), 289.1445 (82.6), 277.1445 (100), 271.134 (1.1), 261.1496 (2.3), 233.1547 (7.4), 219.0663 (3.9), 205.087 (3.2), 205.0506 (4.7), 193.087 (4), 166.0271 (1), 165.0921 (1.2), 161.0972 (1.1), 152.0115 (5.7), 149.0972 (2.6), 139.0401 (12.1), 136.0166 (1.1), 125.0608 (11.7), 124.0166 (1.4), 123.0815 (2.6), 109.0659 (2.5), 95.0502 (13.9)	15.00
**36**	C_24_H_29_O_7_^−^	429.1919	429.1919 (97.8), 289.1445 (3.9), 277.1445 (4.5), 275.1289 (79.5), 263.1289 (100), 261.1132 (2.8), 249.1132 (3.6), 247.134 (1), 219.139 (8.1), 219.0663 (1.1), 205.087 (4.2), 205.0506 (3.9), 194.0584 (2.5), 193.087 (3.4), 191.035 (2.3), 177.0921 (1.2), 161.0972 (1.1), 153.0557 (12.5), 152.0115 (3.5), 151.0764 (3.1), 149.0972 (2.8), 123.0815 (2.6), 111.0451 (7.3), 109.0659 (19.9)	15.40
**37**	C_26_H_31_O_6_^−^	439.2126	439.2126 (65.3), 327.1602 (100), 243.0663 (4.4), 229.0506 (1.9), 215.035 (8.8), 203.035 (30.1), 190.0271 (1.2)	18.35
**38**	C_25_H_29_O_7_^−^	441.1919	441.1919 (39.7), 289.1445 (6.3), 275.1289 (100), 275.1077 (1.6), 252.0639 (2), 205.0506 (5), 176.0115 (1.3), 153.0557 (23.4), 139.0401 (1.6), 133.0659 (1.3), 109.0659 (24.9), 95.0502 (1.5), 69.0346 (1.5)	18.98
**39**	C_25_H_29_O_7_^−^	441.1919	441.1919 (32.5), 275.1289 (100), 275.1077 (1.9), 252.0639 (2.6), 231.0663 (1.1), 219.0663 (3.8), 205.0506 (5.4), 153.0557 (20), 153.0405 (1.2), 133.0659 (3.2), 109.0659 (28)	18.98
**40**	C_25_H_31_O_7_^−^	443.2075	443.2075 (83.3), 289.1809 (1.3), 289.1445 (78.1), 277.1445 (100), 271.134 (1.2), 233.1547 (6.9), 219.0663 (2.5), 208.0741 (1.1), 205.087 (5.1), 205.0506 (5.5), 193.087 (3.1), 161.0972 (1), 153.0557 (12.9), 152.0115 (5.4), 149.0972 (1.4), 139.0401 (1), 137.0972 (1.3), 136.0166 (1.4), 125.0608 (7.2), 123.0815 (2.6), 109.0659 (16.7), 95.0502 (1.4)	17.26
**41**	C_25_H_31_O_7_^−^	443.2075	443.2075 (82.2), 303.1602 (80.5), 291.1602 (100), 291.1238 (1.8), 247.1703 (5.8), 233.0819 (1.2), 205.087 (4.7), 193.087 (4.1), 191.035 (4.4), 161.0972 (1.2), 152.0115 (3), 149.0972 (1.6), 139.0764 (10.1), 139.0401 (12.6), 137.0972 (3.4), 136.0166 (2.7), 124.0166 (1.4), 95.0502 (13.2)	17.95
**42**	C_26_H_31_O_7_^−^	455.2075	455.2075 (93.6), 315.1602 (87.7), 303.1602 (100), 273.1496 (1.3), 259.1703 (4.9), 231.0663 (3), 218.0948 (1.2), 203.0714 (3.5), 192.0428 (1), 191.035 (6), 179.035 (9.7), 167.035 (3.4), 166.0271 (8.4), 164.0479 (2.4), 150.0322 (4), 139.0401 (14.3), 138.0322 (1.2), 137.0244 (1.1), 95.0502 (10.6), 83.0138 (4.7)	16.20
**43**	C_26_H_33_O_7_^−^	457.2232	457.2232 (100), 303.1602 (72.9), 291.1602 (93), 247.1703 (7.2), 219.0663 (2), 205.087 (6.7), 205.0506 (4.8), 193.087 (4.9), 191.035 (6.1), 187.0764 (1.8), 179.035 (2.3), 177.0921 (1.7), 166.0271 (1.9), 161.0972 (3.1), 153.0557 (9.7), 152.0115 (2), 151.1128 (1.1), 151.0764 (1.8), 149.0972 (3.4), 139.0764 (7.5), 137.0972 (1.9), 136.0166 (1.8), 124.0166 (1.1), 109.0659 (15)	19.71
**44**	C_26_H_33_O_7_^−^	457.2232	457.2232 (100), 303.1602 (69.9), 291.1602 (86.2), 247.1703 (6.8), 233.0819 (1), 222.0897 (1), 205.087 (4.9), 193.087 (4.3), 191.035 (3.2), 161.0972 (2), 153.0557 (9.9), 152.0115 (4), 149.0972 (2.1), 139.0764 (7.2), 137.0972 (1.8), 124.0166 (1.7), 109.0659 (15.3)	20.15
**45**	C_25_H_31_O_8_^−^	459.2024	459.2024 (65.5), 307.1551 (22.6), 305.1394 (3.2), 293.1394 (100), 293.1031 (2.4), 249.1132 (2.1), 235.0976 (15.2), 191.1077 (6.5), 153.0557 (69.6), 151.0401 (1.8), 139.0764 (1.9), 139.0401 (13.1), 125.0608 (2.8), 109.0659 (54.2), 95.0502 (10.7), 51.024 (2.7)	12.37
**46**	C_27_H_31_O_7_^−^	467.2075	467.2075 (83.1), 331.1915 (1.4), 301.1445 (57.1), 153.071 (2.5), 153.0557 (100), 109.0659 (59), 83.0138 (1.6)	15.18
**47**	C_27_H_33_O_7_-	469.2232	469.2232 (100), 315.1966 (1.4), 315.1602 (77.9), 315.1238 (1.2), 303.1602 (100), 261.1496 (1.4), 259.1703 (5.5), 231.0663 (3.1), 203.0714 (2.6), 191.035 (6.2), 189.0921 (1.1), 179.035 (11.5), 175.0764 (1.2), 167.035 (3), 166.0271 (12.8), 164.0479 (1.1), 153.0557 (10.8), 150.0322 (4.2), 138.0322 (1.2), 137.0244 (2.4), 109.0659 (12), 83.0138 (8), 81.0346 (1.5)	18.34
**48**	C_26_H_31_O_8_^−^	471.2024	471.2024 (100), 331.1551 (73.4), 319.1551 (80.2), 319.1187 (1.7), 277.1445 (1.2), 205.0506 (8.1), 192.0428 (1.4), 191.035 (1.4), 179.035 (1.5), 166.0271 (6.7), 163.0401 (1.5), 150.0322 (5.9), 139.0401 (9.7), 138.0322 (1.1), 123.0451 (2), 95.0502 (10.5), 83.0138 (5.4), 81.0346 (2.1), 57.0346 (1.1)	7.71
**49**	C_26_H_31_O_8_^−^	471.2024	471.2024 (64.9), 403.2126 (2.7), 331.1551 (2.7), 319.1551 (100), 193.0506 (16.4), 179.035 (2.3), 151.0401 (6.4), 149.0608 (8.4), 139.0401 (79.2), 109.0295 (1.6), 107.0502 (2.5), 95.0502 (59.4), 83.0138 (2.4)	11.56
**50**	C_28_H_35_O_7_^−^	483.2388	483.2388 (100), 315.1602 (62.7), 303.1602 (80.1), 259.1703 (2.5), 246.0897 (2.1), 191.035 (4.4), 179.035 (8.5), 167.0714 (10.3), 166.0271 (3.7), 150.0322 (2.8), 123.0815 (10.9), 83.0138 (3.4), 81.0346 (2.2)	20.40
**51**	C_27_H_33_O_8_^−^	485.2181	485.2181 (64.3), 331.1551 (5.4), 319.1551 (100), 319.1187 (2.3), 193.0506 (20.3), 179.035 (4.6), 177.0193 (1.9), 153.071 (2), 153.0557 (82.6), 151.0401 (6.2), 149.0608 (10.5), 109.0659 (71.8), 109.0295 (1.6), 107.0502 (1.6), 83.0138 (2)	13.72
**52**	C_27_H_33_O_8_^−^	485.2181	485.2181 (48.4), 331.1551 (4), 319.1551 (100), 319.1187 (2.8), 193.1023 (2), 193.0506 (3.8), 191.035 (3.5), 179.035 (29.9), 163.0037 (2.4), 153.0557 (73.7), 137.0244 (4.7), 109.0659 (53.9)	16.38
**53**	C_27_H_33_O_8_^−^	485.2181	485.2181 (100), 331.1551 (61.2), 328.0741 (1), 319.1551 (76.8), 319.1187 (2), 275.1653 (1), 205.0506 (6.6), 192.0428 (1.6), 191.035 (2.2), 166.0271 (6.3), 164.0479 (1.2), 153.0557 (5.5), 150.0322 (4.7), 109.0659 (10.4), 83.0138 (4.5), 81.0346 (1.1)	9.50
**54**	C_27_H_33_O_8_^−^	485.2181	485.2181 (100), 331.1551 (57), 319.1551 (88.5), 319.1187 (1.9), 301.1445 (8.2), 232.0741 (1.6), 219.0663 (1.6), 217.0506 (1.6), 193.0506 (6.9), 192.0428 (1.9), 191.035 (2.5), 181.0506 (1.6), 180.0428 (1.4), 179.035 (10.7), 164.0479 (12.9), 153.0557 (8.9), 151.0401 (1.8), 139.0401 (1.9), 137.0608 (2.8), 137.0244 (1.6), 109.0659 (10.9), 99.0451 (2), 95.0502 (1.5), 83.0138 (5.6), 81.0346 (9.3)	20.59
**55**	C_29_H_31_O_7_^−^	491.2075	491.2075 (90.4), 337.1445 (69.7), 325.1445 (100), 325.1023 (1.1), 281.1547 (2.2), 205.087 (3.5), 193.087 (3.8), 178.0271 (1.3), 173.0608 (6.6), 167.0138 (1), 161.0972 (1.3), 153.0557 (10.2), 152.0115 (4.1), 150.0322 (1.6), 149.0972 (2.2), 136.0166 (5.6), 133.0659 (1), 124.0166 (1.6), 109.0659 (7.5)	18.46
**56**	C_26_H_31_O_8_^−^	471.2024	471.2024 (34.1), 331.1551 (3.1), 319.1551 (100), 319.1187 (2.4), 193.0506 (6.5), 179.035 (20.8), 139.0401 (78.3), 137.0244 (5.8), 95.0502 (49.8)	14.30
**59**	C_20_H_21_O_6_^−^	357.1344	357.1344 (23.6), 259.0976 (100), 243.0663 (1.5), 236.0326 (1), 217.087 (5.3), 215.035 (5.7), 204.0428 (2.5), 203.035 (1.3), 162.0322 (1.9), 161.0244 (1.5)	9.21
**60**	C_20_H_21_O_6_^−^	357.1344	357.1344 (26.2), 259.0976 (100), 244.0741 (1.9), 243.0663 (4.3), 217.087 (3.1), 216.0428 (1.4), 215.035 (1.8), 204.0428 (2.1), 161.0244 (1.2)	9.57
**61**	C_25_H_31_O_7_^−^	443.2075	443.2075 (100), 303.1602 (73.2), 291.1602 (93.5), 275.1289 (2.4), 263.1289 (2.3), 247.1703 (6.2), 205.087 (4.3), 205.0506 (2.3), 193.087 (3.3), 191.035 (4.2), 152.0115 (2.6), 149.0972 (2.5), 139.0764 (5.8), 139.0401 (9.9), 137.0972 (2.9), 136.0166 (2.1), 95.0502 (10)	17.45

**Table A2:** Comparative assessment of semi-quantitatively determined PG-α-pyrone levels in *Helichrysum italicum* extract against previously reported isolation yields from *Helichrysum* species. Values from the present study are reported as recovery-corrected % dry weight (% DW) and expressed as 2′,4′,6′-trihydroxyacetophenone equivalents.

№	Compound/Assignment	Subclass	This Study, %DW	Total PG-α-Pyrones (%) Contribution	Literature Yield (% *w*/*w*)	Species/Material	Citation	Fold-Symbol	Comparison
**1**	micropyrone	monopyr	0.0000595	0.31	0.0054	*H. italicum* ssp. *microphyllum*, aerial parts with flowers	[33]	↓	Lower than the reported *H. italicum* ssp. *microphyllum* yield.
**1**	micropyrone	monopyr	0.0000595	0.31	0.013	*H. italicum* ssp. *microphyllum*, aerial parts with flowers	[11]	↓↓	About two orders of magnitude lower than this report.
**1**	micropyrone	monopyr	0.0000595	0.31	0.0482	*H. italicum* ssp. *microphyllum*, aerial parts with flowers	[10]	↓↓	About two to three orders of magnitude lower than this report.
**1**	micropyrone	monopyr	0.0000595	0.31	0.0015	*H. italicum* (Roth) G. Don, aerial parts with flowers	[12]	↓	Lower than this *H. italicum* report.
**2**	helipyrone B/norhelipyrone	dipyr	0.0000128	0.07	0.00001	*H. oocephalum*, dried aerial parts	[5]	≈	Same order of magnitude as the very low *H. oocephalum* yield.
**2**	helipyrone B/norhelipyrone	dipyr	0.0000128	0.07	not reported	*H. stoechas*	[16,17]	n/a	Reported qualitatively or without yield.
**2**	helipyrone B/norhelipyrone	dipyr	0.0000128	0.07	not reported	*H. arenarium*	[6]	n/a	Reported without quantitative yield.
**4**	helipyrone A	dipyr	0.0000558	0.29	0.041	*H. stoechas*, flowers	[14]	↓↓	Much lower than the high *H. stoechas* flower yield.
**4**	helipyrone A	dipyr	0.0000558	0.29	0.0256	*H. italicum* ssp. *microphyllum*, aerial parts with flowers	[11]	↓↓	Much lower than this *H. italicum* ssp. *microphyllum* report.
**4**	helipyrone A	dipyr	0.0000558	0.29	0.0245	*H. italicum* ssp. *microphyllum*, aerial parts with flowers	[10]	↓↓	Much lower than this *H. italicum* ssp. *microphyllum* report.
**4**	helipyrone A	dipyr	0.0000558	0.29	0.022	*H. italicum* ssp. *microphyllum*, aerial parts with flowers	[33]	↓↓	Much lower than this *H. italicum* ssp. *microphyllum* report.
**4**	helipyrone A	dipyr	0.0000558	0.29	0.00007	*H. oocephalum*, aerial parts	[5]	≈	Close to the low *H. oocephalum* yield.
**4**	helipyrone A	dipyr	0.0000558	0.29	0.0025	*H. italicum*	[15]	↓	Lower than this *H. italicum* report.
**8**	helispiroketal F	spiro	0.000679	3.55	0.000023	*H. italicum*, aerial parts with flowers	[12]	↑	Higher than the reported isolated yield; response-factor effects should be considered.
**18**, **19**	italipyrone	benzofuran	0.000246	1.29	0.000068	*H. italicum*	[8]	≈	Combined apparent level is higher but still the same order of magnitude.
**18**, **19**	italipyrone	benzofuran	0.000246	1.29	qualitative	*H. stoechas*, aerial parts	[17]	n/a	Qualitative confirmation only; no yield reported.
**20**	arzanol	phlor_pyr	0.00542	28.29	0.48	*H. stoechas*, flowers	[14]	↓	Lower than the high arzanol-rich *H. stoechas* source.
**20**	arzanol	phlor_pyr	0.00542	28.29	0.295	*H. italicum* ssp. *microphyllum*, dried aerial parts with flowers	[11]	↓	Lower than this arzanol-rich *H. italicum* ssp. *microphyllum* report.
**20**	arzanol	phlor_pyr	0.00542	28.29	0.0965	*H. italicum* ssp. *microphyllum*, dried aerial parts with flowers	[10]	↓	Lower than this *H. italicum* ssp. *microphyllum* report.
**20**	arzanol	phlor_pyr	0.00542	28.29	0.081	*H. italicum* ssp. *microphyllum*, dried aerial parts with flowers	[33]	↓	Lower than this *H. italicum* ssp. *microphyllum* report.
**20**	arzanol	phlor_pyr	0.00542	28.29	0.002	*H. italicum* (Roth) G. Don, dried aerial parts with flowers	[12]	≈	Higher than this lower *H. italicum* report, but the same order of magnitude.
**26**	helitalone B	phlor_pyr	0.0000805	0.42	0.00062	*H. italicum*	[12]	≈	Slightly lower, but still within the same order of magnitude.
**28**	arenol B	phlor_pyr	0.000187	0.98	0.00001	*H. oocephalum*, aerial parts	[5]	↑	Higher than the very low *H. oocephalum* yield.
**30**, **31**	plicatipyrone	benzopyran	0.000267	1.39	0.00022	*H. plicatum*, flower heads	[8]	≈	Very close to the reported *H. plicatum* isolated yield.
**30**, **31**	plicatipyrone	benzopyran	0.000267	1.39	not reported	*H. stoechas*, aerial parts and flowers	[17]	n/a	Identified but yield was not reported.
**35**	arenol C	phlor_pyr	0.00063	3.29	0.00002	*H. oocephalum*, aerial parts	[5]	↑	Higher than the reported *H. oocephalum* yield.
**36**	3-prenyl norauricepyrone	phlor_pyr	0.000491	2.56	0.00001	*H. oocephalum*, aerial parts	[5]	↑	Higher than the very low *H. oocephalum* yield.
**36**	6-O-Desmethylauricepyrone	phlor_pyr	0.000491	2.56	0.133	*H. odoratissimum*	[8]	↓↓	Much lower than the high *H. odoratissimum* yield.
**38**, **39**	22-Methyl-22-ethyl-italipyrone	benzofuran	0.0000323	0.17	0.005	*H. cephaloideum*, roots	[8]	↓↓	Much lower than the reported root yield.
**38**, **39**	22-Methyl-22-ethyl-italipyrone	benzofuran	0.0000323	0.17	0.0079	*H. cephaloideum*, roots	[18]	↓↓	Much lower than the reported root yield.
**38**, **39**	22-Methyl-22-ethyl-italipyrone	benzofuran	0.0000323	0.17	0.05	*H. mixtum*, roots	[18]	↓↓↓	More than three orders of magnitude lower than the *H. mixtum* root yield.
**40**	23-Methyl-6-O-desmethylauricepyrone	phlor_pyr	0.000739	3.86	0.033	*H. odoratissimum*	[8]	↓	Lower than the *H. odoratissimum* yield.
**40**	23-Methyl-6-O-desmethylauricepyrone	phlor_pyr	0.000739	3.86	0.07	*H. mixtum*, roots	[18]	↓	Lower than the *H. mixtum* root yield.
**40**	23-Methyl-6-O-desmethylauricepyrone	phlor_pyr	0.000739	3.86	0.09	*H. stenopterum*, aerial parts	[18]	↓↓	About two orders of magnitude lower than the *H. stenopterum* aerial-part yield.
**42**	18,18-bis-desmethyl achyroclinopyrone C	phlor_pyr	0.00134	6.99	0.0857	*H. stoechas*, flowers and aerial parts	[14]	↓	Lower than the high *H. stoechas* yield.
**42**	18,18-bis-desmethyl achyroclinopyrone C	phlor_pyr	0.00134	6.99	0.0032	*H. decumbens*, aerial parts	[19]	≈	Lower, but within the same order of magnitude.
**42**	18,18-bis-desmethyl achyroclinopyrone C	phlor_pyr	0.00134	6.99	not reported	*H. stoechas*, roots	[16]	n/a	Qualitative report only; no yield available.
**43**, **44**	23-ethyl-6-O-desmethyl-auricepyrone	phlor_pyr	0.000505	2.64	0.07	*H. mixtum*, roots	[18]	↓↓	Much lower than the *H. mixtum* root yield.
**43**, **44**	23-ethyl-6-O-desmethyl-auricepyrone	phlor_pyr	0.000505	2.64	0.09	*H. stenopterum*, aerial parts	[18]	↓↓	Much lower than the *H. stenopterum* aerial-part yield.
**47**	18,18-bis-desmethyl achyroclinopyrone A	phlor_pyr	0.00178	9.27	0.0008	*H. decumbens*	[19]	≈	Higher than the *H. decumbens* yield, but the same order of magnitude.
**47**	18,18-bis-desmethyl achyroclinopyrone A	phlor_pyr	0.00178	9.27	not reported	*H. stoechas*, roots	[16]	n/a	Qualitative report only; no yield available.
**50**	8′-methyl-18,18-bis-desmethyl achyroclinopyrone A	phlor_pyr	0.0000144	0.08	trace	*H. decumbens*	[19]	n/a	Consistent with trace-level occurrence; no exact literature yield available.
**59**, **60**	helispiroketal A	spiro	0.000476	2.51	0.000048	*H. italicum*, aerial parts with flowers	[12]	↑	An order of magnitude higher than reported.

Literature values represent reported isolated yields (% *w*/*w*) and are therefore used for order-of-magnitude comparison rather than direct absolute quantification. When several literature reports were available for the same compound, each species/material source was listed separately, and the original citation was retained. The semi-quantitative workflow used a 1/x weighted calibration with 2′,4′,6′-trihydroxyacetophenone monohydrate as reference standard. Assignments in this study correspond to a Level 2b confidence level [36]. Legend for “Fold-symbol” column: ≈ = same order of magnitude; this/literature ratio between 0.1 and 10. ↓ = approximately 10-fold lower; ratio between 0.01 and 0.1. ↓↓ = approximately 100-fold lower; ratio between 0.001 and 0.01. ↓↓↓ = approximately 1000-fold lower or more; ratio below 0.001. ↑ = approximately 10-fold higher; ratio between 10 and 100. n/a = comparison not calculated because the literature report was qualitative, described the compound as trace, or did not provide an exact yield.
