# Supplementary material for: Comprehensive UHPLC–Orbitrap–MS Profiling of Phloroglucinol α-Pyrones in Helichrysum italicum (Roth) G. Don (Asteraceae)
Source: Plants (Basel). 2026 Aug 27;15(17):2619. doi: 10.3390/plants15172619 (PMC13567118; doi:10.3390/plants15172619)

ESI(–) MS/MS spectra of the annotated compounds

This document compiles the negative-ion (deprotonated, $[M–H]^-$) tandem mass spectrometry (MS/MS) spectra for every compound annotated in this study. One panel is shown per compound; compounds that were annotated as isomers or co-eluting analogues are presented on separate panels so that each retention time (R_t) is documented individually.

Associated article

Voynikov Y, Marinov T, Nedialkov P. *Comprehensive UHPLC–Orbitrap–MS Profiling of Phloroglucinol α-Pyrones in Helichrysum italicum (Roth) G. Don (Asteraceae)*. Plants, 2026.

Compound #1 **Rt = 2.93 min**

$C_{14}H_{19}O_4^-$ $[M\text{-}H]^- = 251.1289$

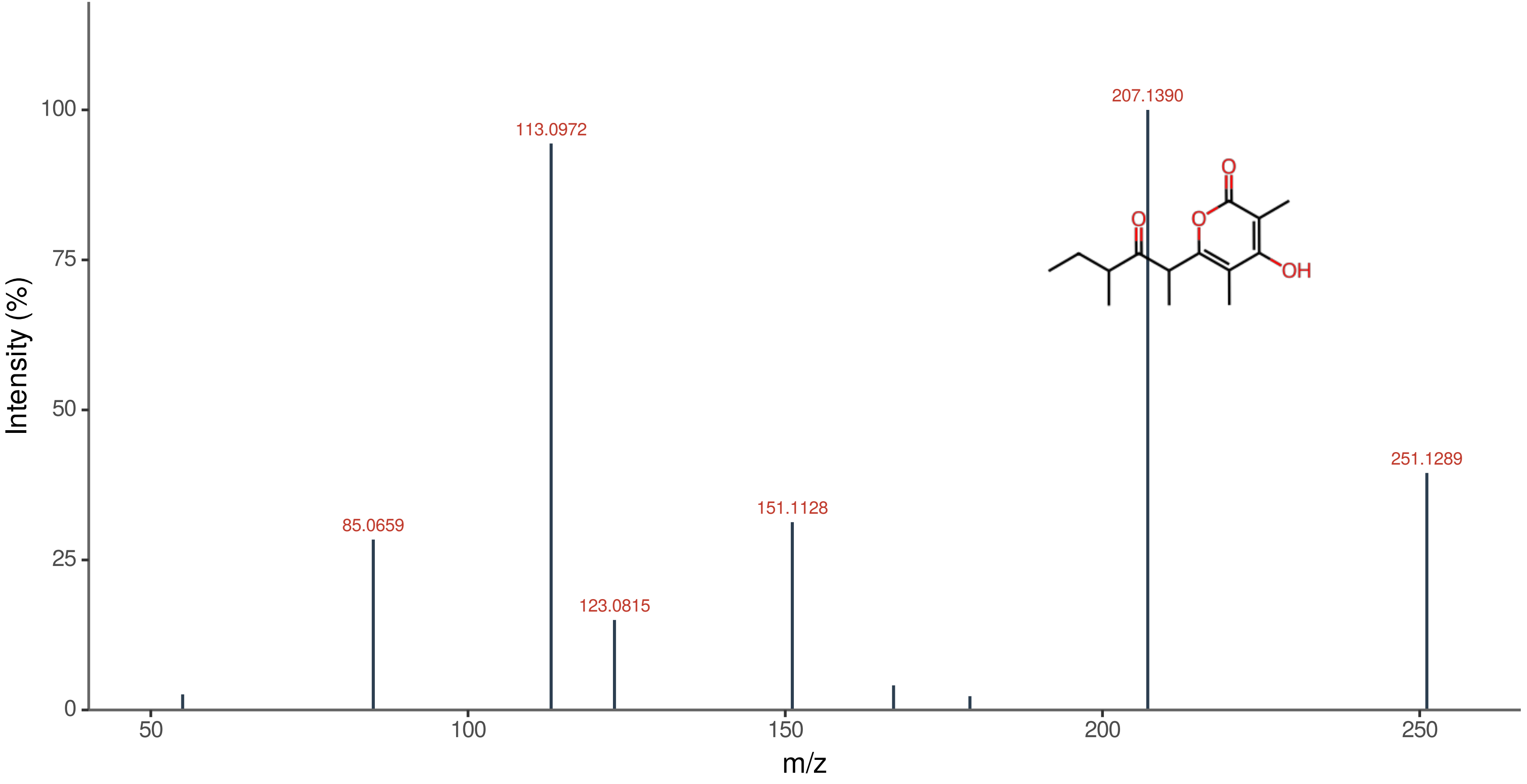

Compound #2 **Rt = 8.74 min**

$C_{16}H_{17}O_6^-$ $[M-H]^- = 305.1033$

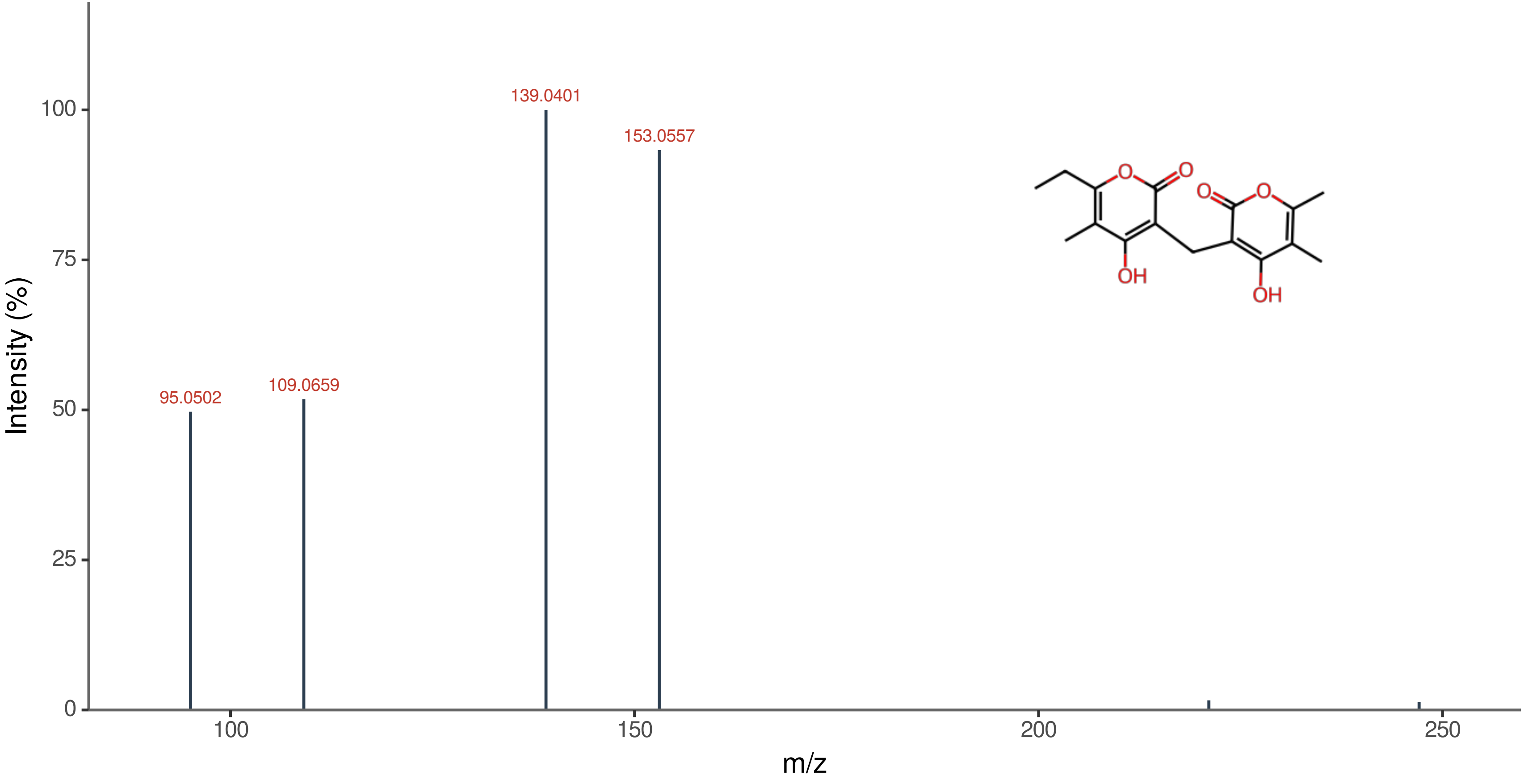

Compound #3 Rt = 5.12 min

$C_{16}H_{15}O_7^-$ $[M-H]^- = 319.0825$

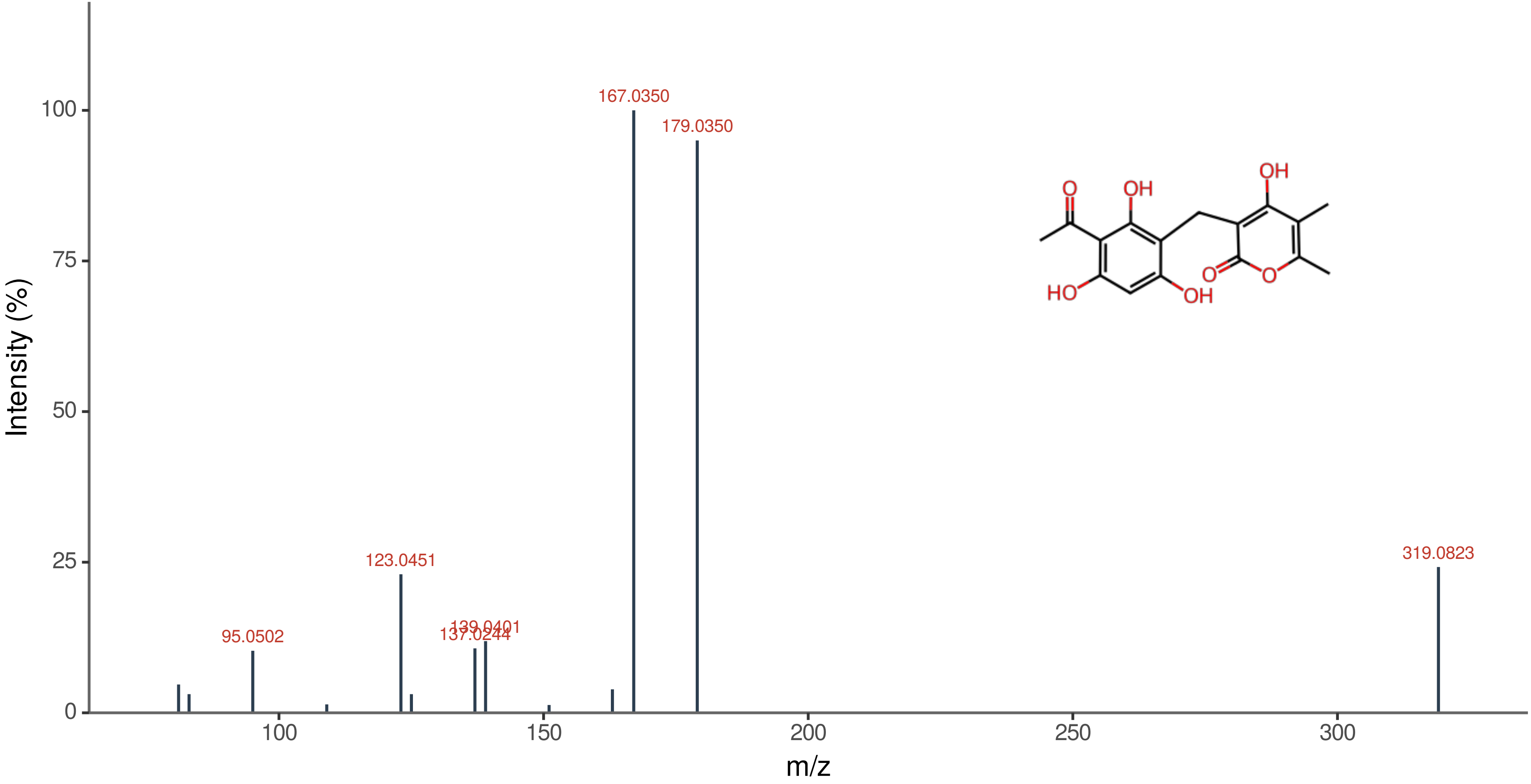

Compound #4 **Rt = 11.25 min**

$C_{17}H_{19}O_6^-$ $[M-H]^- = 319.1186$

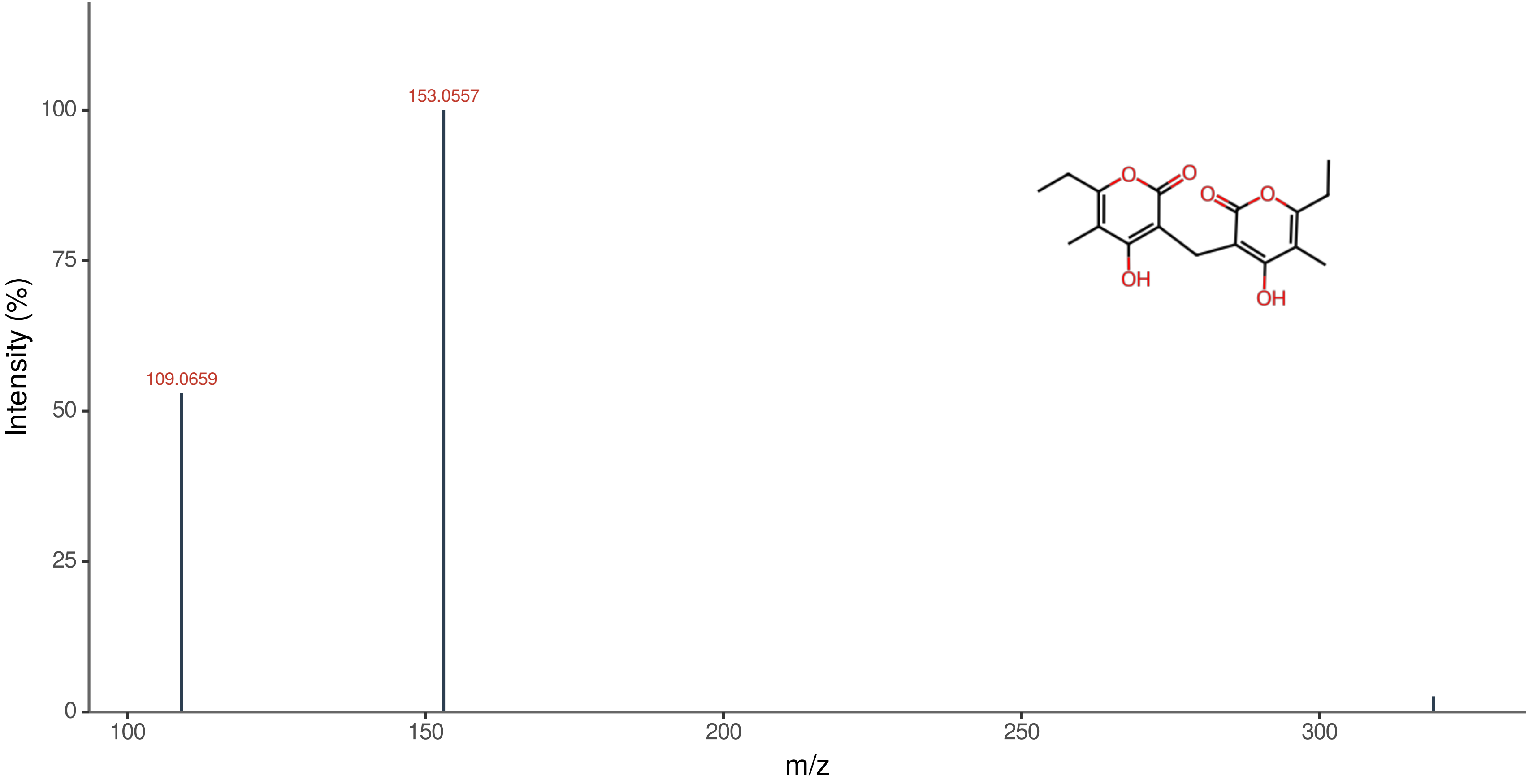

Compound #5 Rt = 6.77 min

$C_{17}H_{17}O_7^-$ $[M-H]^- = 333.0985$

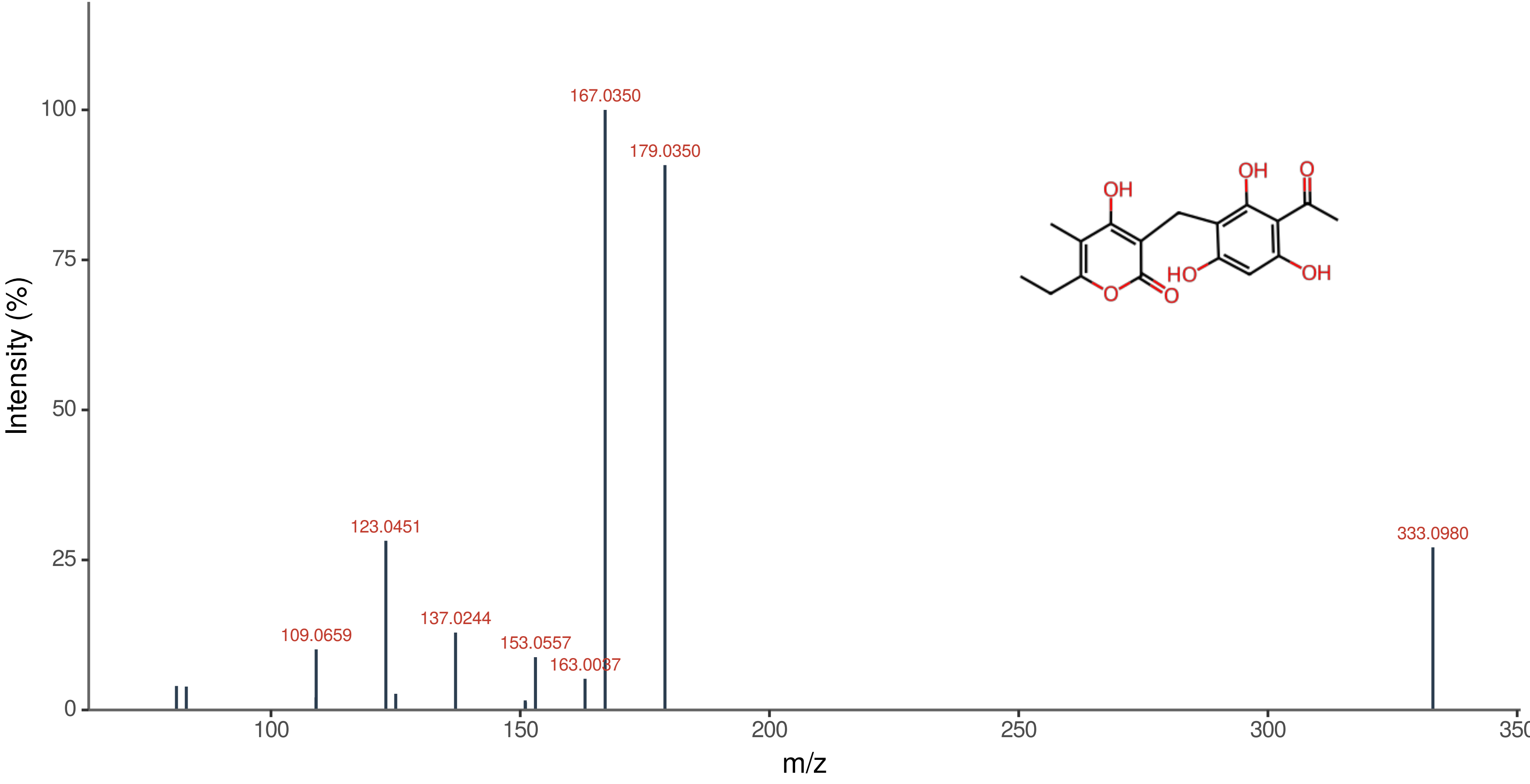

Compound #6 Rt = 10.93 min

$C_{19}H_{21}O_7^-$ $[M\text{-}H]^- = 361.1295$

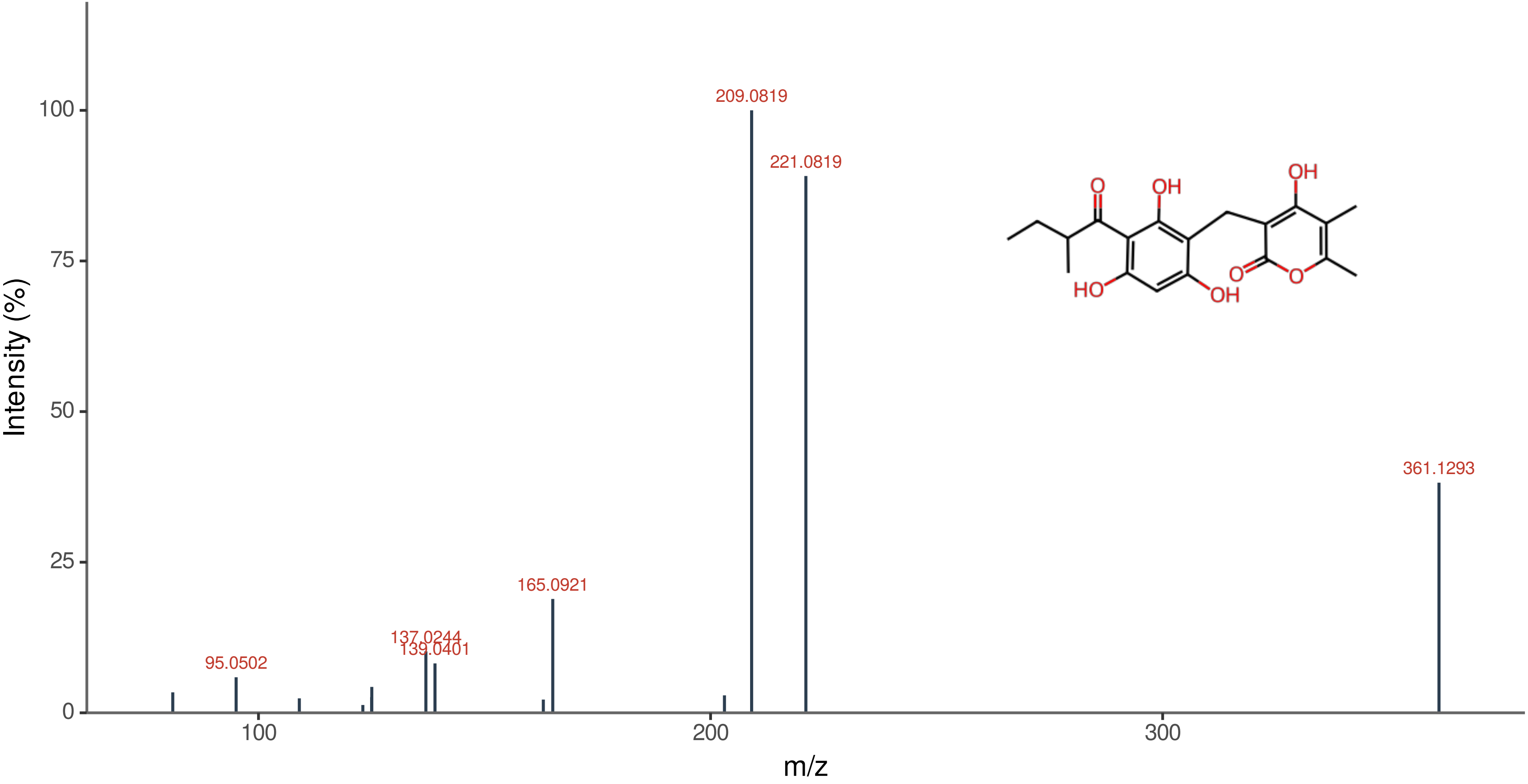

Compound #7 **Rt = 11.35 min**

$C_{19}H_{21}O_7^-$ $[M-H]^- = 361.1295$

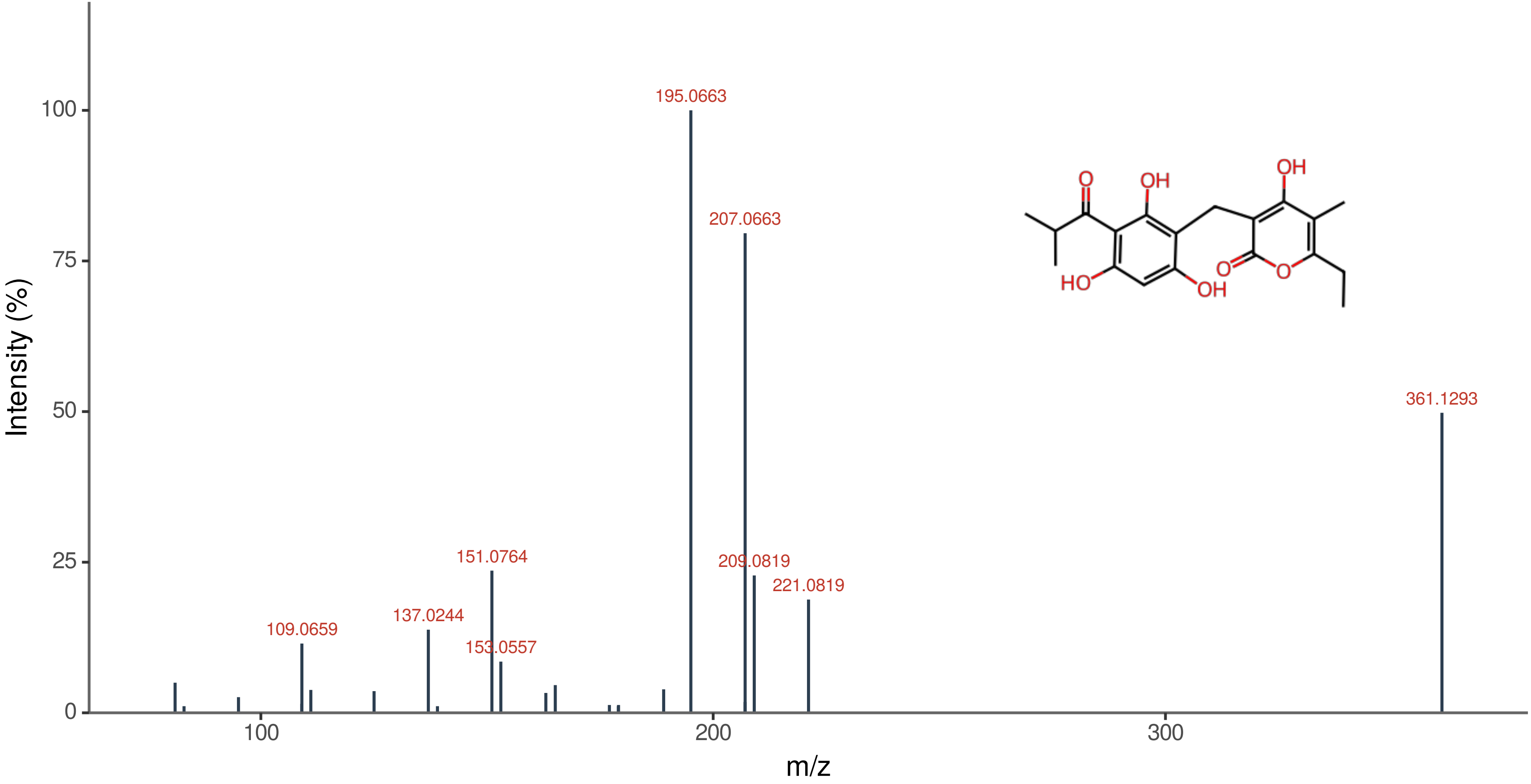

Compound #8 Rt = 11.24 min

$C_{21}H_{23}O_6^-$ $[M-H]^- = 371.1498$

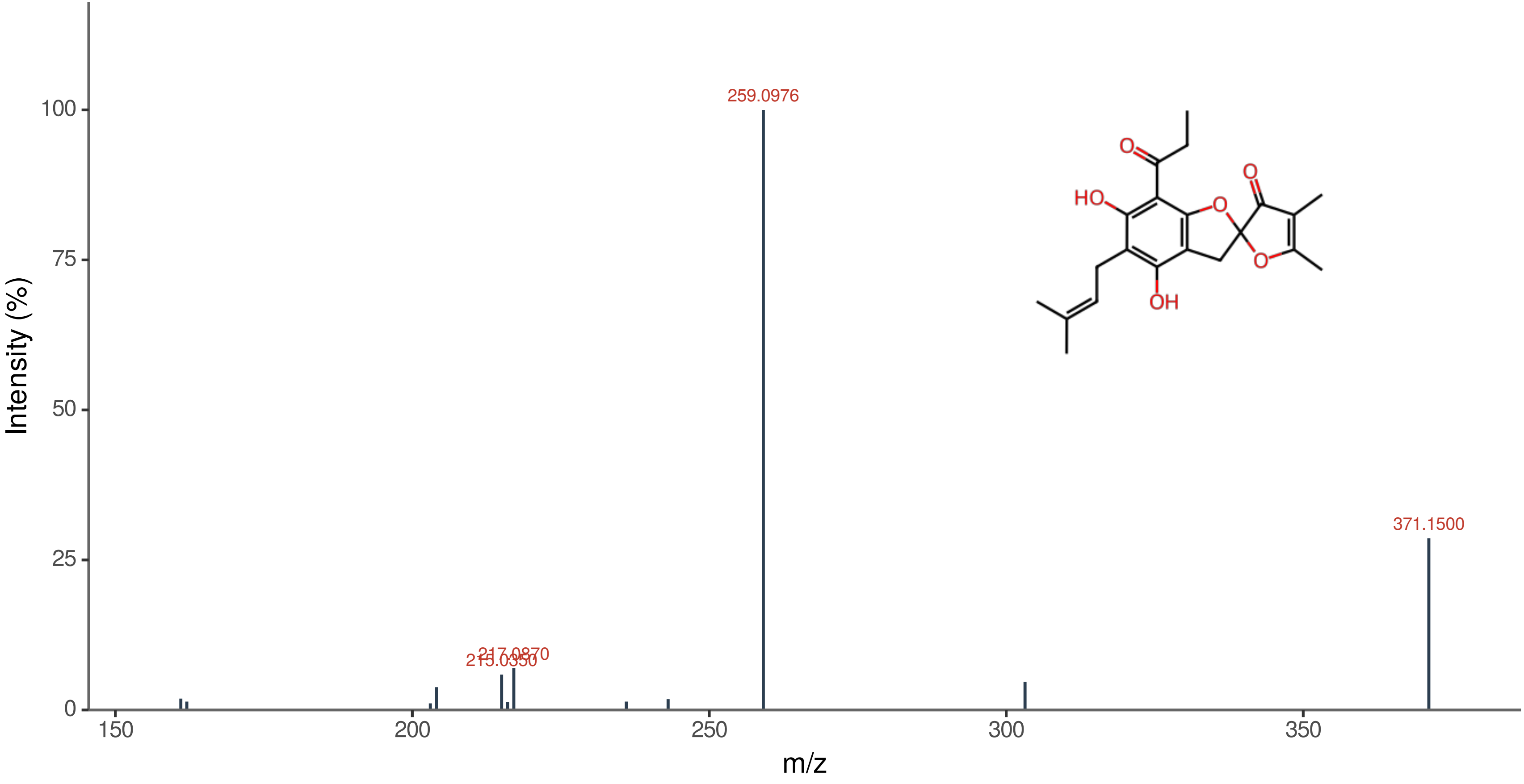

Compound #9 **Rt = 13.60 min**

$C_{20}H_{23}O_7^-$ $[M-H]^- = 375.1451$

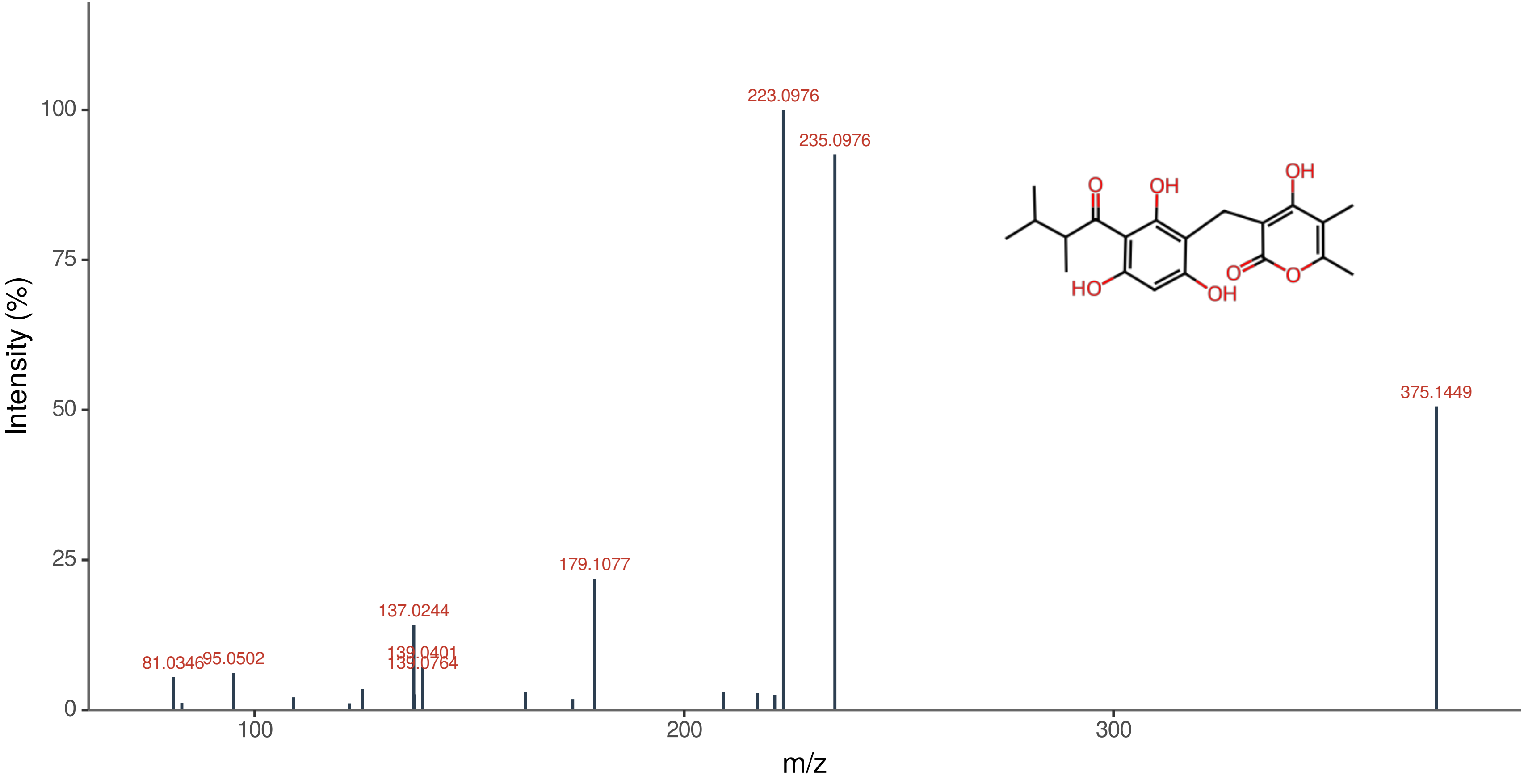

Compound #10 Rt = 13.14 min

$C_{20}H_{23}O_7^-$ $[M-H]^- = 375.1451$

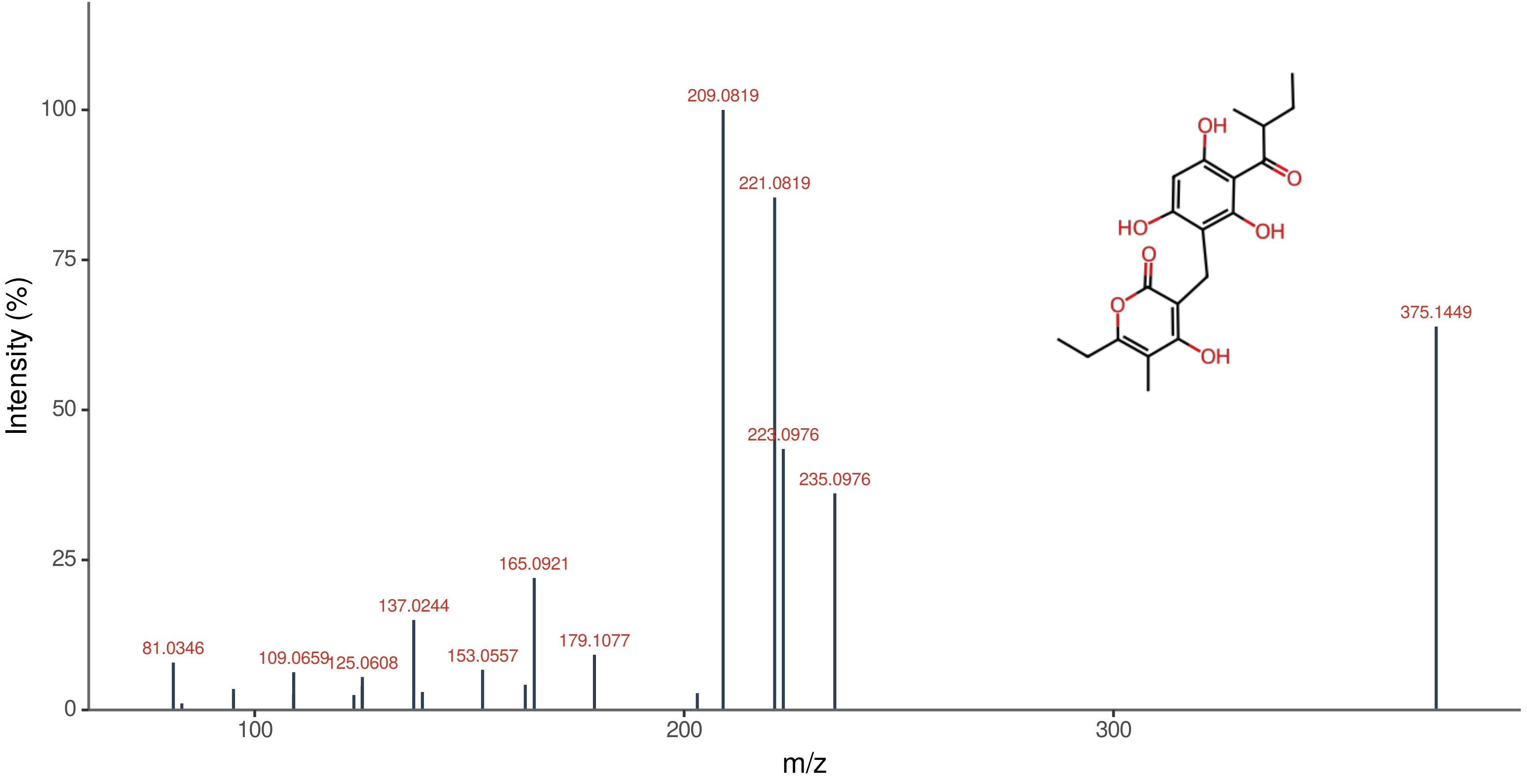

Compound #11   Rt = 13.90 min

$C_{21}H_{21}O_7^-$ $[M-H]^- = 385.1297$

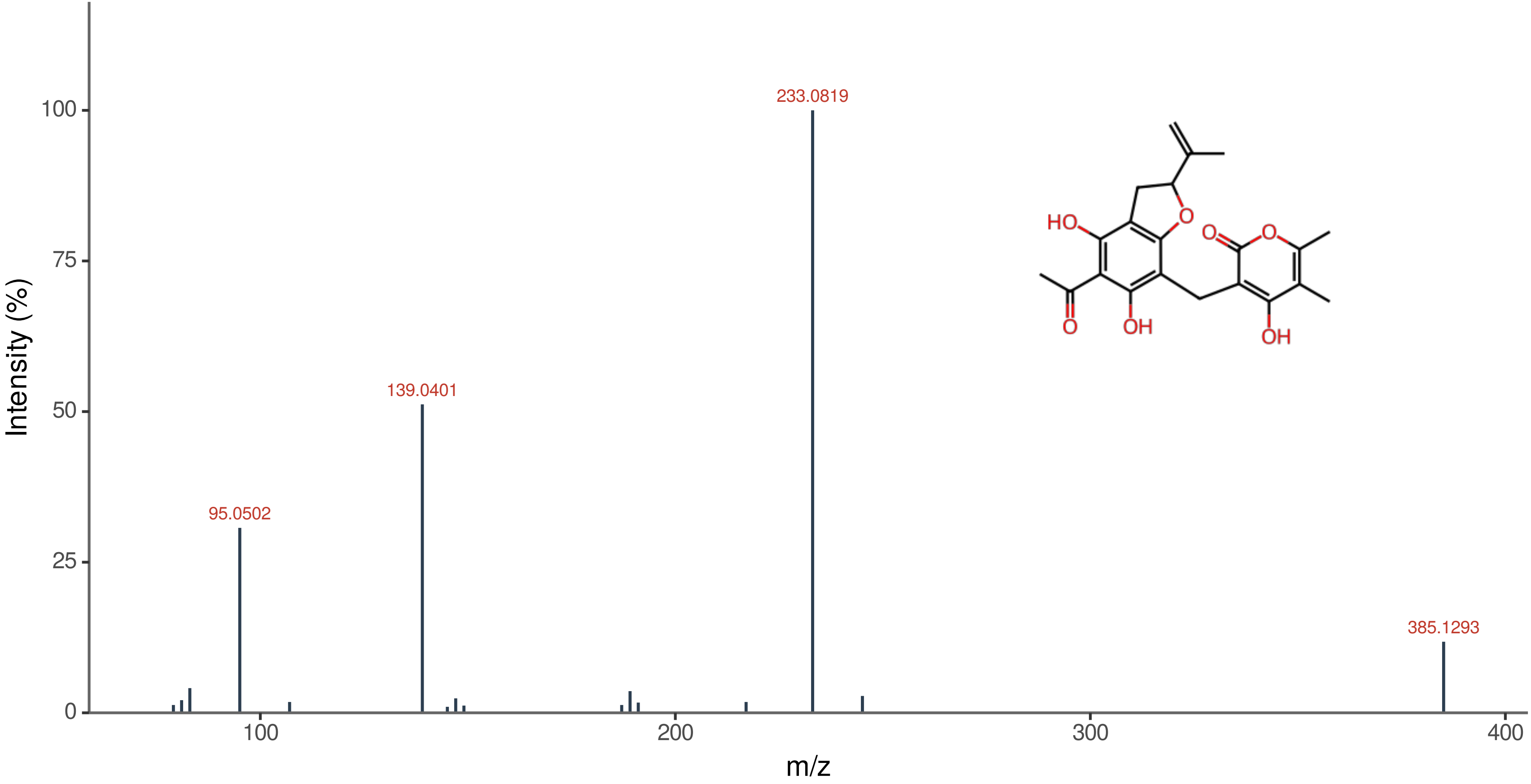

Compound #12 **Rt = 17.00 min**

$C_{21}H_{21}O_7^-$ $[M-H]^- = 385.1294$

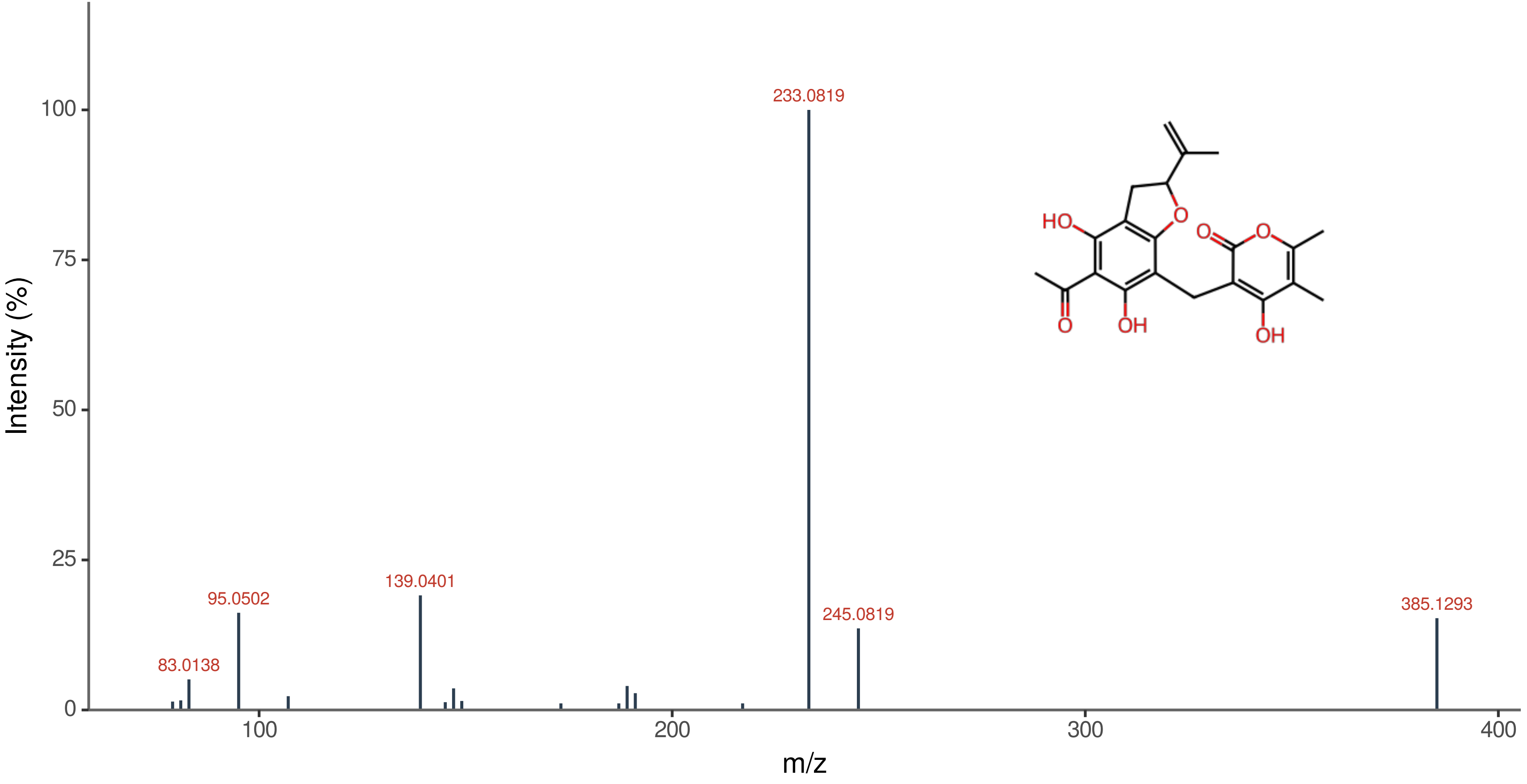

Compound #13 Rt = 13.61 min

$C_{22}H_{25}O_6^-$ $[M-H]^- = 385.1660$

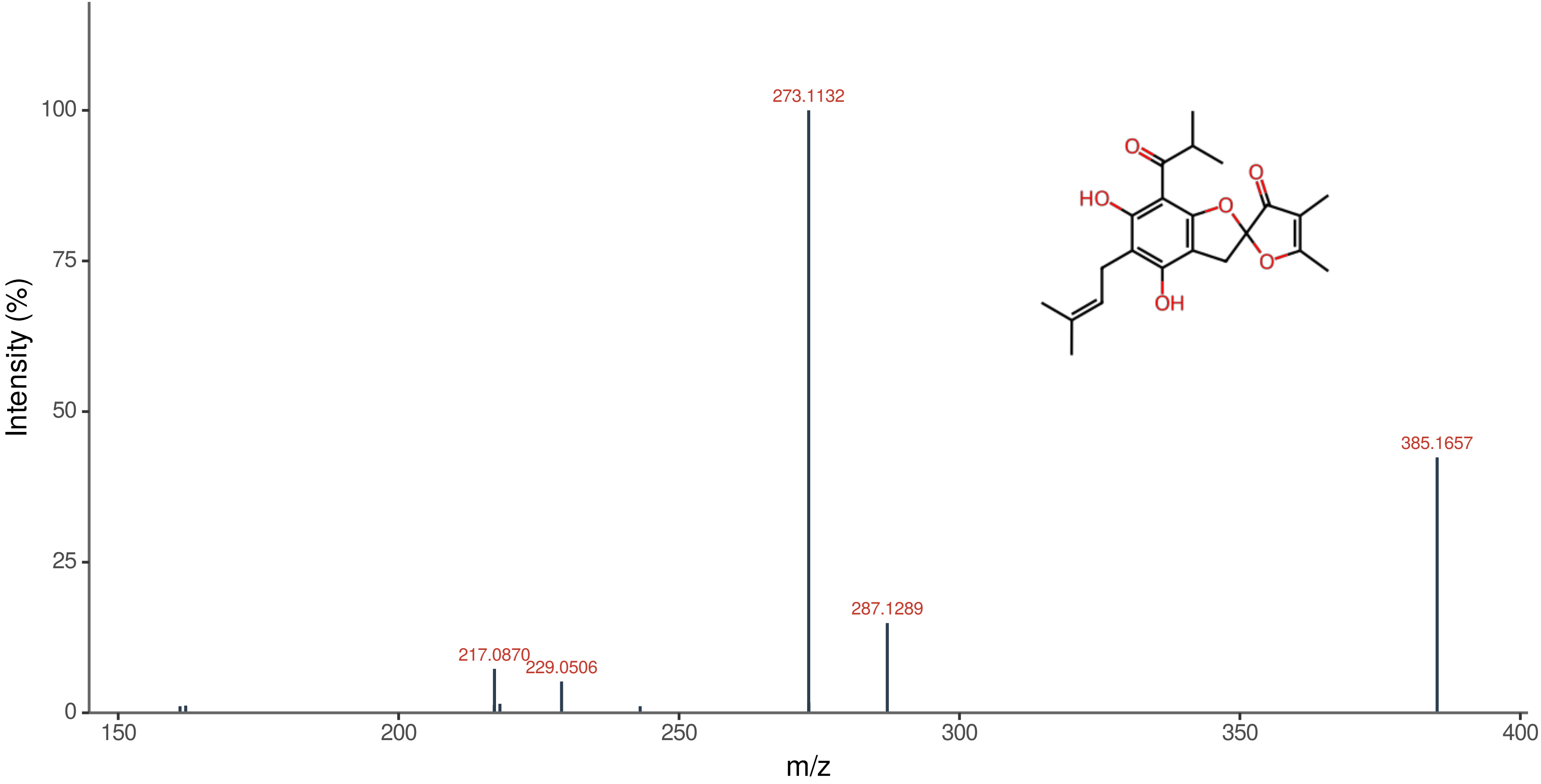

Compound #14 **Rt = 8.46 min**

$C_{21}H_{23}O_7^-$ $[M-H]^- = 387.1448$

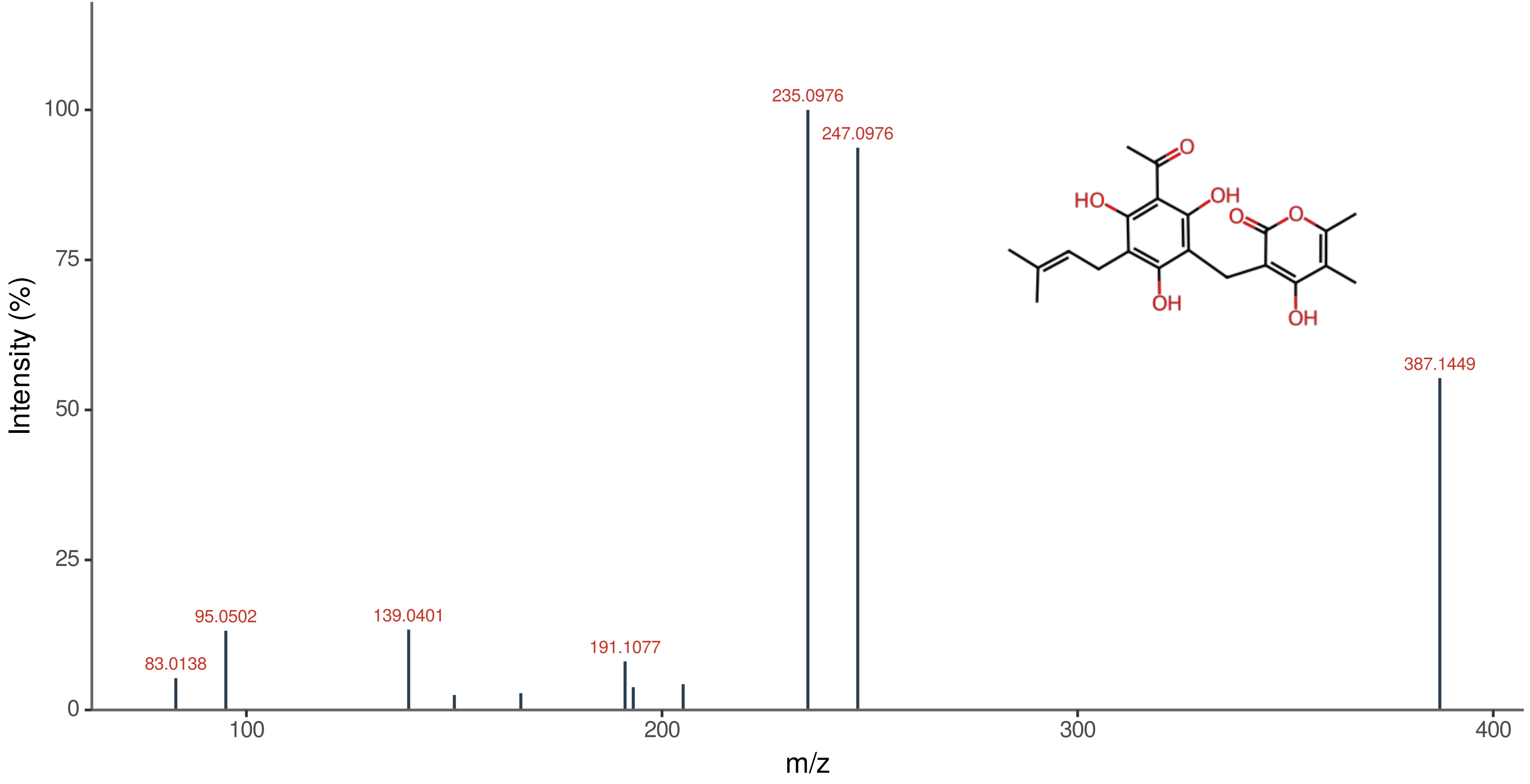

Compound #15 **Rt = 15.37 min**

$C_{21}H_{25}O_7^-$ $[M-H]^- = 389.1610$

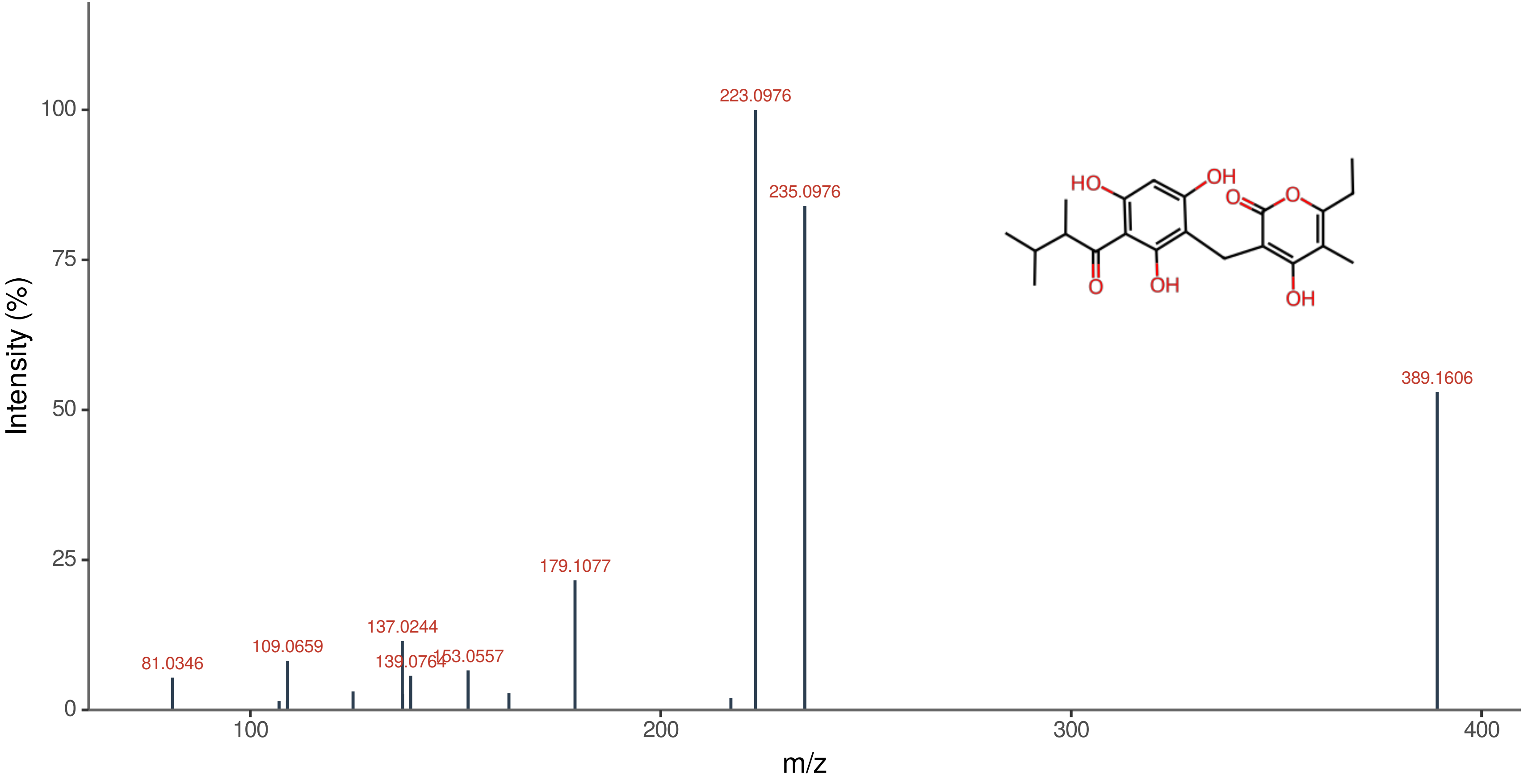

Compound #16 **Rt = 15.86 min**

$C_{21}H_{25}O_7^-$ $[M\text{-}H]^- = 389.1610$

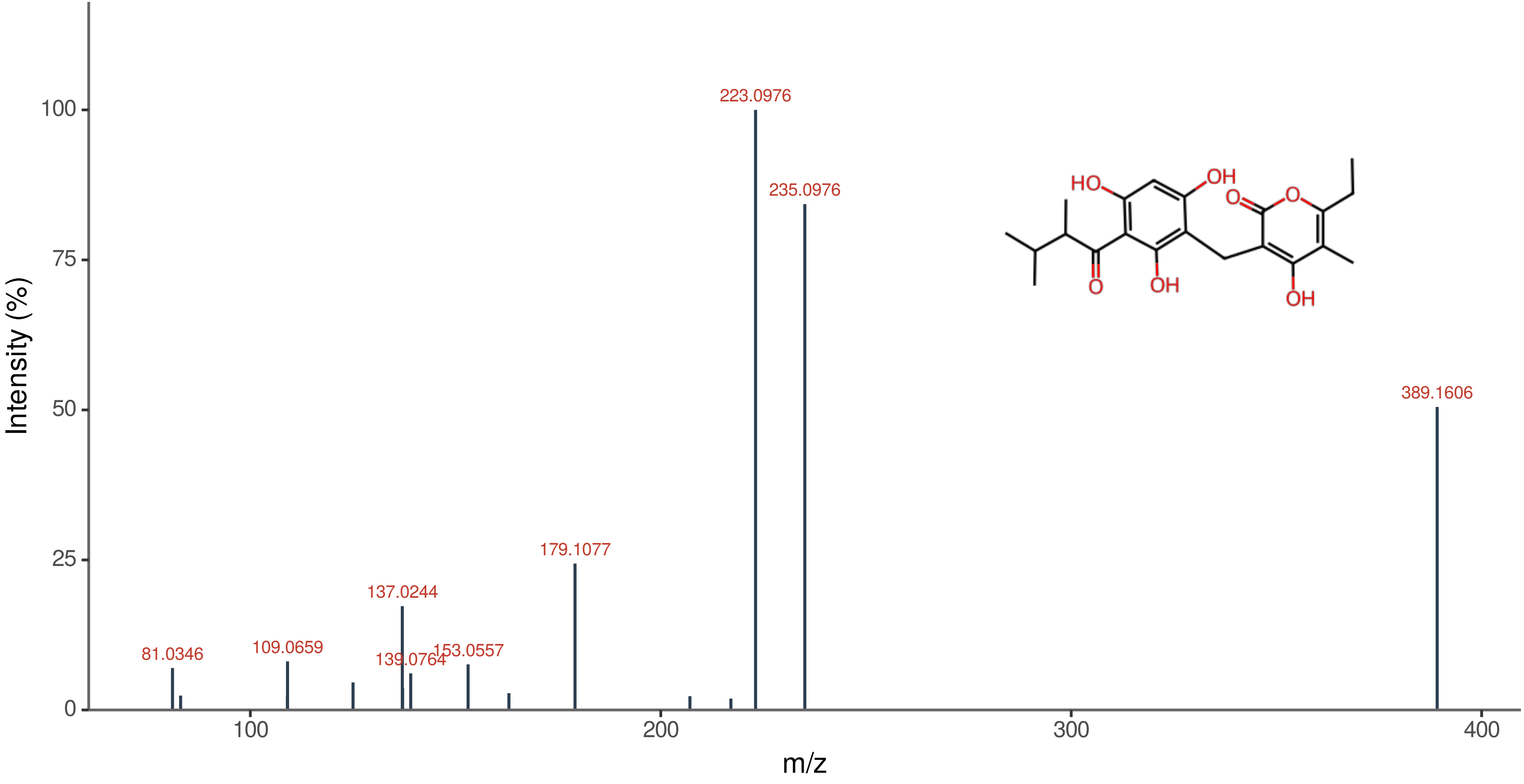

Compound #17 Rt = 15.10 min

$C_{21}H_{25}O_7^-$ $[M-H]^- = 389.1608$

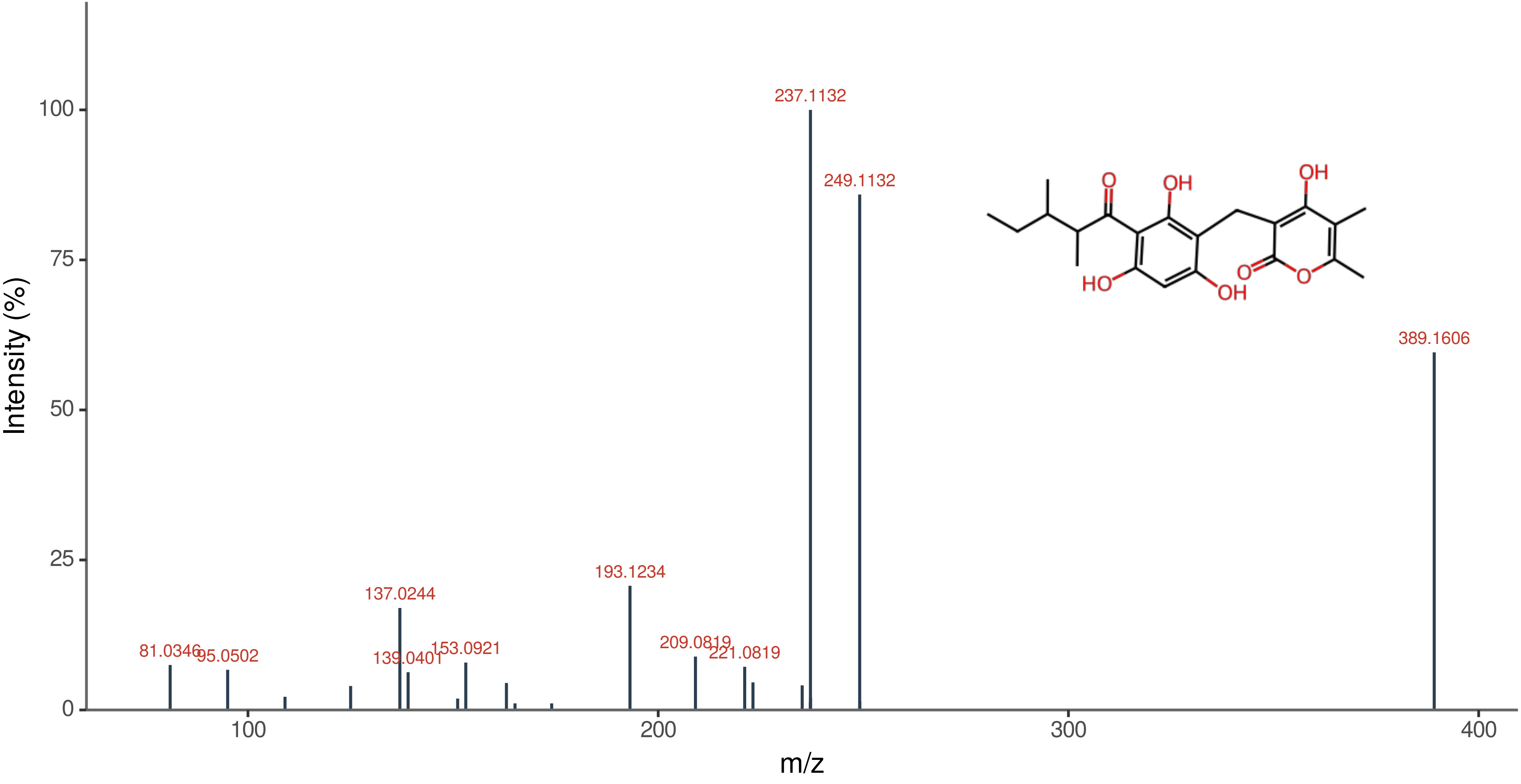

Compound #18 Rt = 16.43 min

$C_{22}H_{23}O_7^-$ $[M-H]^- = 399.1449$

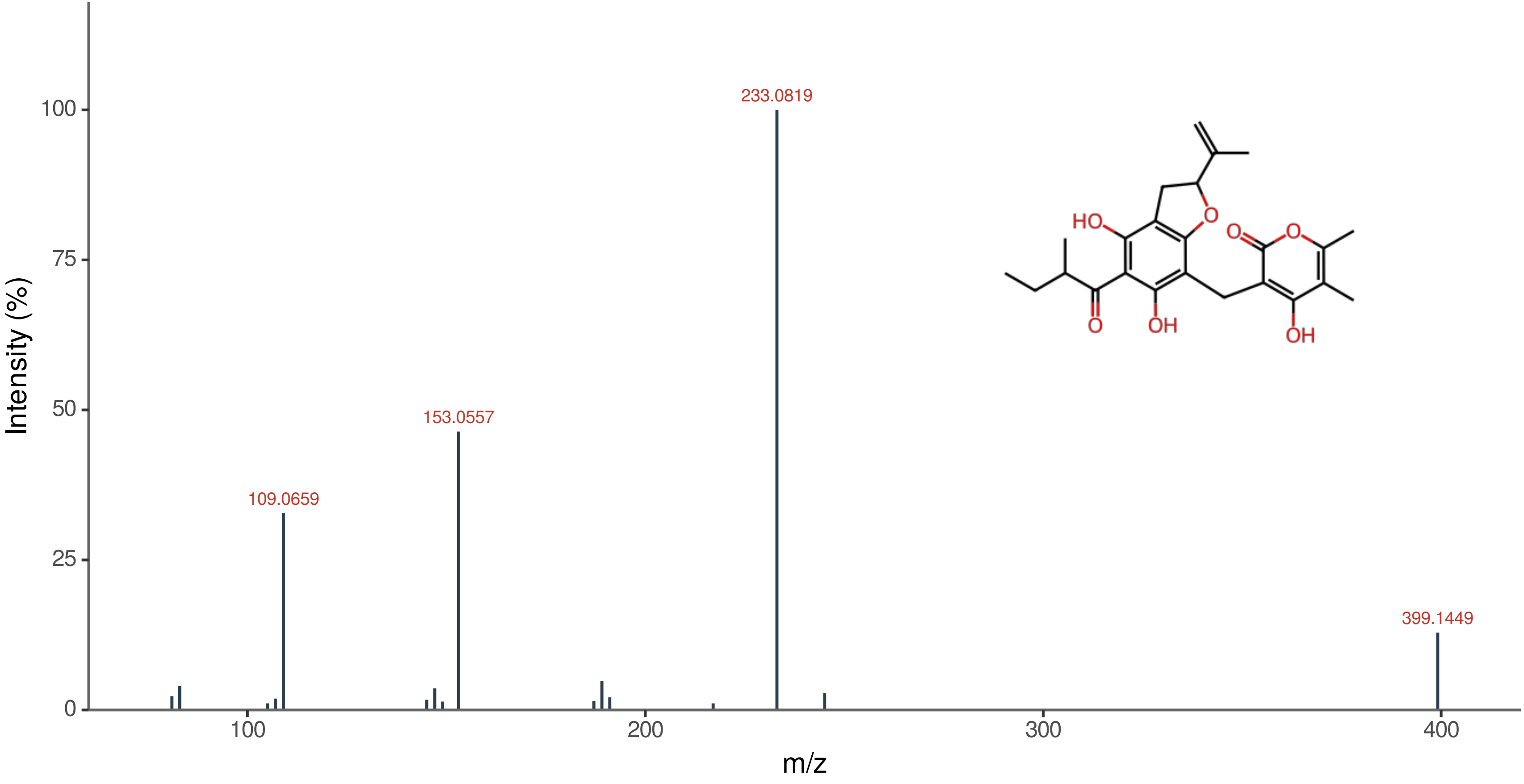

Compound #19 **Rt = 19.64 min**

$C_{22}H_{23}O_7^-$ $[M-H]^- = 399.1450$

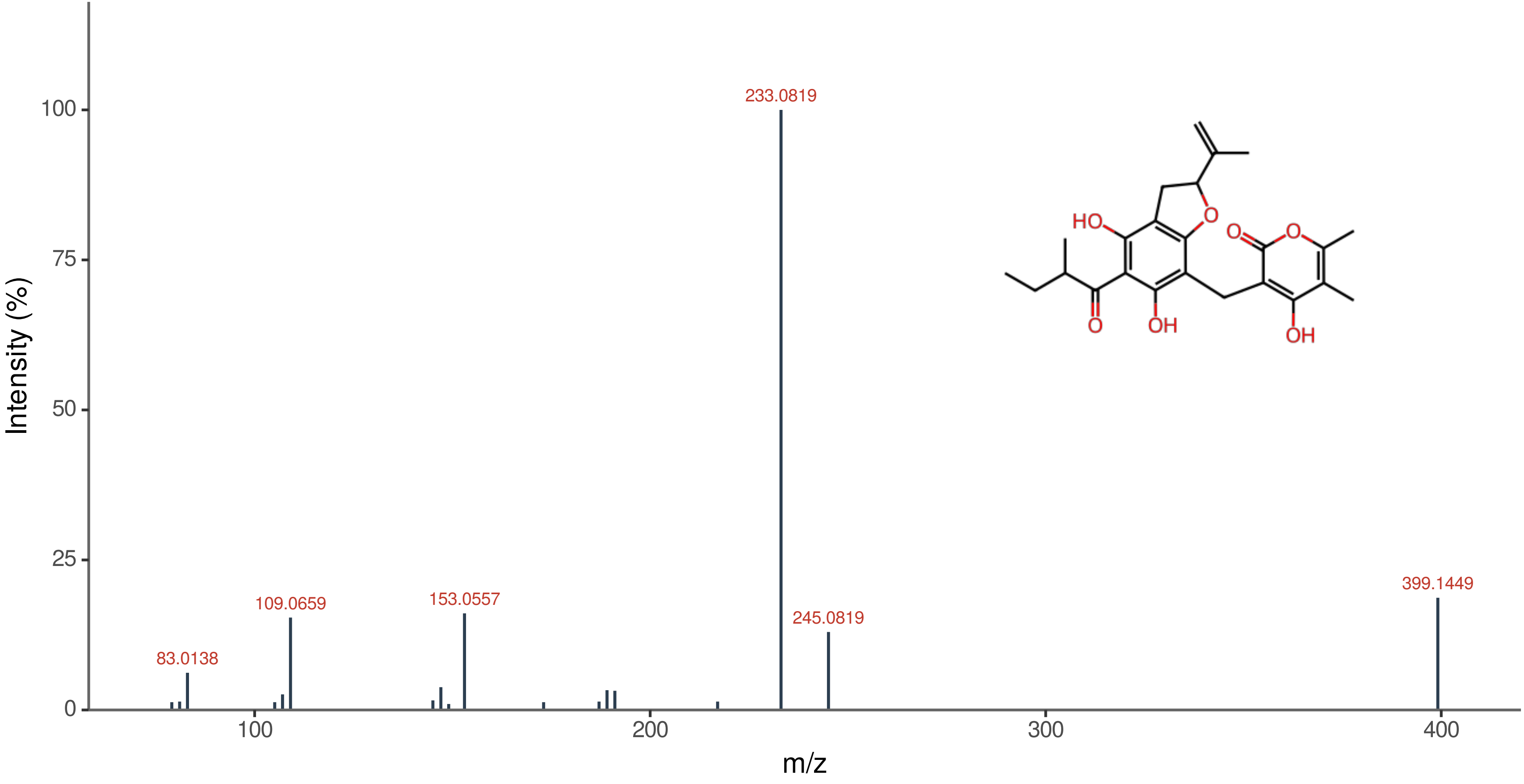

Compound #20 Rt = 10.37 min

$C_{22}H_{25}O_7^-$ $[M-H]^- = 401.1602$

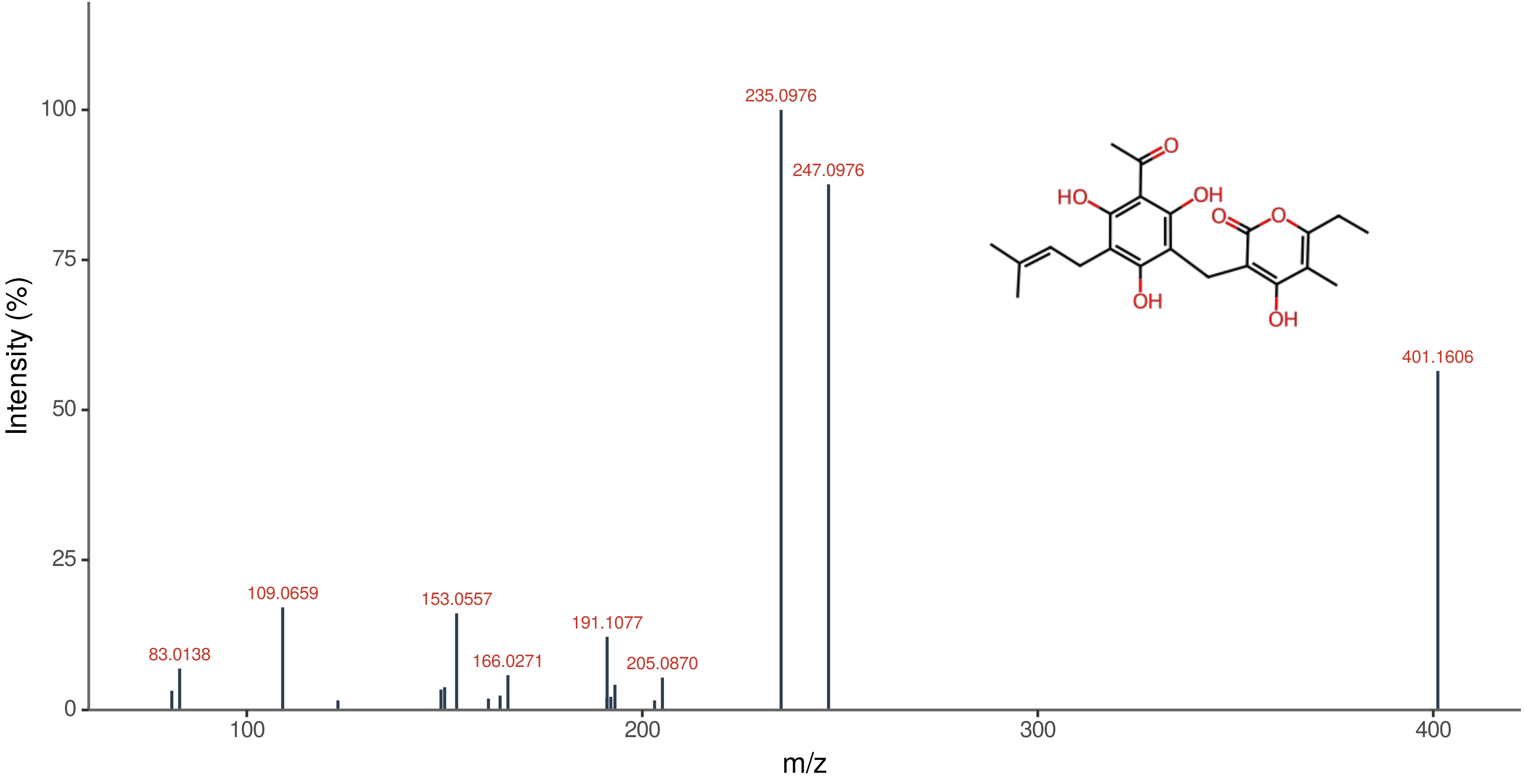

Compound #21 Rt = 11.23 min

$C_{22}H_{25}O_7^-$ $[M-H]^- = 401.1605$

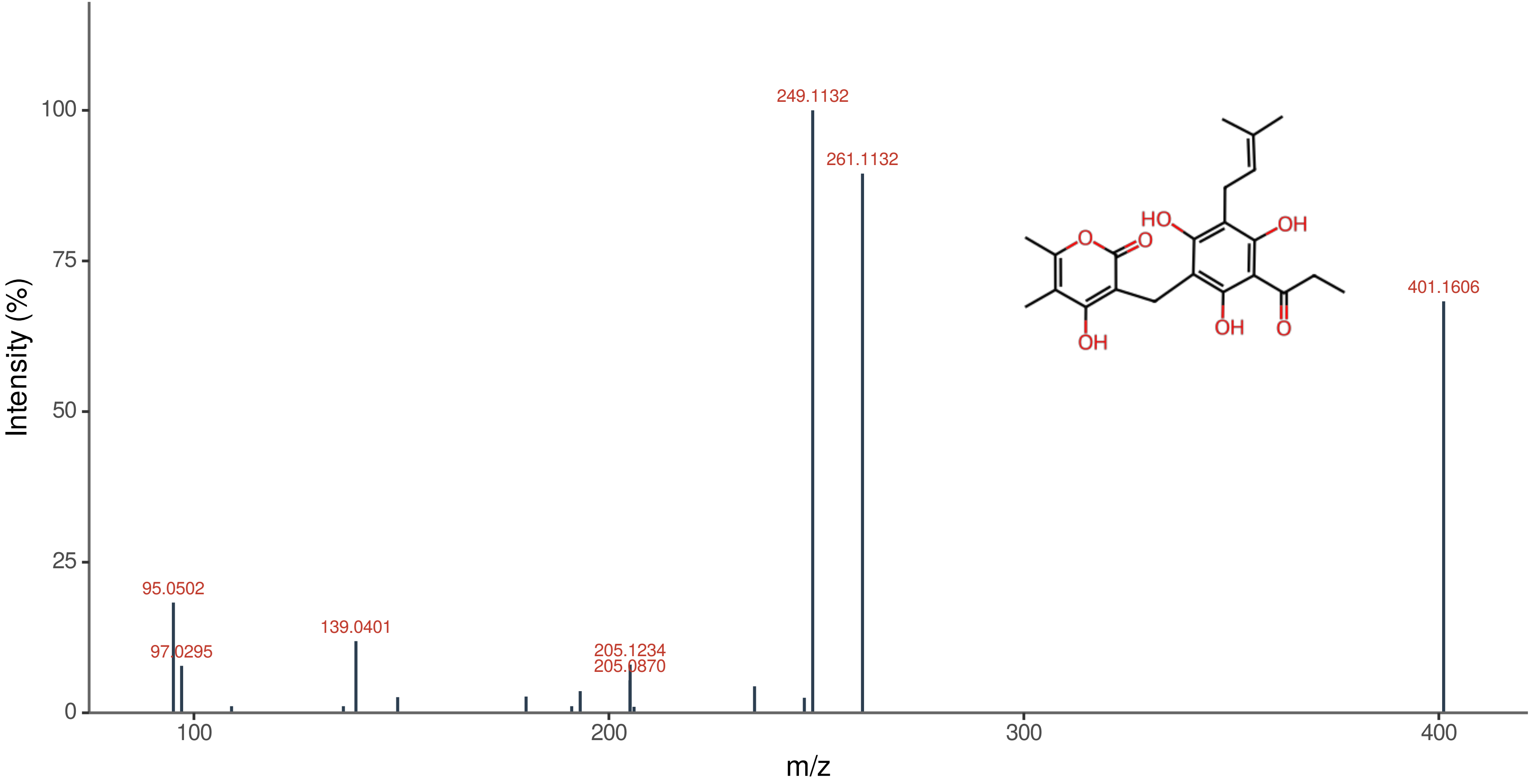

Compound #22 **Rt = 4.85 min**

$C_{21}H_{23}O_8^-$ $[M-H]^- = 403.1400$

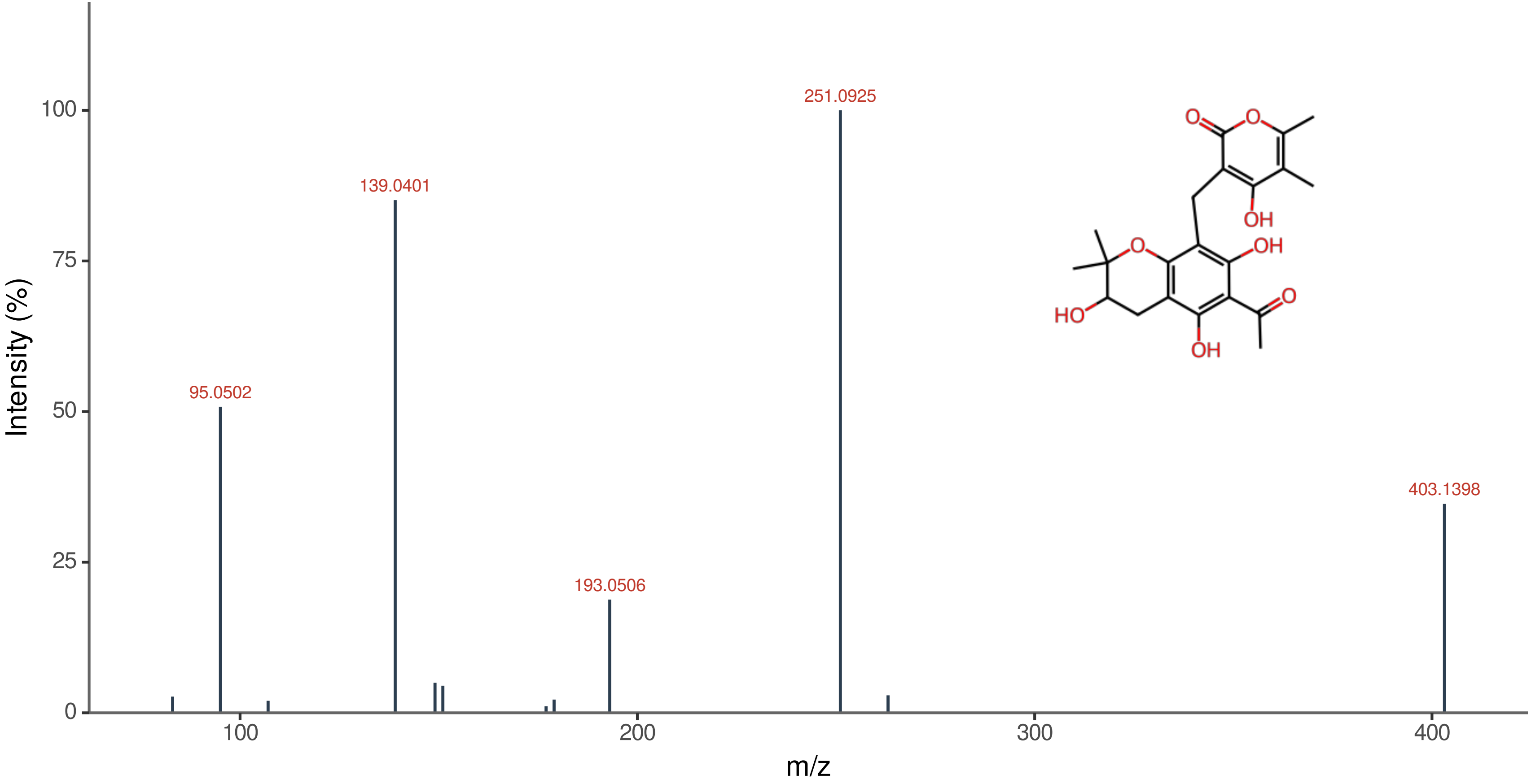

Compound #23 **Rt = 6.90 min**

$C_{21}H_{23}O_8^-$ $[M-H]^- = 403.1400$

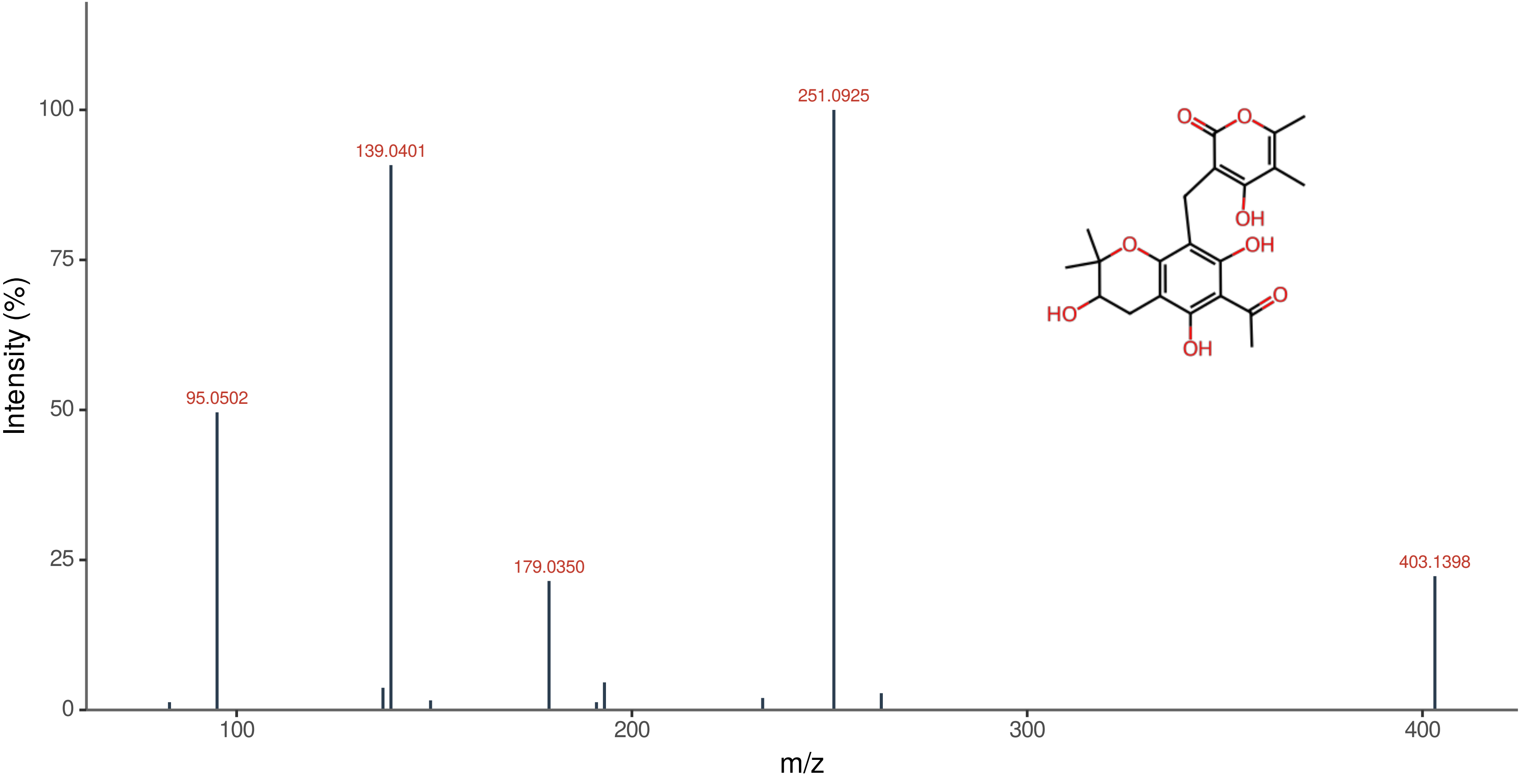

Compound #24 Rt = 9.97 min

$C_{21}H_{23}O_8^-$ $[M-H]^- = 403.1400$

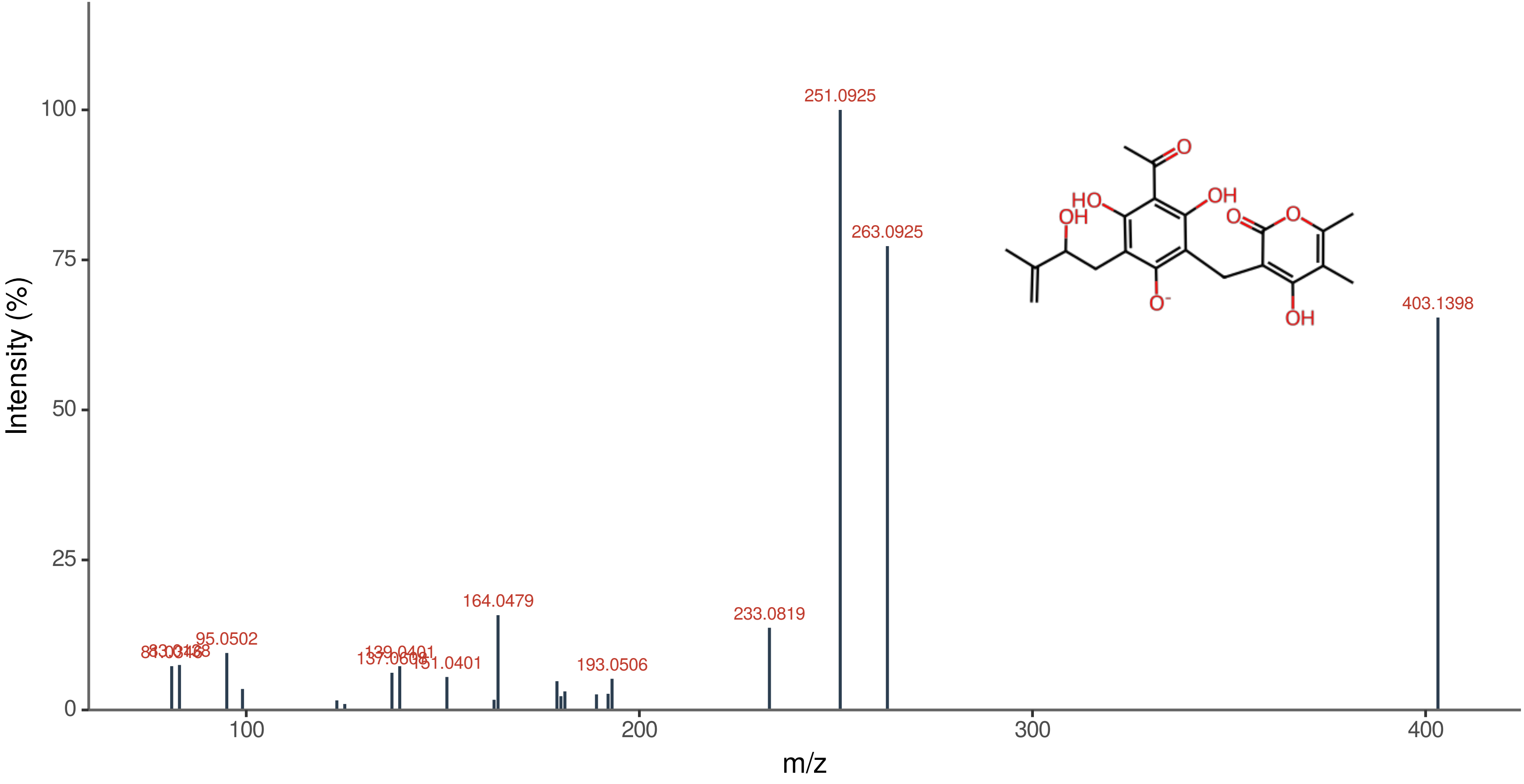

Compound #25 Rt = 5.44 min

$C_{21}H_{25}O_8^-$ $[M-H]^- = 405.1556$

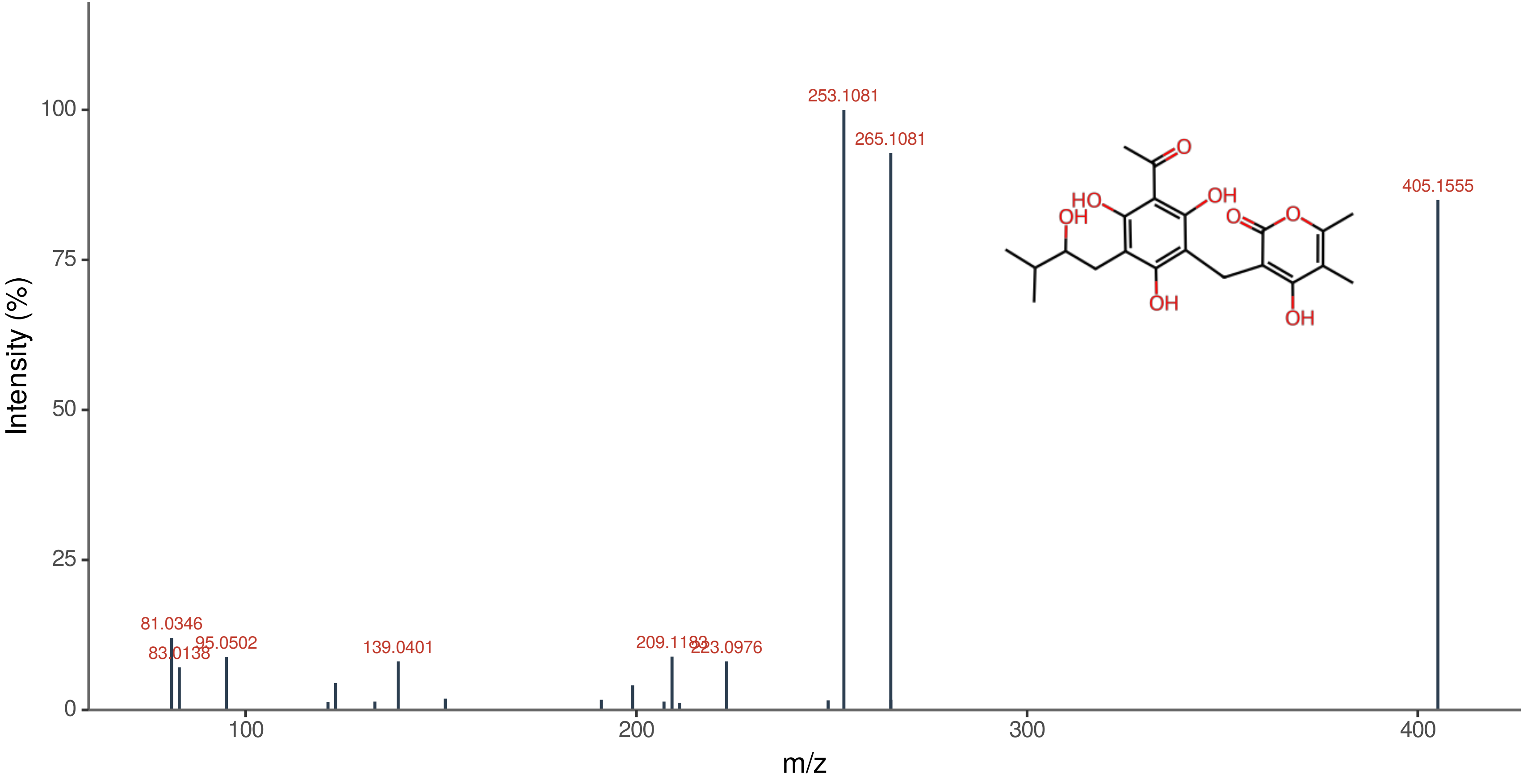

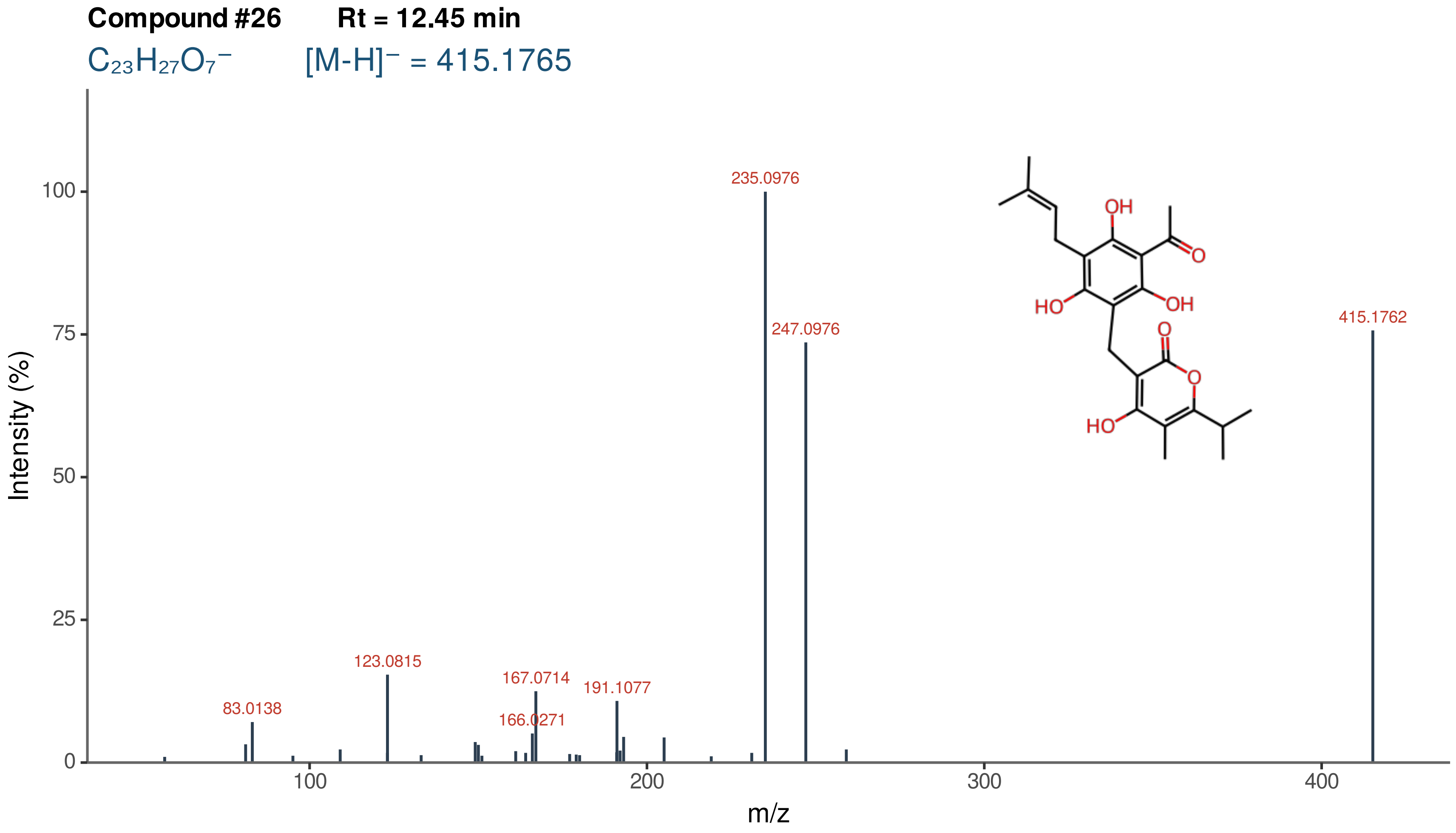

Compound #26
Rt = 12.45 min
$C_{23}H_{27}O_7^-$
$[M-H]^-$ = 415.1765
235.0976
247.0976
415.1762
123.0815
167.0714
191.1077
83.0138
166.0271
OH
O
HO
OH
O
O
HO
Intensity (%)
m/z
100
75
50
25
0
100
200
300
400

Compound #27   **Rt = 13.45 min**

$C_{23}H_{27}O_7^-$ $[M-H]^- = 415.1765$

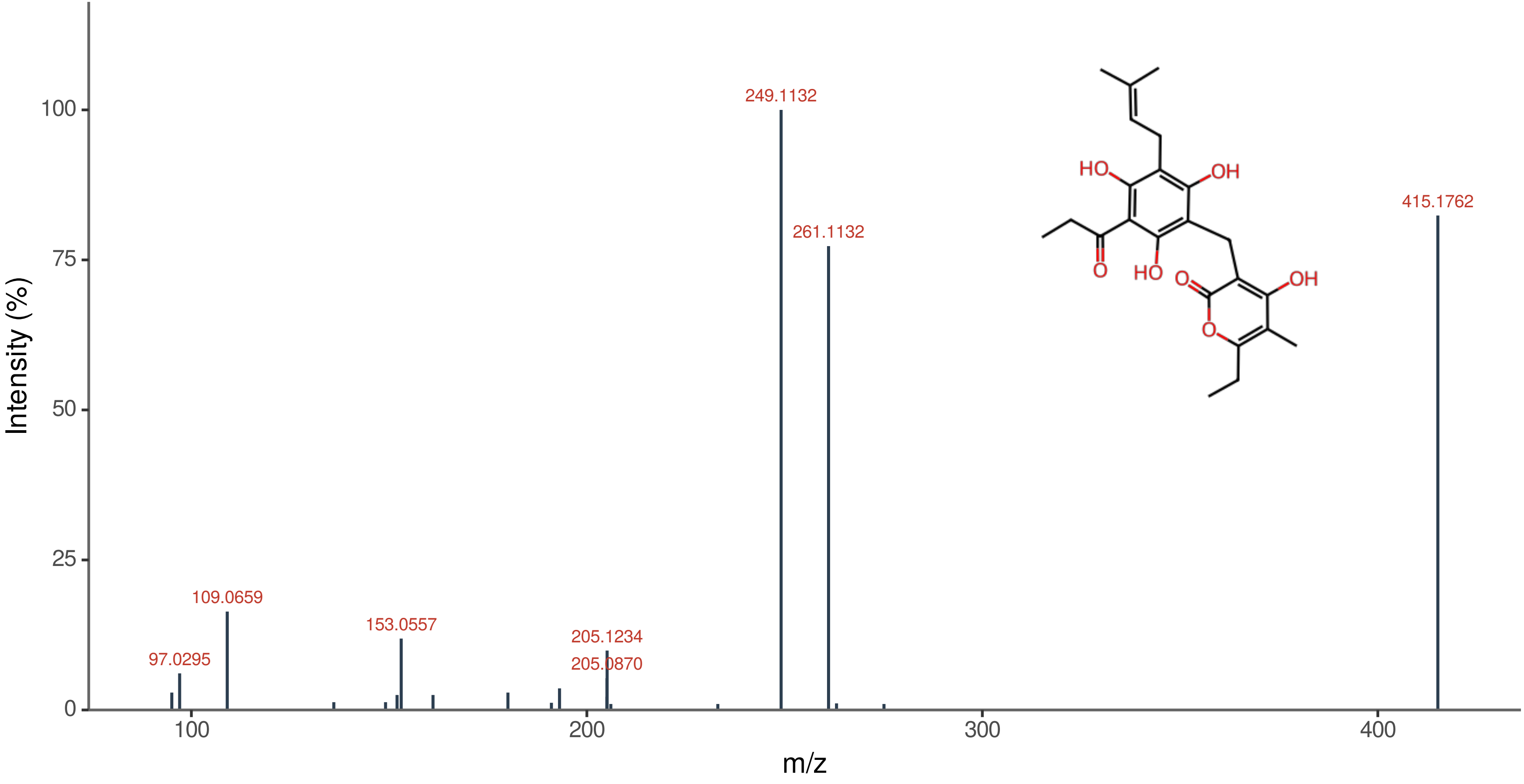

Compound #28 Rt = 13.13 min

$C_{23}H_{27}O_7^-$ $[M-H]^- = 415.1765$

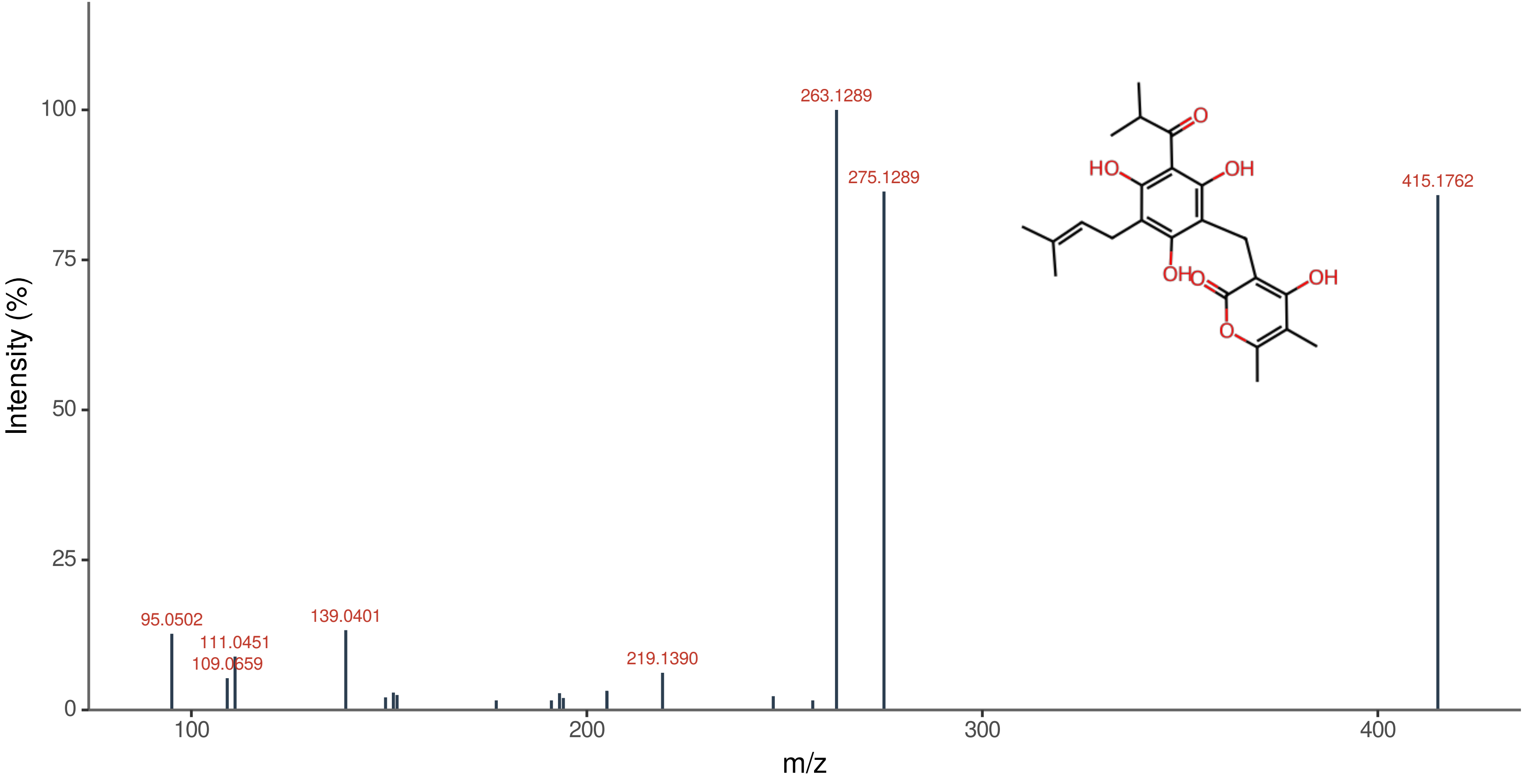

Compound #29 Rt = 12.42 min

$C_{22}H_{25}O_8^-$ $[M-H]^- = 417.1558$

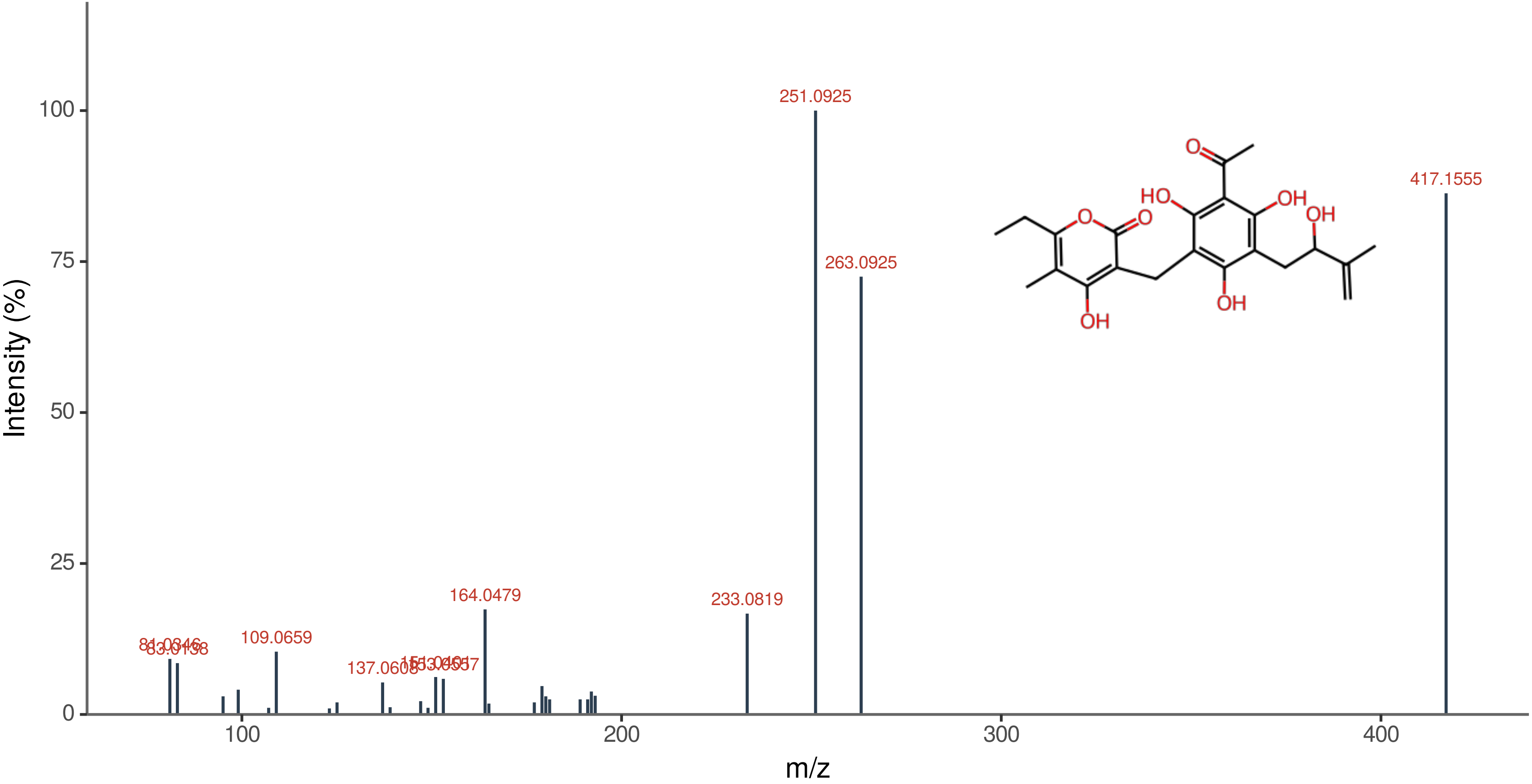

Compound #30 Rt = 6.30 min

$C_{22}H_{25}O_8^-$ $[M-H]^-$ = 417.1557

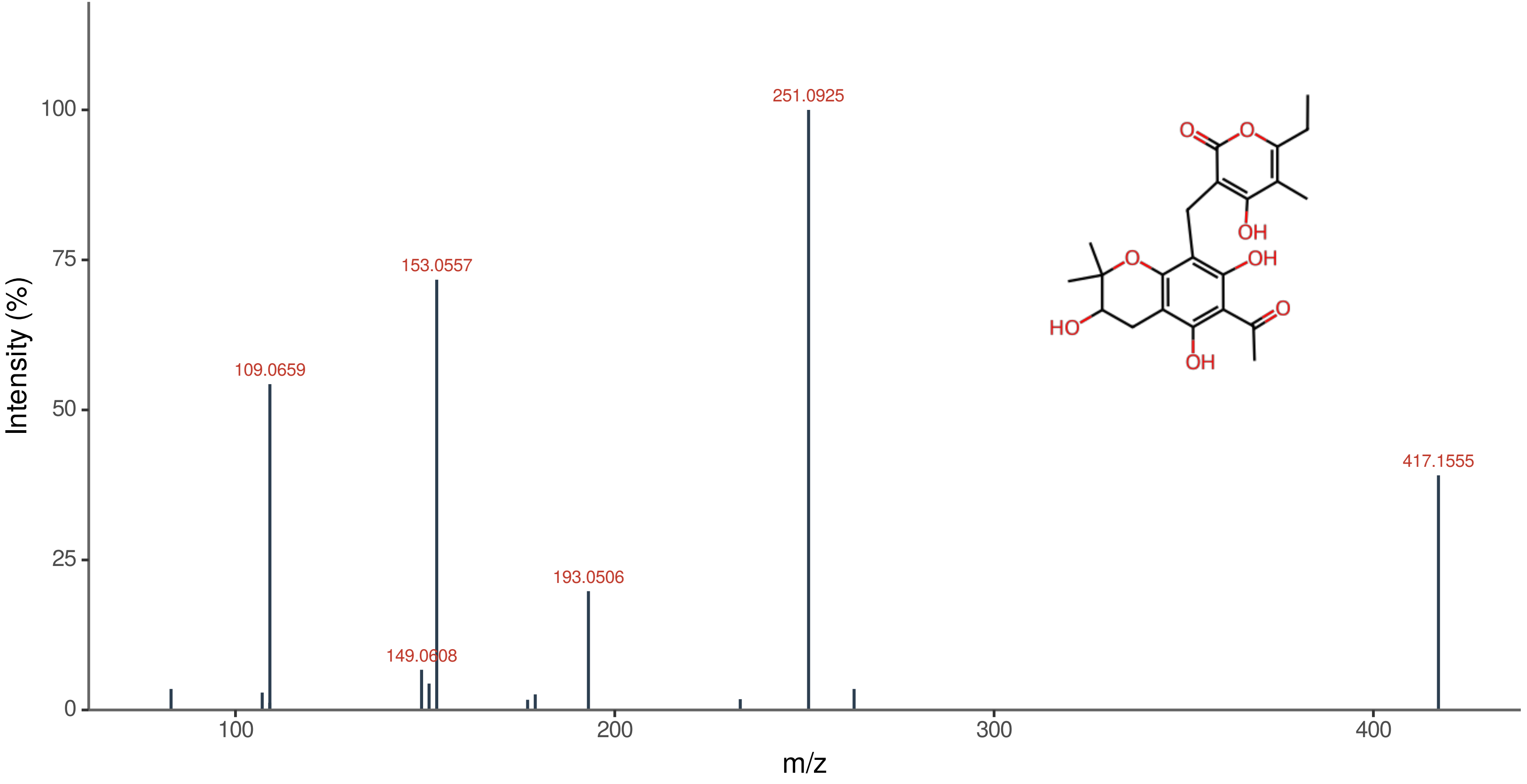

Compound #31 **Rt = 8.87 min**

$C_{22}H_{25}O_8^-$ $[M-H]^- = 417.1557$

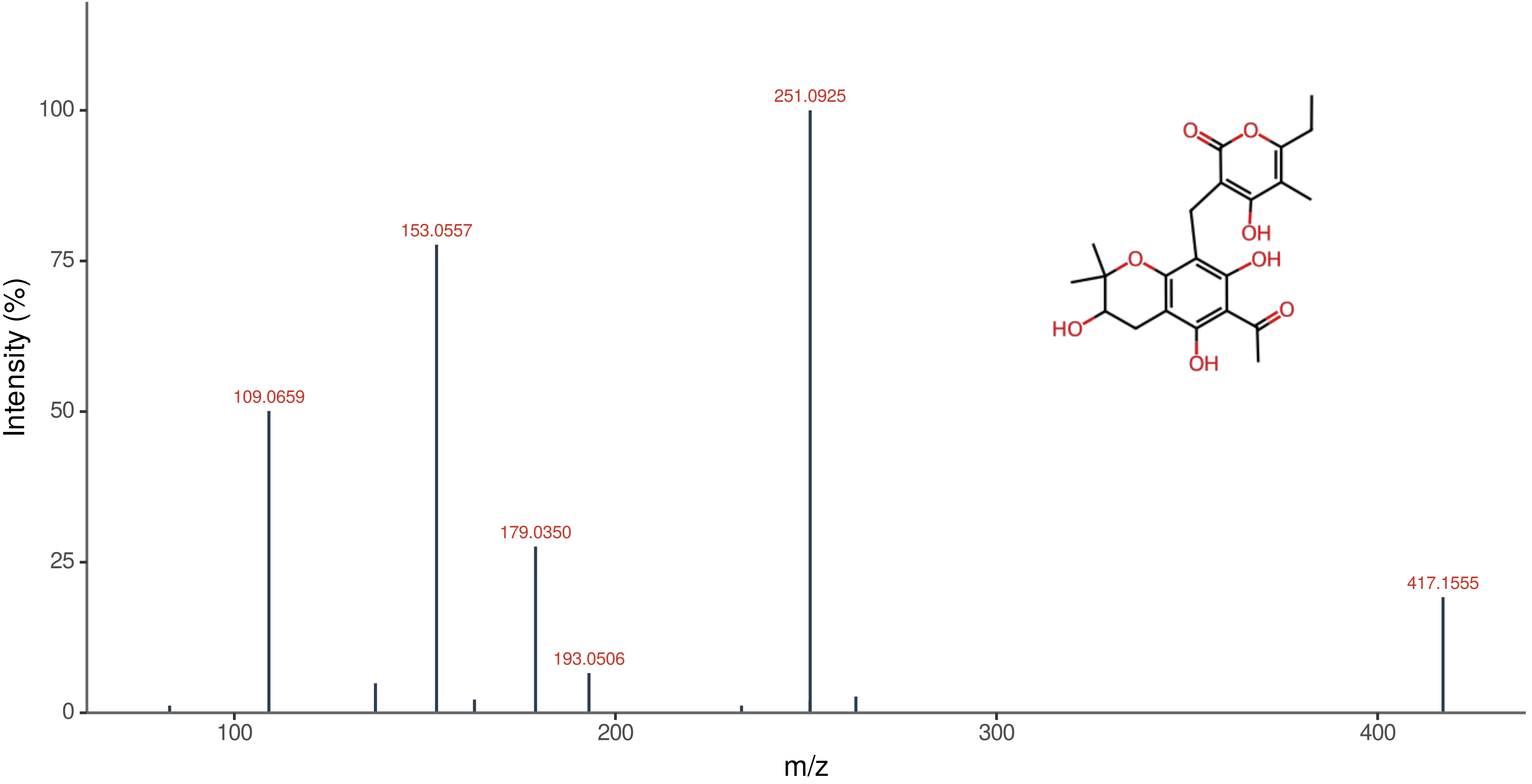

Compound #32 Rt = 7.22 min

$C_{22}H_{27}O_8^-$ $[M-H]^- = 419.1715$

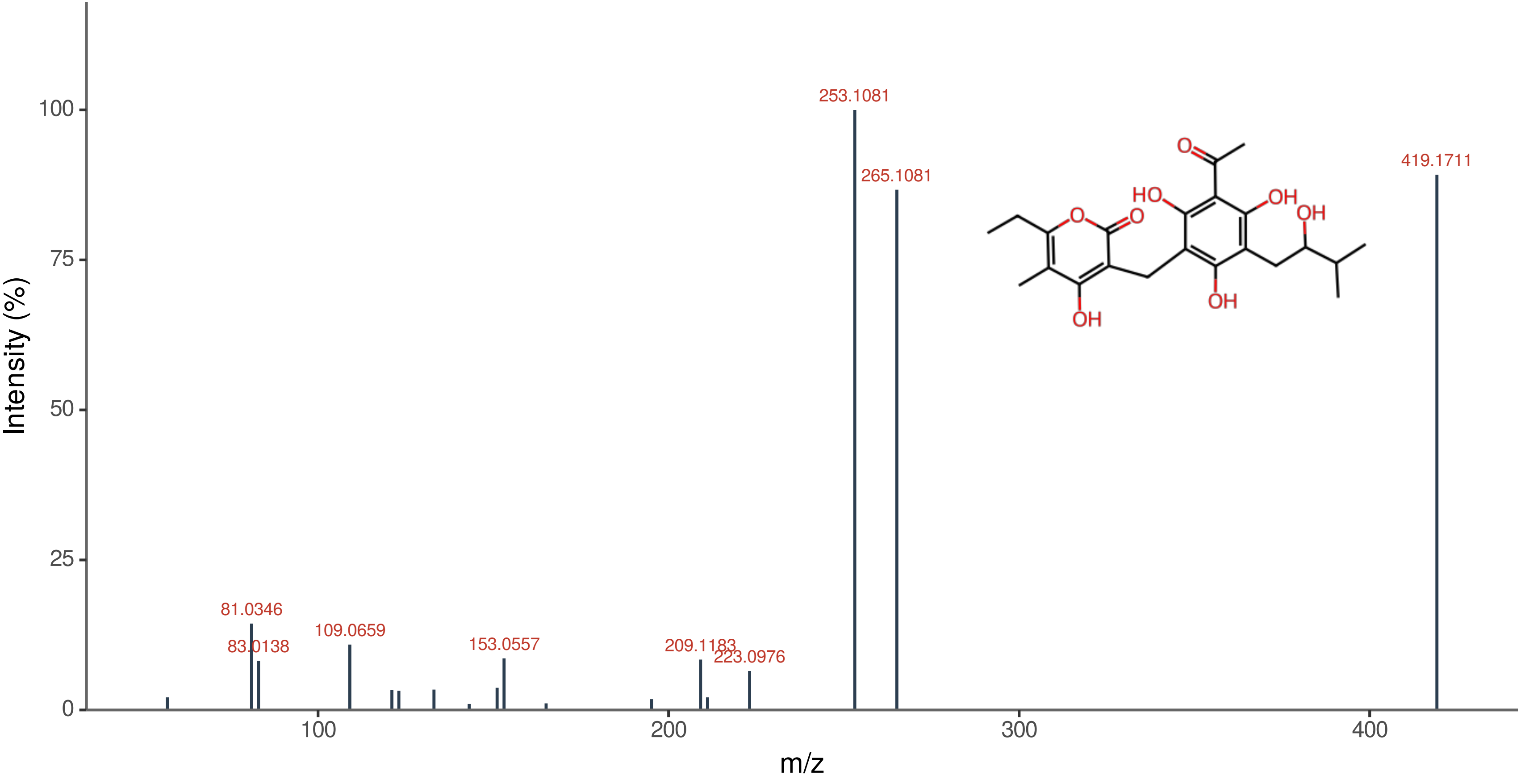

Compound #33 Rt = 16.76 min

$C_{24}H_{27}O_7^-$ $[M-H]^- = 427.1760$

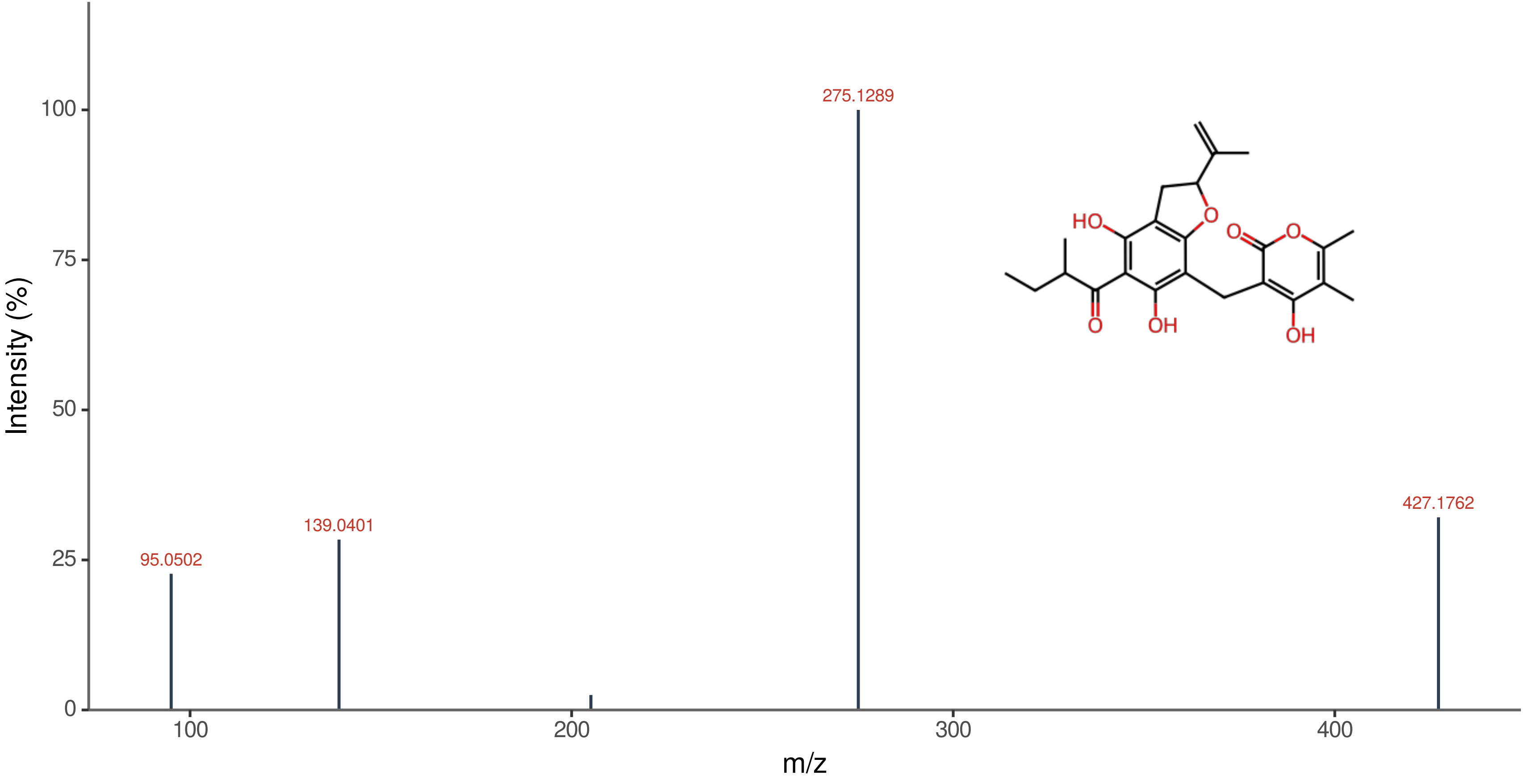

Compound #34 **Rt = 17.86 min**

$C_{24}H_{27}O_7^-$ $[M-H]^- = 427.1761$

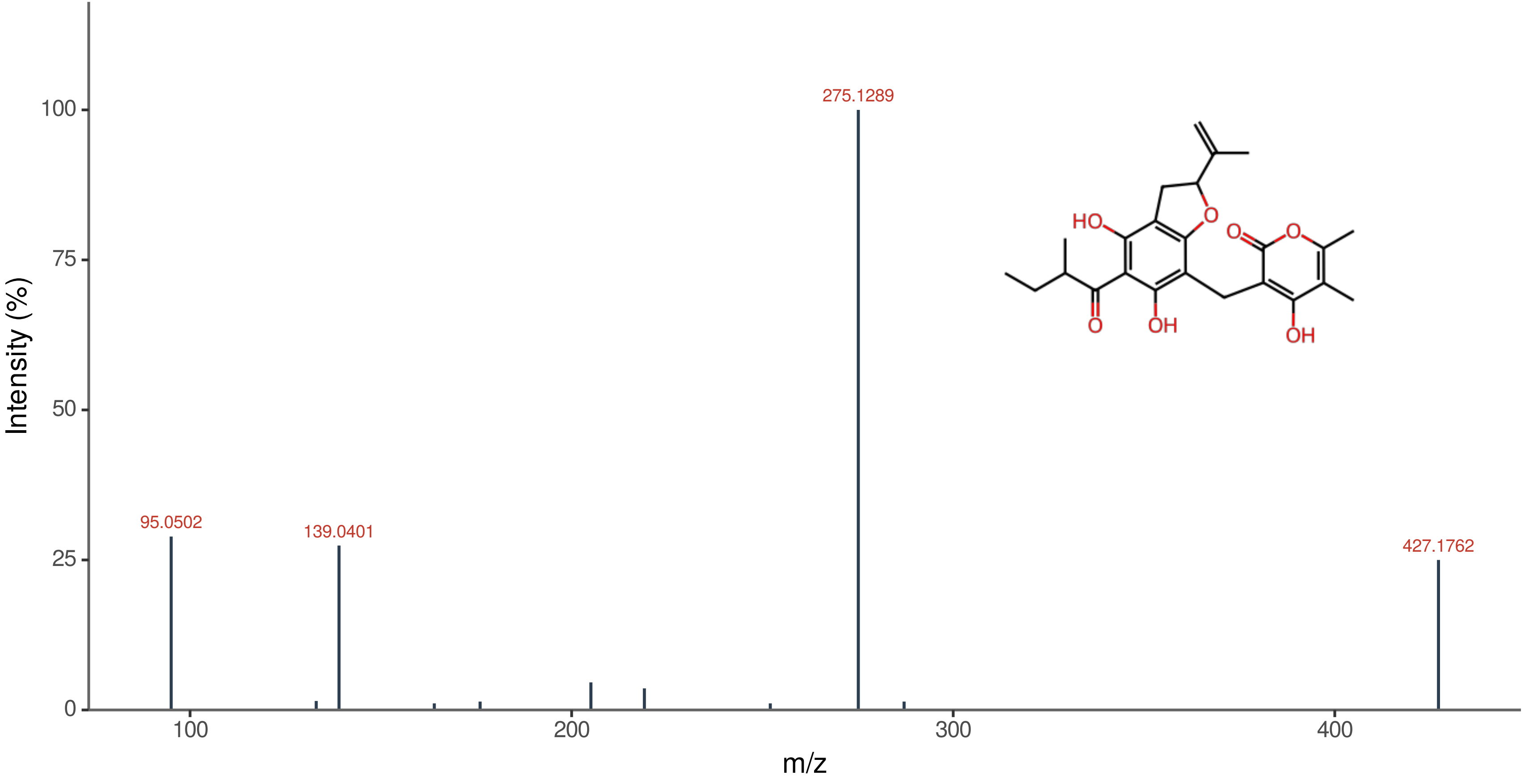

Compound #35 **Rt = 15.00 min**

$C_{24}H_{29}O_7^-$ $[M\text{-}H]^- = 429.1917$

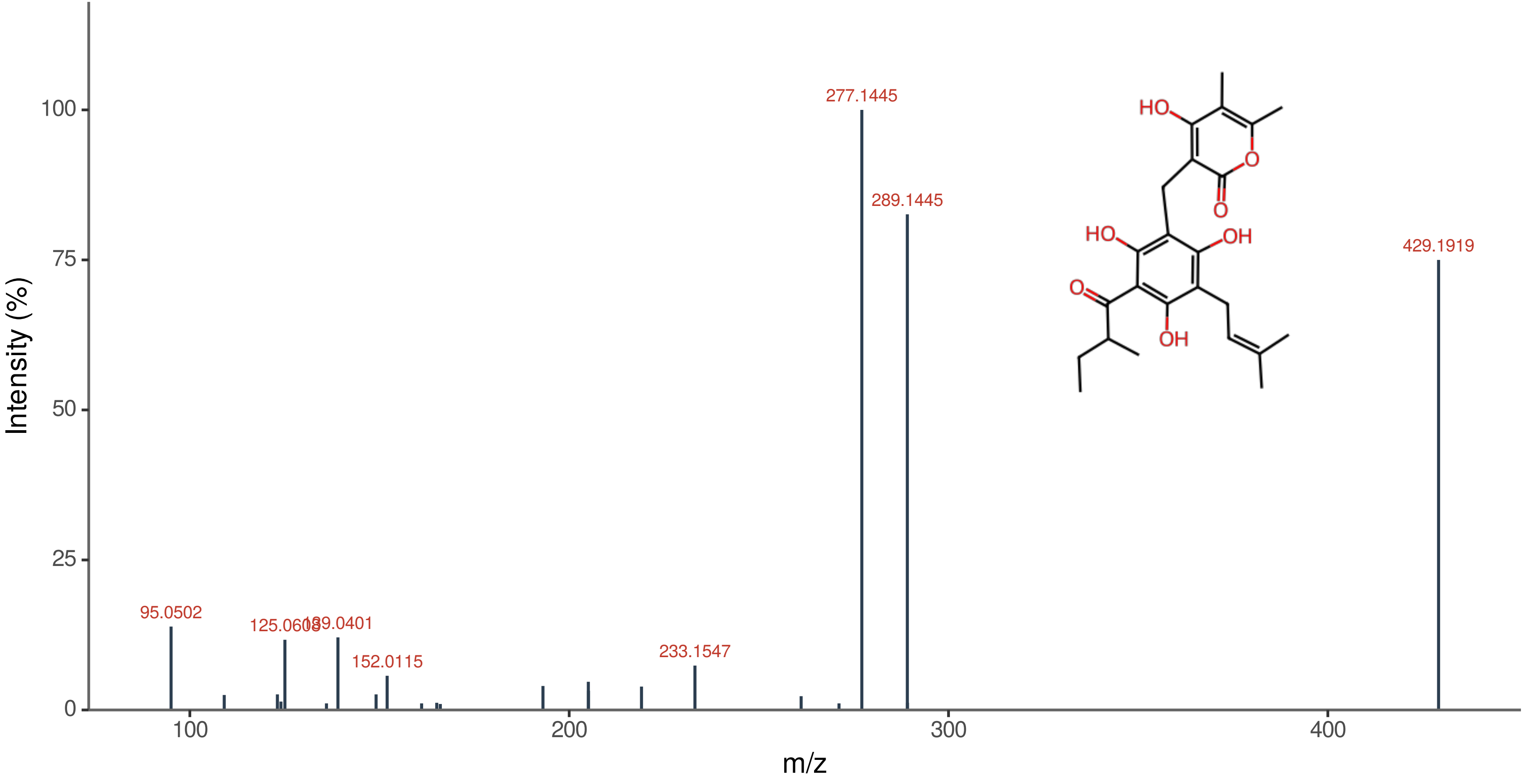

Compound #36 **Rt = 15.40 min**

$C_{24}H_{29}O_7^-$ $[M-H]^- = 429.1918$

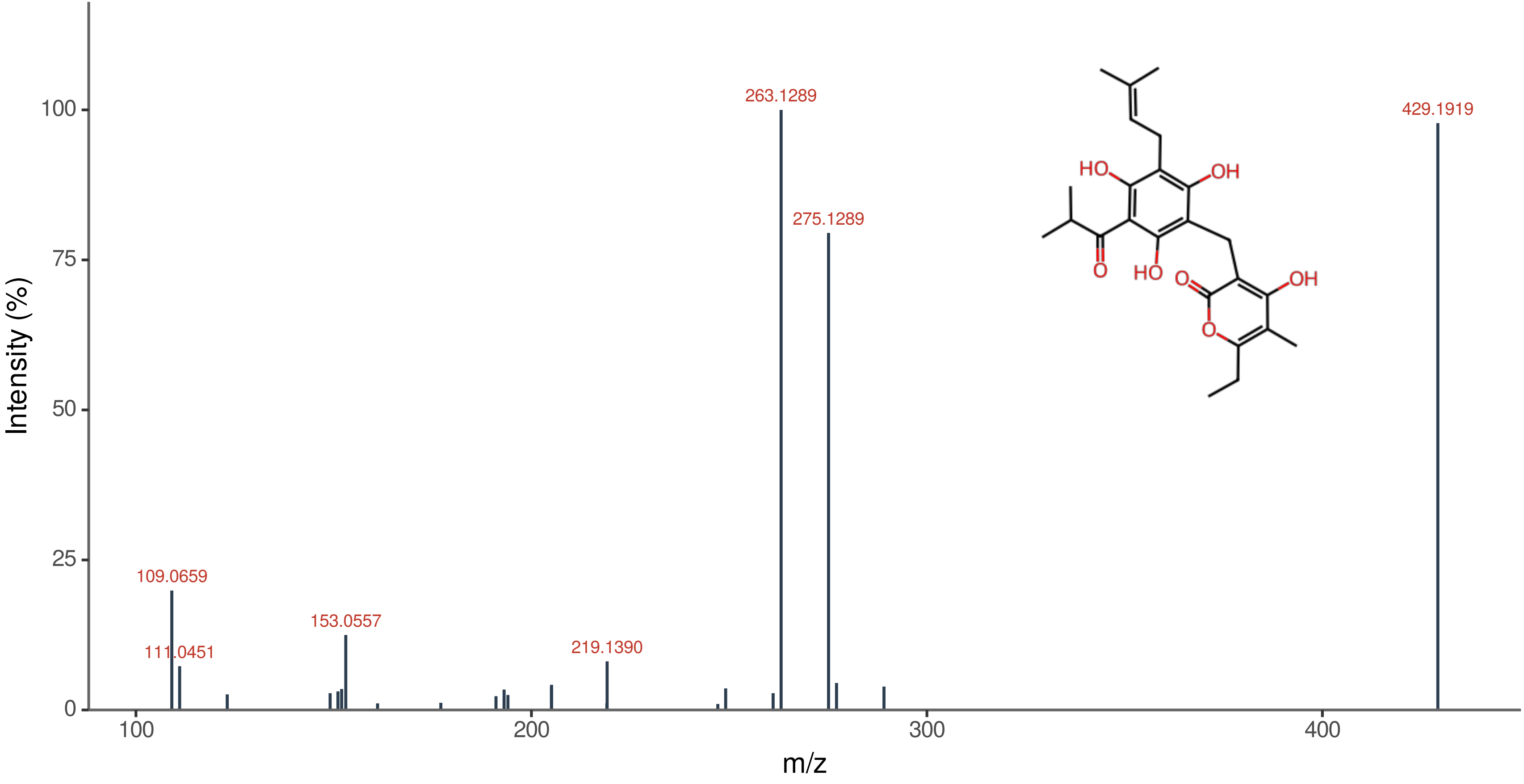

Compound #37   Rt = 18.35 min

$C_{26}H_{31}O_6^-$ $[M\text{-}H]^- = 439.2125$

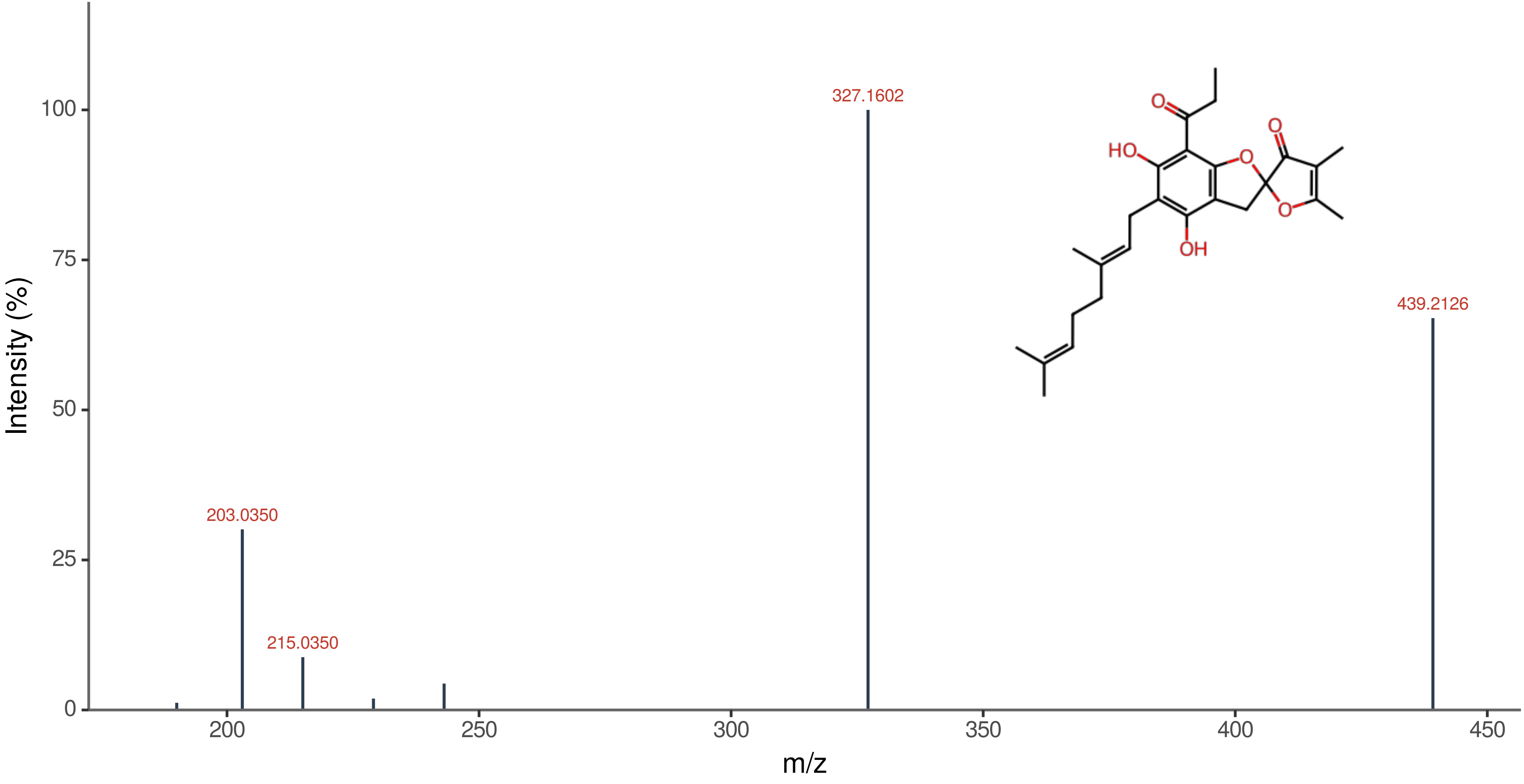

Compound #38 **Rt = 18.98 min**

$C_{25}H_{29}O_7^-$ $[M-H]^- = 441.1919$

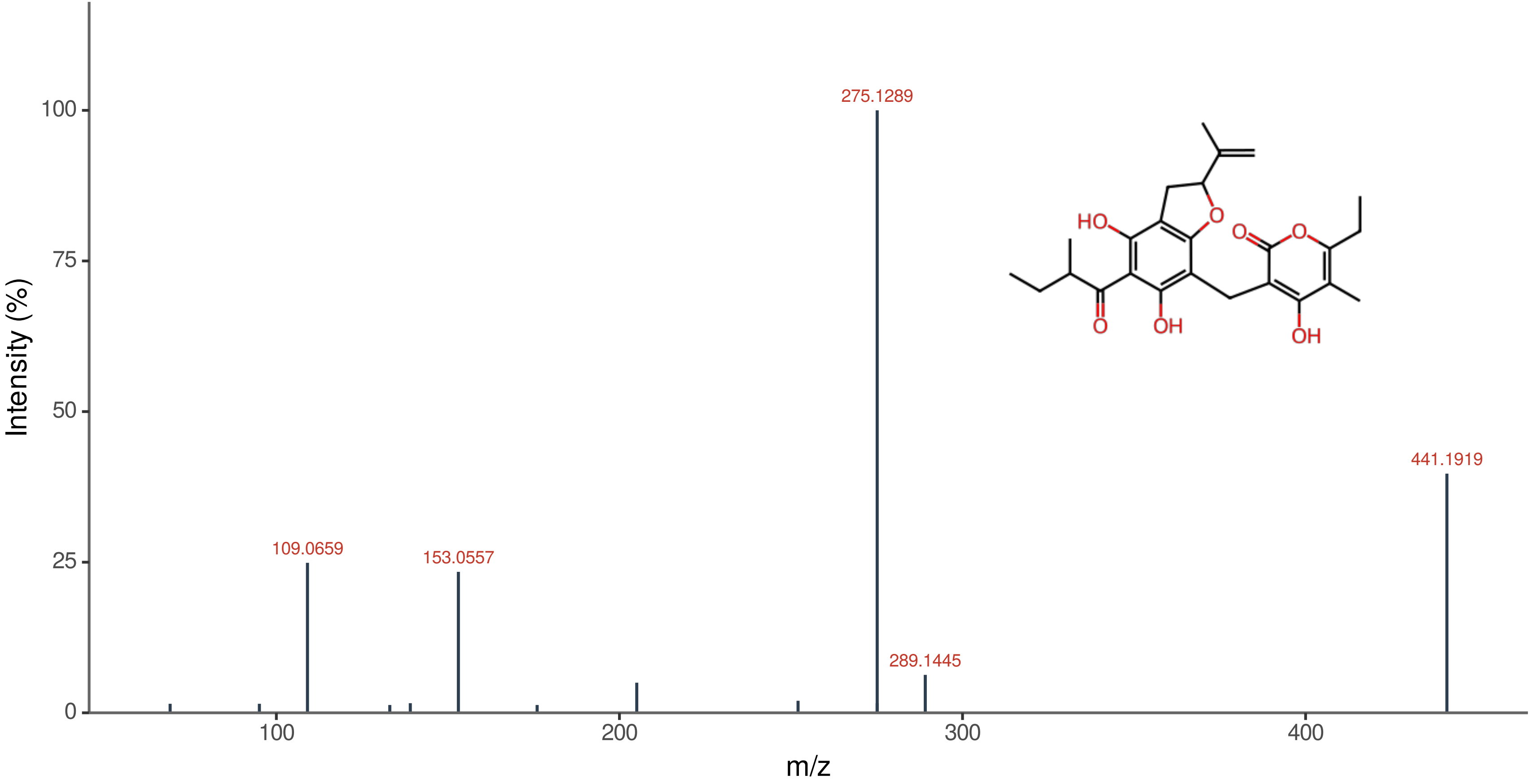

Compound #39 Rt = 18.98 min

$C_{25}H_{29}O_7^-$ $[M-H]^- = 441.1919$

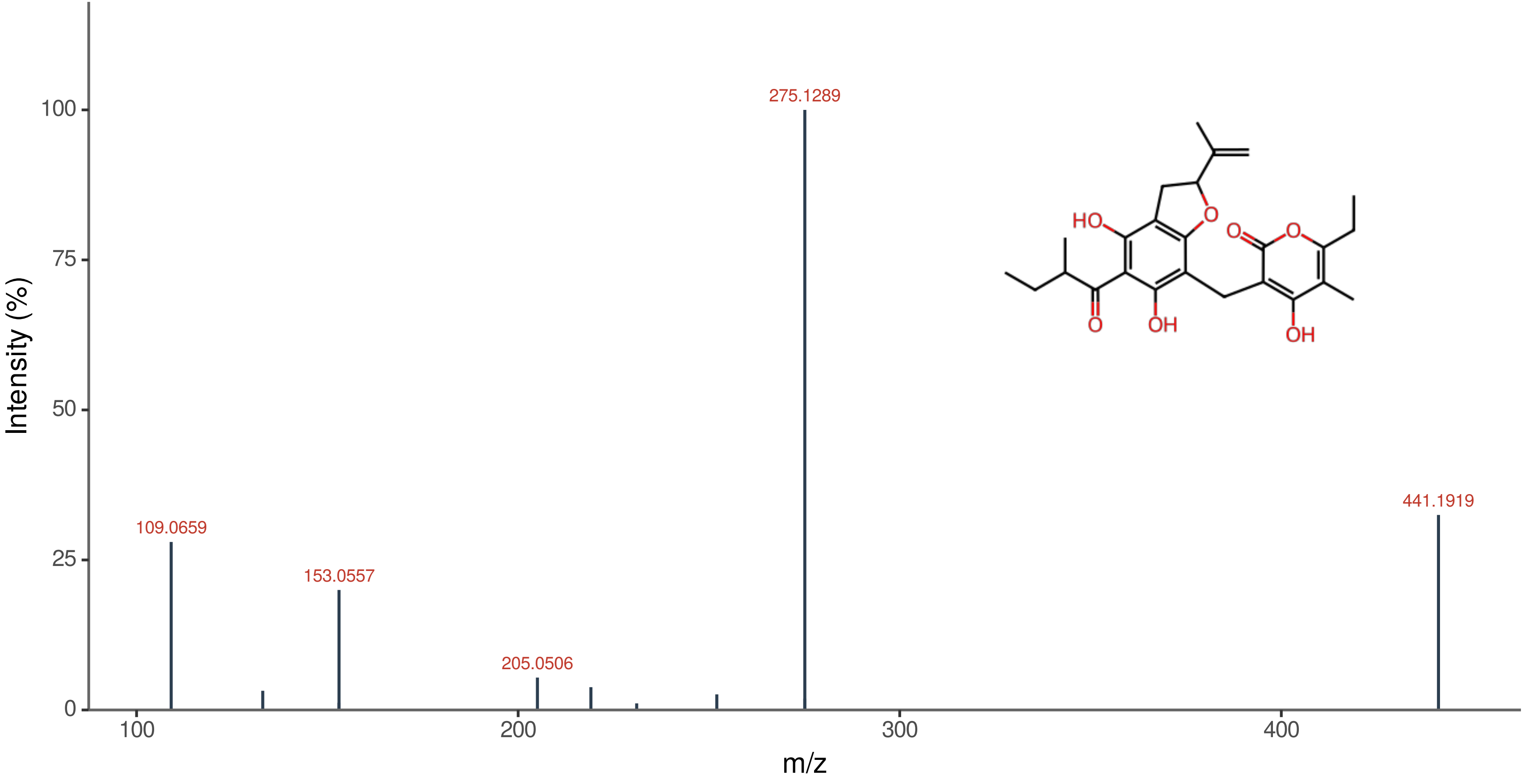

Compound #40 Rt = 17.26 min

$C_{25}H_{31}O_7^-$ $[M-H]^-$ = 443.2075

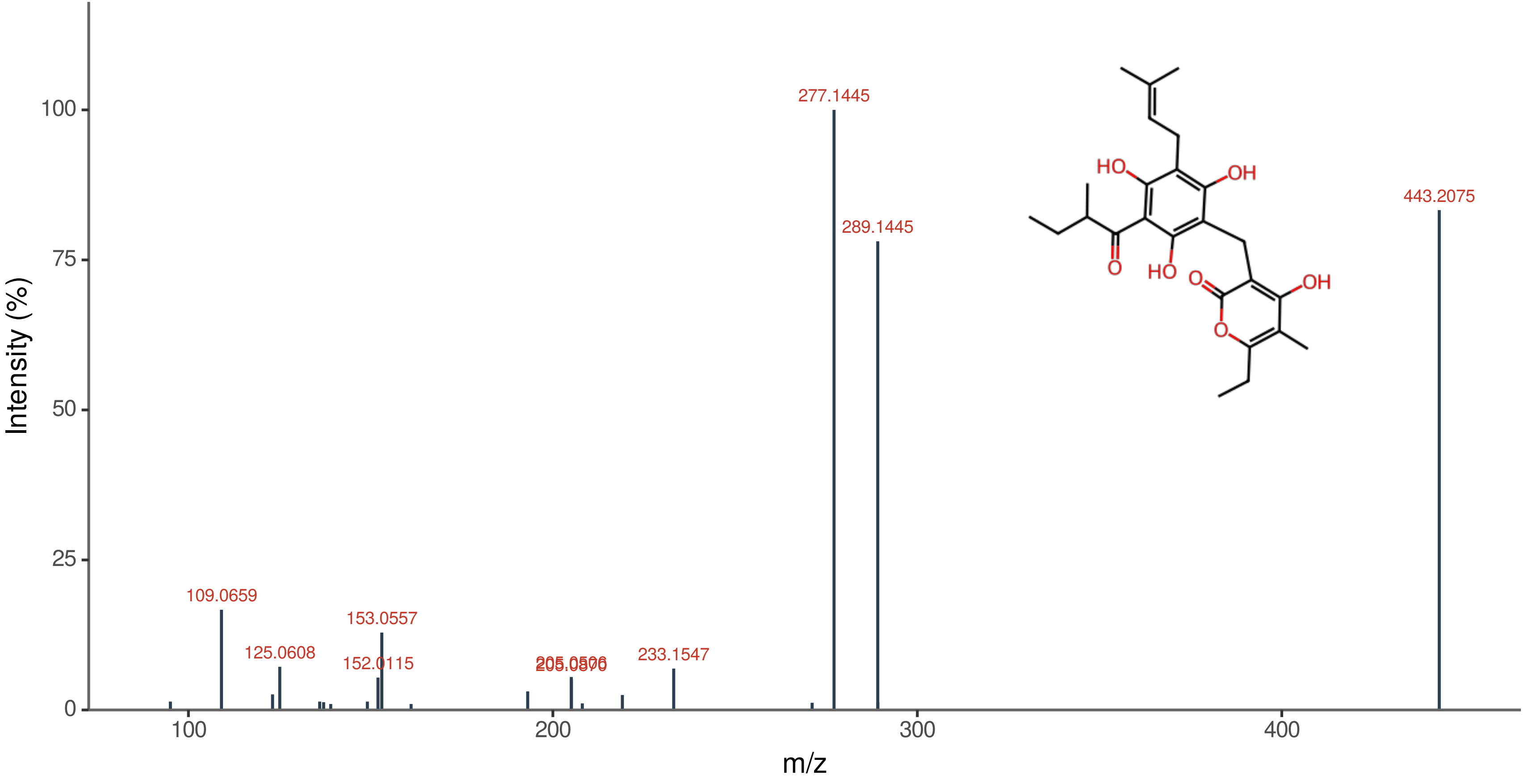

Compound #41 **Rt = 17.95 min**

$C_{25}H_{31}O_7^-$ $[M-H]^- = 443.2075$

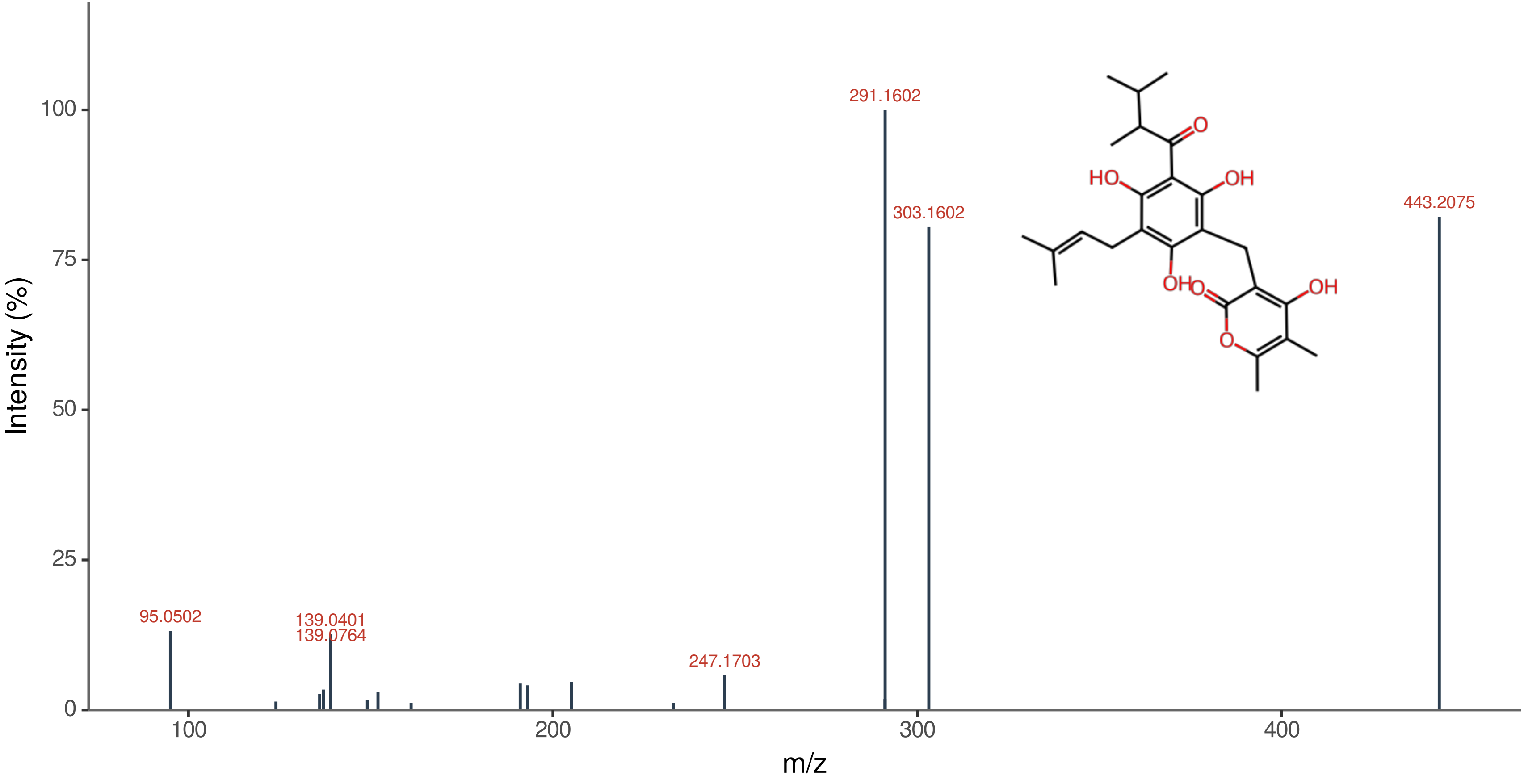

Compound #42 Rt = 16.20 min

$C_{26}H_{31}O_7^-$ $[M-H]^- = 455.2079$

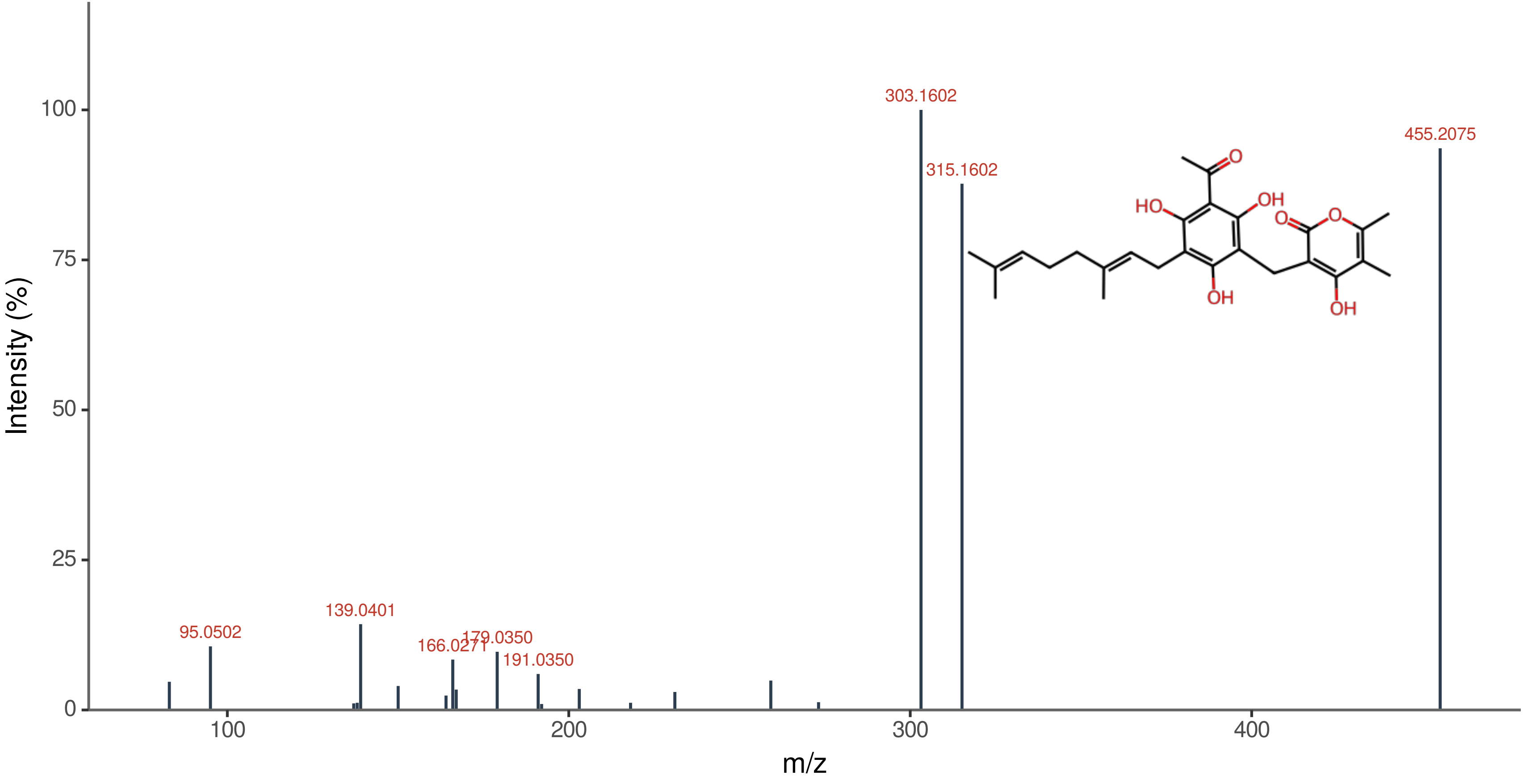

Compound #43 **Rt = 19.71 min**

$C_{26}H_{33}O_7^-$ $[M-H]^- = 457.2232$

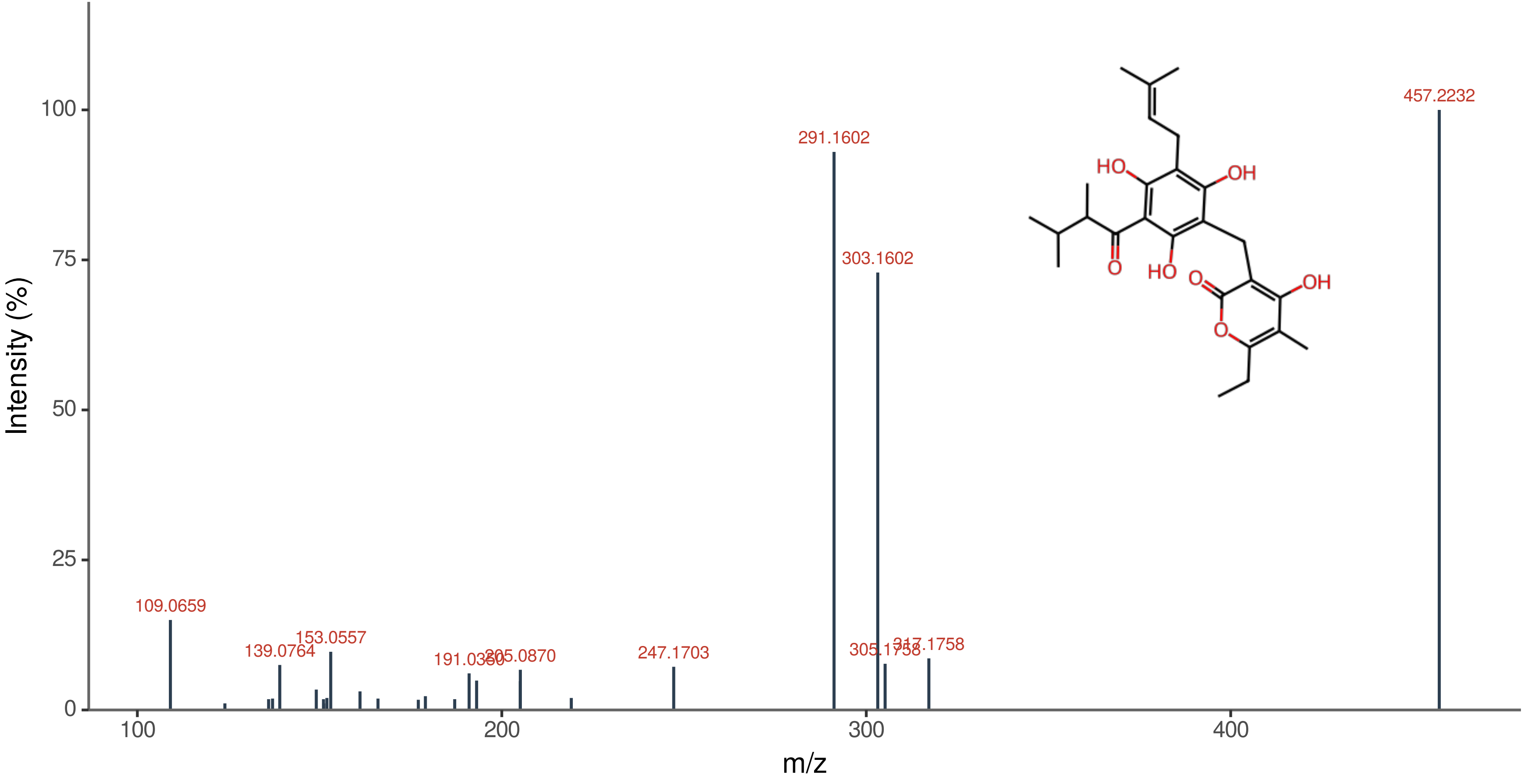

Compound #44 **Rt = 20.15 min**

$C_{26}H_{33}O_7^-$ $[M-H]^- = 457.2235$

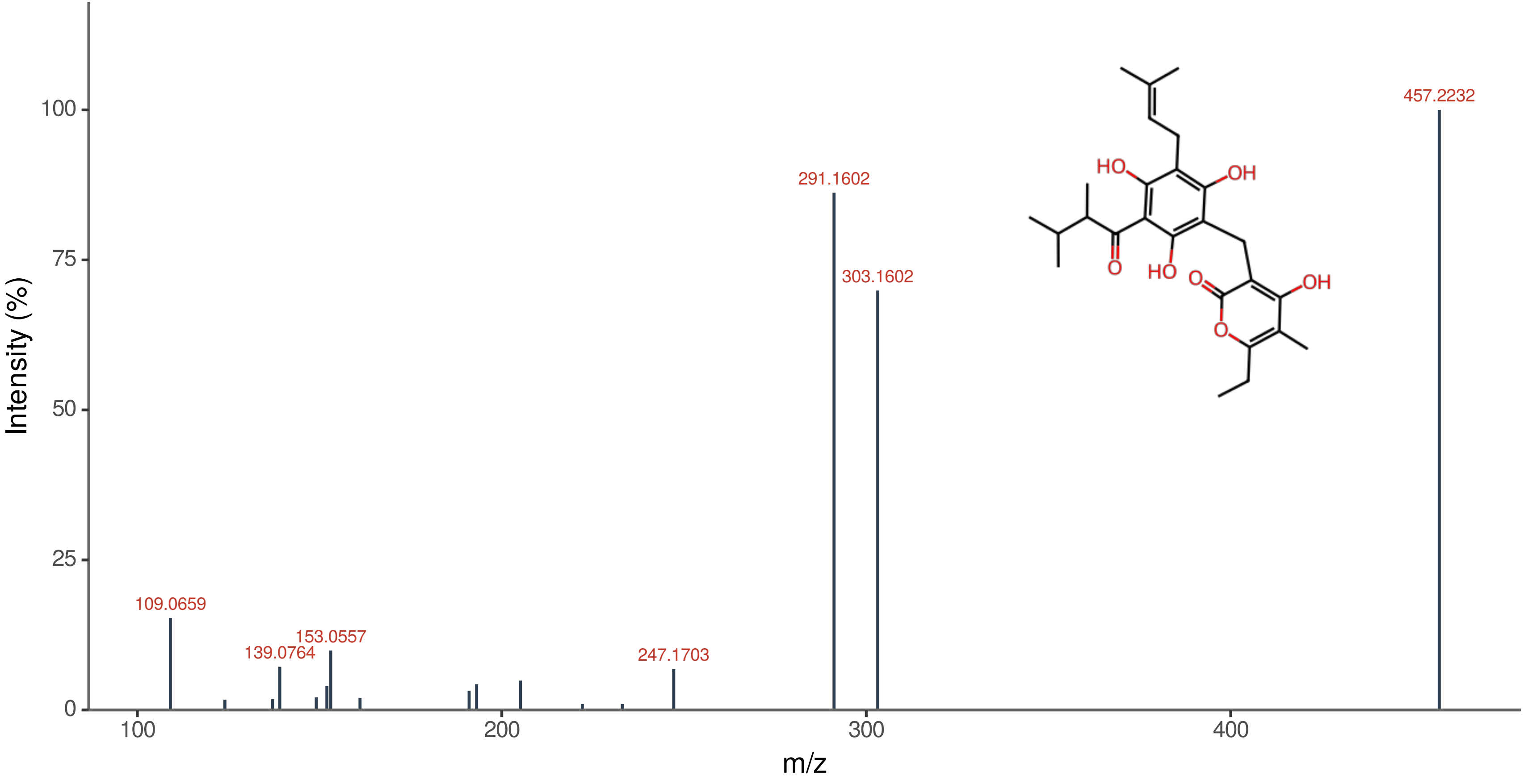

Compound #45 Rt = 12.37 min

$C_{25}H_{31}O_8^-$ $[M-H]^- = 459.2028$

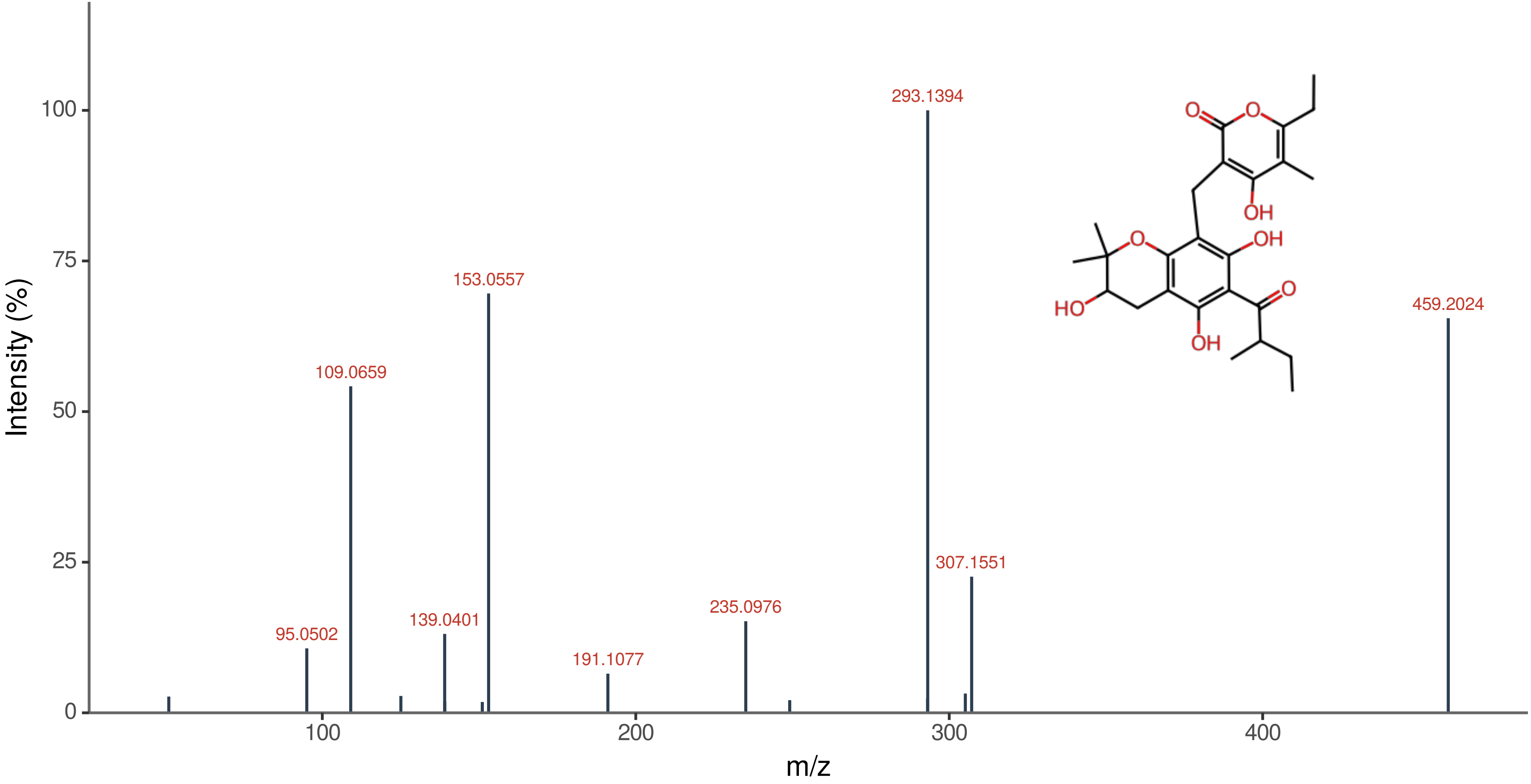

Compound #46   Rt = 15.18 min

$C_{27}H_{31}O_7^-$ $[M-H]^- = 467.2074$

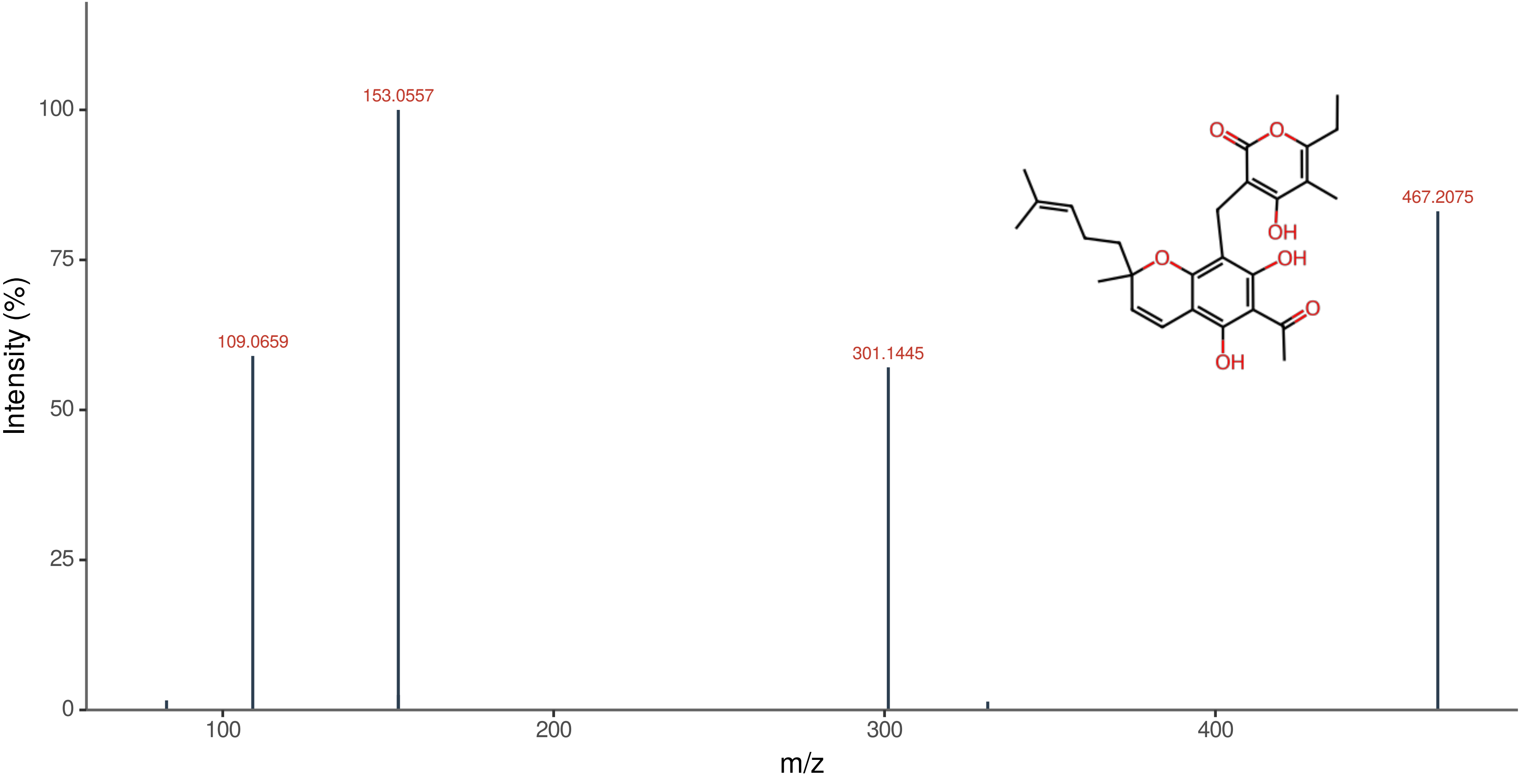

Compound #47　　**Rt = 18.34 min**

$C_{27}H_{33}O_7^-$　　$[M-H]^- = 469.2232$

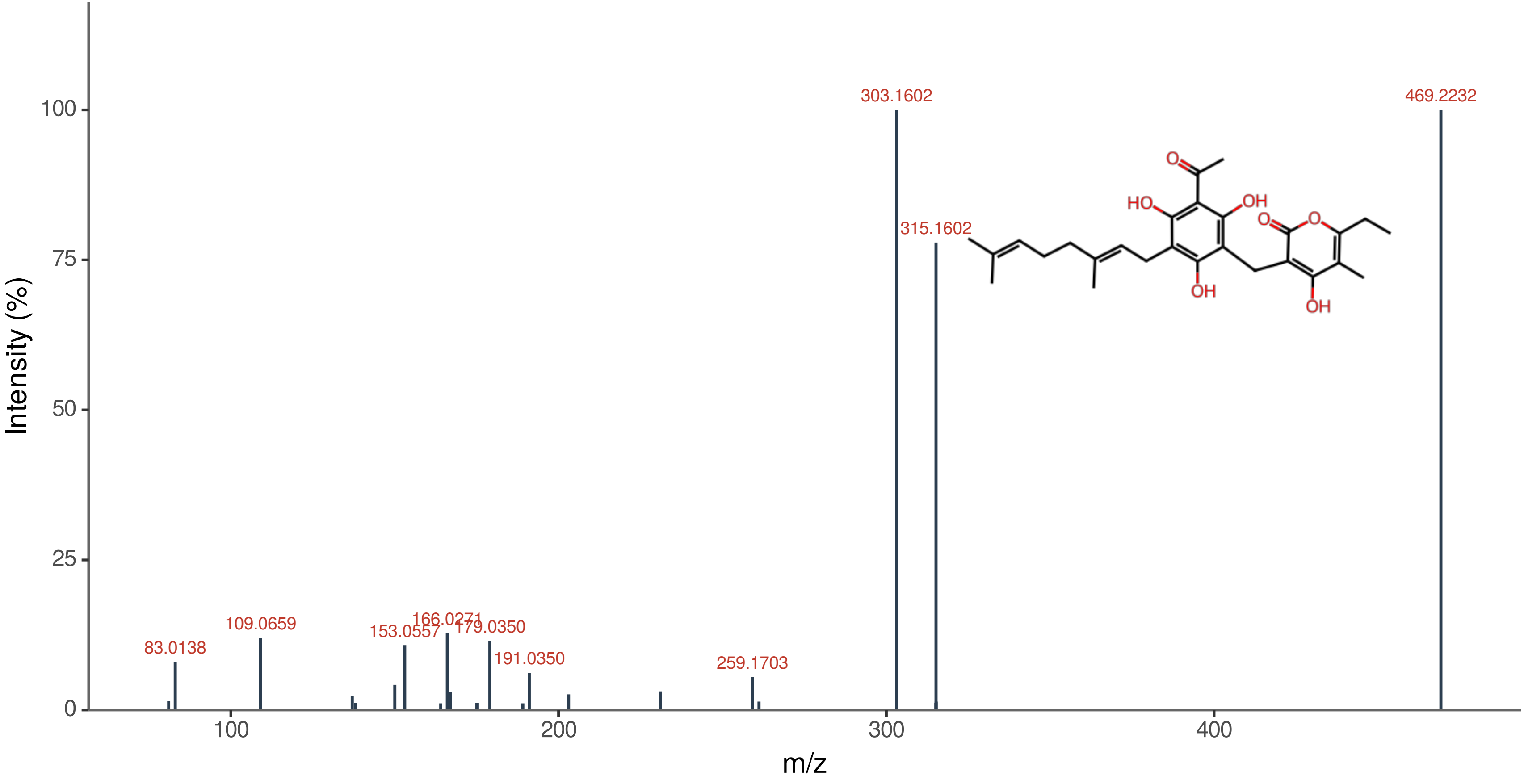

Compound #48 **Rt = 7.71 min**

$C_{26}H_{31}O_8^-$ $[M-H]^- = 471.2025$

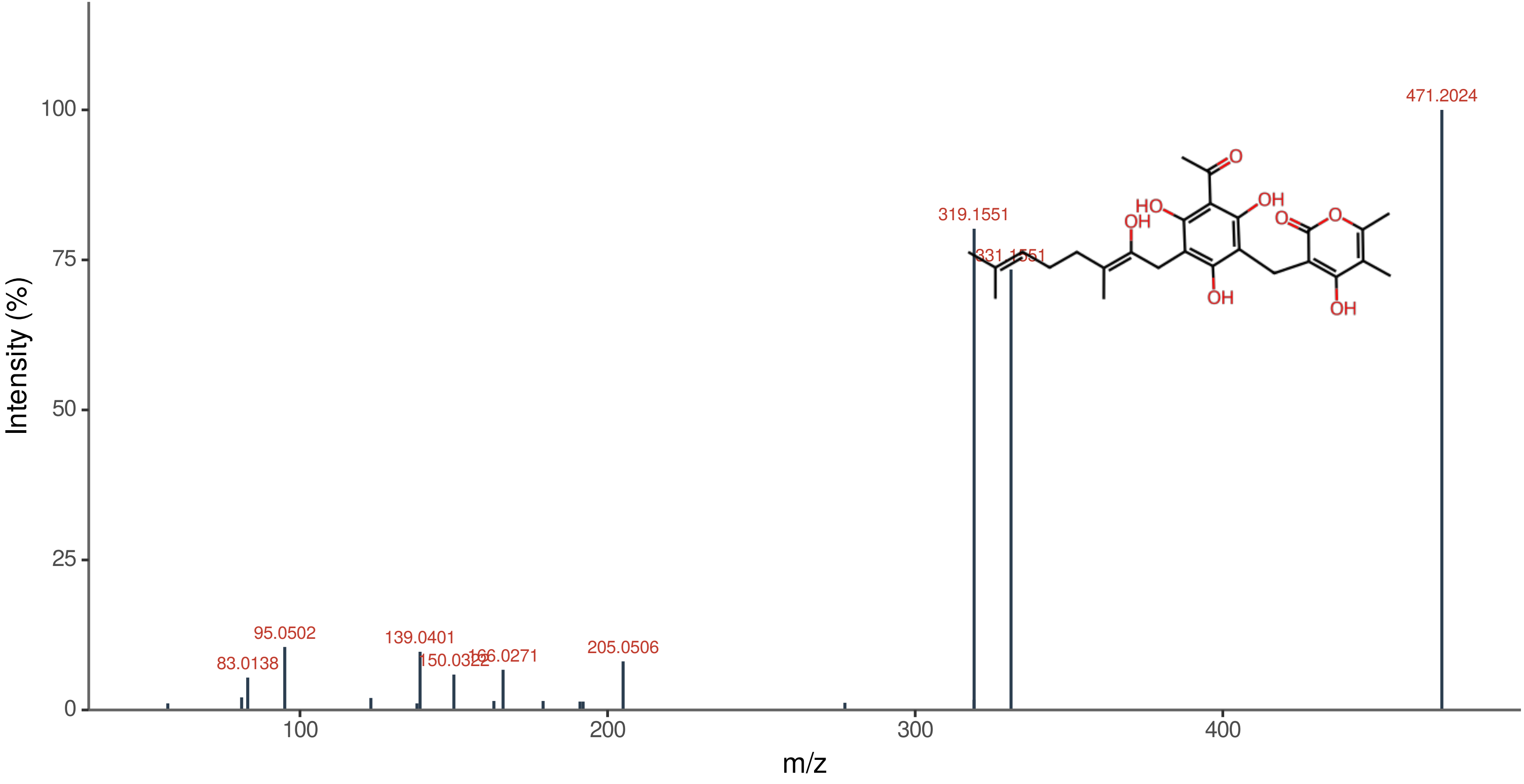

Compound #49 **Rt = 11.56 min**

$C_{26}H_{31}O_8^-$ $[M-H]^- = 471.2027$

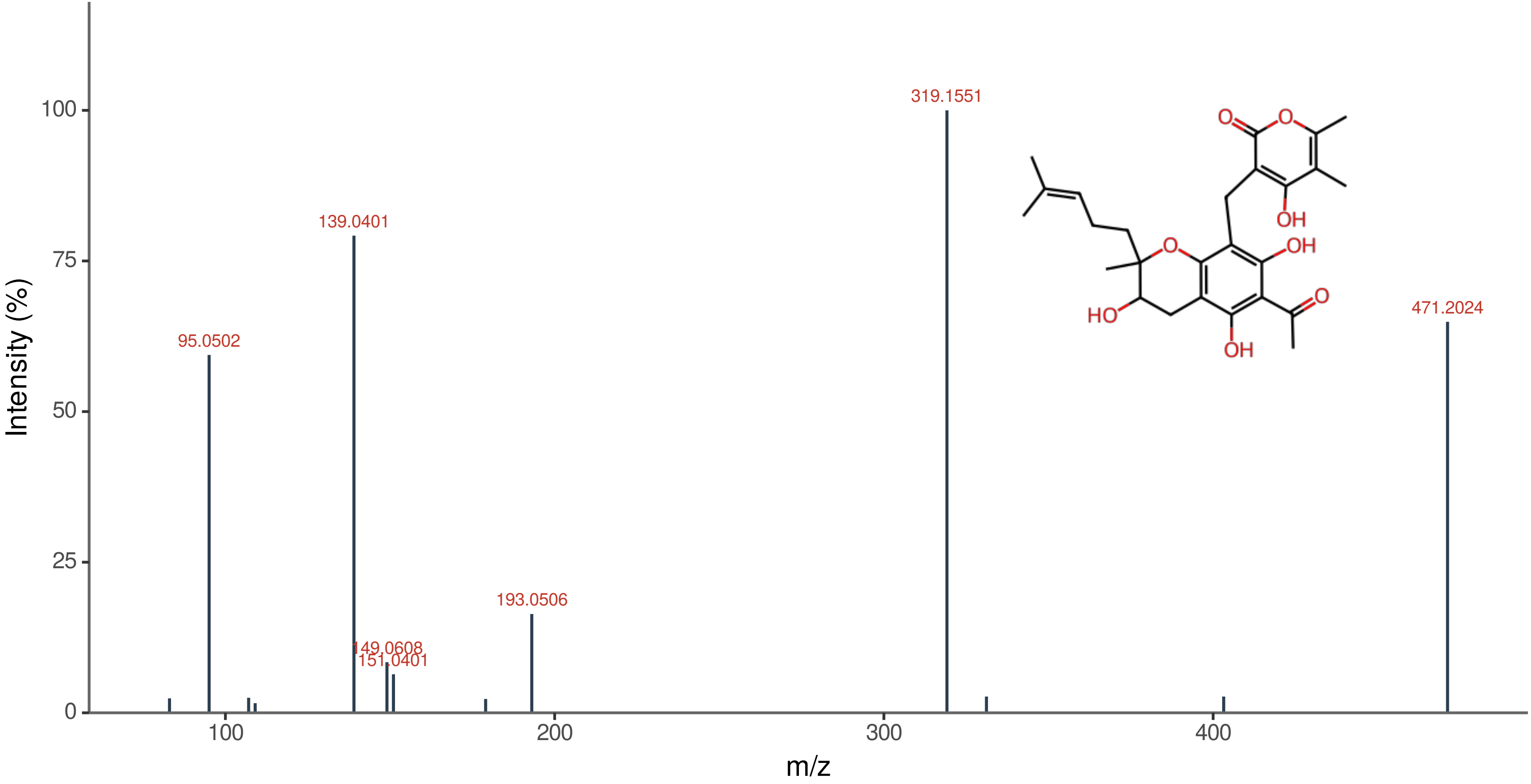

Compound #50 Rt = 20.40 min

$C_{28}H_{35}O_7^-$ $[M\text{-}H]^- = 483.2392$

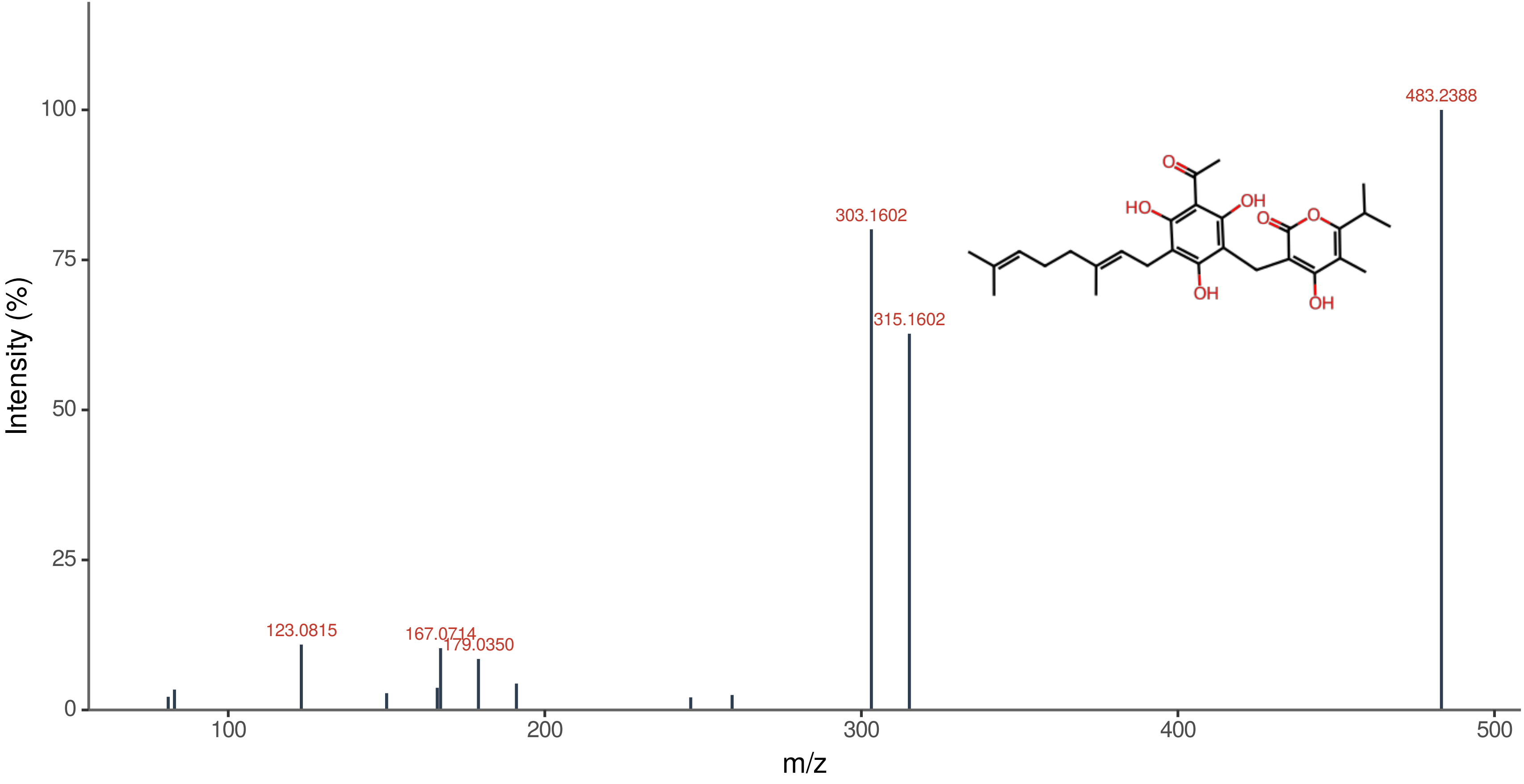

Compound #51　　**Rt = 13.72 min**

$C_{27}H_{33}O_8^-$　　$[M-H]^- = 485.2184$

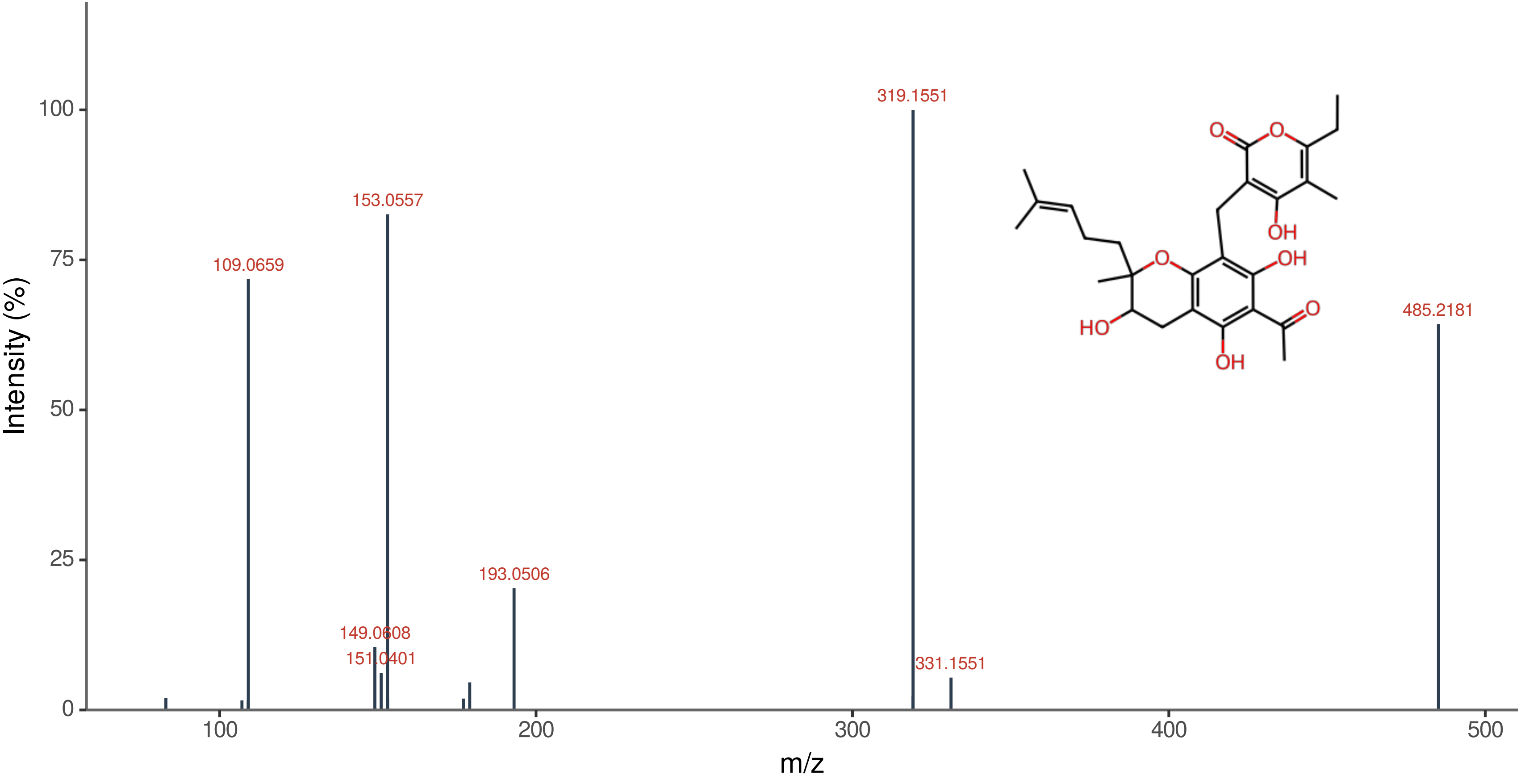

Compound #52 **Rt = 16.38 min**

$C_{27}H_{33}O_8^-$ $[M-H]^- = 485.2179$

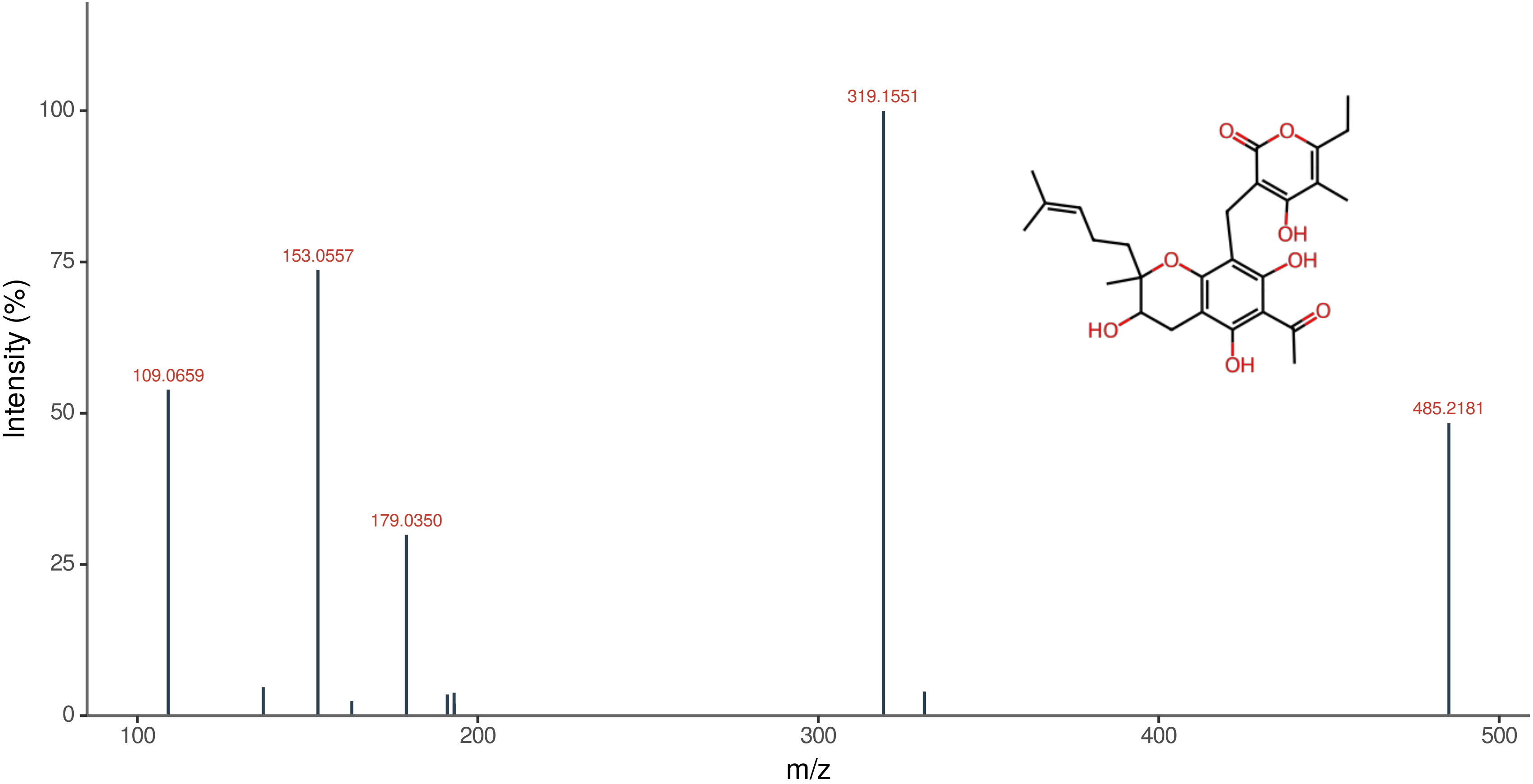

Compound #53 **Rt = 9.50 min**

$C_{27}H_{33}O_8^-$ $[M-H]^- = 485.2185$

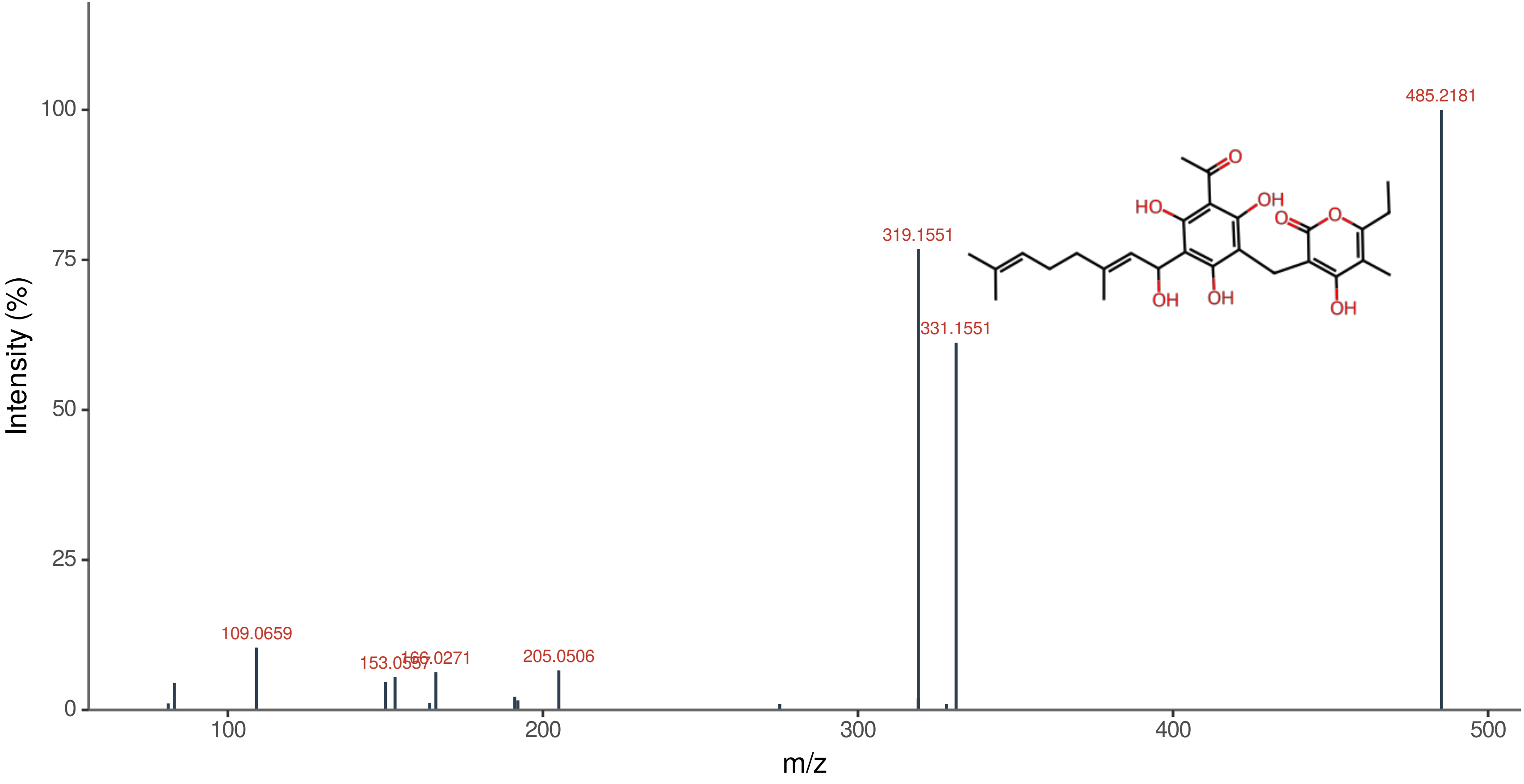

Compound #54 Rt = 20.59 min

$C_{27}H_{33}O_8^-$ $[M-H]^- = 485.2184$

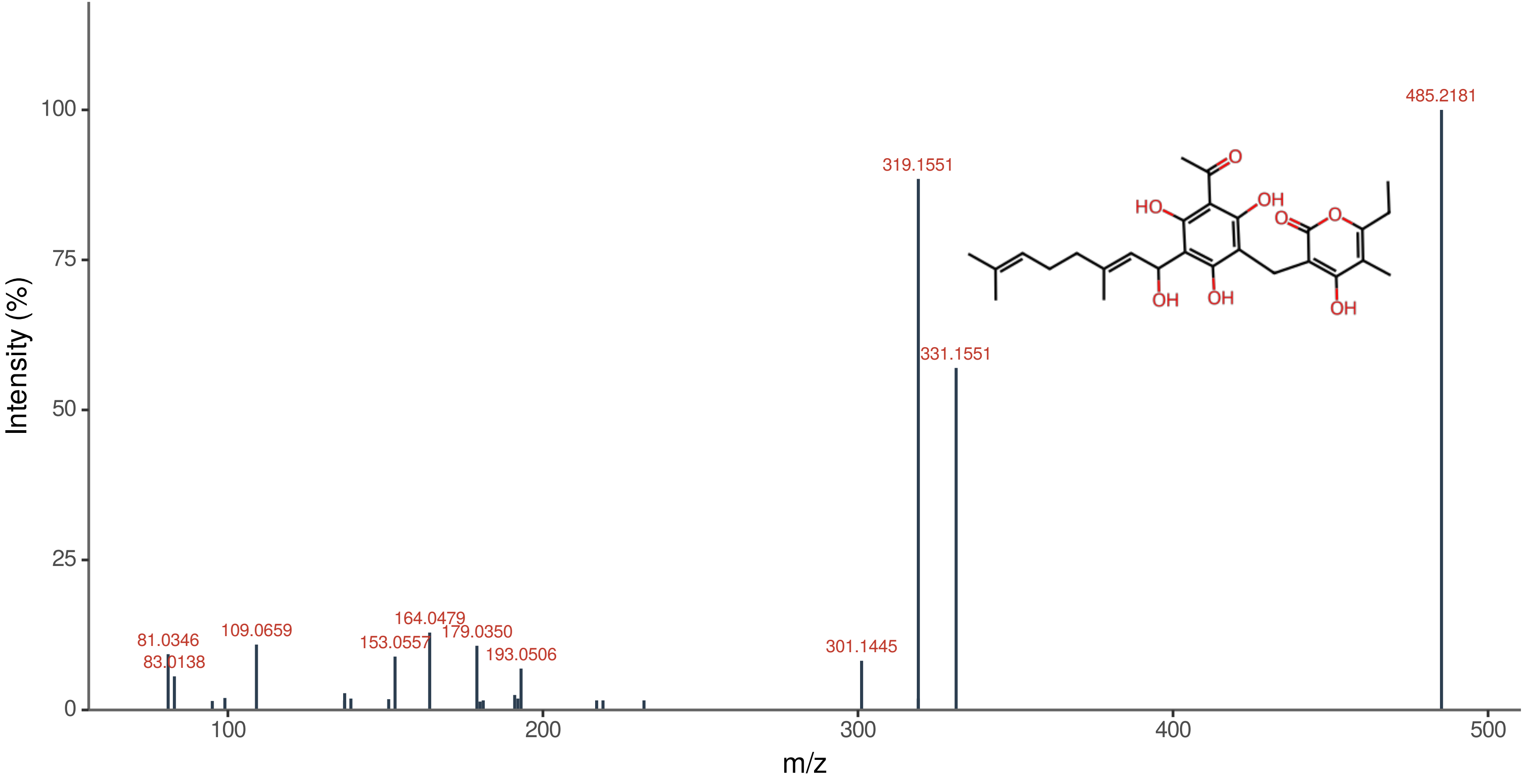

Compound #55 **Rt = 18.46 min**

$C_{29}H_{31}O_7^-$ $[M-H]^- = 491.2077$

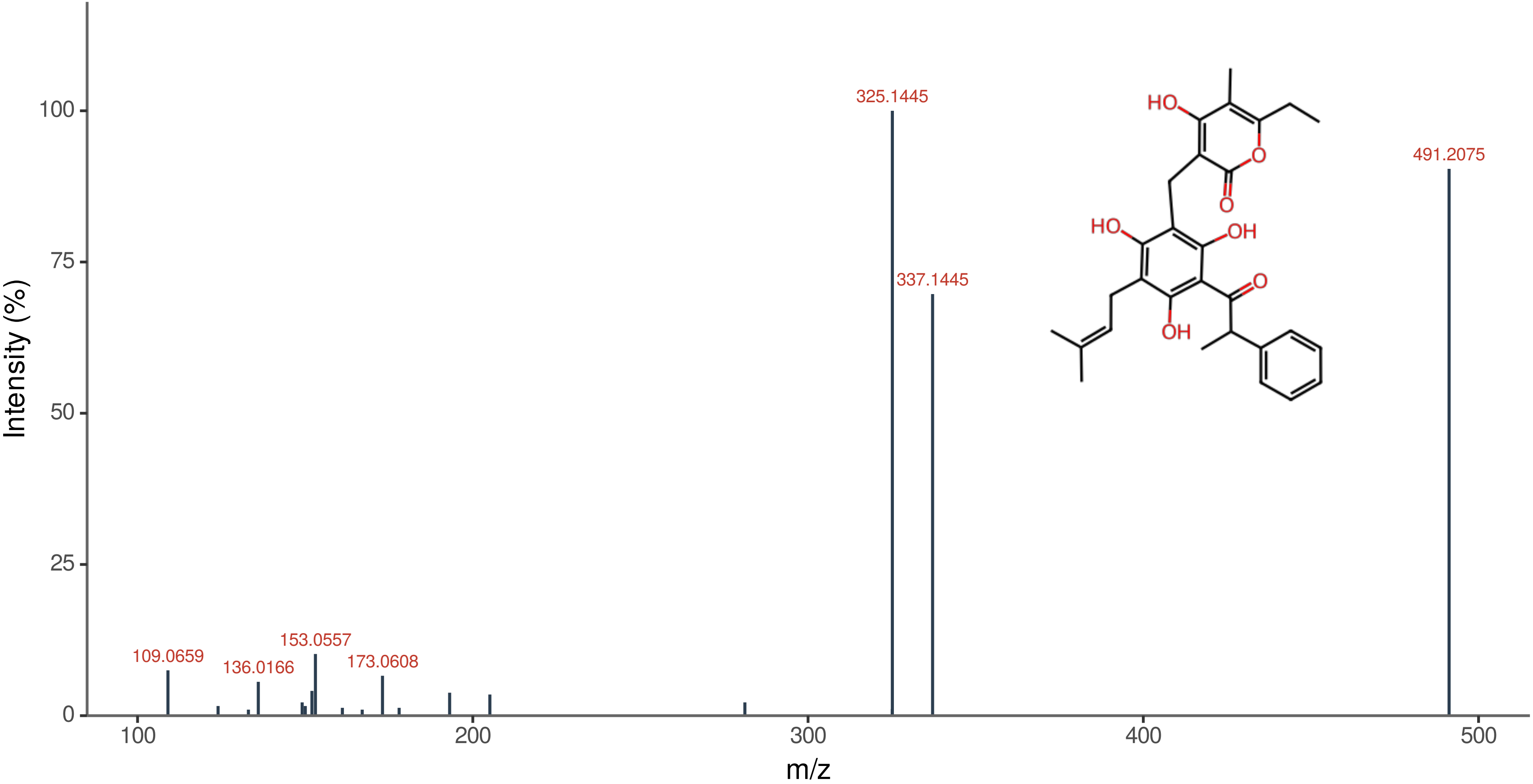

Compound #56 Rt = 14.30 min

$C_{26}H_{31}O_8^-$ $[M-H]^- = 471.2021$

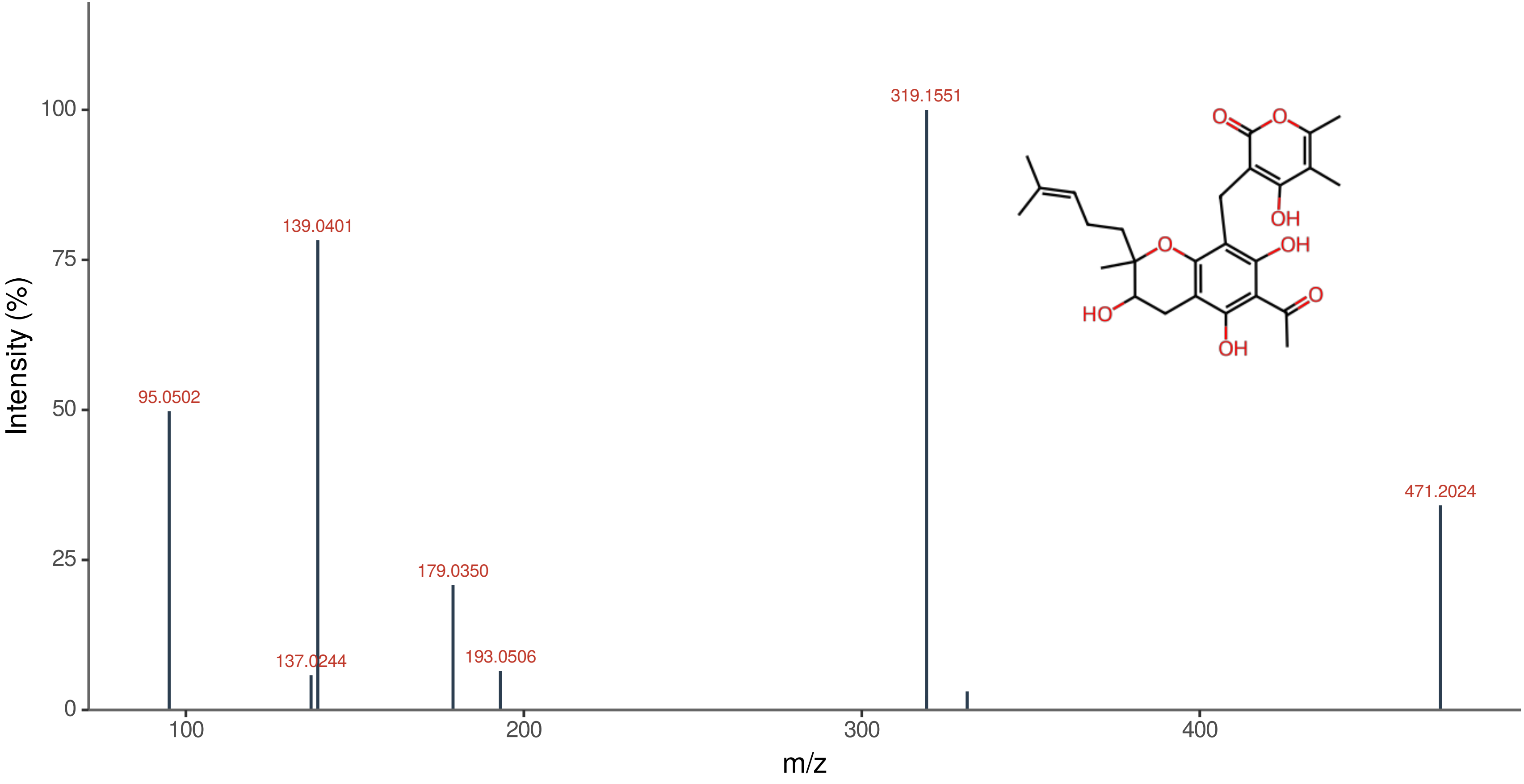

Compound #59 **Rt = 9.21 min**

$C_{20}H_{21}O_6^-$ $[M-H]^- = 357.1346$

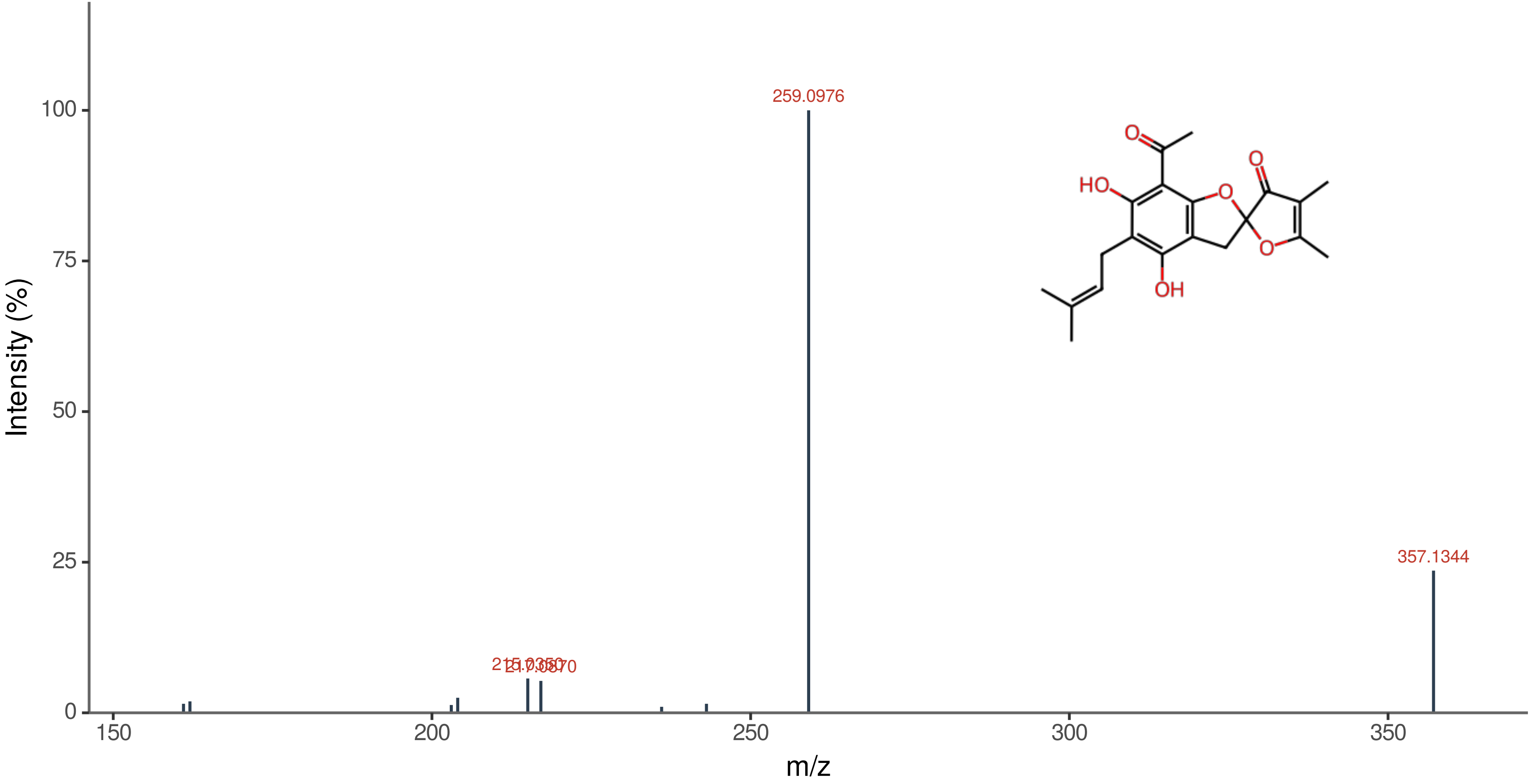

Compound #60 **Rt = 9.57 min**

$C_{20}H_{21}O_6^-$ $[M-H]^- = 357.1346$

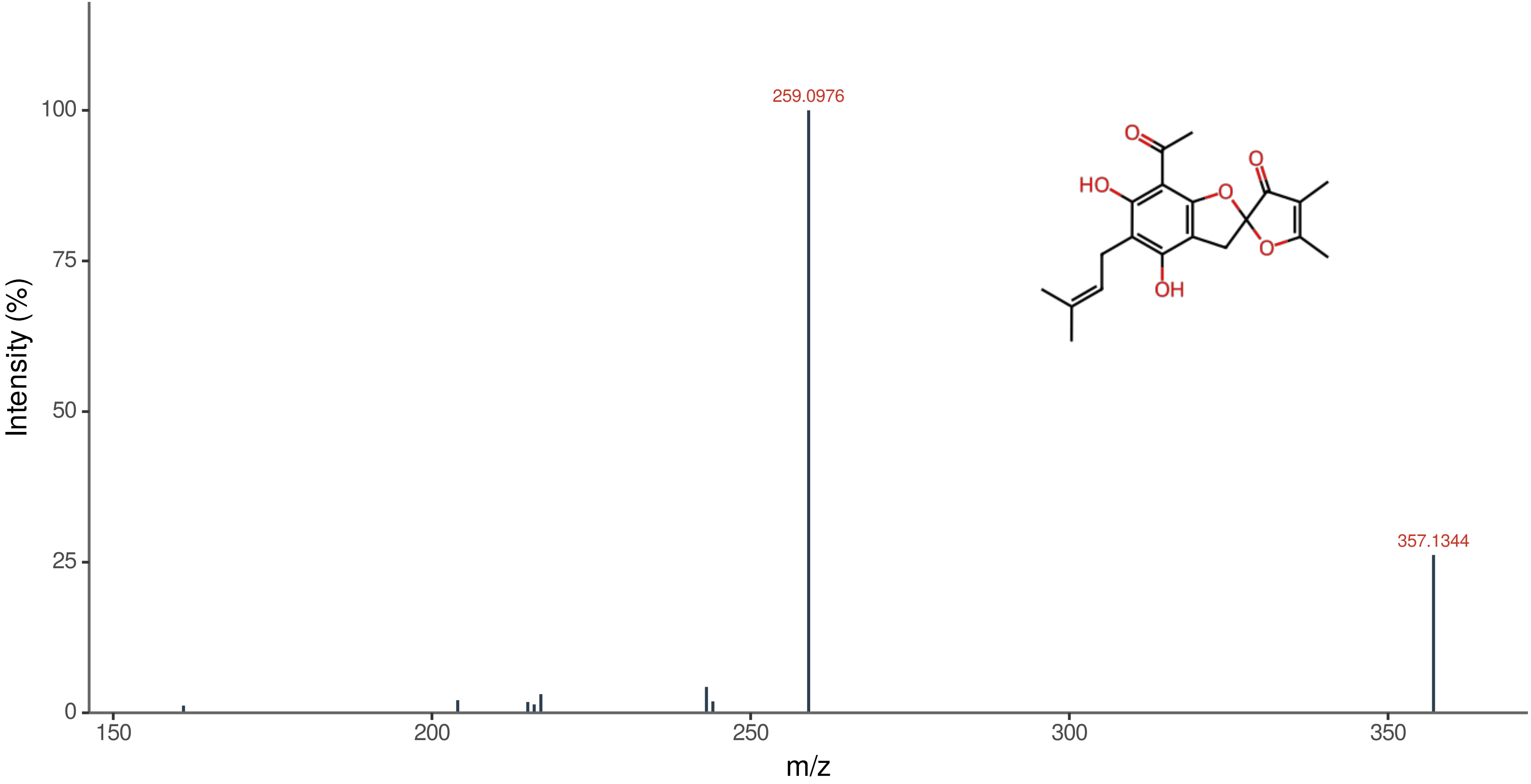

Compound #61　　**Rt = 17.45 min**

$C_{25}H_{31}O_7^-$　　$[M-H]^- = 443.2077$

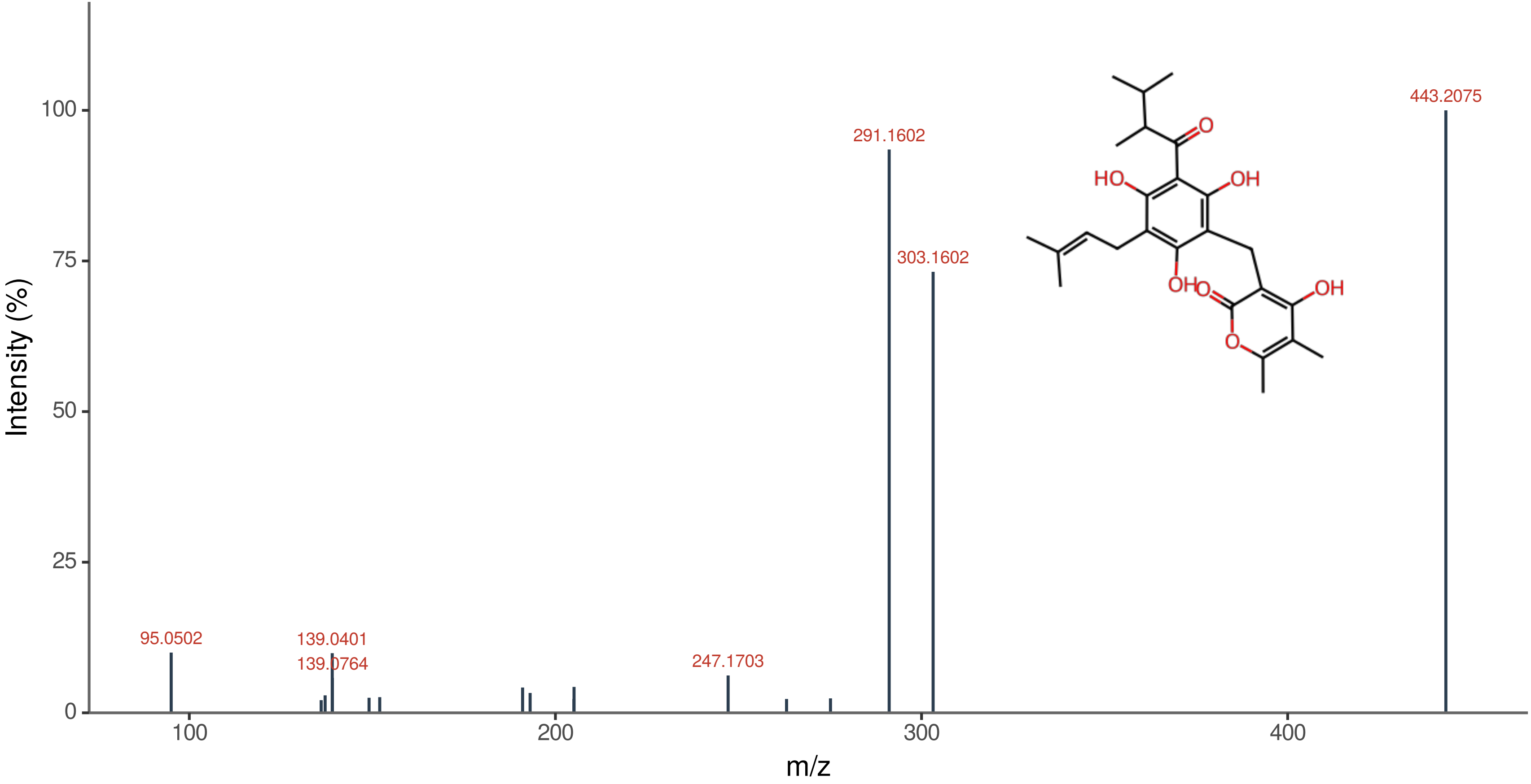

Supplement: Supplementary file 1 [file plants-15-02619-s001.zip › File S1.pdf]
