# Supplementary material for: Comprehensive UHPLC–Orbitrap–MS Profiling of Phloroglucinol α-Pyrones in Helichrysum italicum (Roth) G. Don (Asteraceae)
Source: Plants (Basel). 2026 Aug 27;15(17):2619. doi: 10.3390/plants15172619 (PMC13567118; doi:10.3390/plants15172619)
Supplement: Supplementary file 1 [file plants-15-02619-s001.zip › File S2.pdf]

SUPPLEMENTARY MATERIAL

Proposed ESI(–) MS/MS fragmentation pathways of the detected compounds

This document compiles the proposed negative-ion (deprotonated, $[M–H]^-$) tandem mass spectrometry (MS/MS) fragmentation pathways for the compounds detected in this study. Some compounds share an identical proposed fragmentation pathway; in those cases a single scheme is presented for the group and its page is labelled with all of the relevant compound numbers. The precursor ion is shown at the centre of each scheme, with arrows leading to the proposed product ions.

Associated article

Voynikov Y, Marinov T, Nedialkov P. *Comprehensive UHPLC–Orbitrap–MS Profiling of Phloroglucinol α-Pyrones in Helichrysum italicum (Roth) G. Don (Asteraceae)*. Plants, 2026.

cmpd_1

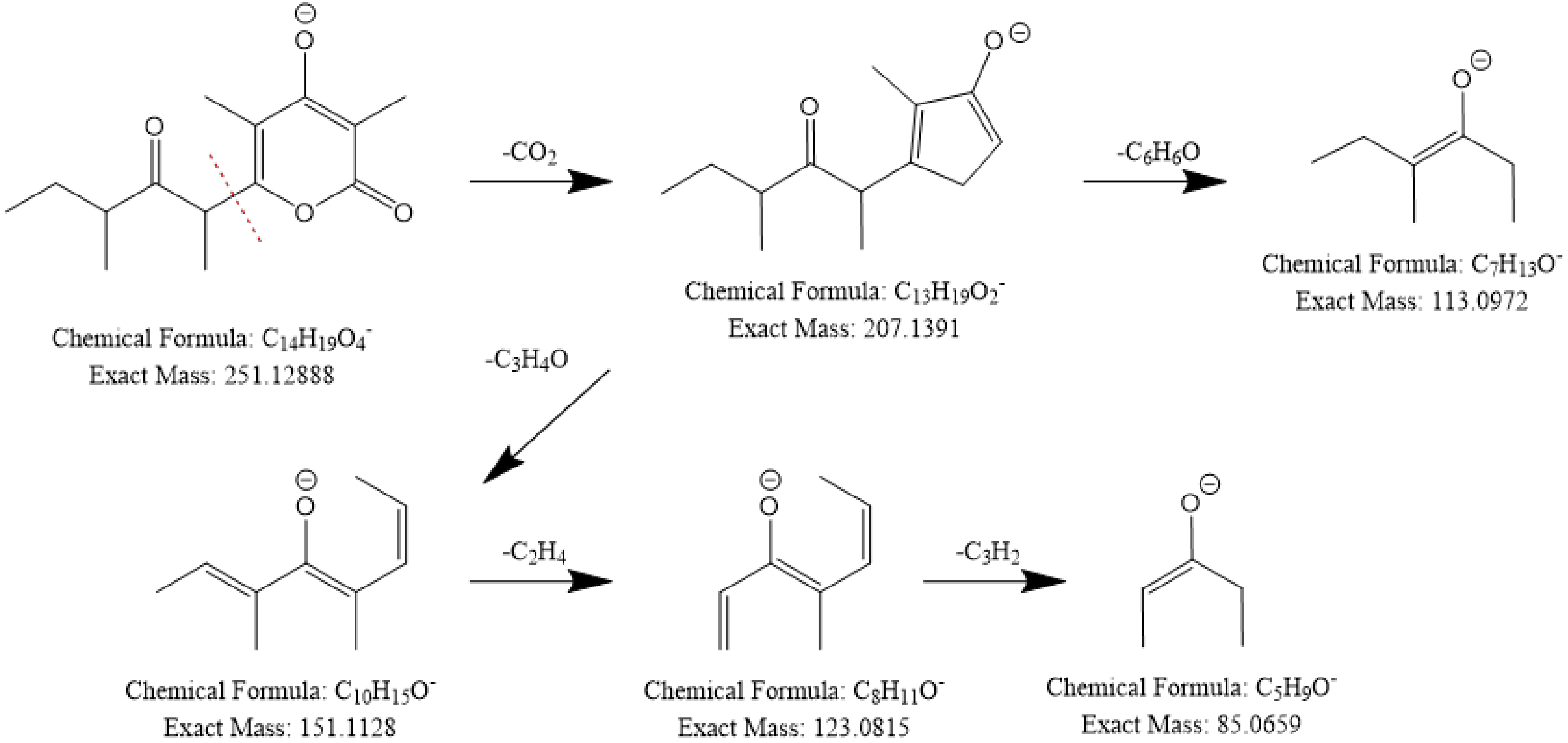

Figure S1. Proposed negative-ion MS/MS fragmentation pathway of compound 1.

cmpd_2

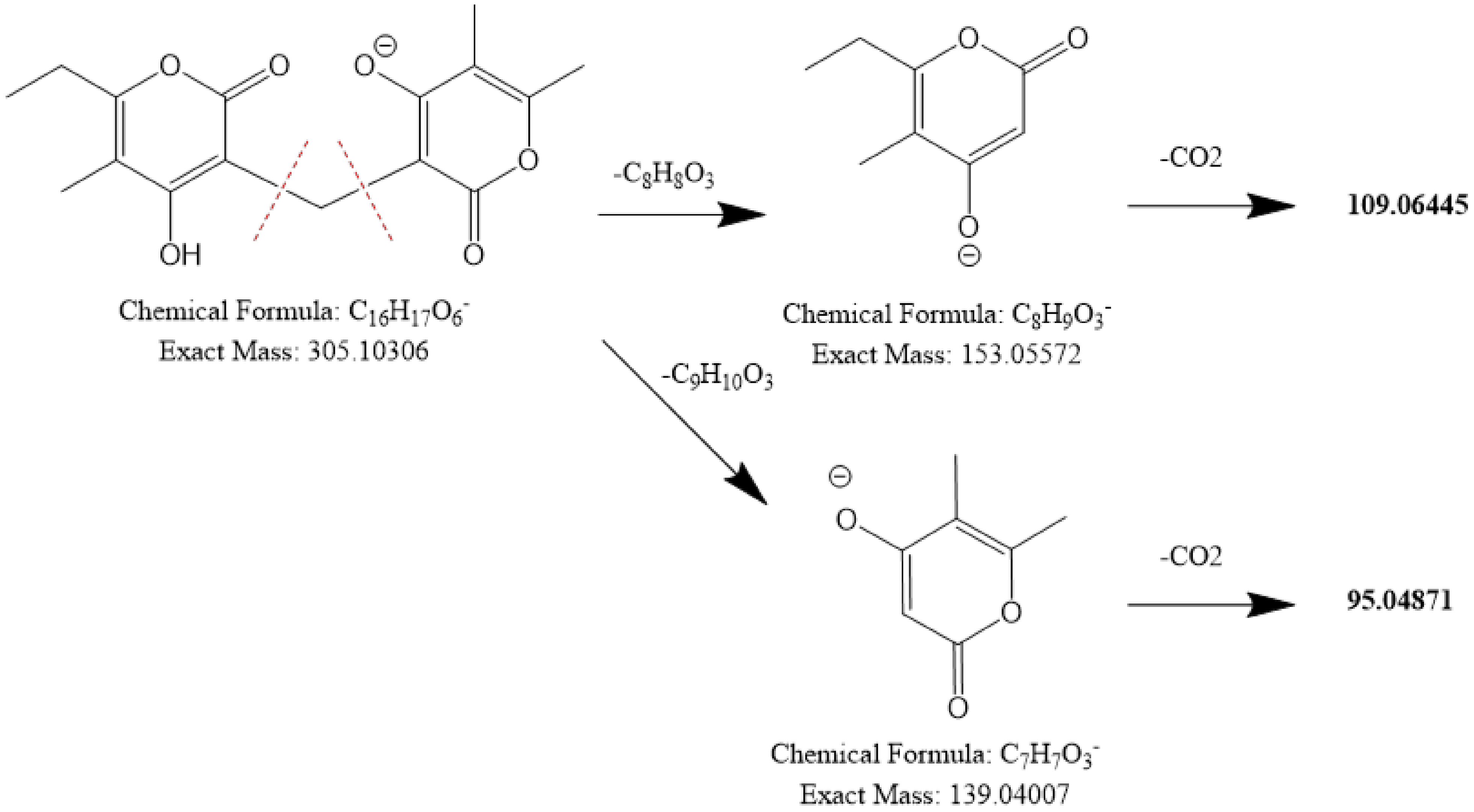

Figure S2. Proposed negative-ion MS/MS fragmentation pathway of compound 2.

cmpd_3

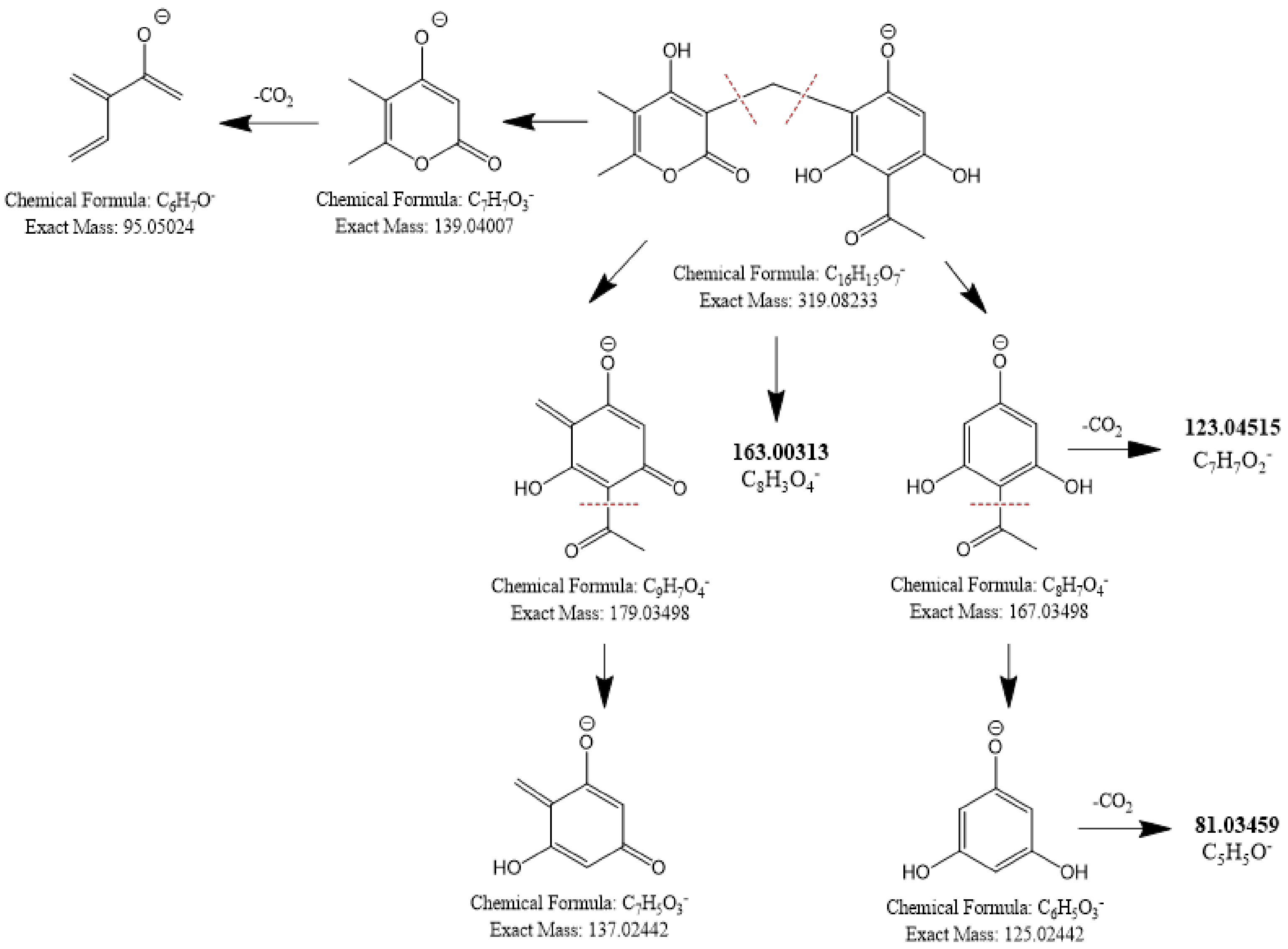

Figure S3. Proposed negative-ion MS/MS fragmentation pathway of compound 3.

cmpd_4

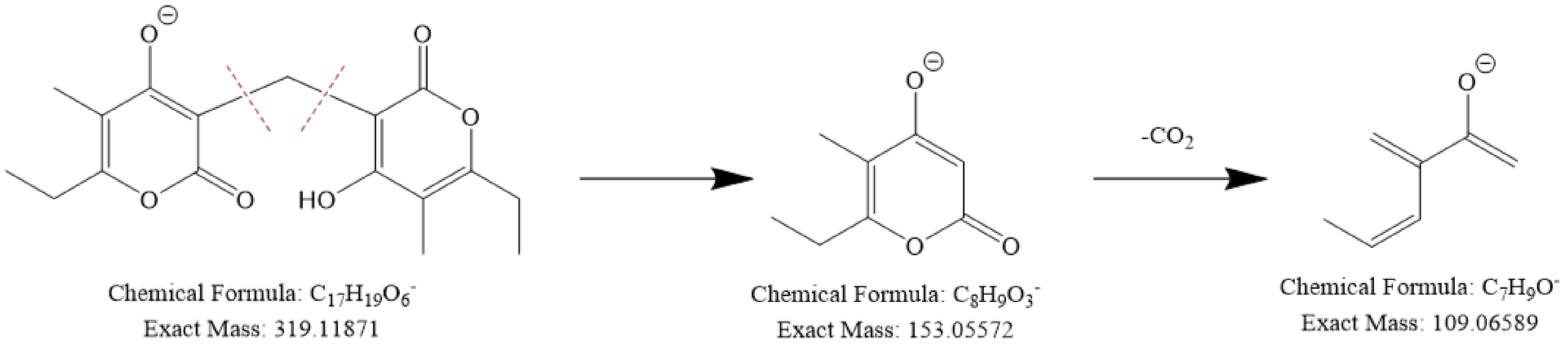

Figure S4. Proposed negative-ion MS/MS fragmentation pathway of compound 4.

cmpd_5

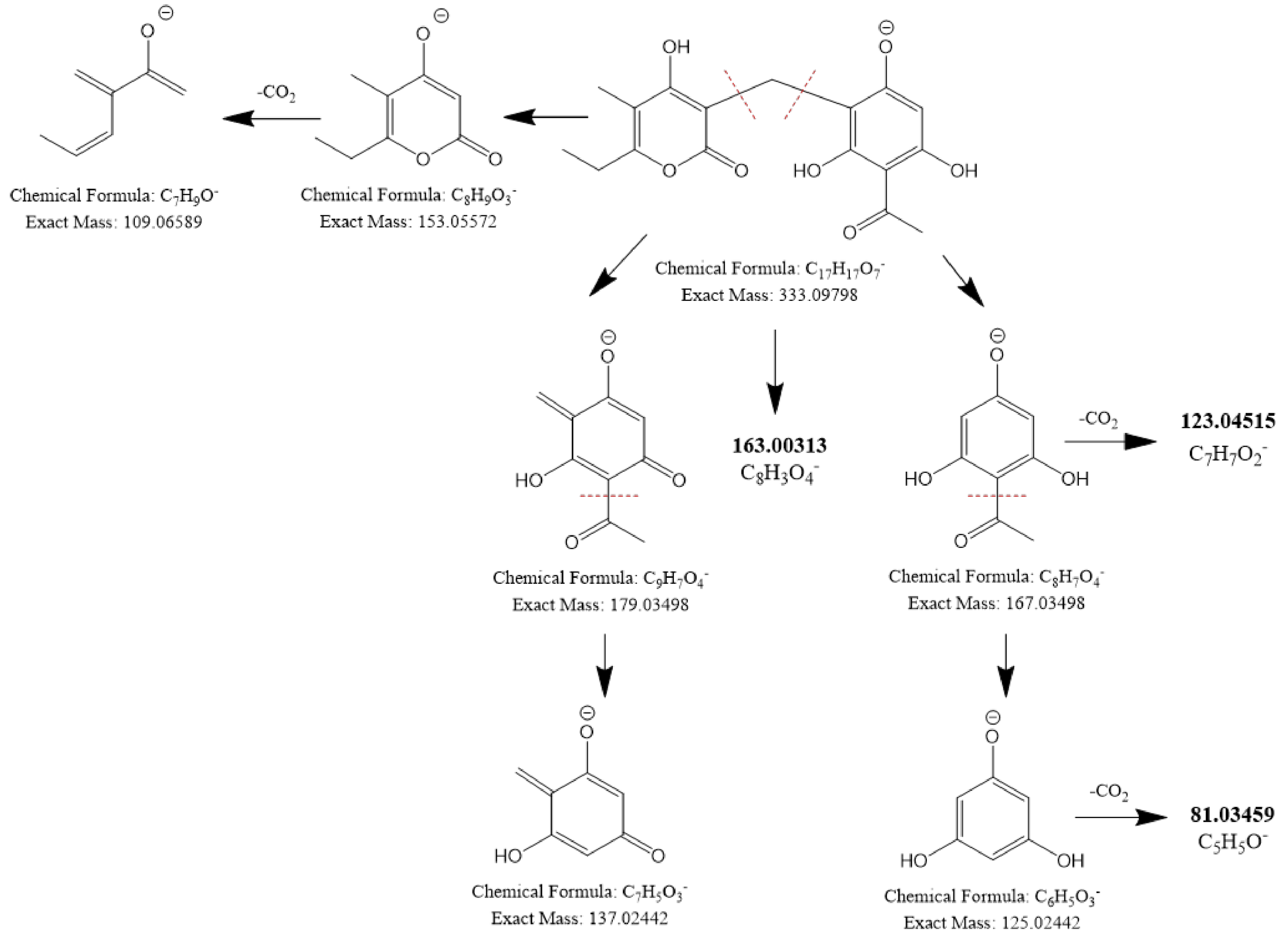

Figure S5. Proposed negative-ion MS/MS fragmentation pathway of compound 5.

cmpd_6

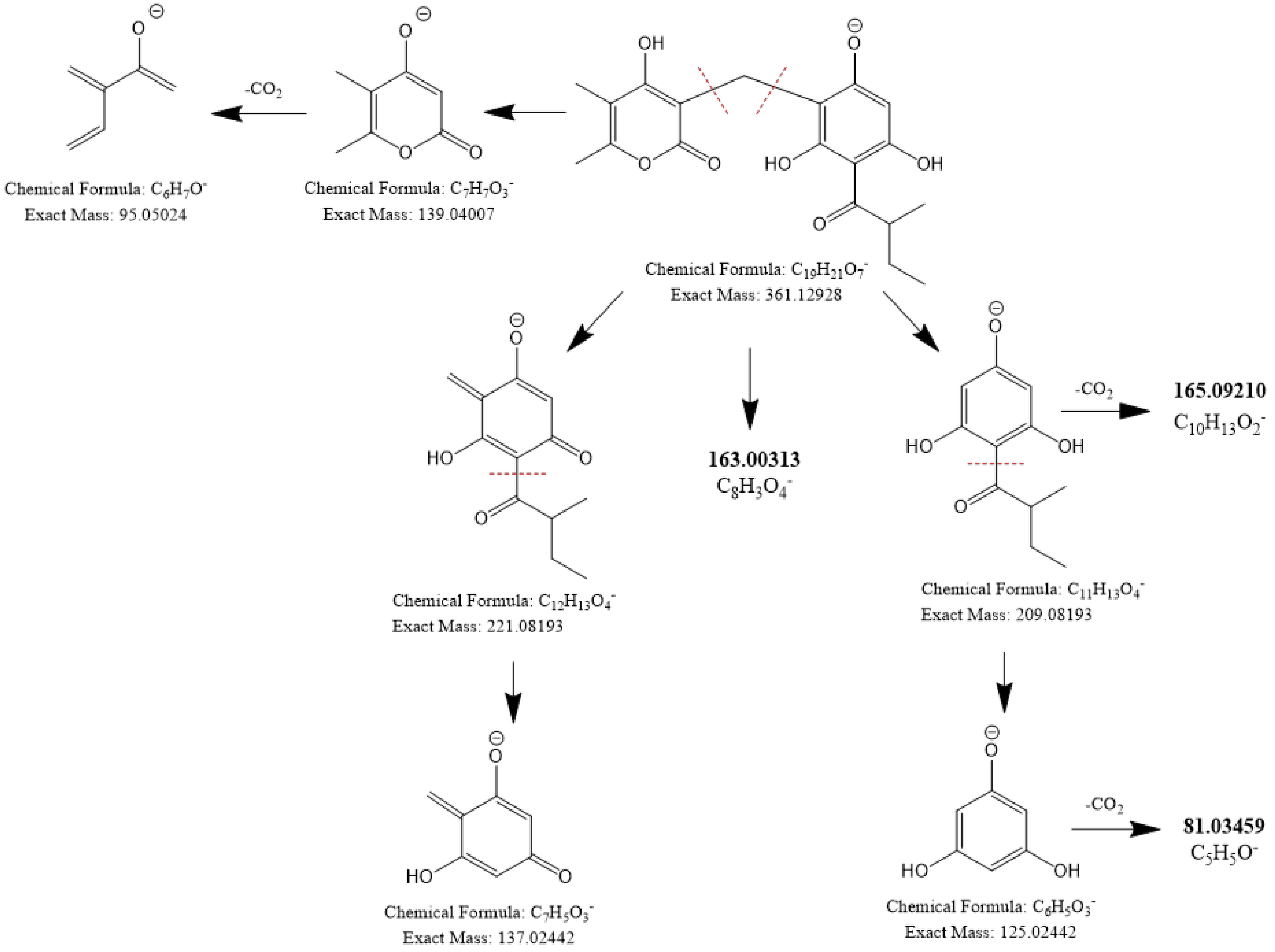

Figure S6. Proposed negative-ion MS/MS fragmentation pathway of compound 6.

cmpd_7

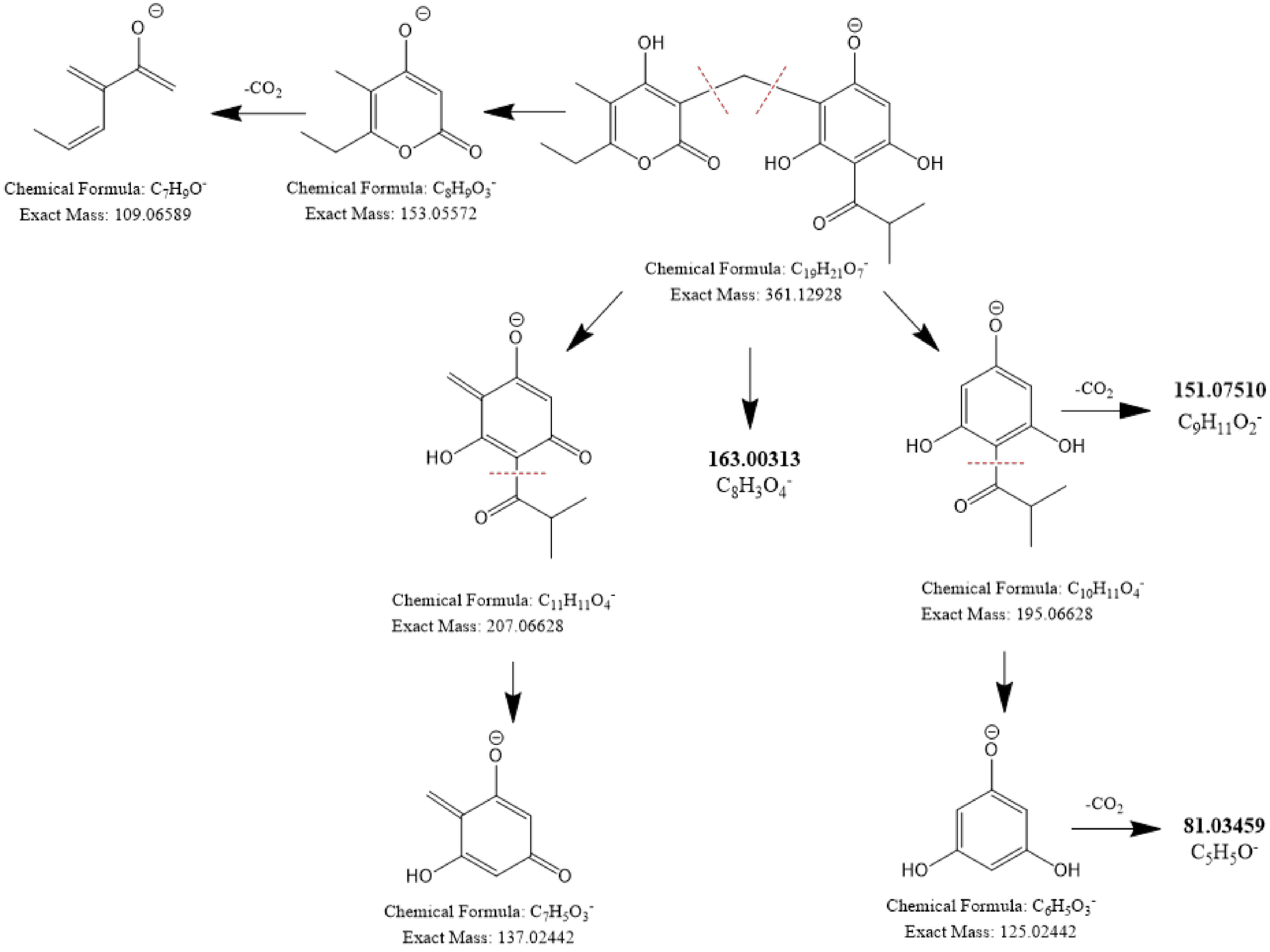

Figure S7. Proposed negative-ion MS/MS fragmentation pathway of compound 7.

cmpd_8

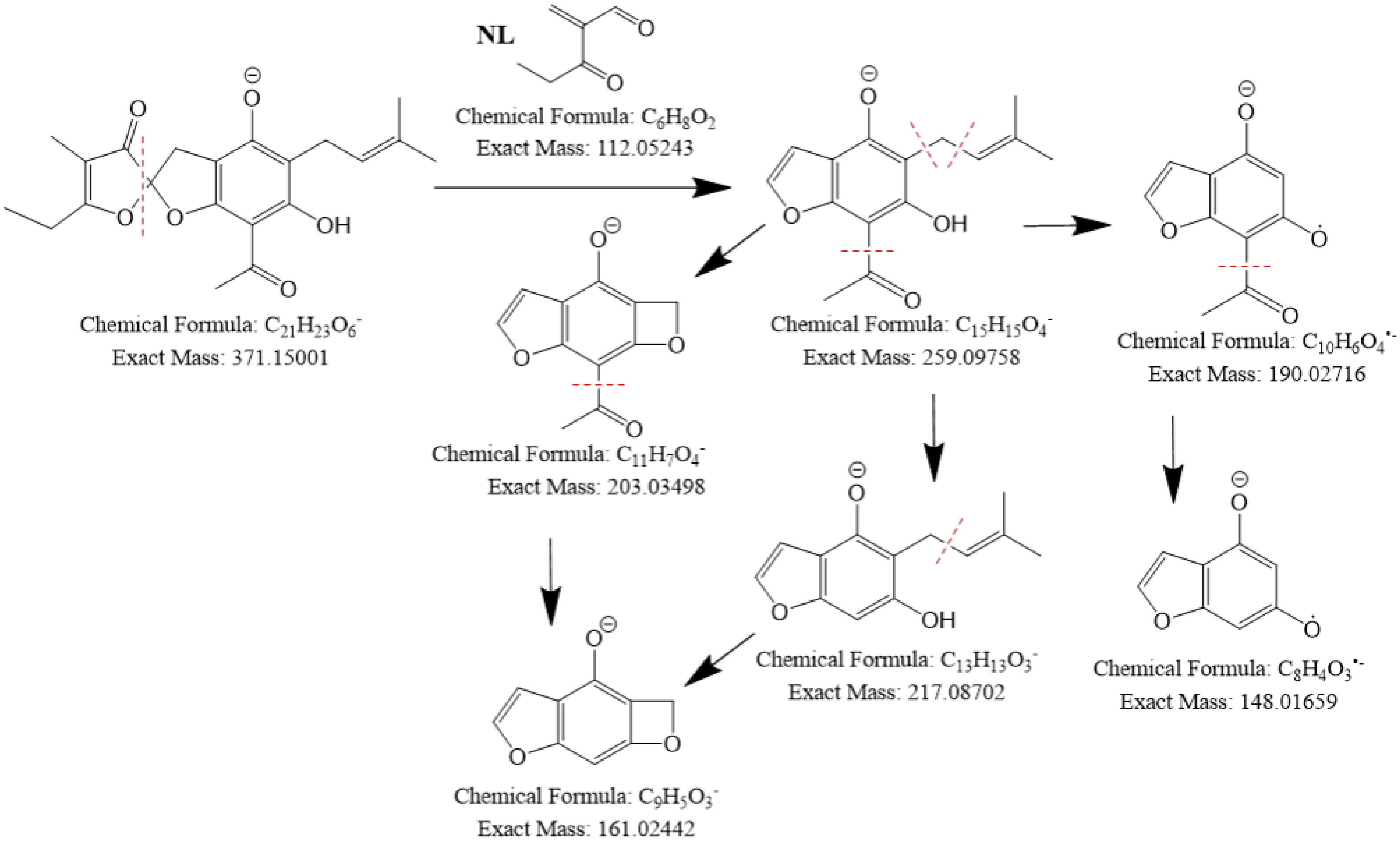

Figure S8. Proposed negative-ion MS/MS fragmentation pathway of compound 8.

cmpd_9

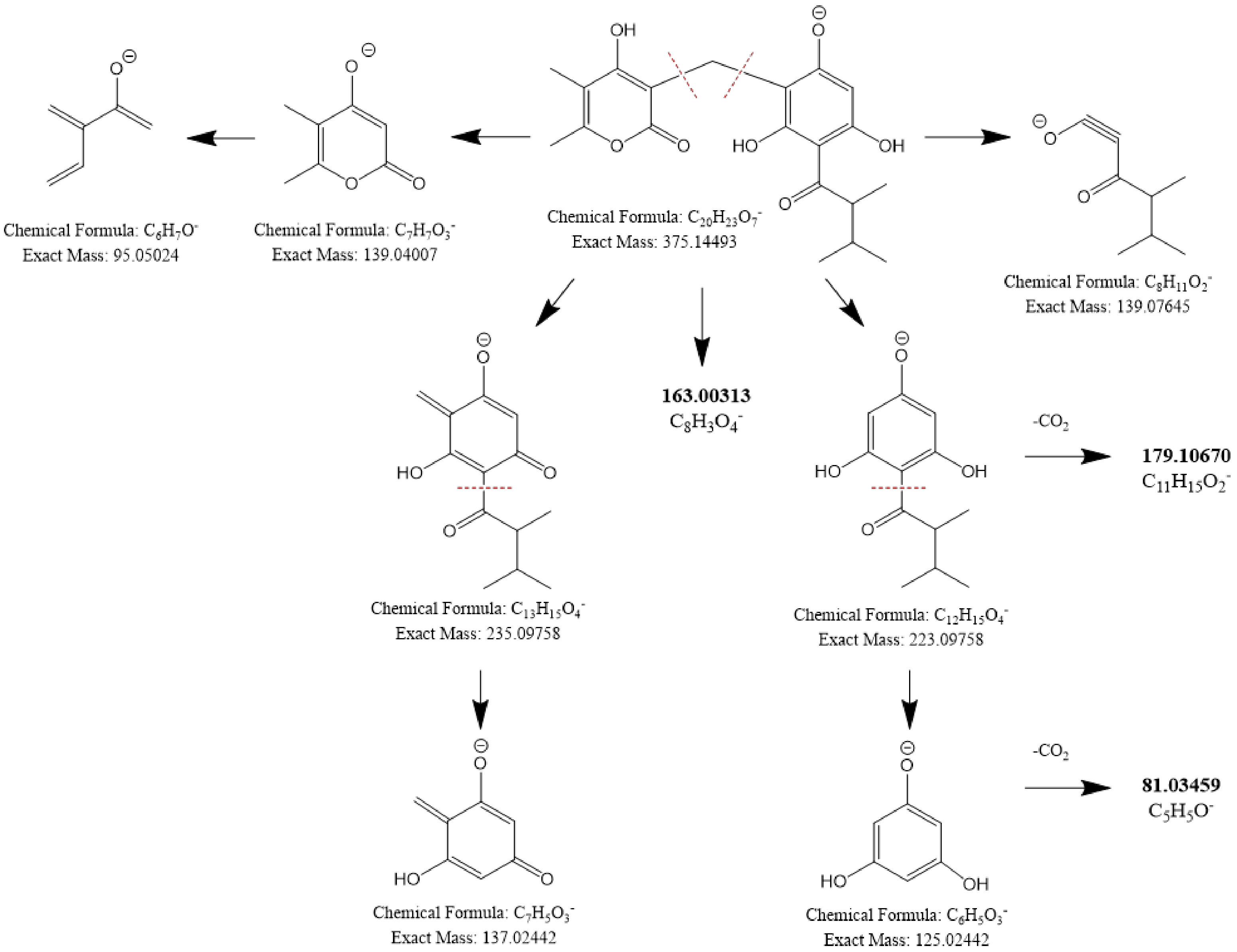

Figure S9. Proposed negative-ion MS/MS fragmentation pathway of compound 9.

cmpd_10

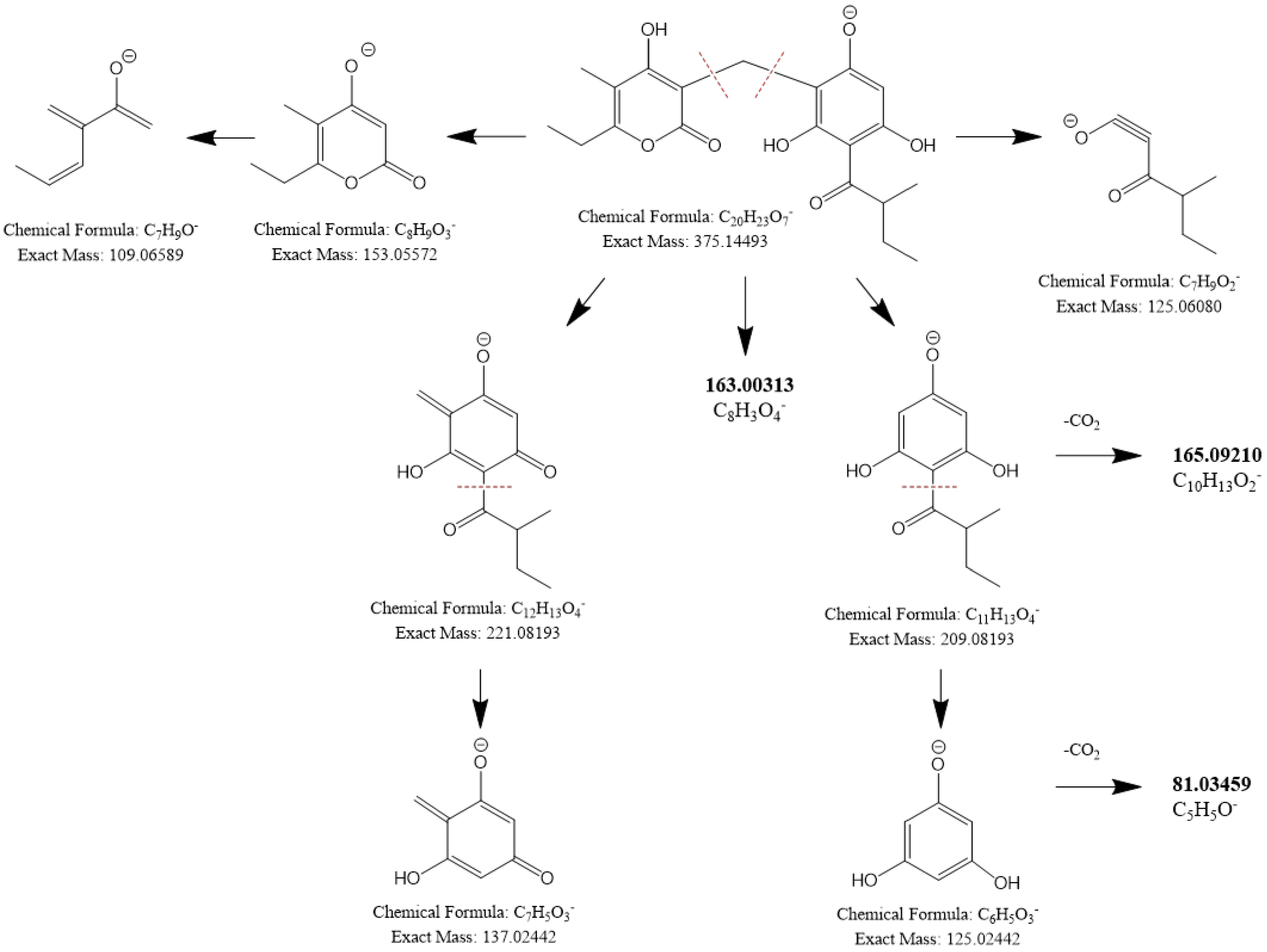

Figure S10. Proposed negative-ion MS/MS fragmentation pathway of compound 10.

cmpd_11_12

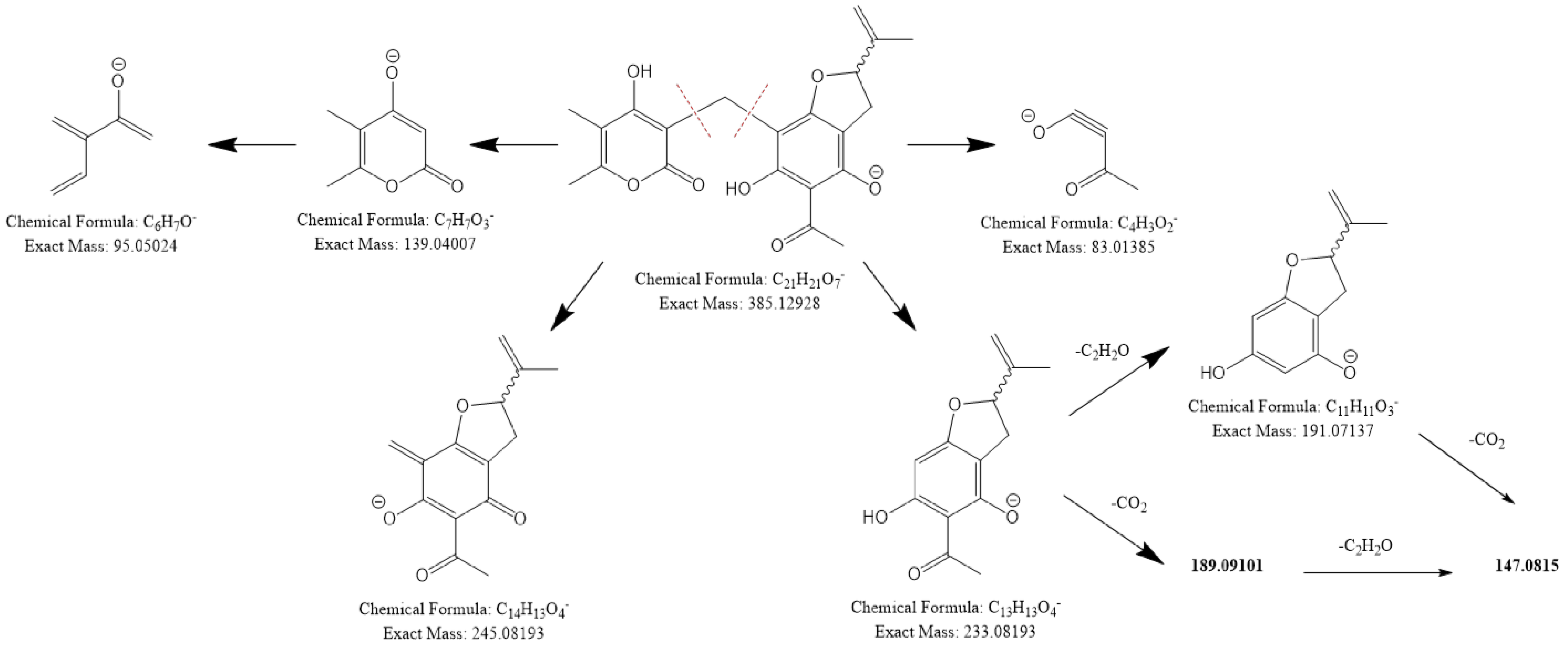

Figure S11. Proposed negative-ion MS/MS fragmentation pathway of compound 11/12.

cmpd_13

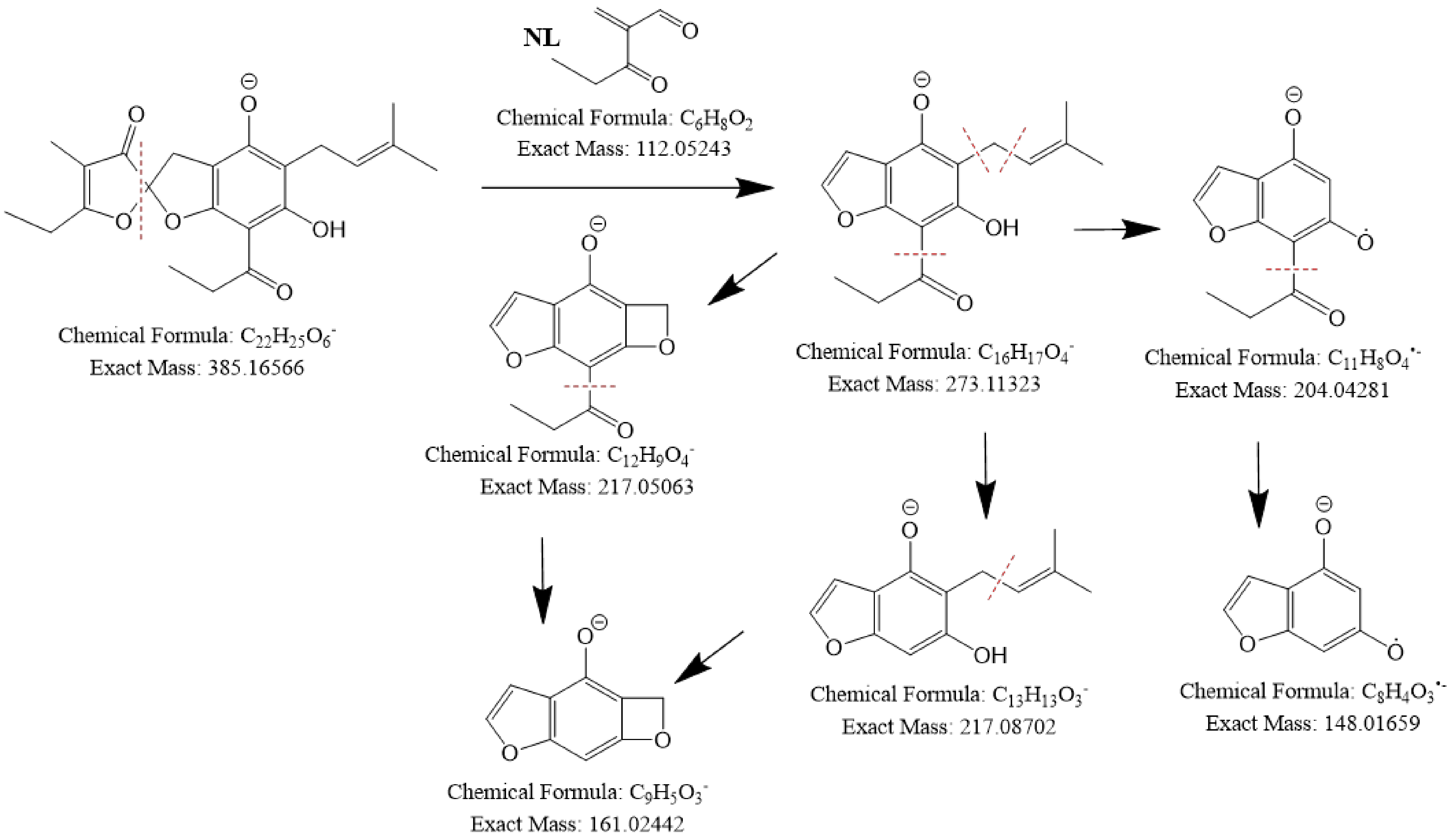

Figure S12. Proposed negative-ion MS/MS fragmentation pathway of compound 13.

cmpd_14

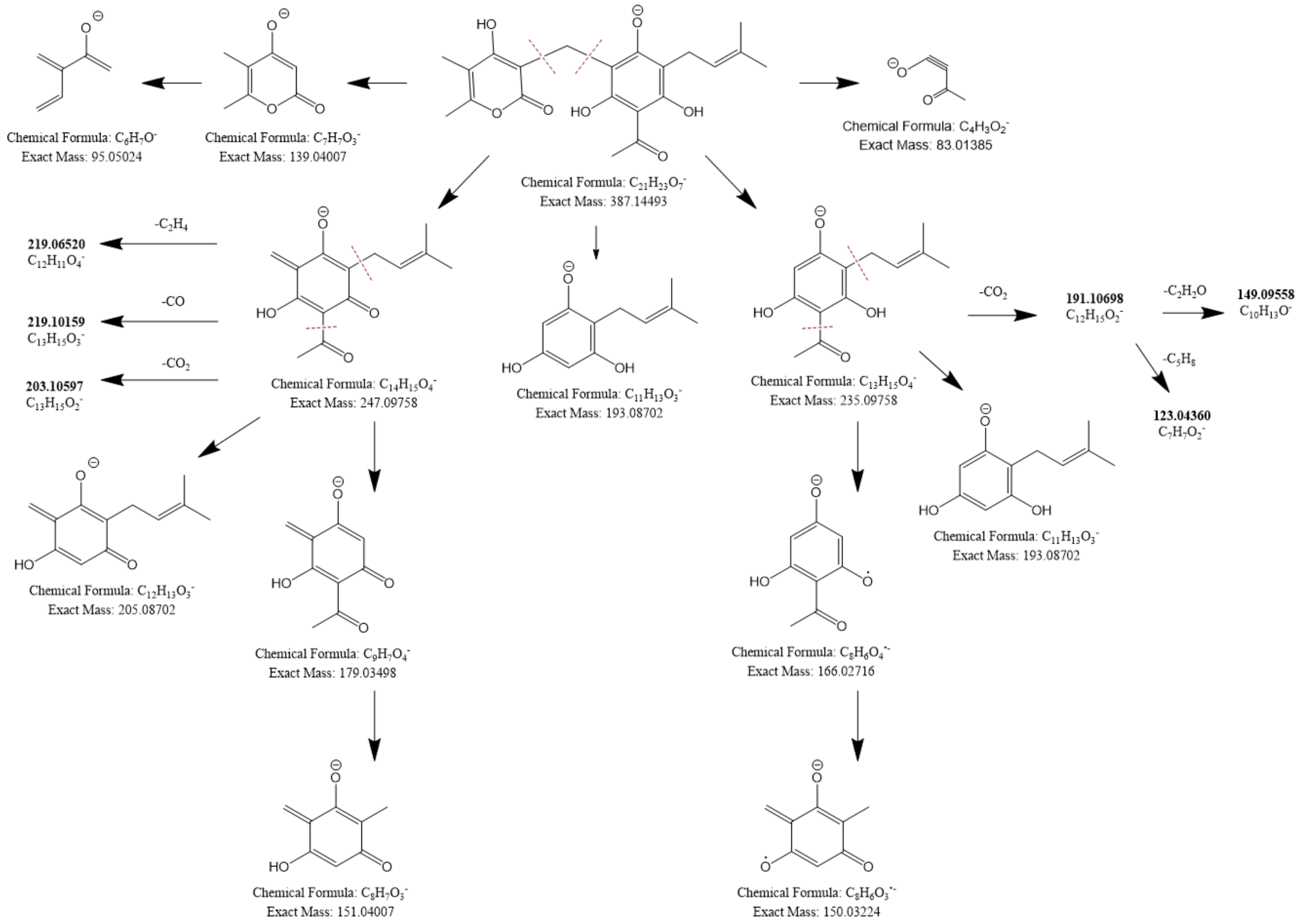

Figure S13. Proposed negative-ion MS/MS fragmentation pathway of compound 14.

cmpd_15_16

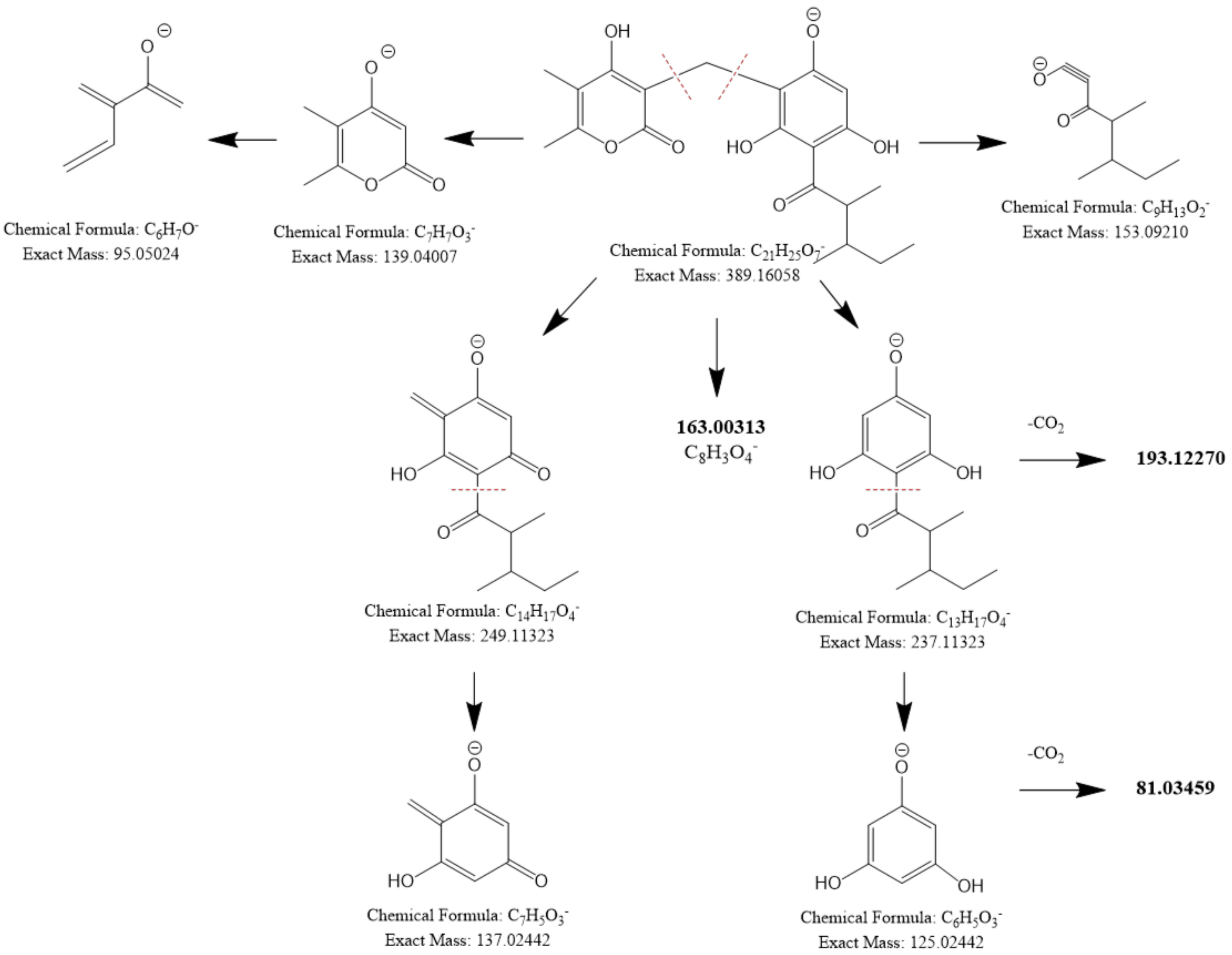

Figure S14. Proposed negative-ion MS/MS fragmentation pathway of compound 15/16.

cmpd_17

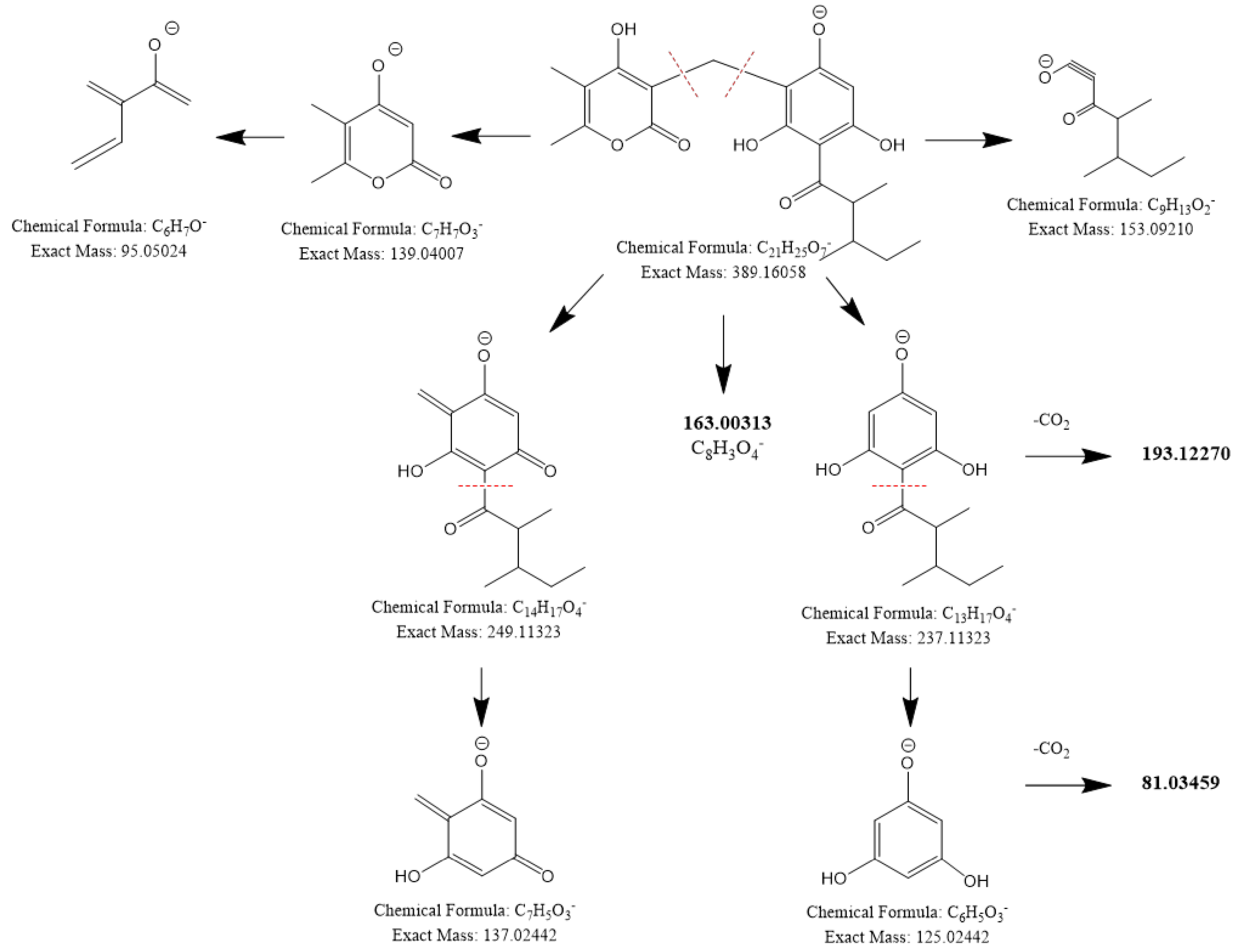

Figure S15. Proposed negative-ion MS/MS fragmentation pathway of compound 17.

cmpd_18_19

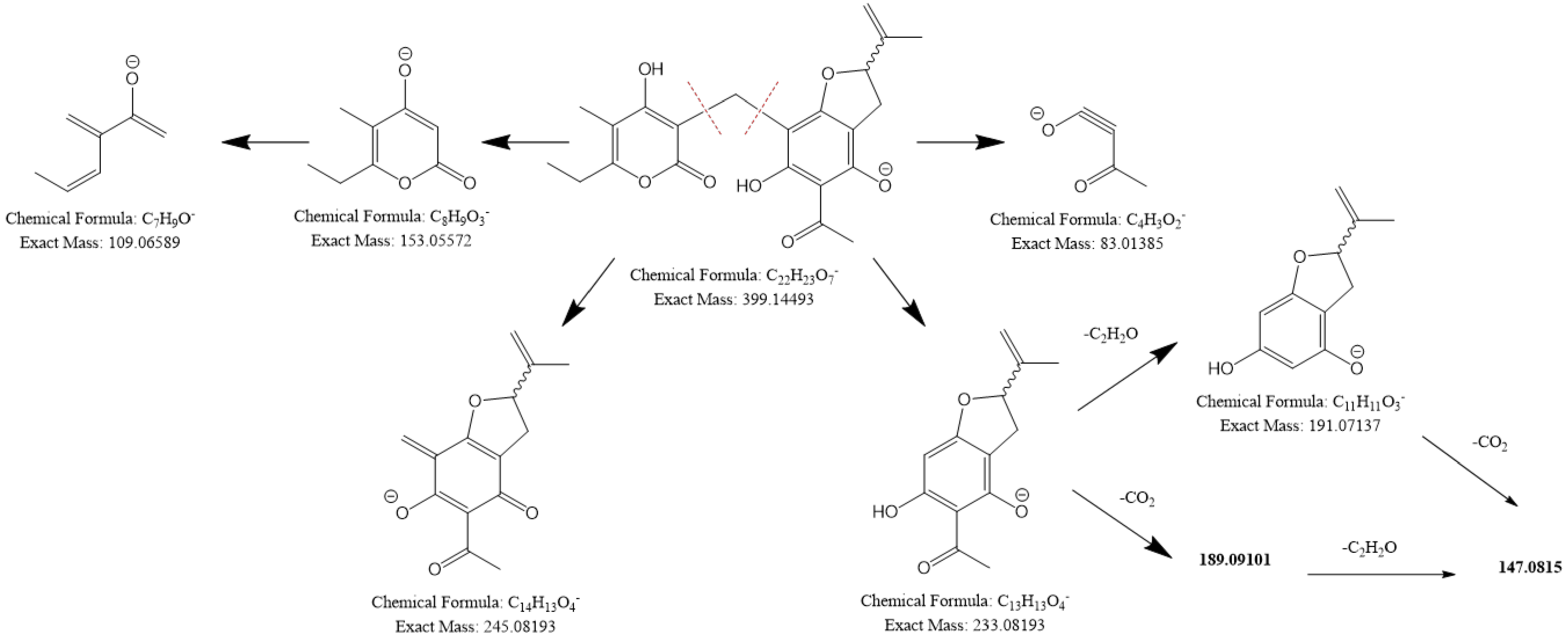

Figure S16. Proposed negative-ion MS/MS fragmentation pathway of compound 18/19.

cmpd_20

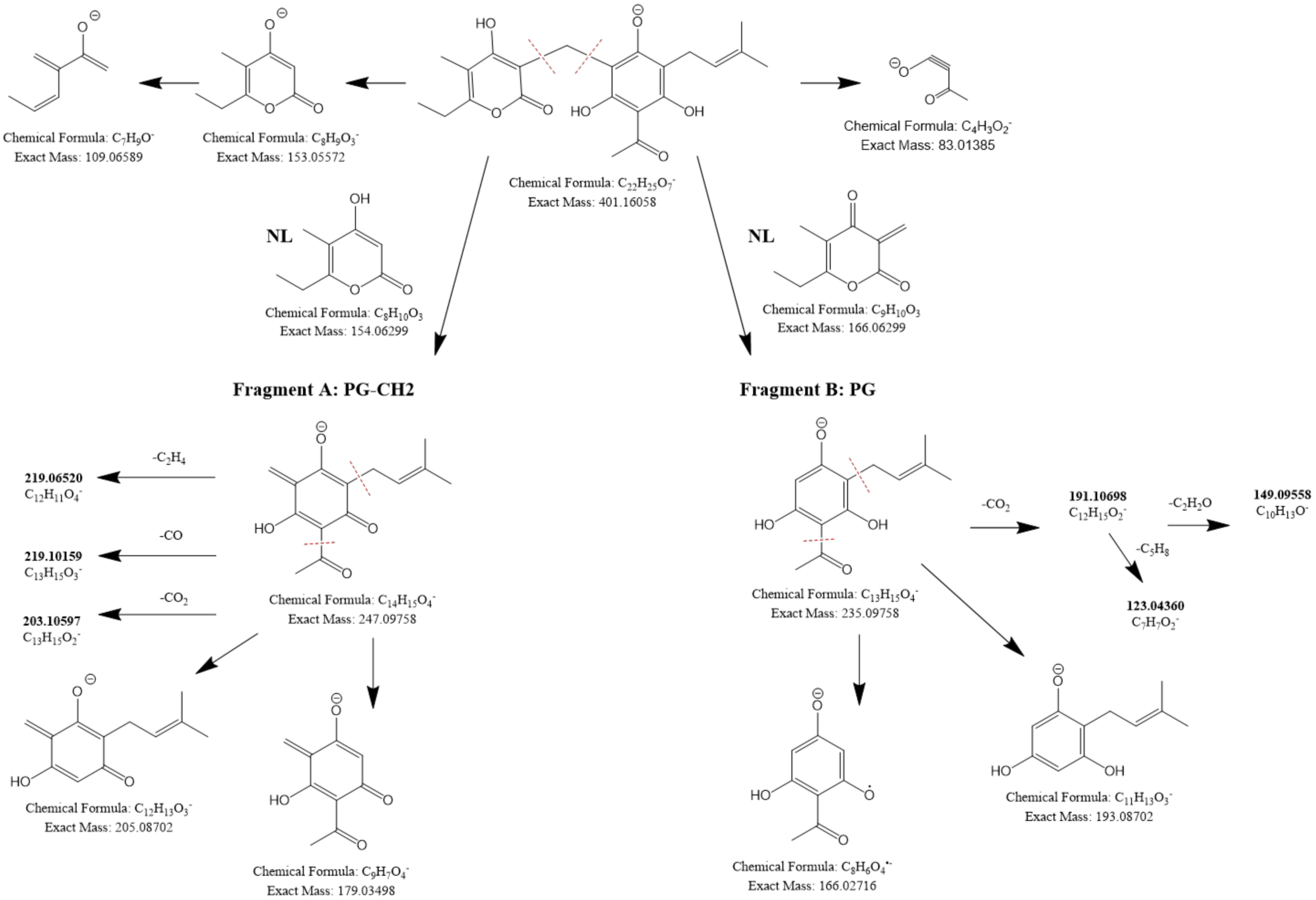

Figure S17. Proposed negative-ion MS/MS fragmentation pathway of compound 20.

cmpd_21

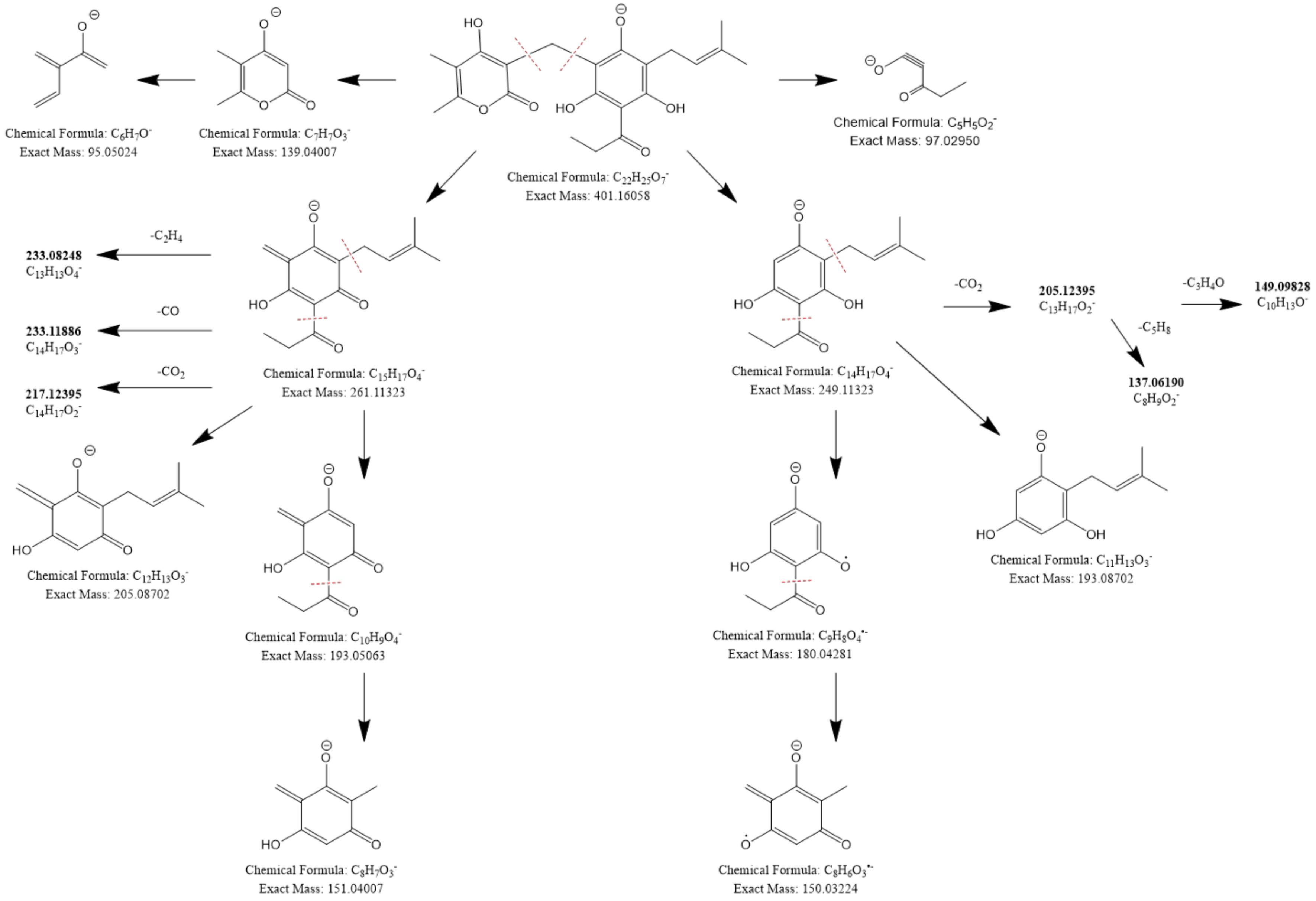

Figure S18. Proposed negative-ion MS/MS fragmentation pathway of compound 21.

cmpd_22_23

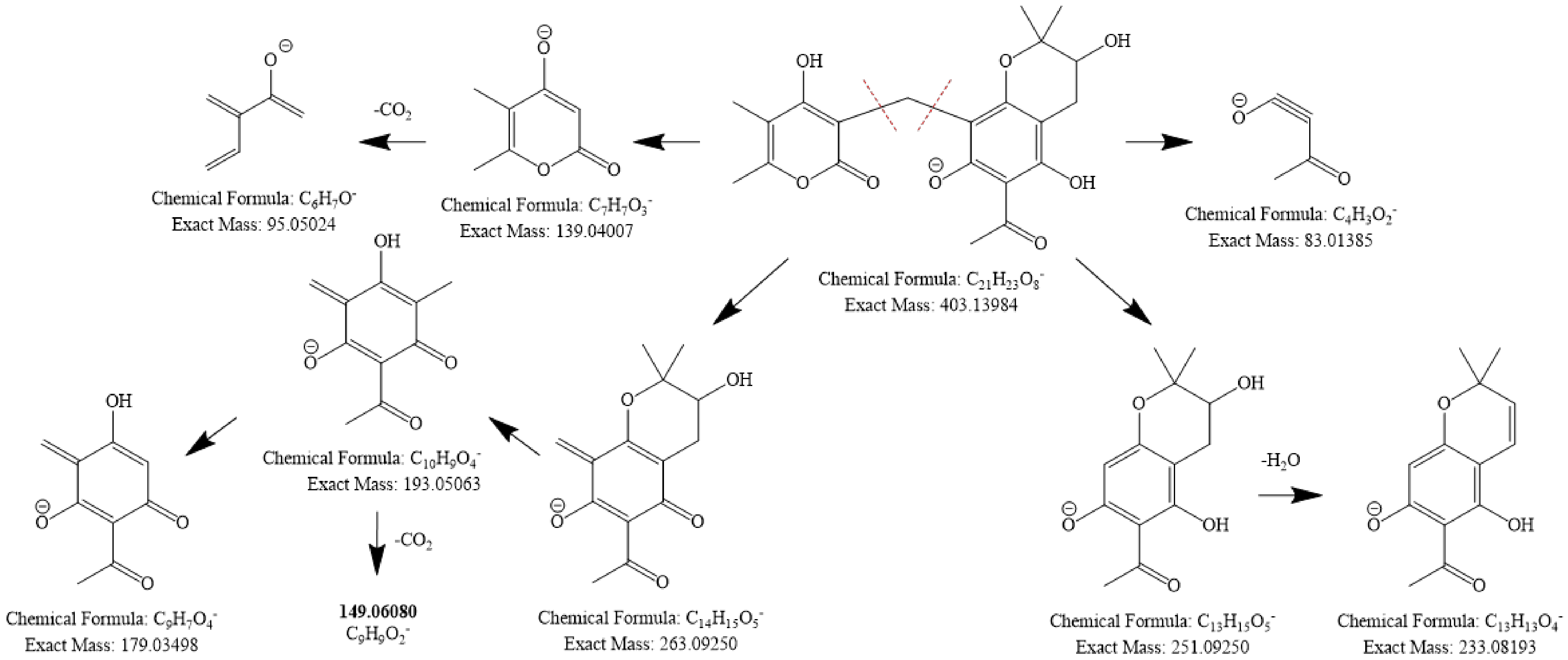

Figure S19. Proposed negative-ion MS/MS fragmentation pathway of compound 22/23.

cmpd_24

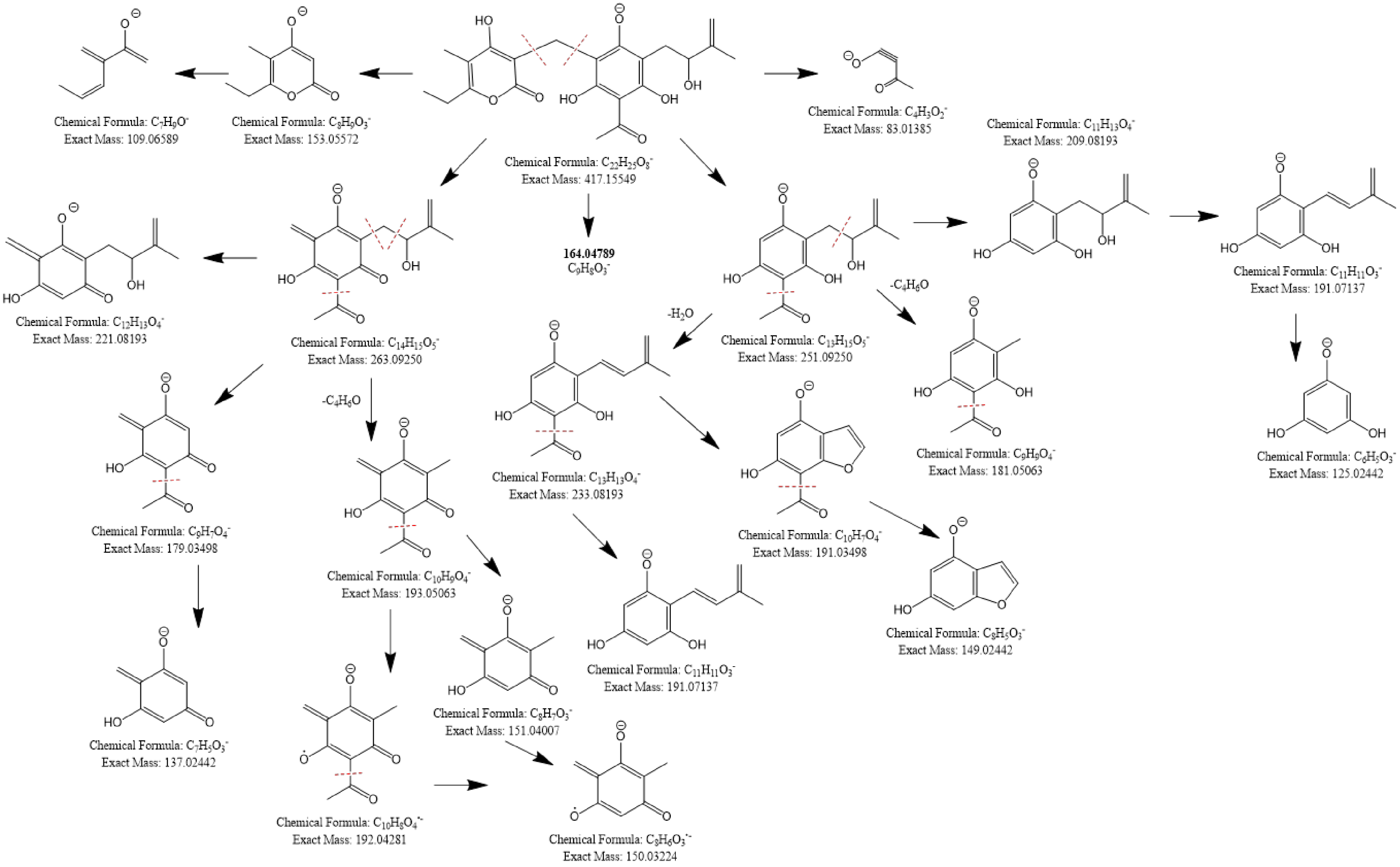

Figure S20. Proposed negative-ion MS/MS fragmentation pathway of compound 24.

cmpd_25

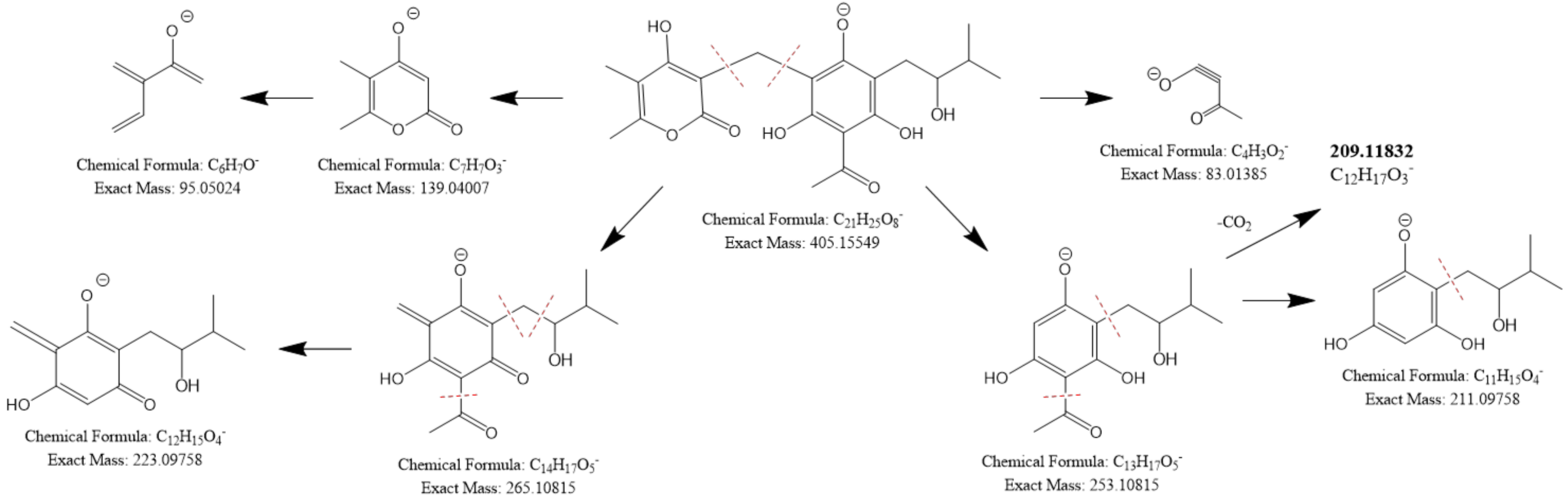

Figure S21. Proposed negative-ion MS/MS fragmentation pathway of compound 25.

cmpd_26

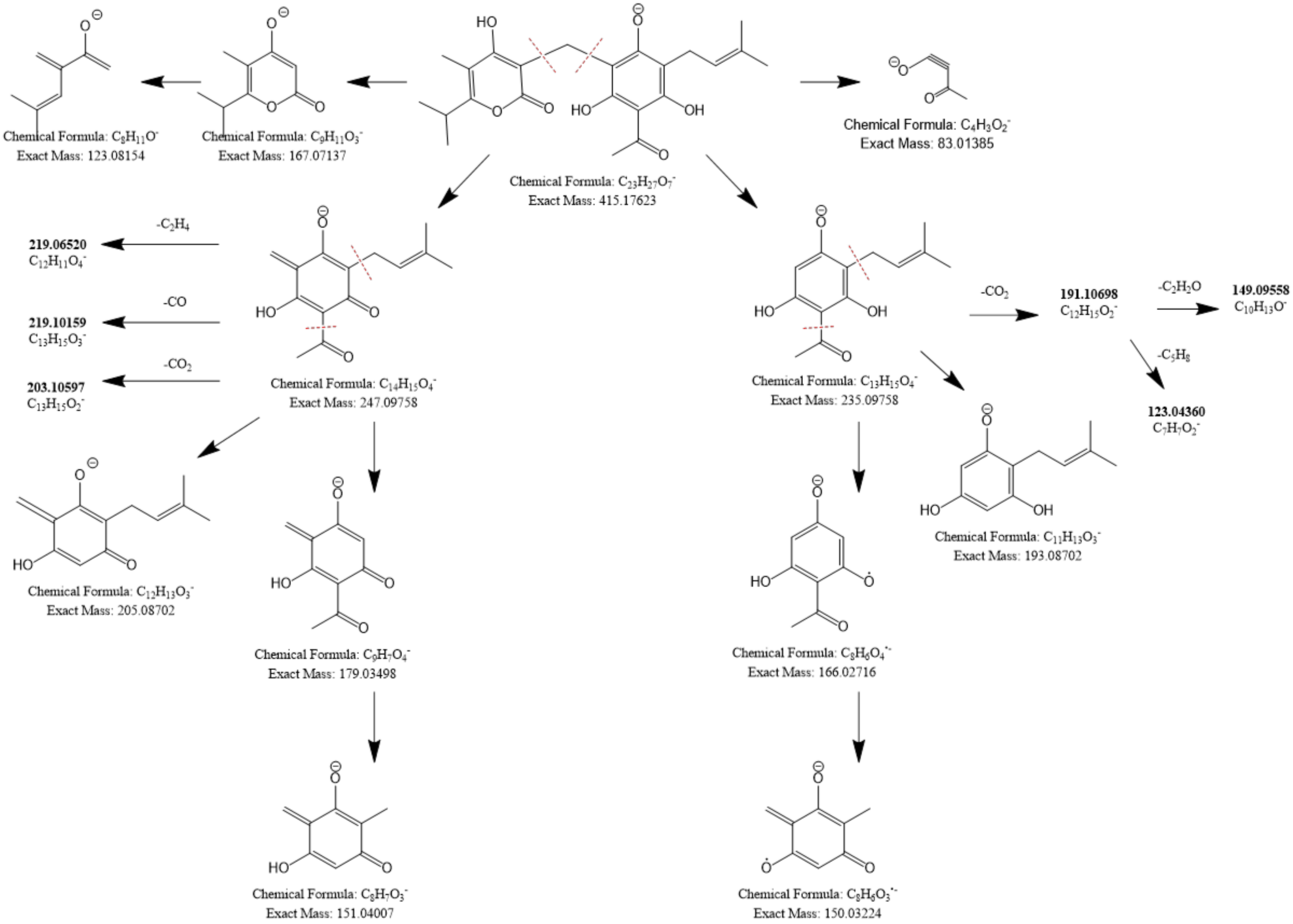

Figure S22. Proposed negative-ion MS/MS fragmentation pathway of compound 26.

cmpd_27

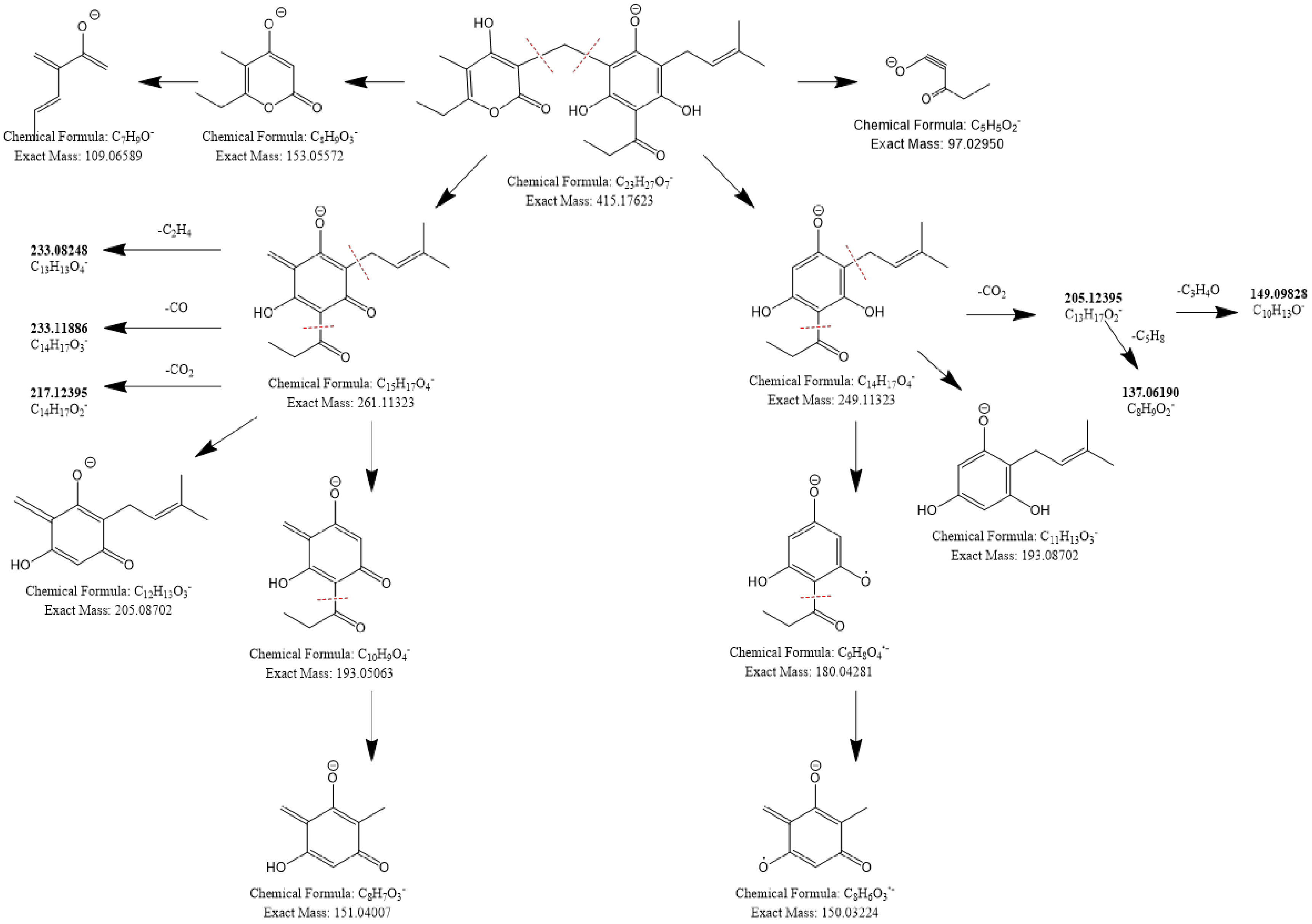

Figure S23. Proposed negative-ion MS/MS fragmentation pathway of compound 27.

cmpd_28

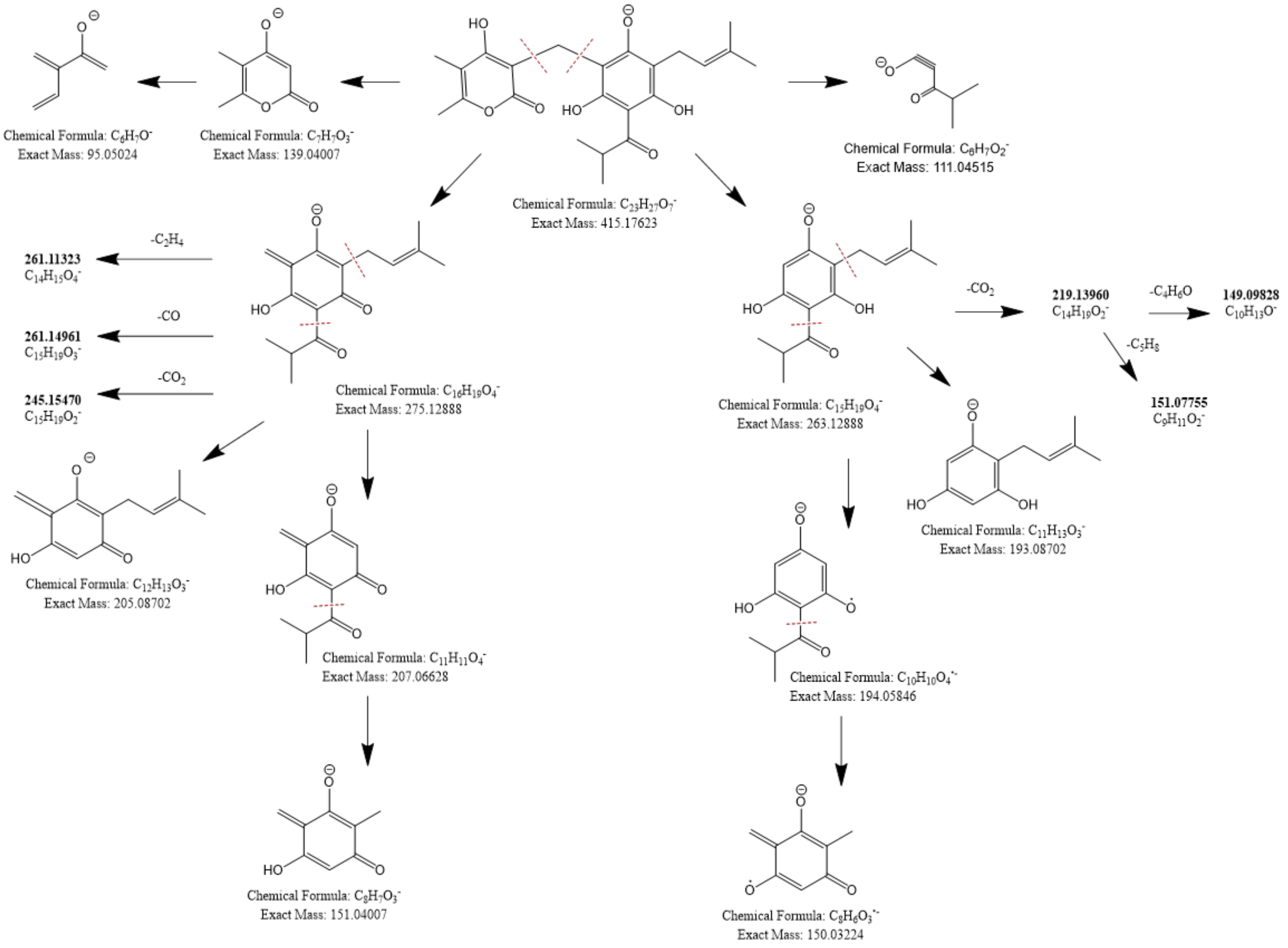

Figure S24. Proposed negative-ion MS/MS fragmentation pathway of compound 28.

cmpd_29

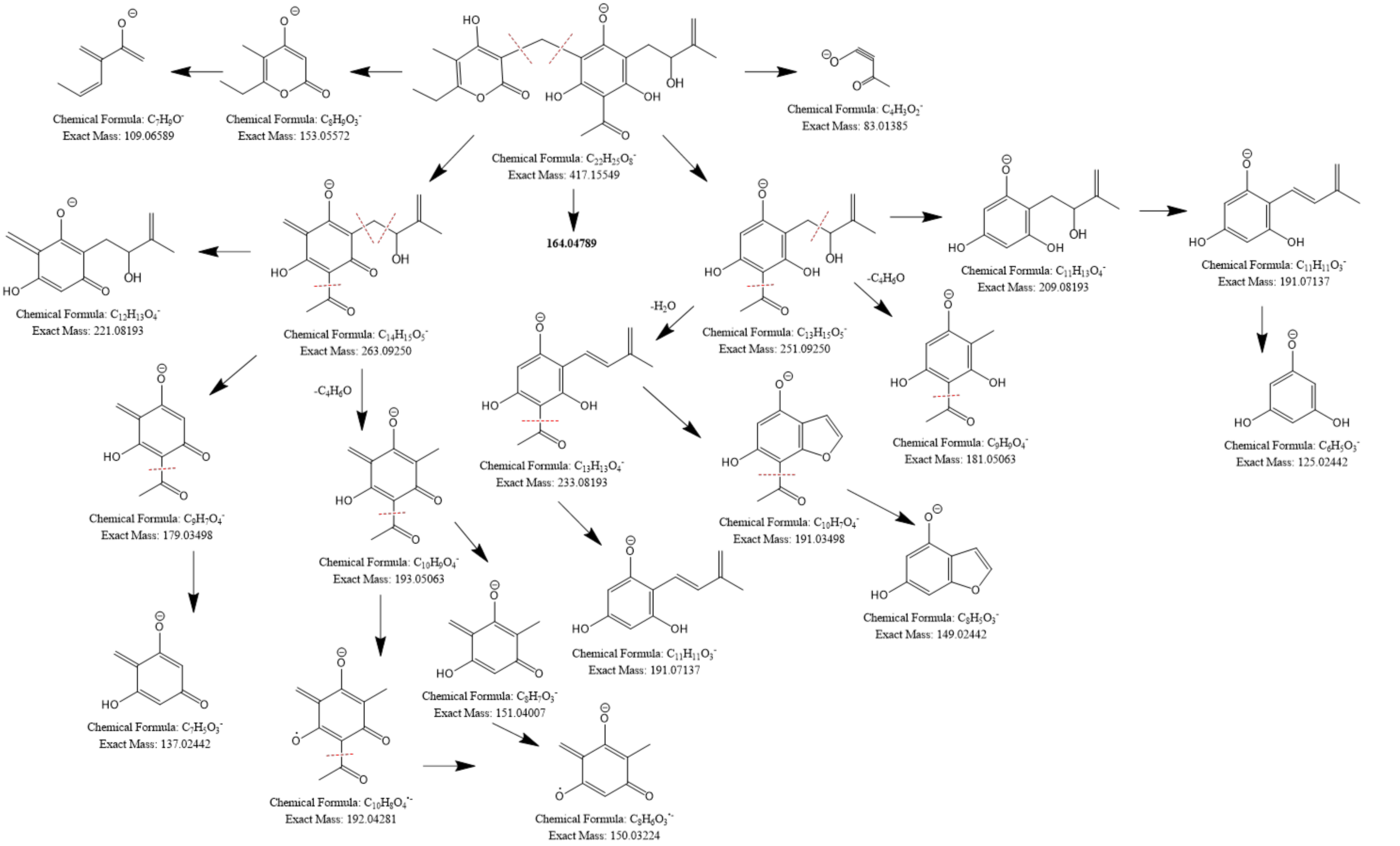

Figure S25. Proposed negative-ion MS/MS fragmentation pathway of compound 29.

cmpd_30_31

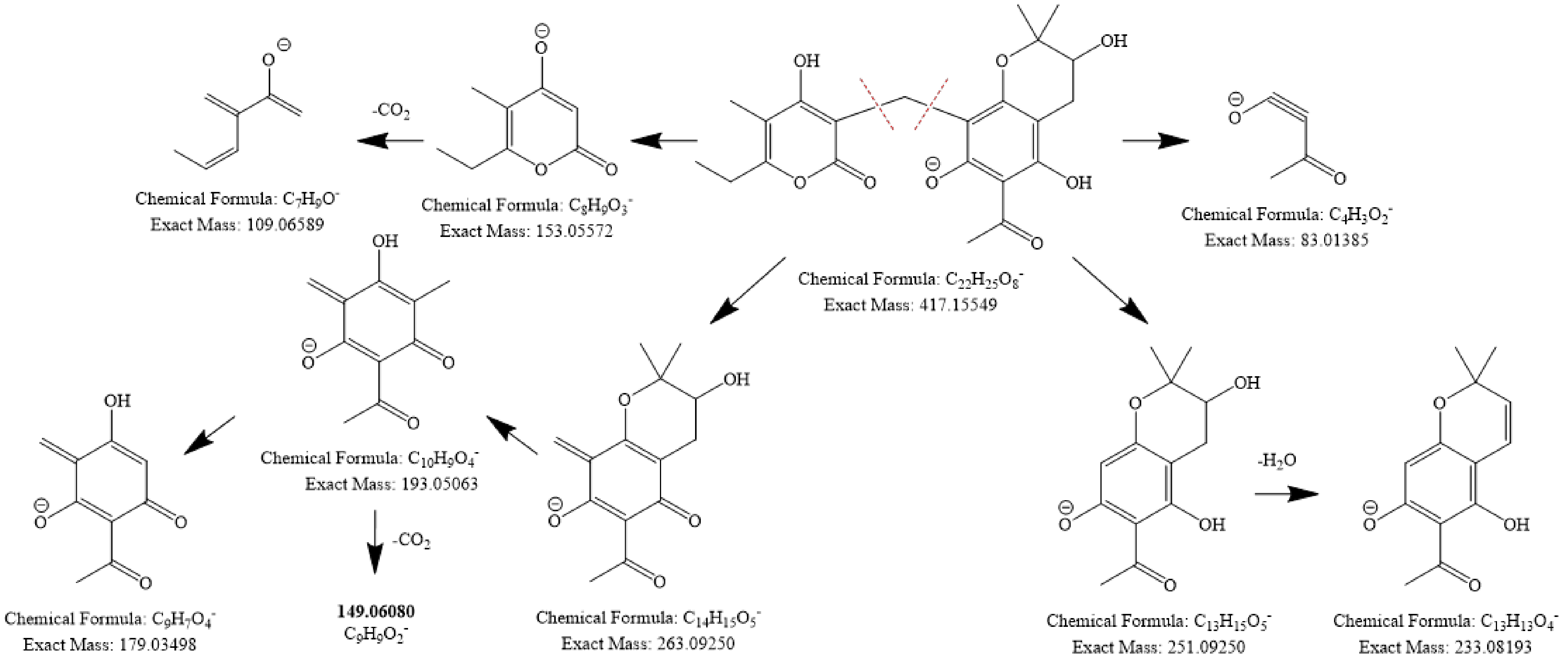

Figure S26. Proposed negative-ion MS/MS fragmentation pathway of compound 30/31.

cmpd_32

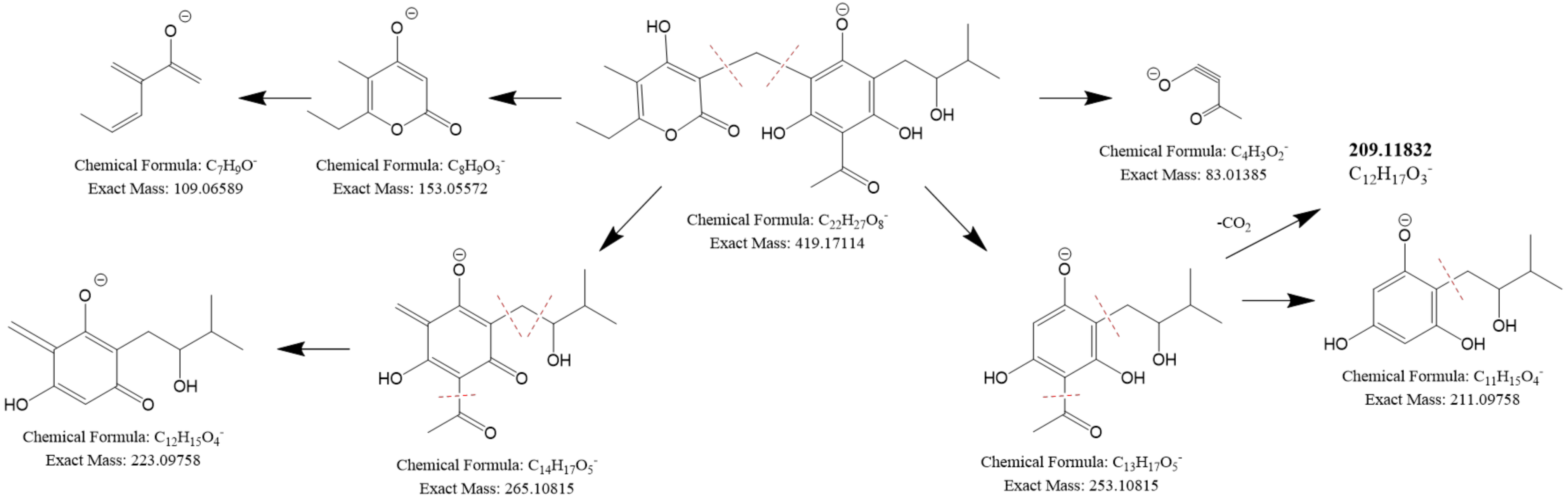

Figure S27. Proposed negative-ion MS/MS fragmentation pathway of compound 32.

cmpd_33_34

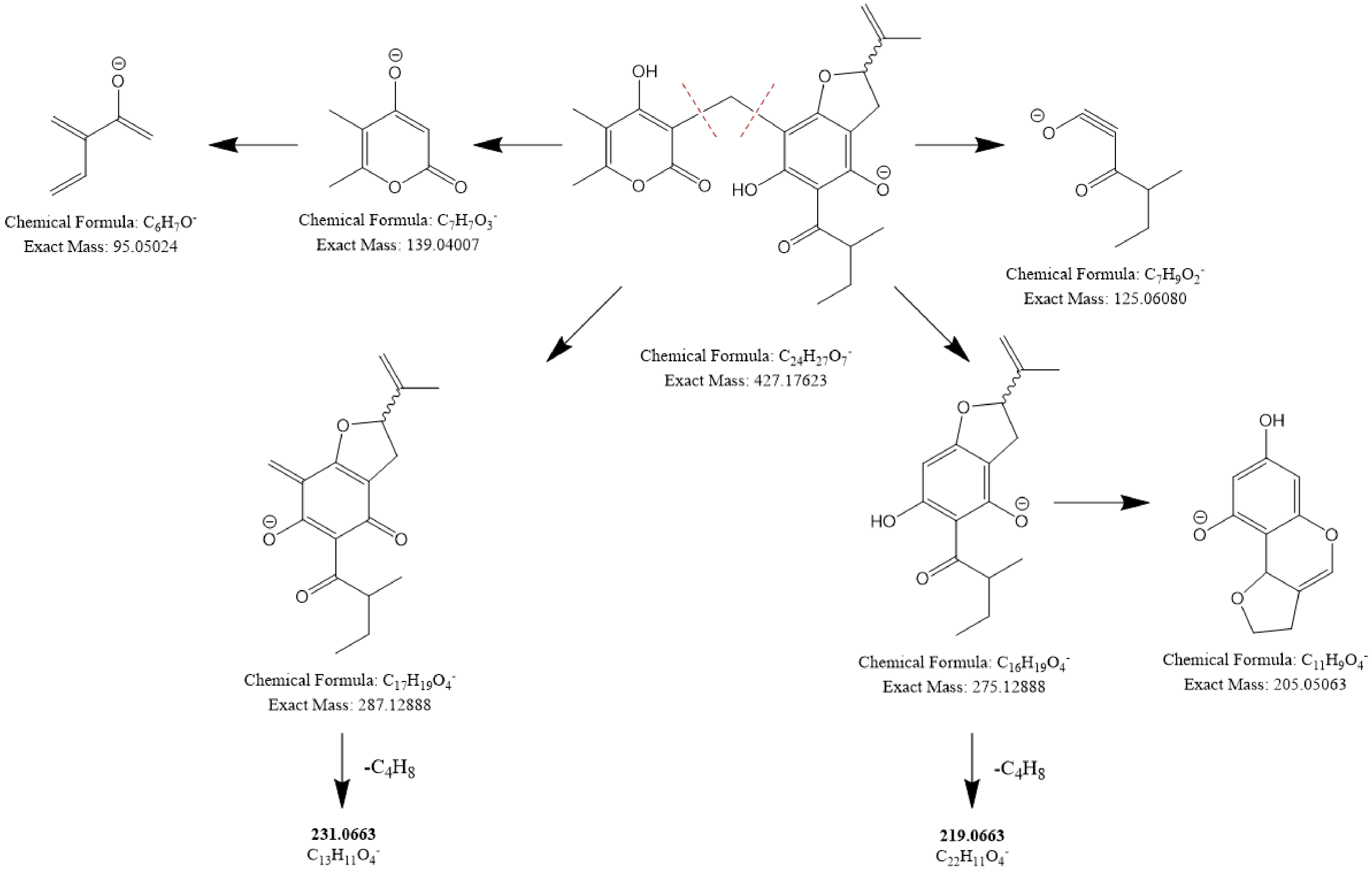

Figure S28. Proposed negative-ion MS/MS fragmentation pathway of compound 33/34.

cmpd_35

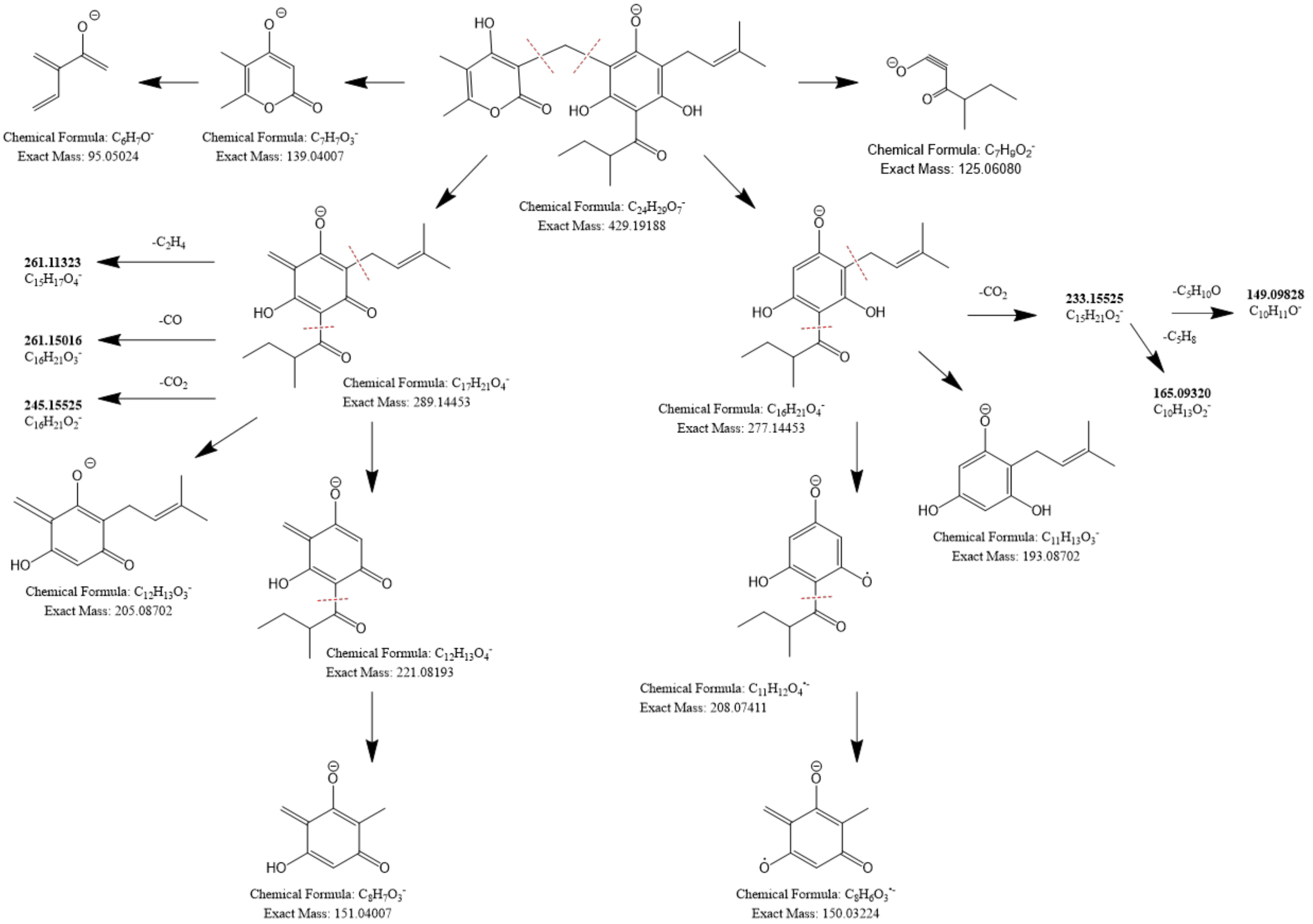

Figure S29. Proposed negative-ion MS/MS fragmentation pathway of compound 35.

cmpd_36

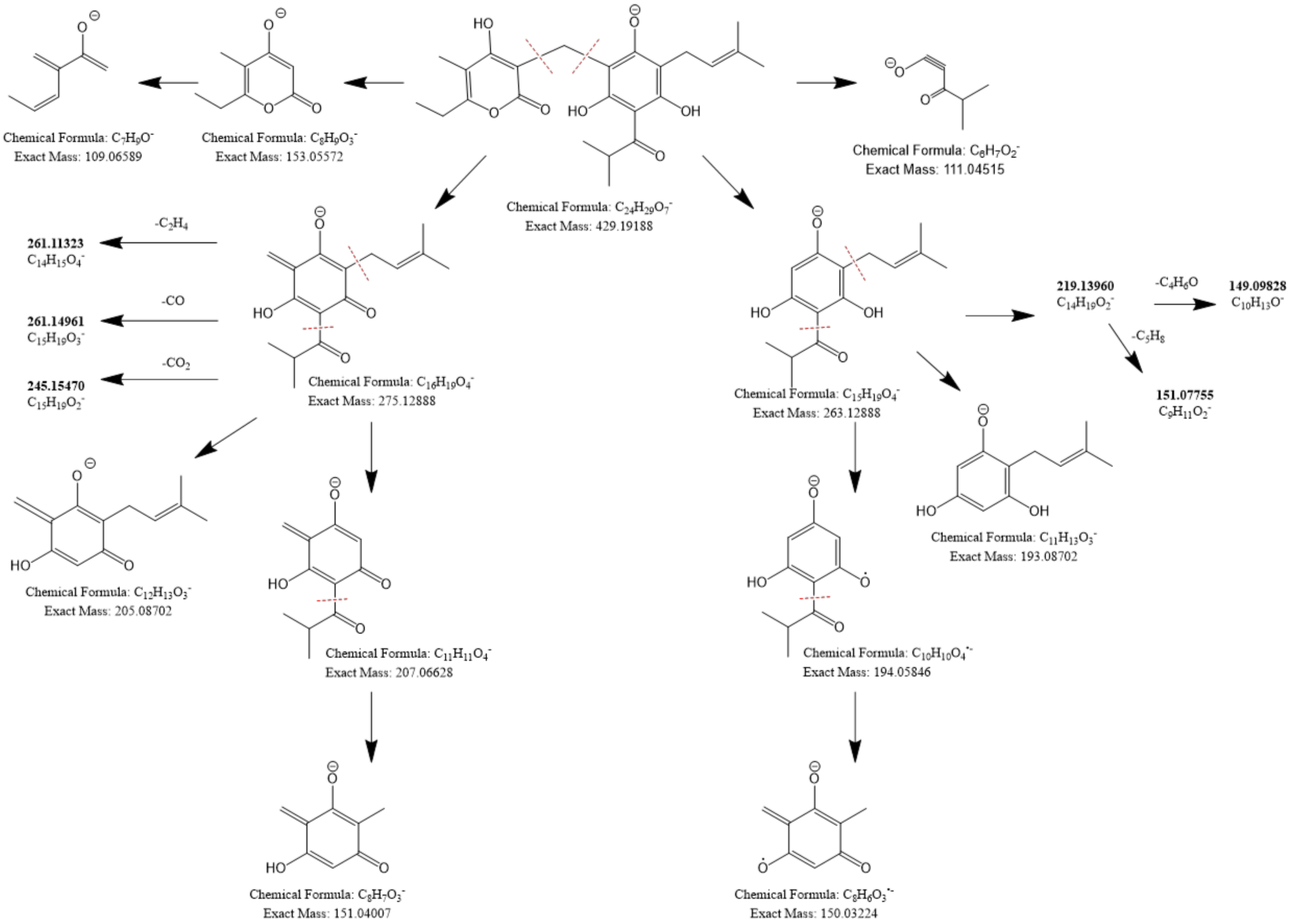

Figure S30. Proposed negative-ion MS/MS fragmentation pathway of compound 36.

cmpd_37

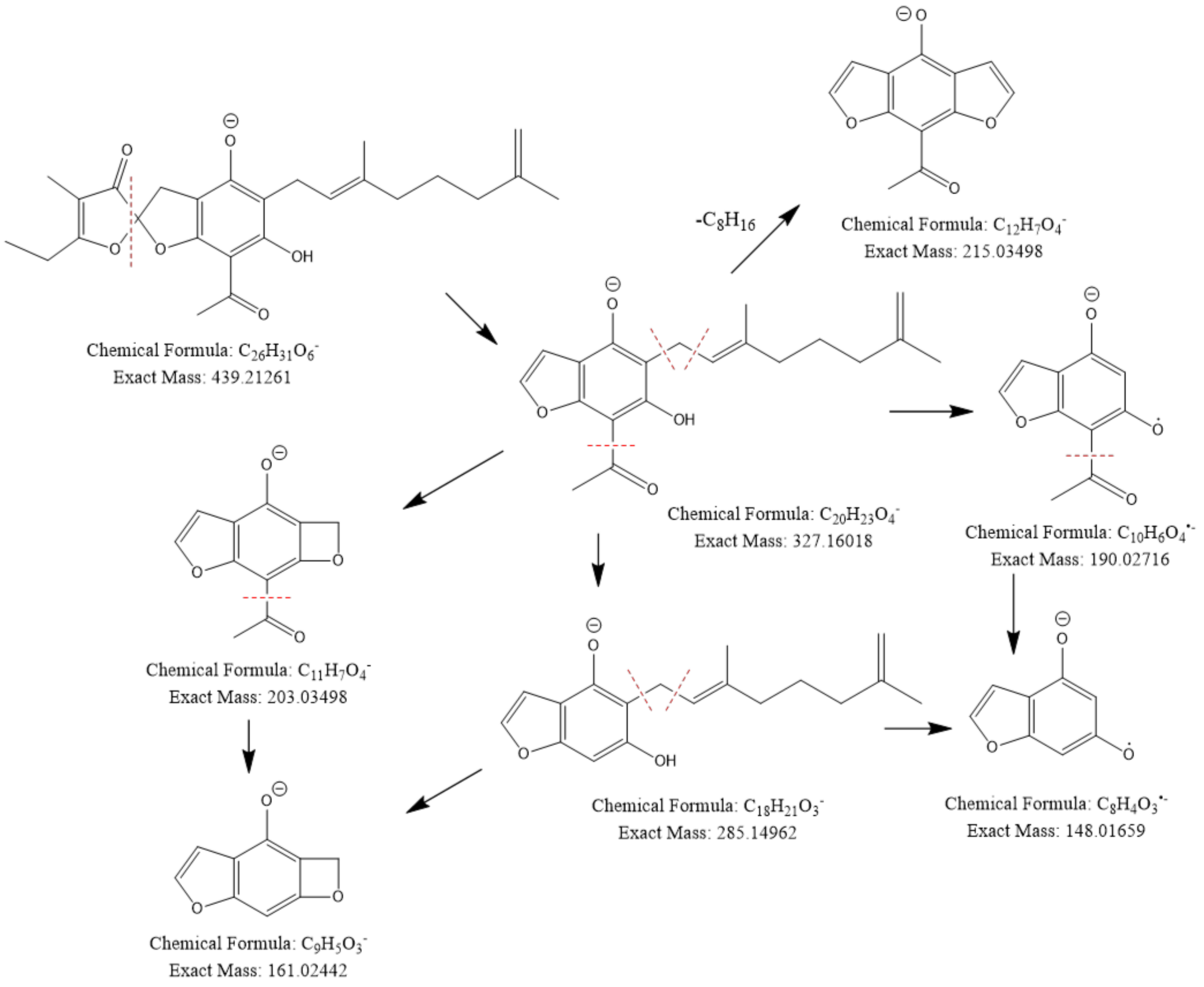

Figure S31. Proposed negative-ion MS/MS fragmentation pathway of compound 37.

cmpd_38_39

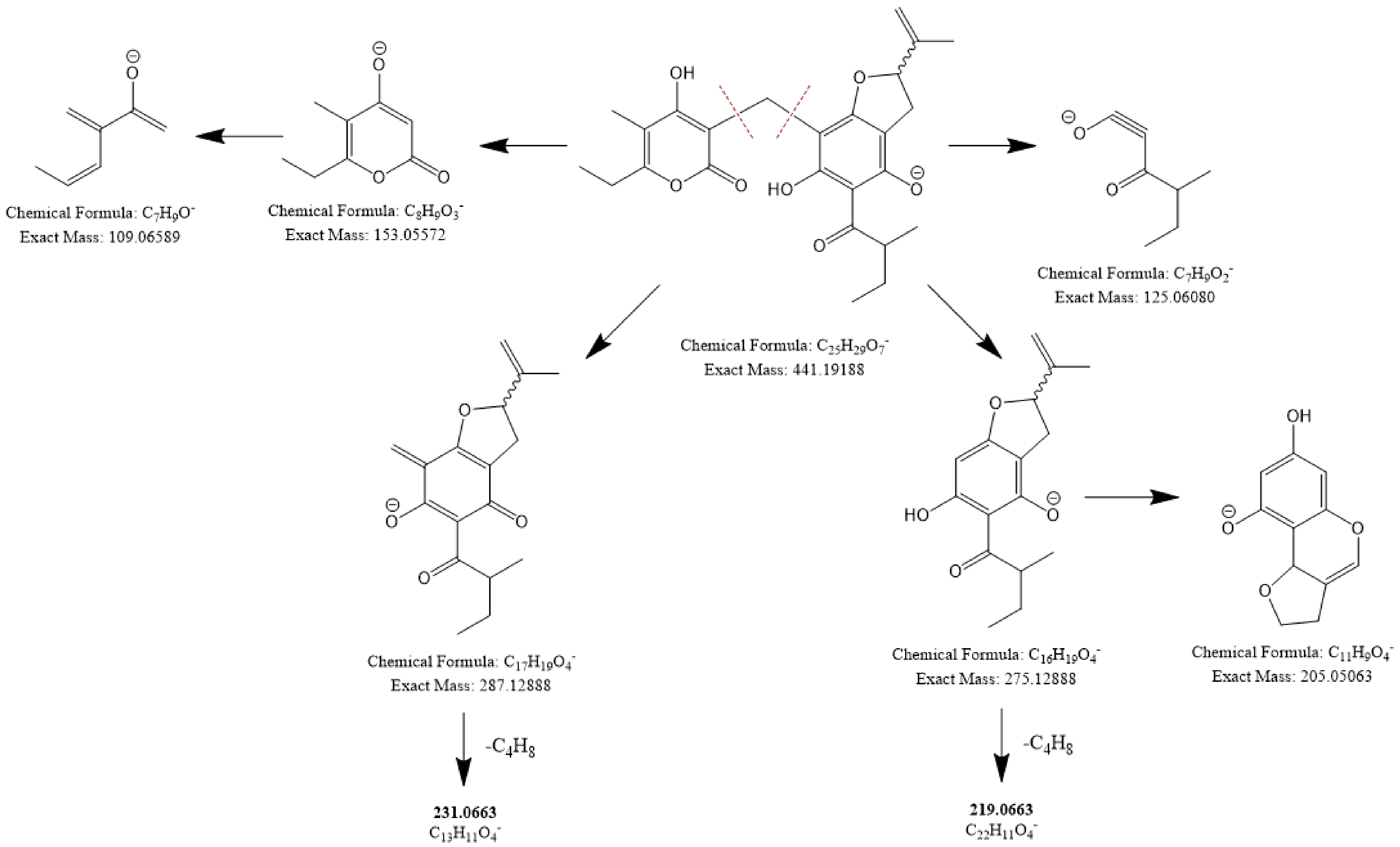

Figure S32. Proposed negative-ion MS/MS fragmentation pathway of compound 38/39.

cmpd_40

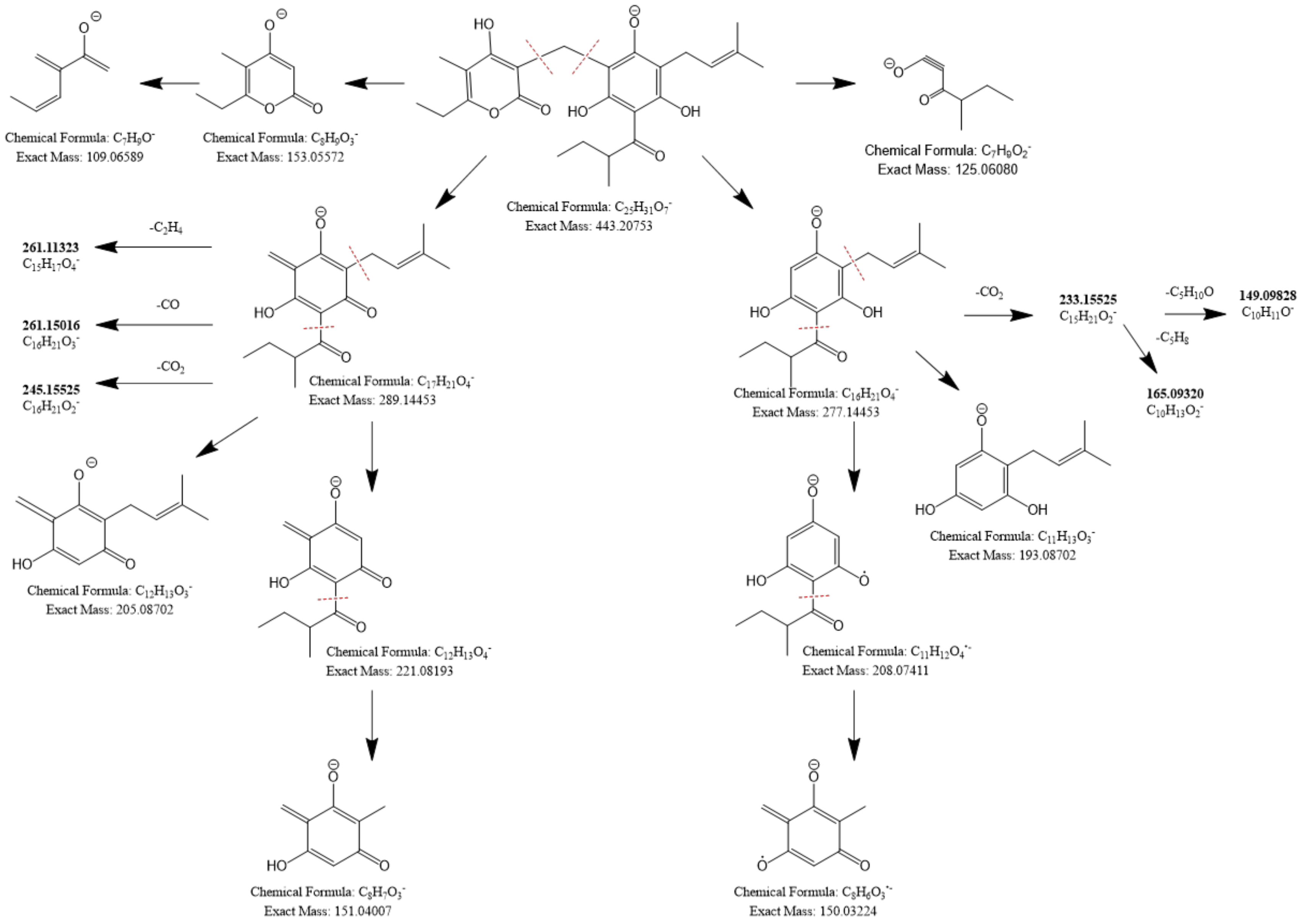

Figure S33. Proposed negative-ion MS/MS fragmentation pathway of compound 40.

cmpd_41_61

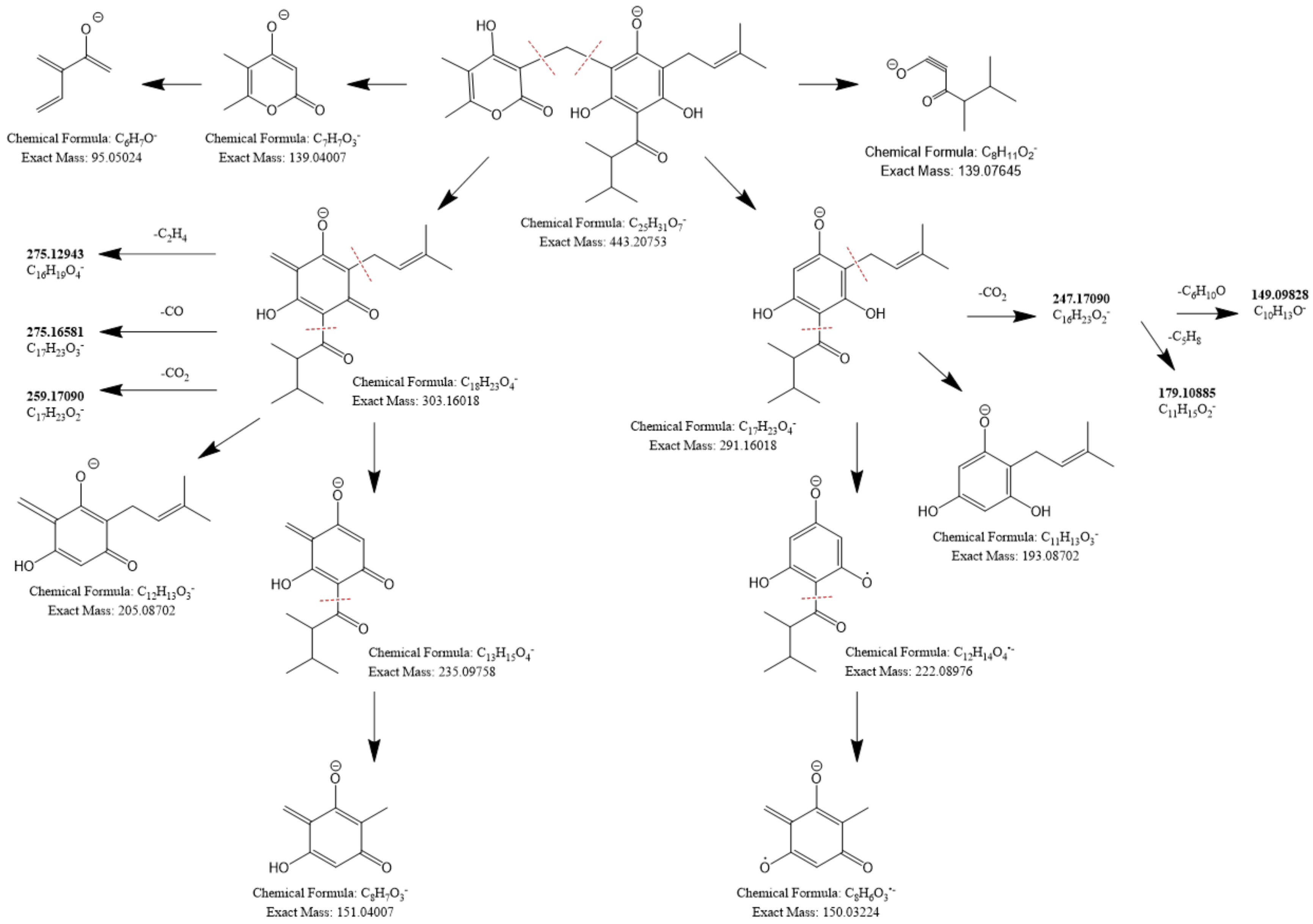

Figure S34. Proposed negative-ion MS/MS fragmentation pathway of compound 41/61.

cmpd_42

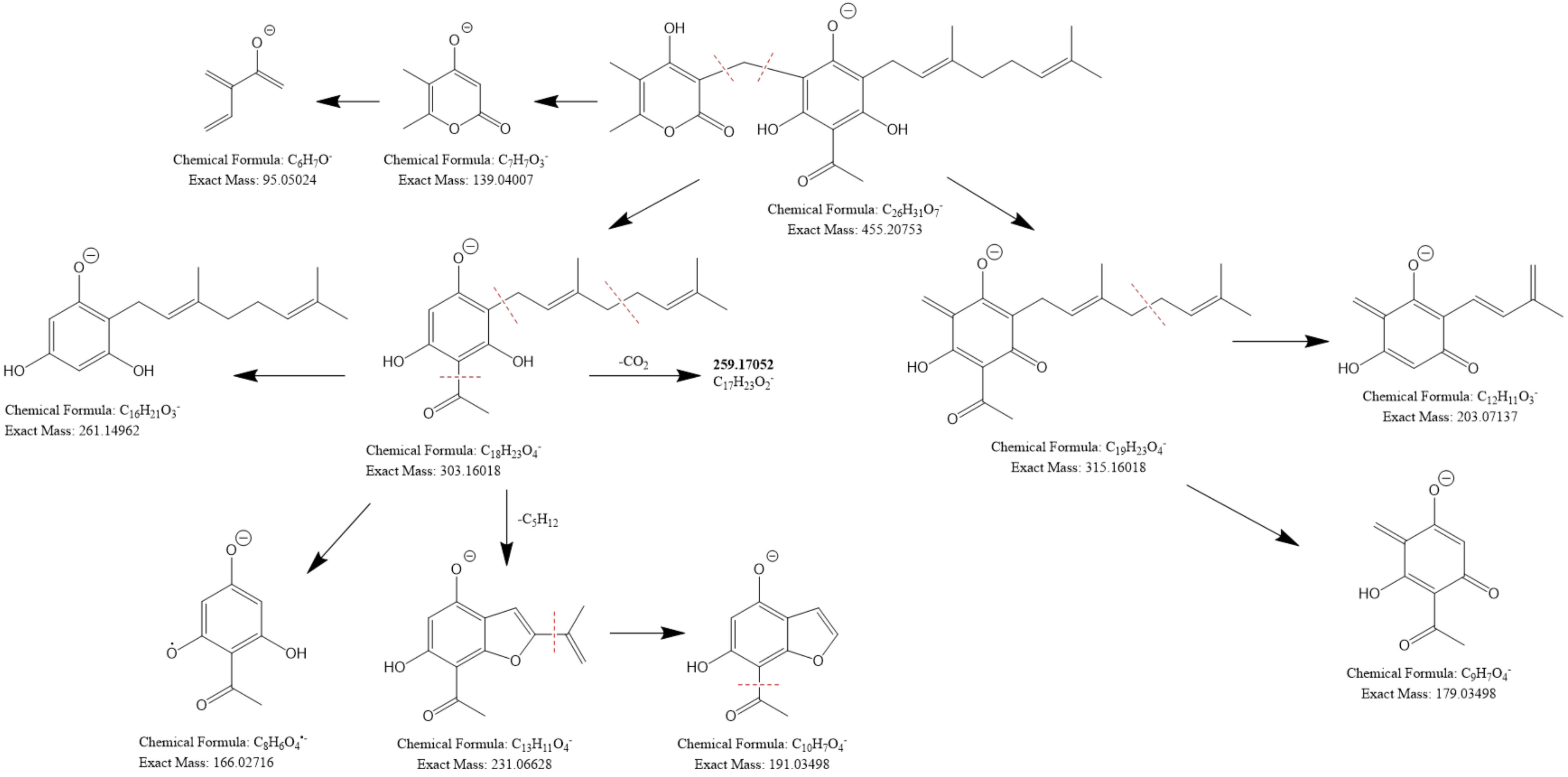

Figure S35. Proposed negative-ion MS/MS fragmentation pathway of compound 42.

cmpd_43_44

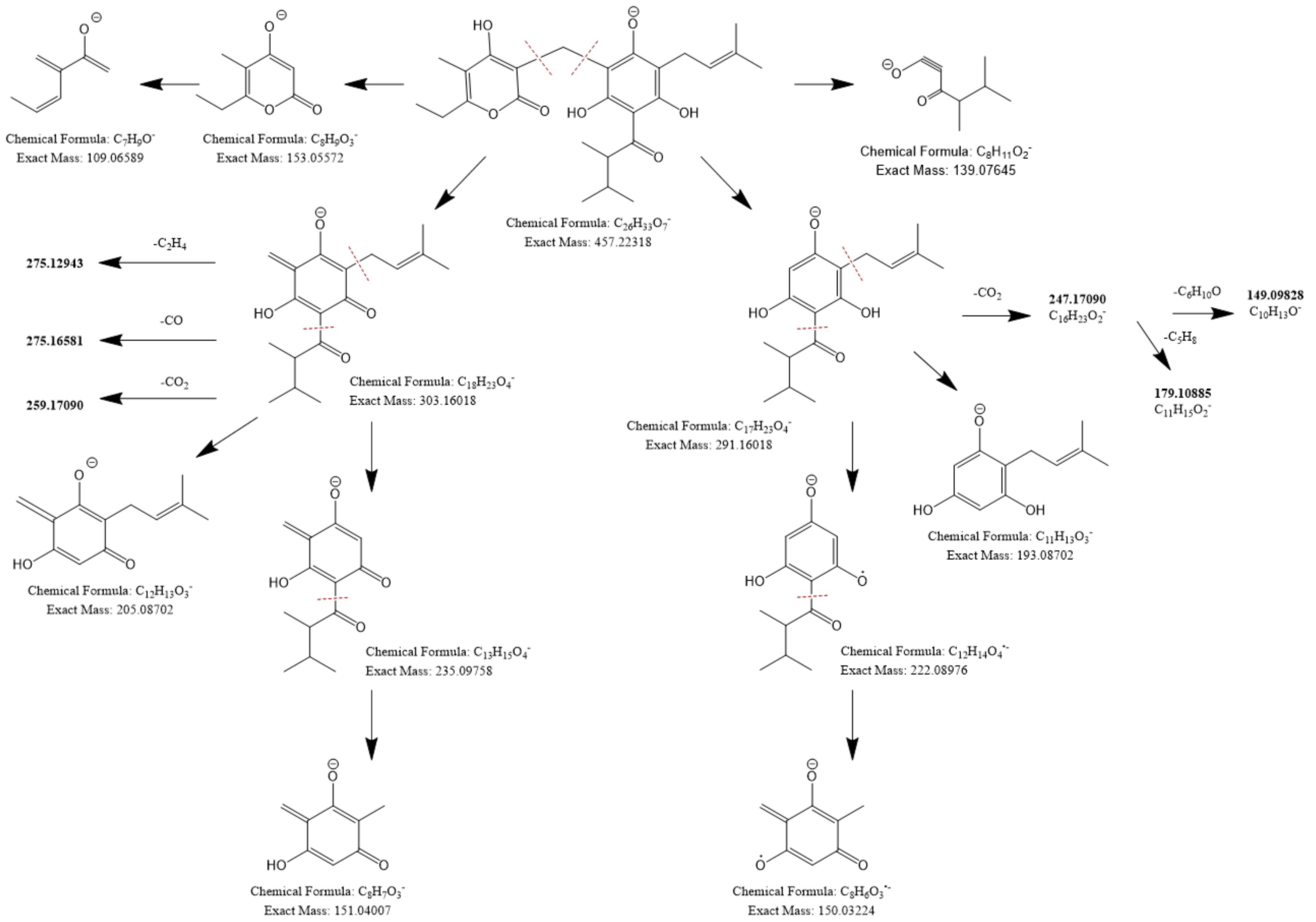

Figure S36. Proposed negative-ion MS/MS fragmentation pathway of compound 43/44.

cmpd_45

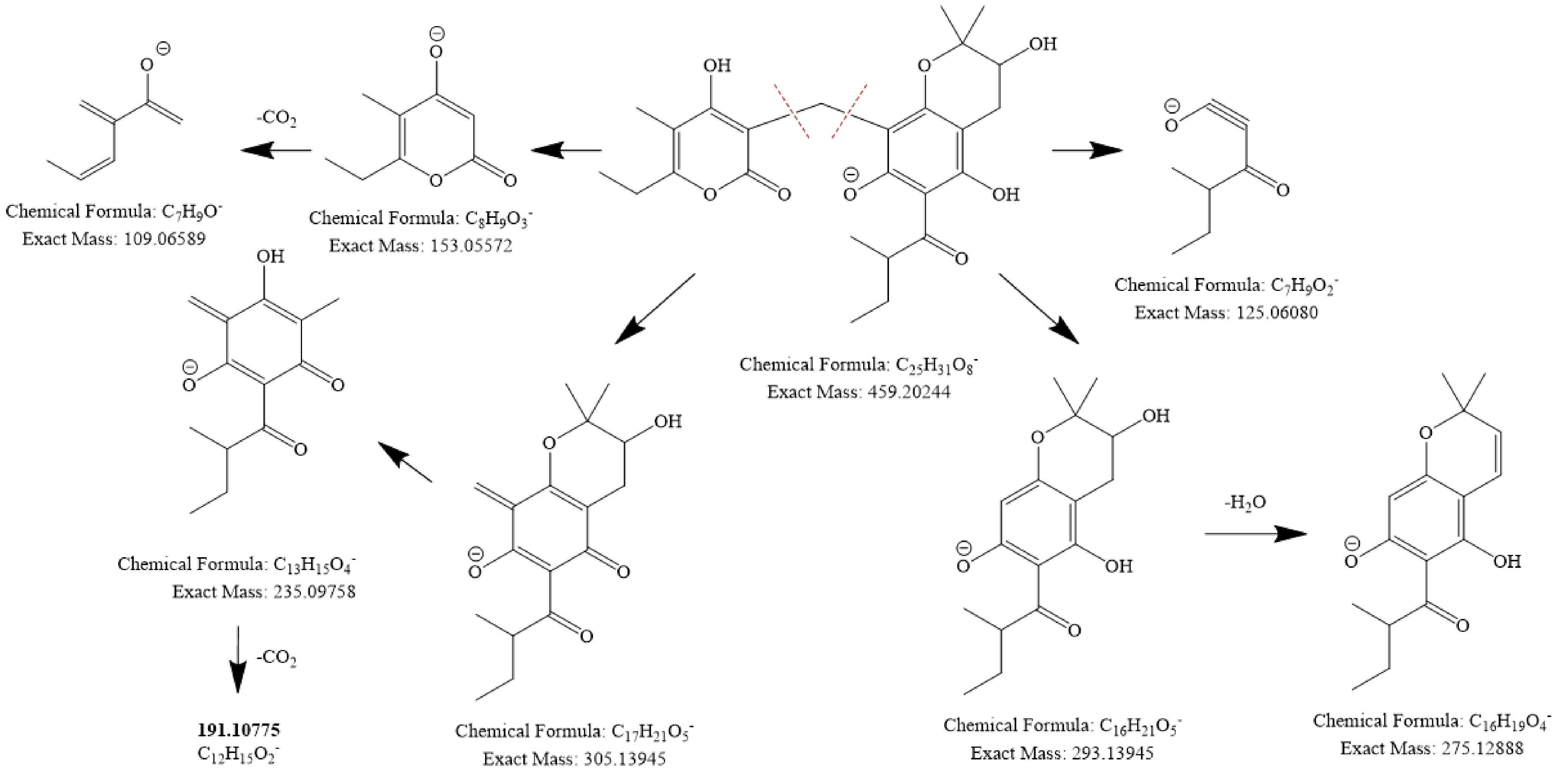

Figure S37. Proposed negative-ion MS/MS fragmentation pathway of compound 45.

cmpd_46

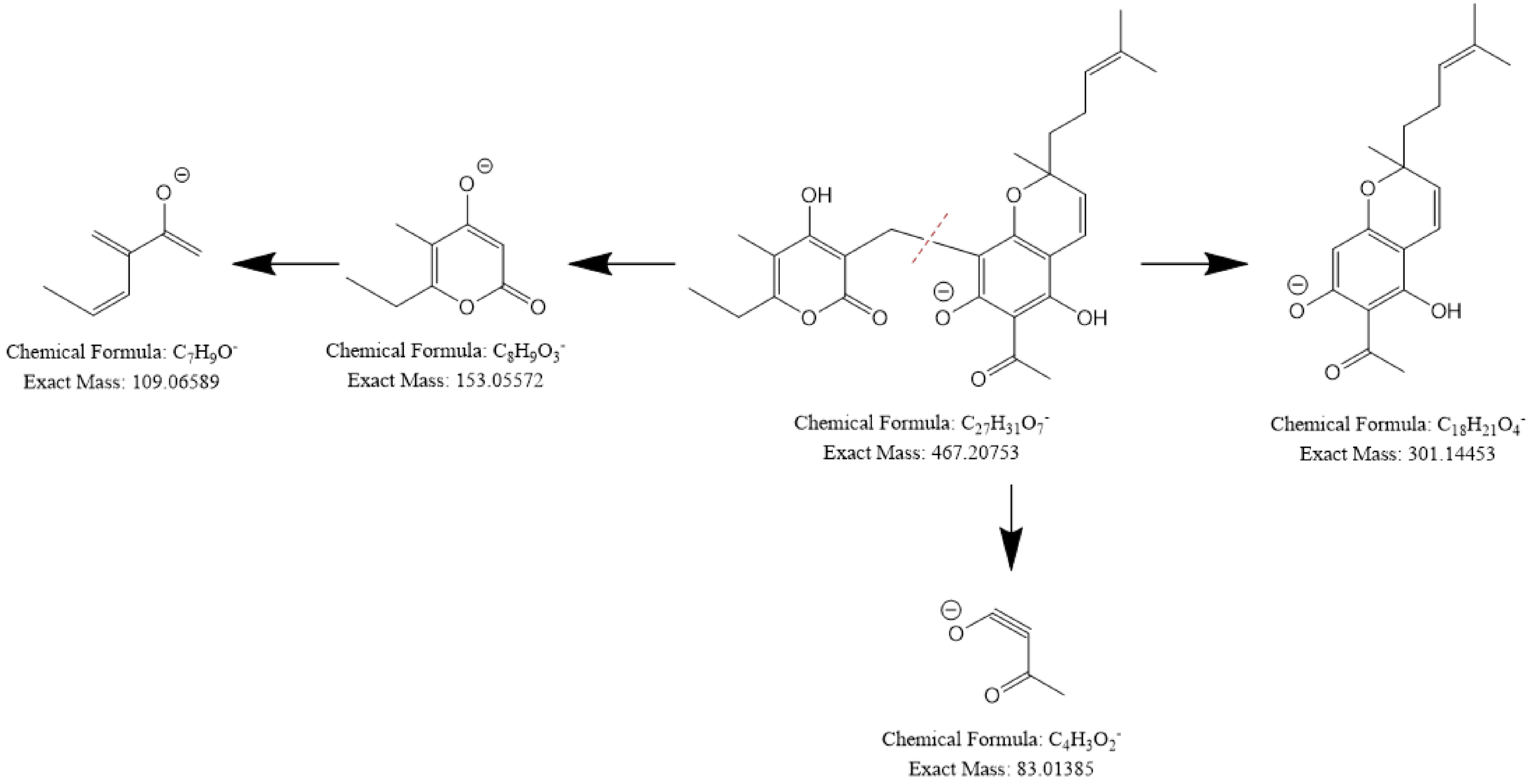

Figure S38. Proposed negative-ion MS/MS fragmentation pathway of compound 46.

cmpd_47

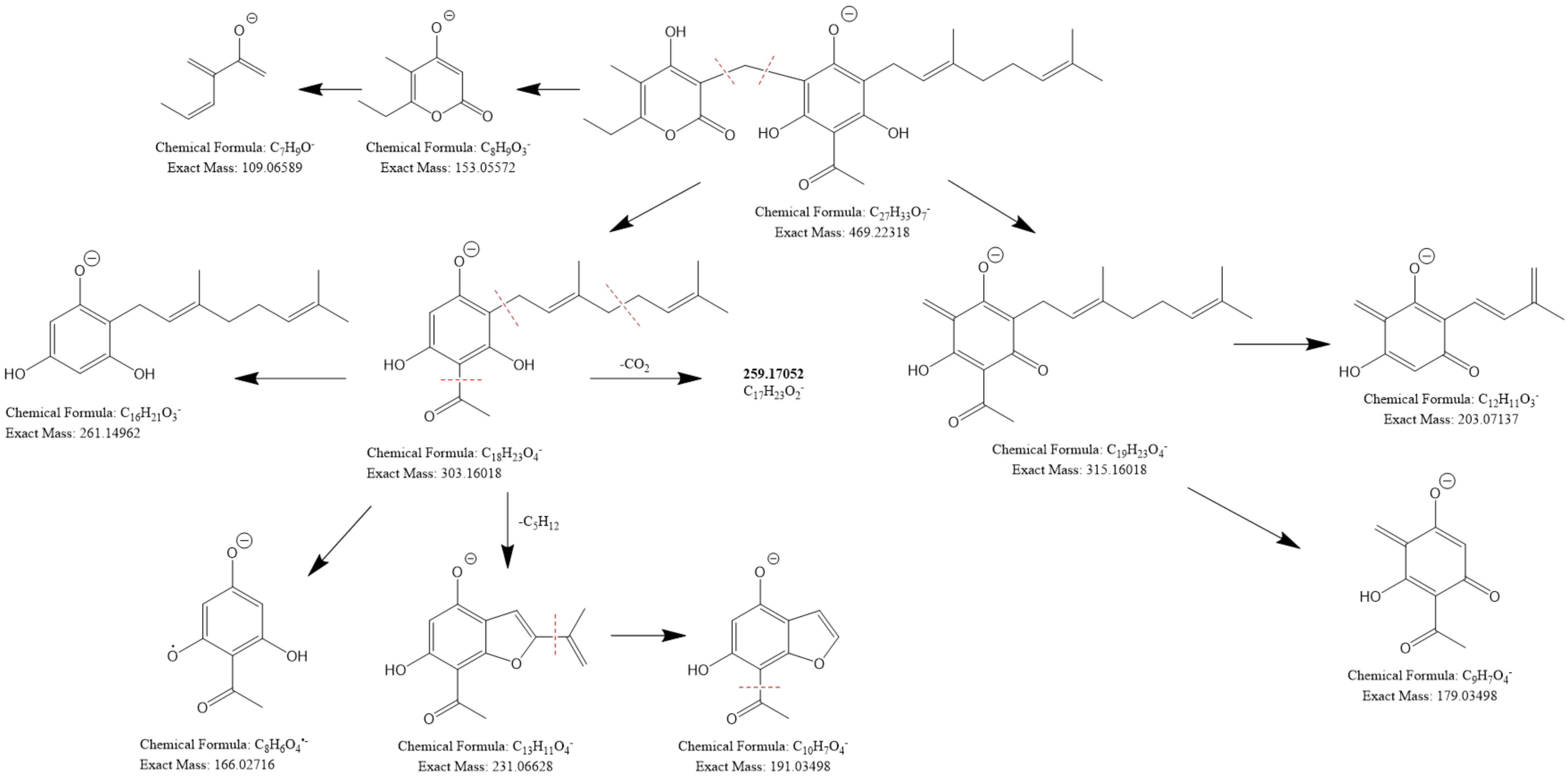

Figure S39. Proposed negative-ion MS/MS fragmentation pathway of compound 47.

cmpd_48

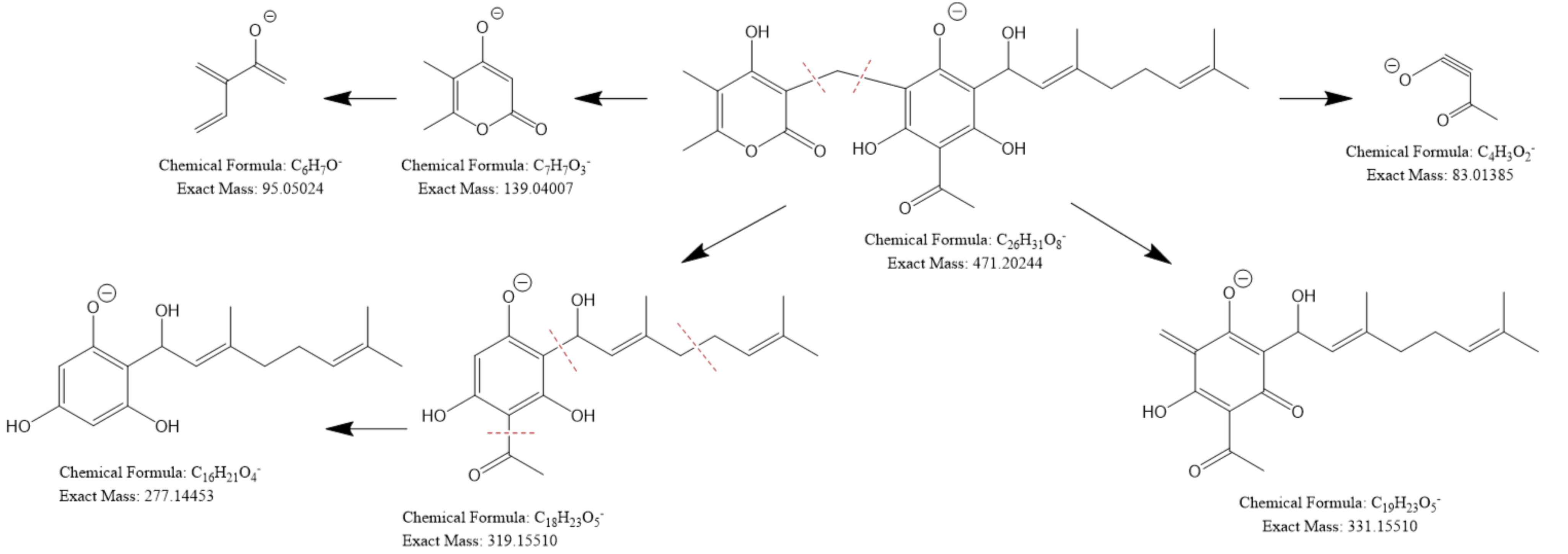

Figure S40. Proposed negative-ion MS/MS fragmentation pathway of compound 48.

cmpd_49_56

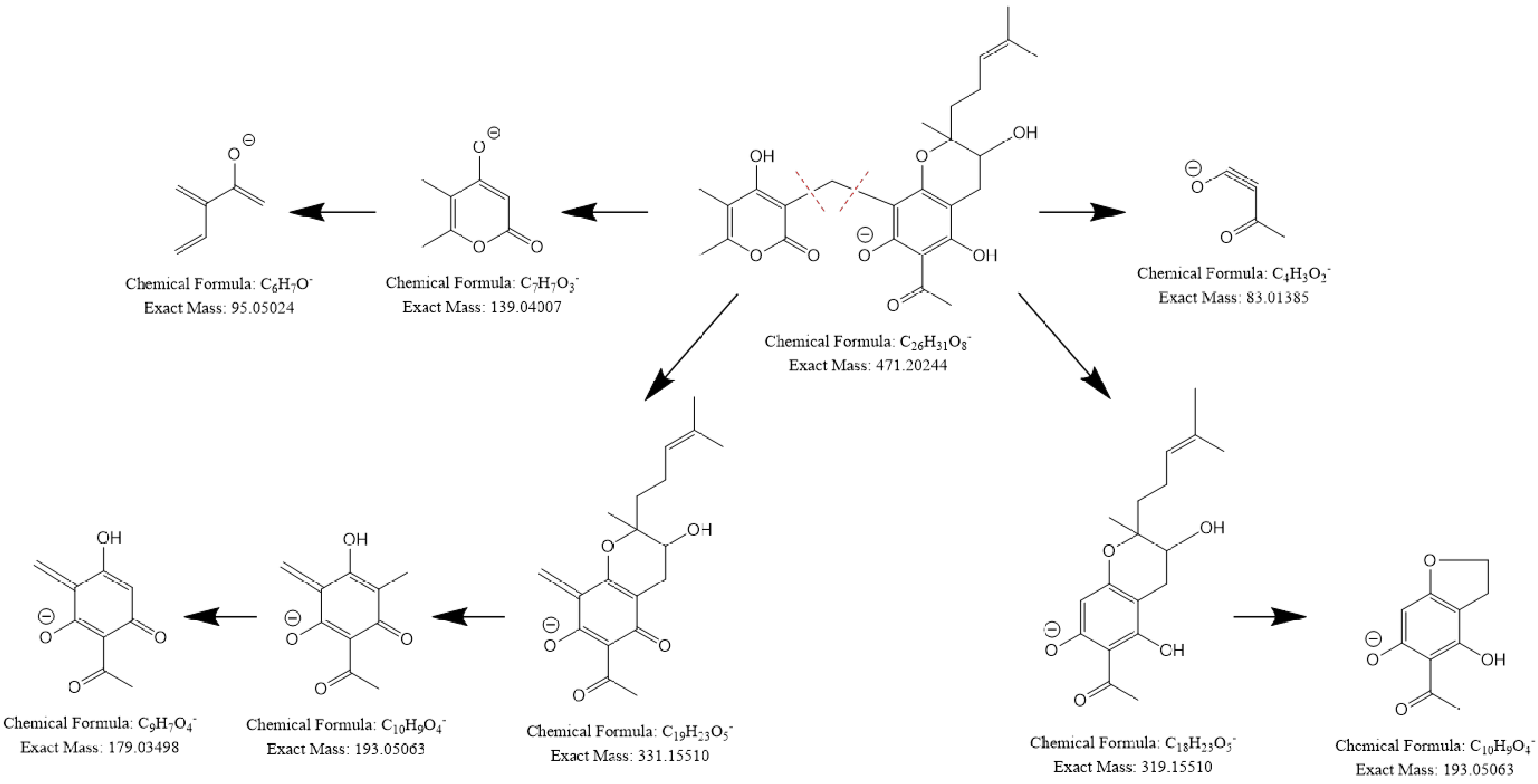

Figure S41. Proposed negative-ion MS/MS fragmentation pathway of compound 49/56.

cmpd_50

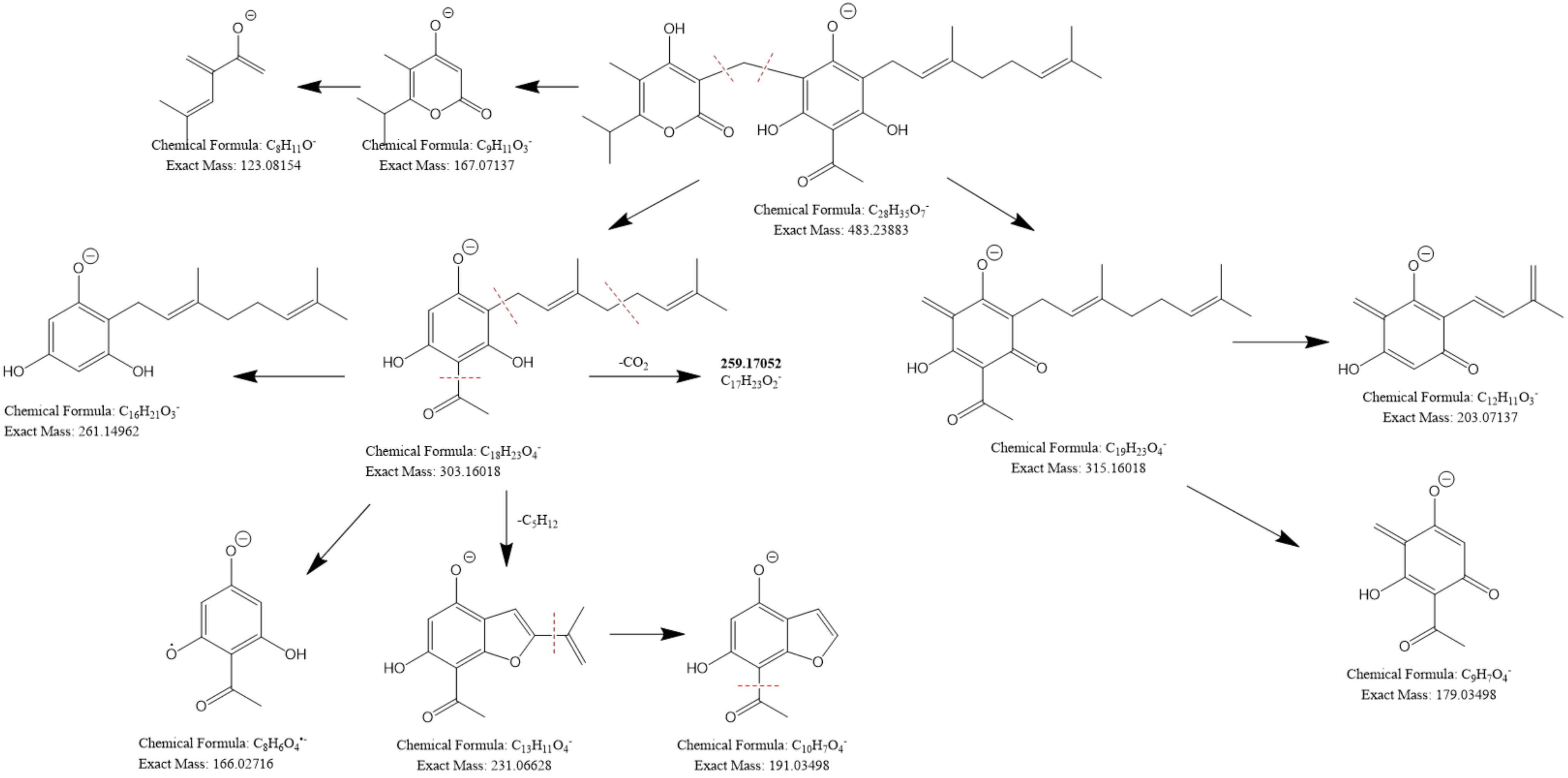

Figure S42. Proposed negative-ion MS/MS fragmentation pathway of compound 50.

cmpd_51_52

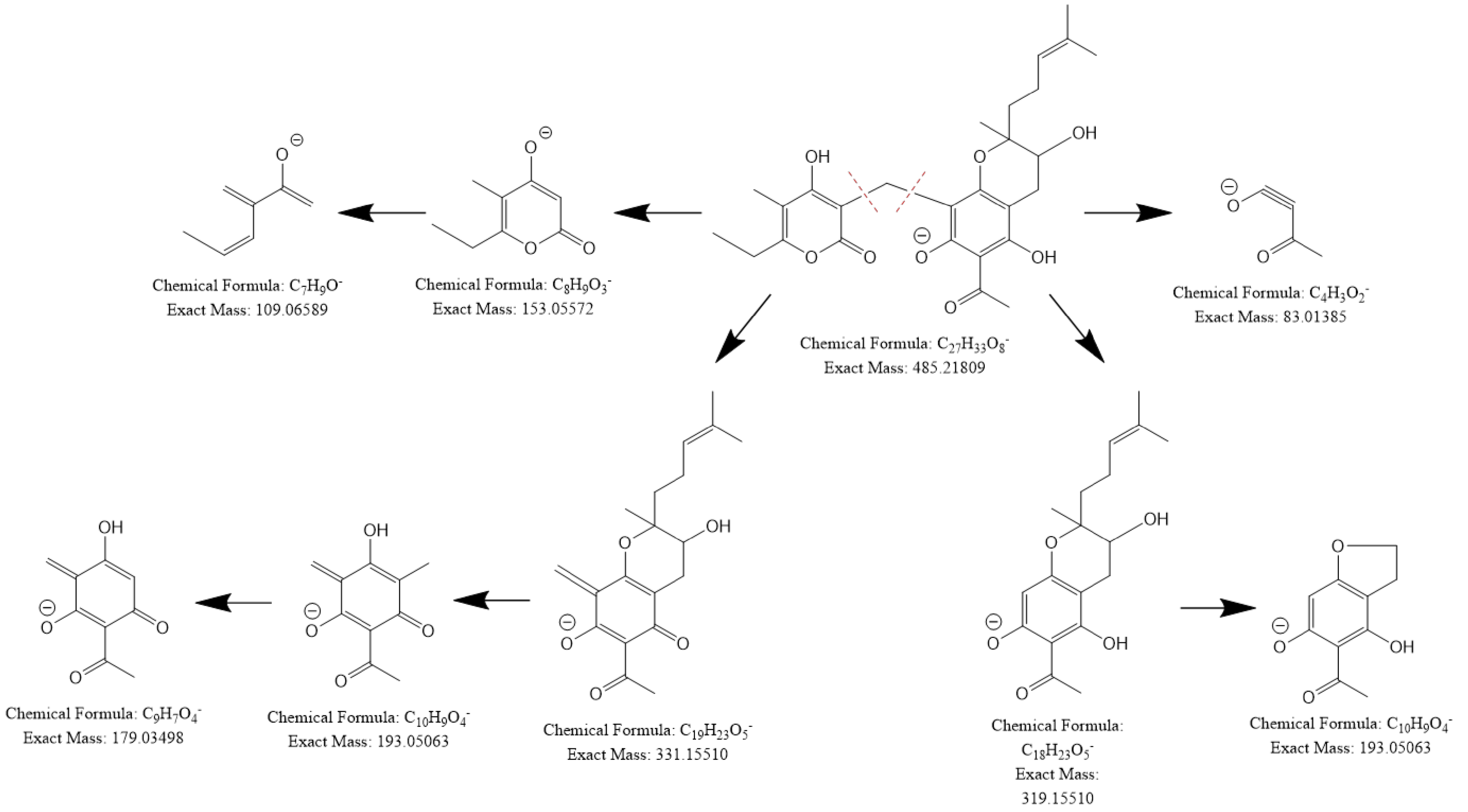

Figure S43. Proposed negative-ion MS/MS fragmentation pathway of compound 51/52.

cmpd_53

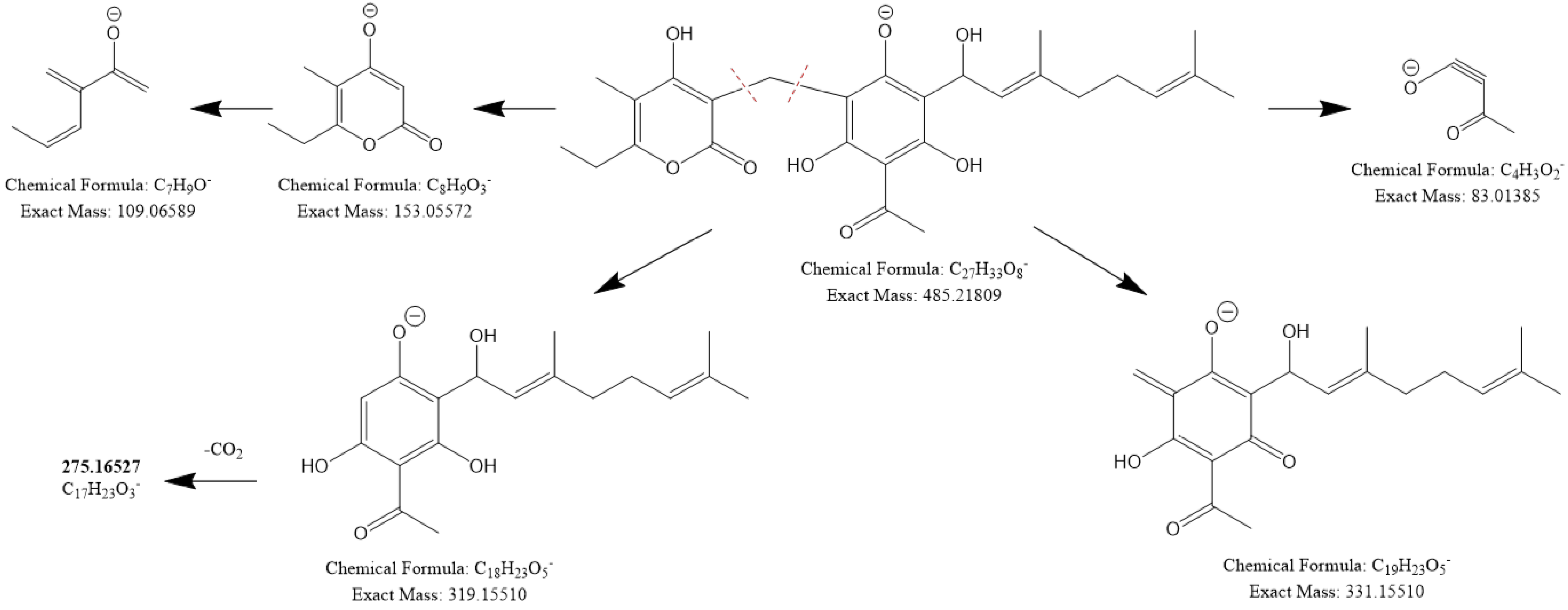

Figure S44. Proposed negative-ion MS/MS fragmentation pathway of compound 53.

cmpd_54

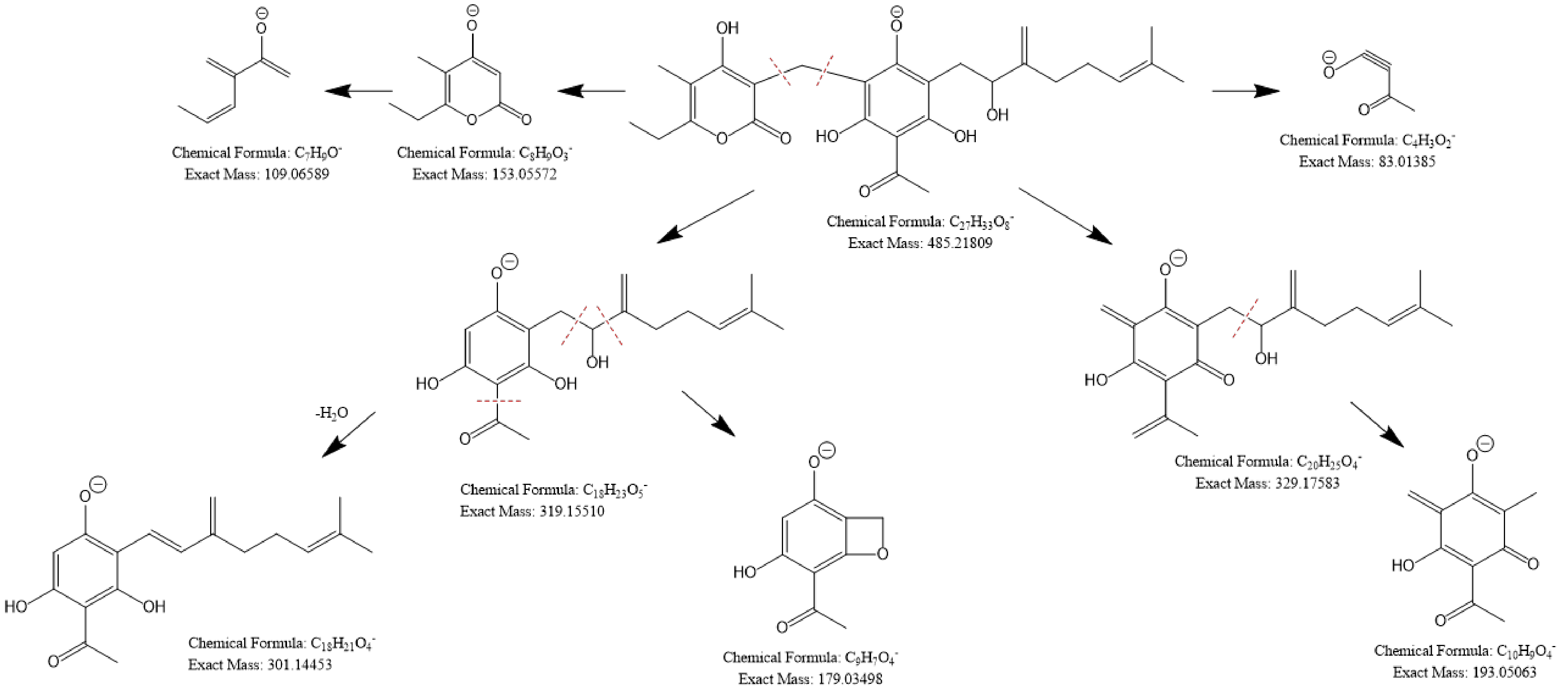

Figure S45. Proposed negative-ion MS/MS fragmentation pathway of compound 54.

cmpd_55

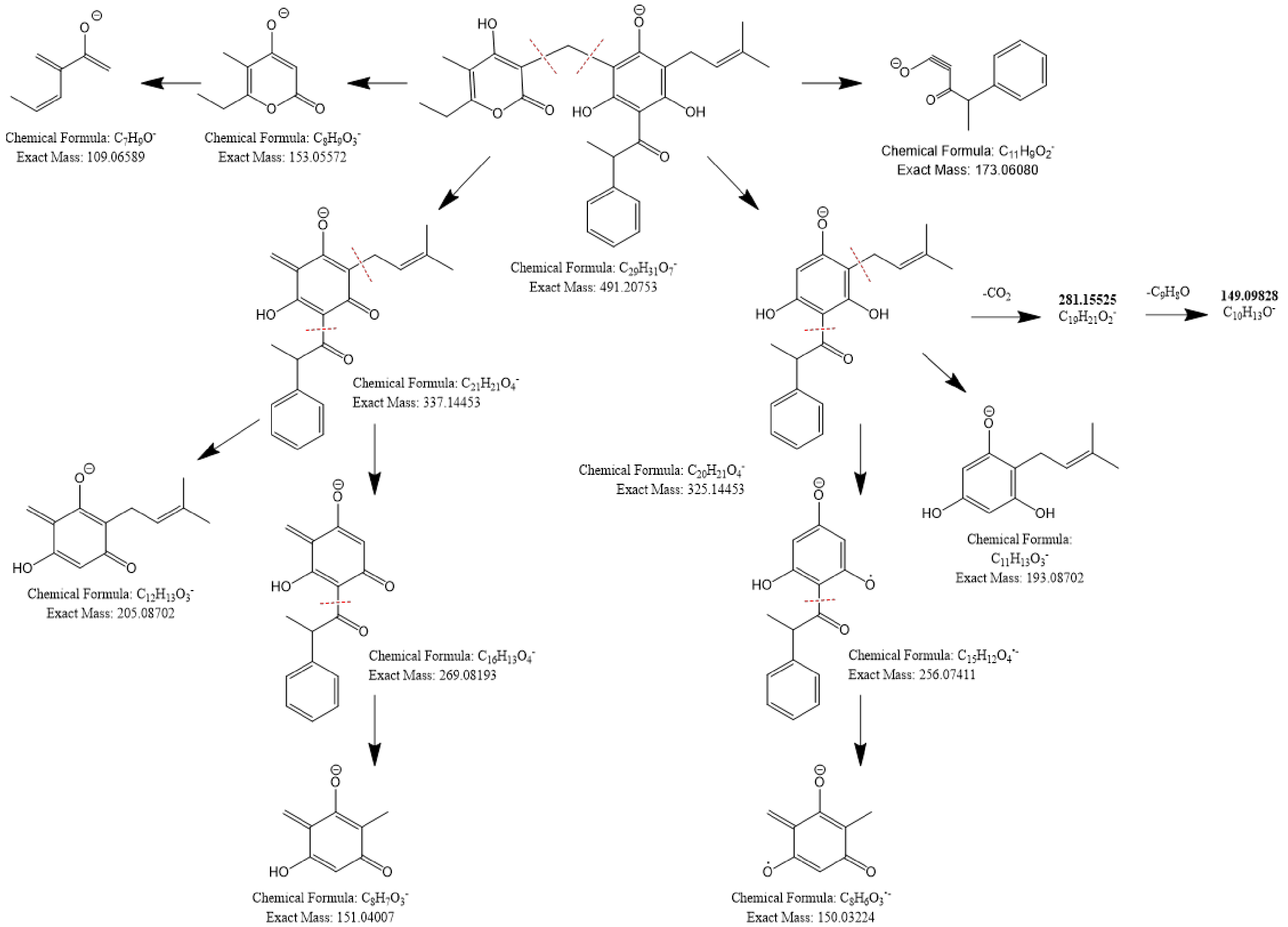

Figure S46. Proposed negative-ion MS/MS fragmentation pathway of compound 55.

cmpd_59_60

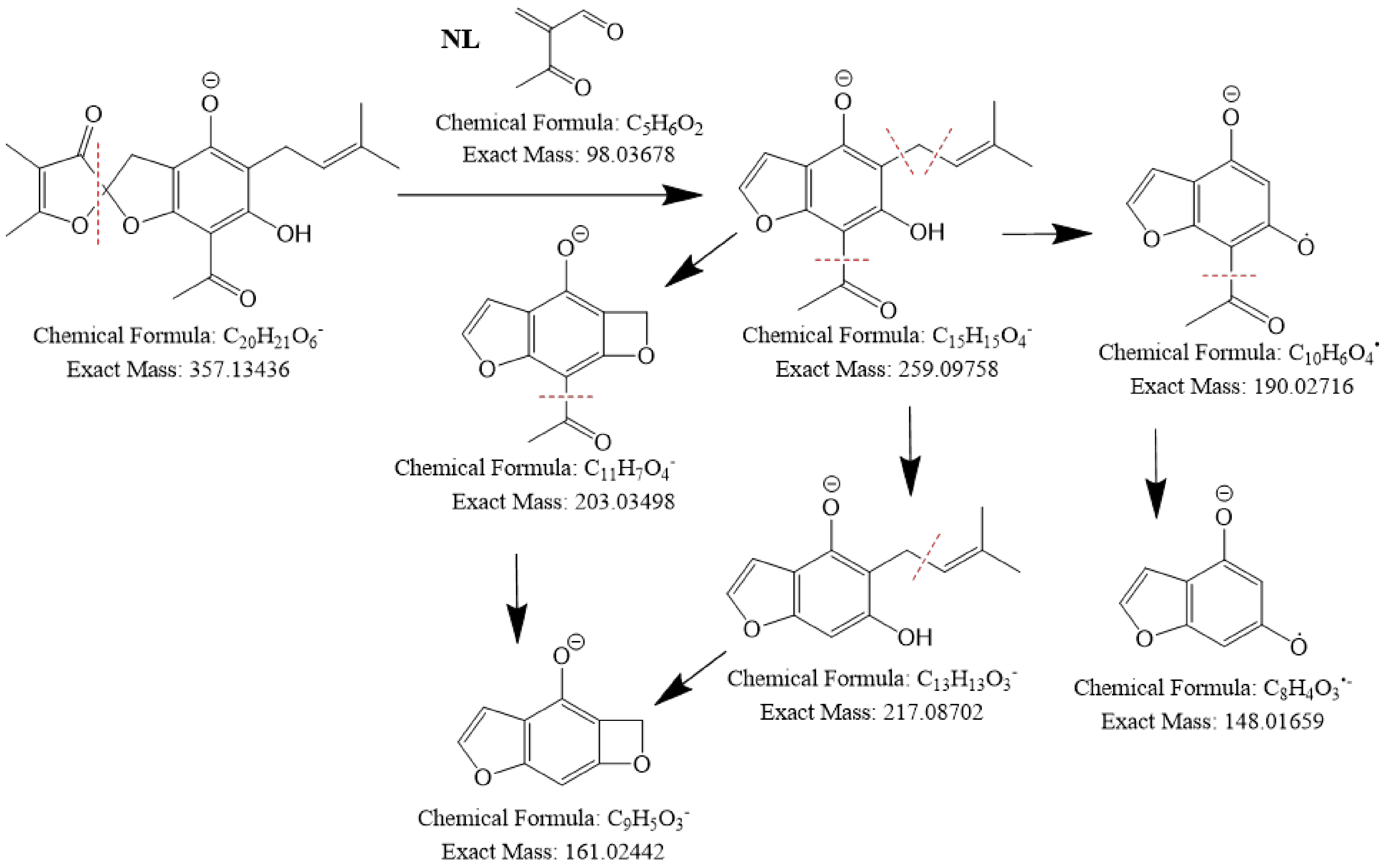

Figure S47. Proposed negative-ion MS/MS fragmentation pathway of compound 59/60.
