# Supplementary material for: Comprehensive UHPLC–Orbitrap–MS Profiling of Phloroglucinol α-Pyrones in Helichrysum italicum (Roth) G. Don (Asteraceae)
Source: Plants (Basel). 2026 Aug 27;15(17):2619. doi: 10.3390/plants15172619 (PMC13567118; doi:10.3390/plants15172619)
Supplement: Supplementary file 1 [file plants-15-02619-s001.zip › Table_S1.pdf]

Table S1. Distribution of the diagnostic fragment ions across the structural subclasses of phloroglucinol α-pyrone derivatives detected in *Helichrysum italicum*. Entries give the number of compounds in which the ion was detected relative to the number of compounds in the subclass; a dash denotes no detection above the reporting threshold of Table A1 (above 1% of base peak).

m/z	Formula	Mono (1)	Di (2)	3-NA (9)	3-Pre (14)	3-OHP (2)	3-OHDP (2)	3-Ger (3)	3-OHG (3)	BF (8)	BP (10)	SK (5)	Specificity
137.0244	$C_7H_5O_3^-$	–	–	9/9	–	–	–	2/3	1/3	–	4/10	–	Intensity-resolved
125.0244	$C_6H_5O_3^-$	–	–	9/9	–	–	–	–	–	–	–	–	Exclusive
81.0346	$C_5H_5O^-$	–	–	9/9	2/14	2/2	2/2	2/3	3/3	4/8	–	–	Non-specific
163.0031	$C_8H_3O_4^-$	–	–	9/9	–	–	–	–	–	–	-	–	Exclusive
205.0870	$C_{12}H_{13}O_3^-$	–	–	–	14/14	–	–	–	–	–	–	–	Exclusive
193.0870	$C_{11}H_{13}O_3^-$	–	–	–	14/14	–	–	–	–	–	–	–	Exclusive
221.0819	$C_{12}H_{13}O_4^-$	–	1/2	4/9	–	0/2	–	–	–	–	–	–	Precursor-dependent [a]
209.0819	$C_{11}H_{13}O_4^-$	–	–	4/9	–	0/2	–	–	–	–	–	–	Precursor-dependent [a]
191.0714	$C_{11}H_{11}O_3^-$	–	–	–	–	1/2	–	–	–	4/8	–	–	Non-specific
223.0976	$C_{12}H_{15}O_4^-$	–	–	4/9	–	–	2/2	–	–	–	–	–	Precursor-dependent
211.0976	$C_{11}H_{15}O_4^-$	–	–	–	–	–	2/2	–	–	–	–	–	Exclusive
203.0714	$C_{12}H_{11}O_3^-$	–	–	2/9	–	–	–	2/3	–	–	–	–	Precursor-dependent
147.0815	$C_{10}H_{11}O^-$	–	–	–	–	1/2	–	–	–	4/8	–	–	Partial coverage [b]
193.0506	$C_{10}H_9O_4^-$	–	–	–	–	2/2	–	–	1/3	–	8/10	–	Partial coverage
149.0608	$C_9H_9O_2^-$	–	–	–	–	–	–	–	–	4/8	5/10	–	Intensity-resolved
215.0350	$C_{12}H_7O_4^-$	–	–	–	–	–	–	–	–	–	–	4/5	Exclusive [c]
229.0506	$C_{13}H_9O_4^-$	–	–	–	–	–	–	–	–	–	–	2/5	Exclusive [c]
243.0663	$C_{14}H_{11}O_4^-$	–	–	–	–	–	–	–	–	–	–	5/5	Exclusive

Subclass abbreviations: Mono, monopyrone; Di, dipyrone; 3-NA, 3-non-alkylated; 3-Pre, 3-prenyl; 3-OHP, 3-(hydroxyprenyl); 3-OHDP, 3-(hydroxydihydroprenyl); 3-Ger, 3-geranyl; 3-OHG, 3-(hydroxygeranyl); BF, benzofuran; BP, benzopyran; SK, spiroketal.

[a] Not detected above the reporting threshold in the compounds to which the ion is assigned in Sections 2.4 and 2.5 (m/z 221.0819 and 209.0819, cmpds 24 and 29; m/z 261.1496, cmpds 42 and 50).

[b] Detected in the prenyl-derived benzofurans (cmpds 11, 12, 18, 19) and not in the geranyl-derived benzofurans (cmpds 33, 34, 38, 39).

[c] Reported in all five spiroketals in Section 2.5; m/z 215.0350 was detected above 1% relative intensity in all except cmpd 13, and m/z 229.0506 in cmpds 13 and 37 only.
